# Supplementary material for: Highly selective nickel-catalyzed gem-difluoropropargylation of unactivated alkylzinc reagents
Source: Nat Commun. 2017 Nov 13;8:1460. doi: 10.1038/s41467-017-01540-1 (PMC5684216; doi:10.1038/s41467-017-01540-1)
Supplement: Supplementary file 1 — Supplementary Information [file 41467_2017_1540_MOESM1_ESM.pdf]

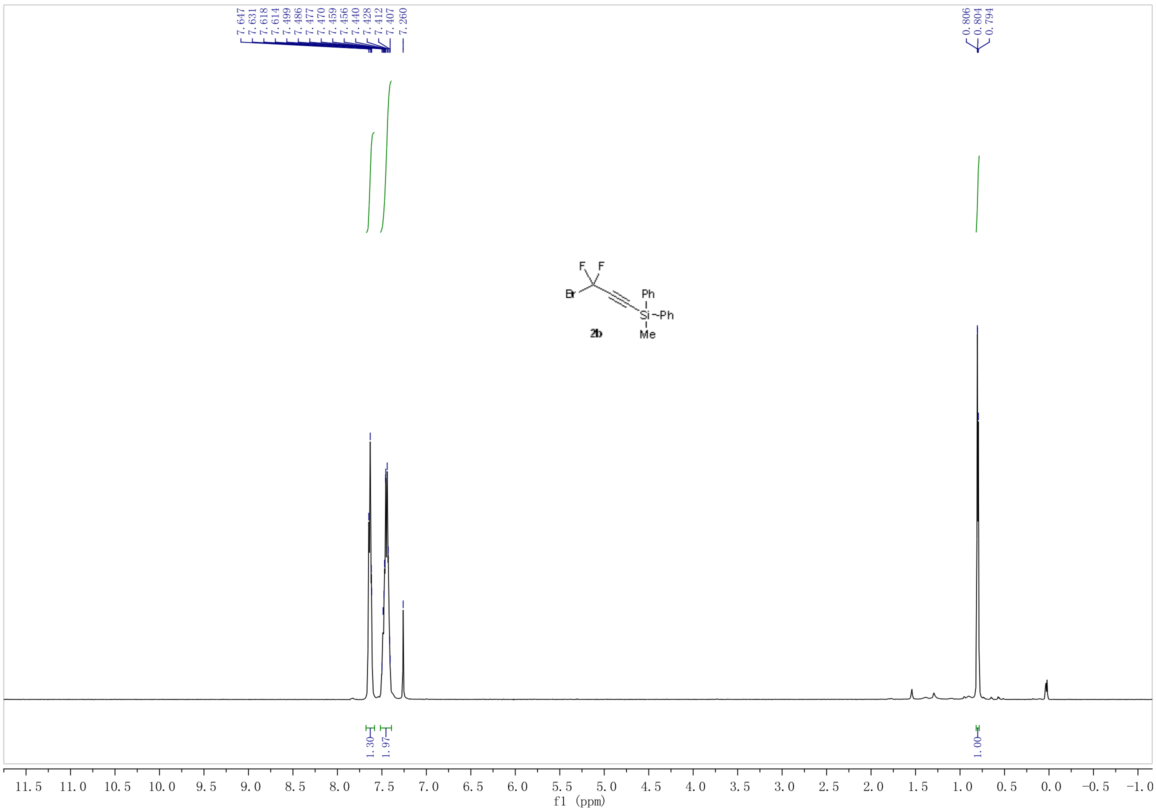

Supplementary Figure 1. ^{1}H NMR of (3-bromo-3,3-difluoroprop-1-yn-1-yl)(methyl)diphenylsilane (2b)

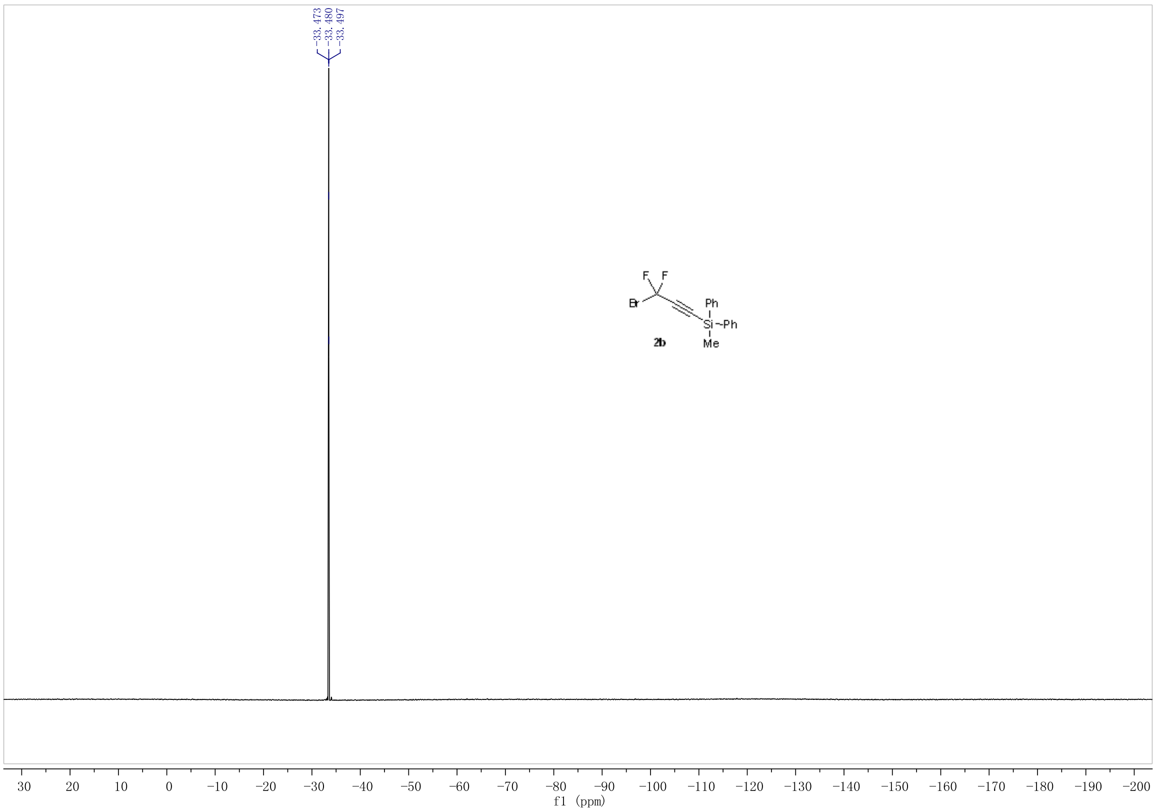

Supplementary Figure 2. ^{19}F NMR of (3-bromo-3,3-difluoroprop-1-yn-1-yl)(methyl)diphenylsilane (2b)

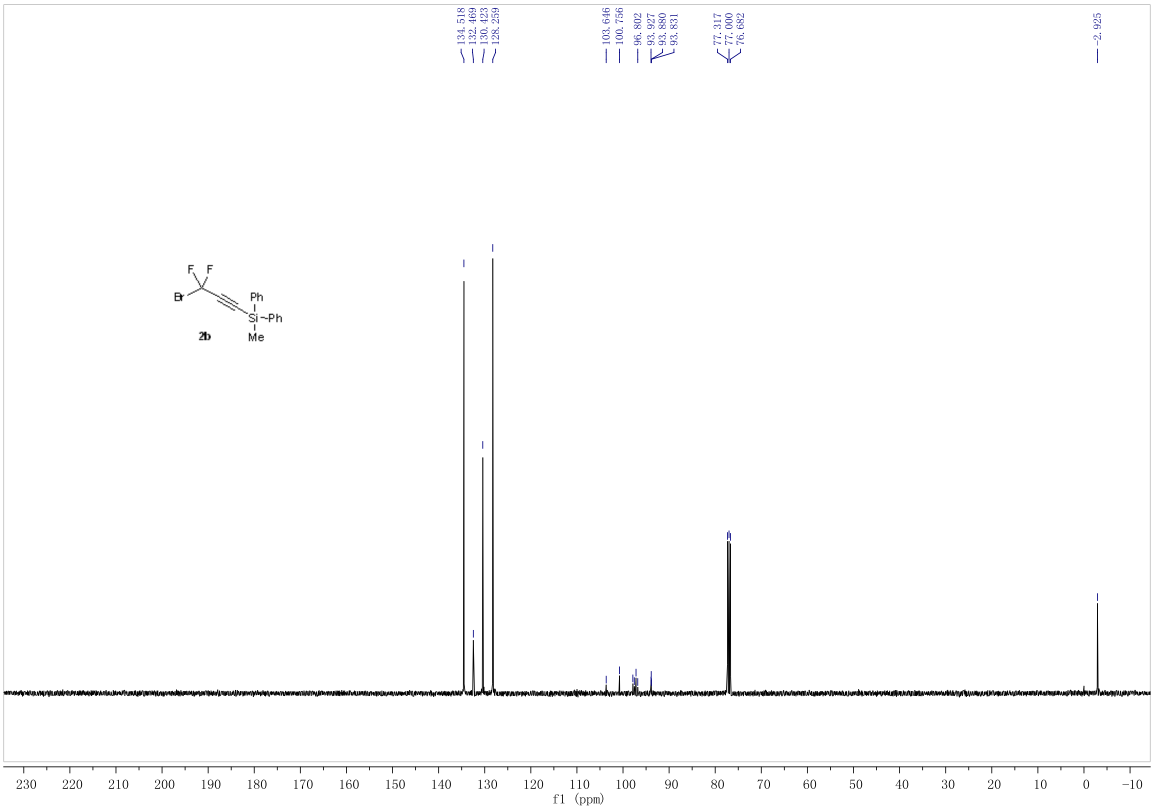

Supplementary Figure 3. ^{13}C NMR of (3-bromo-3,3-difluoroprop-1-yn-1-yl)(methyl)diphenylsilane (2b)

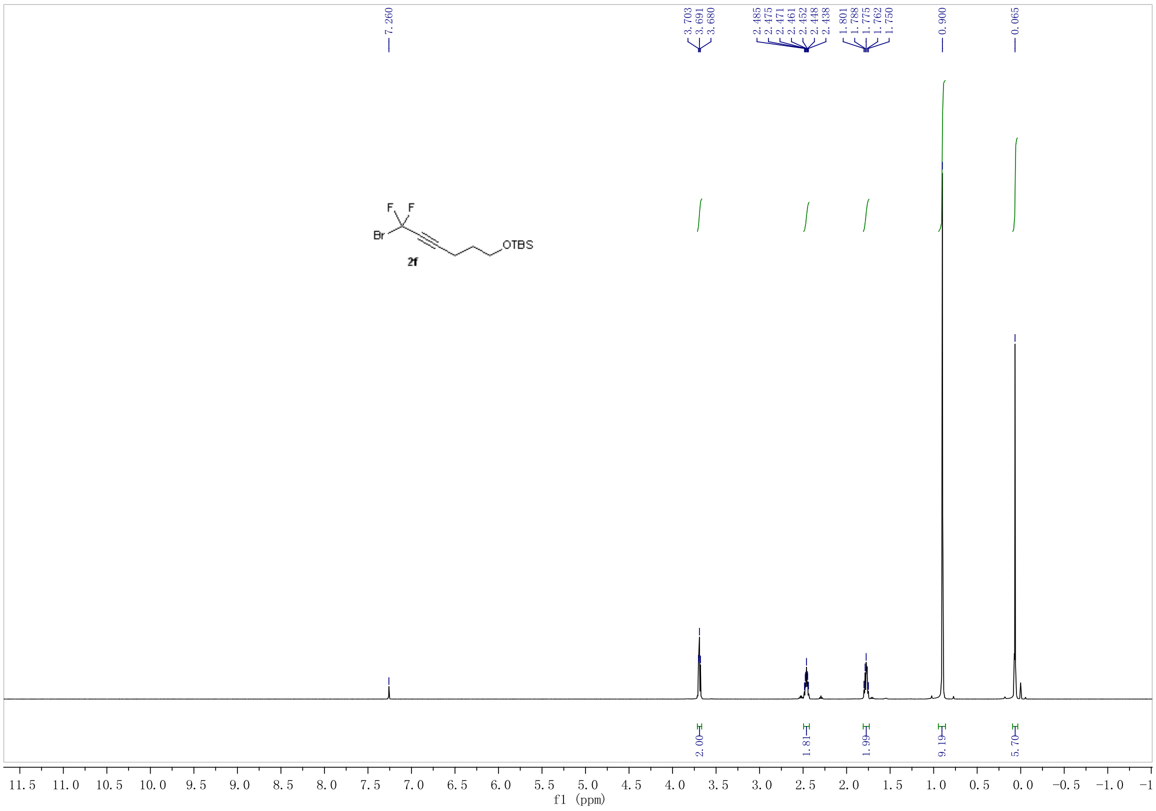

Supplementary Figure 4. ^{1}H NMR of ((6-bromo-6,6-difluorohex-4-yn-1-yl)oxy)(*tert*-butyl)dimethylsilane (2f)

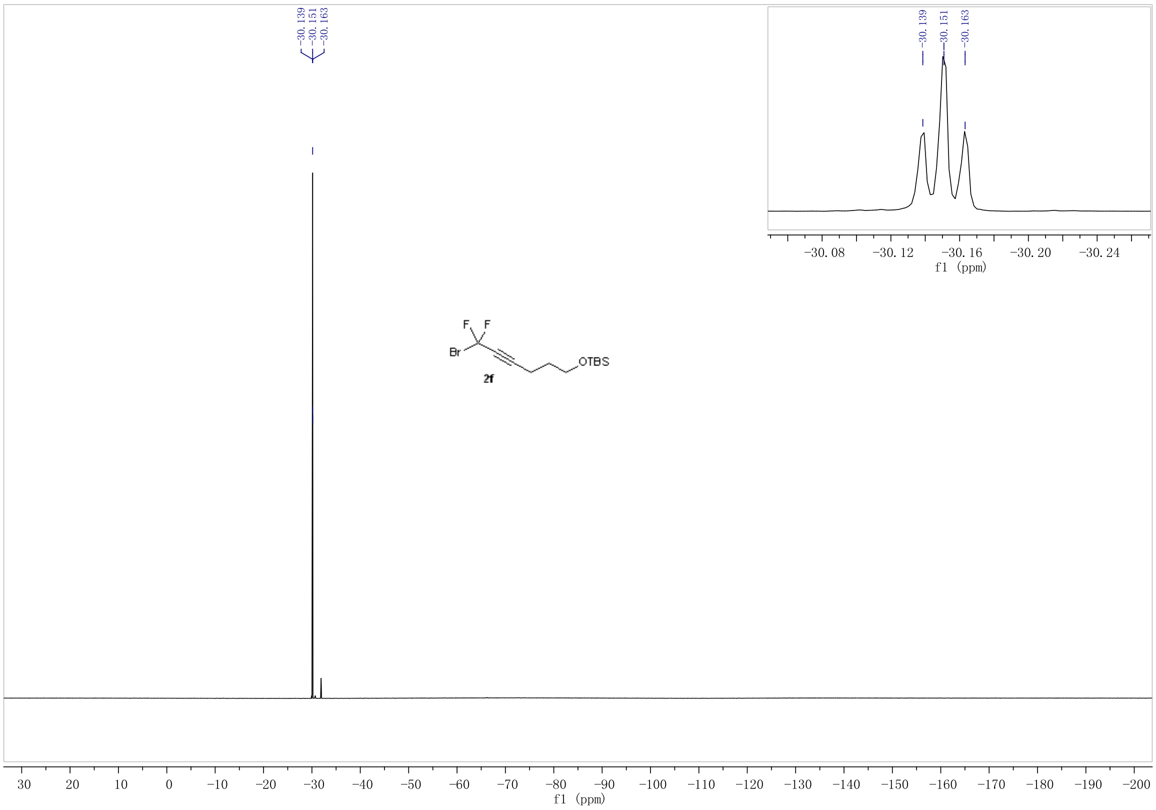

Supplementary Figure 5. ^{19}**F NMR of ((6-bromo-6,6-difluorohex-4-yn-1-yl)oxy)(*tert*-butyl)dimethylsilane (2f)**

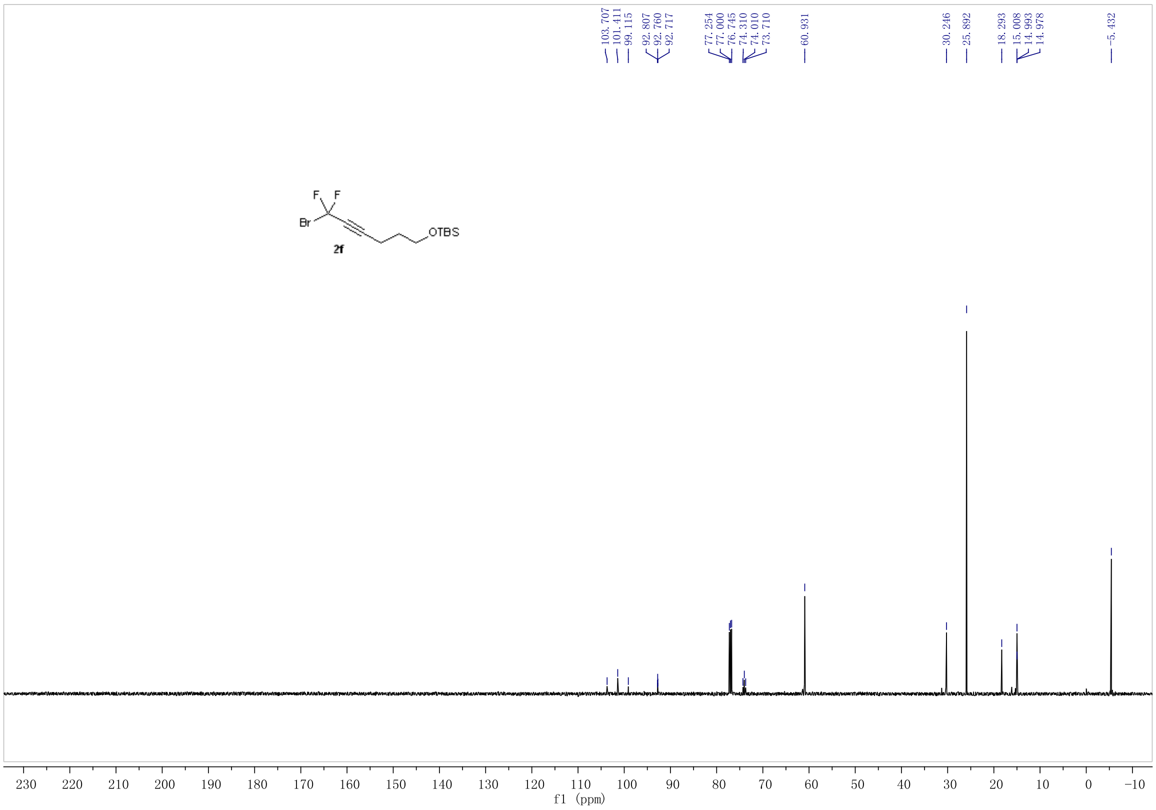

Supplementary Figure 6. ^{13}**C NMR of ((6-bromo-6,6-difluorohex-4-yn-1-yl)oxy)(*tert*-butyl)dimethylsilane (2f)**

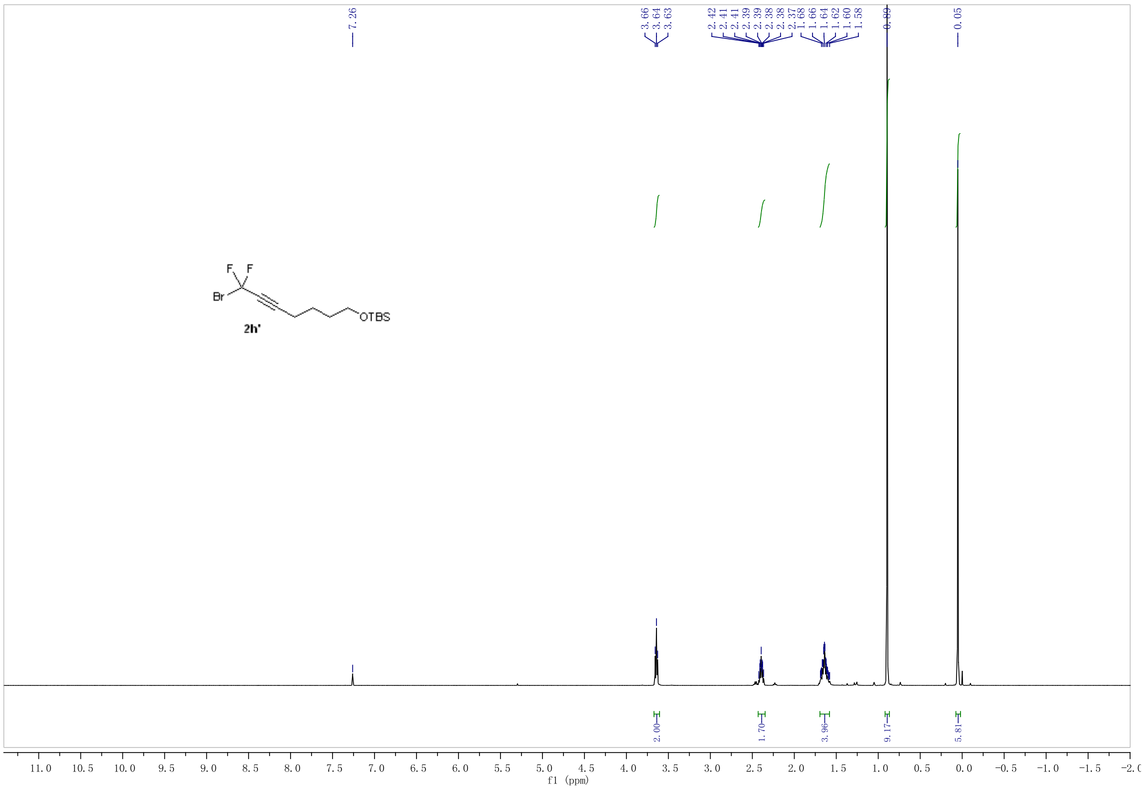

Supplementary Figure 7. ^{1}**H NMR of ((7-bromo-7,7-difluorohept-5-yn-1-yl)oxy)(*tert*-butyl)dimethylsilane (2h′)**

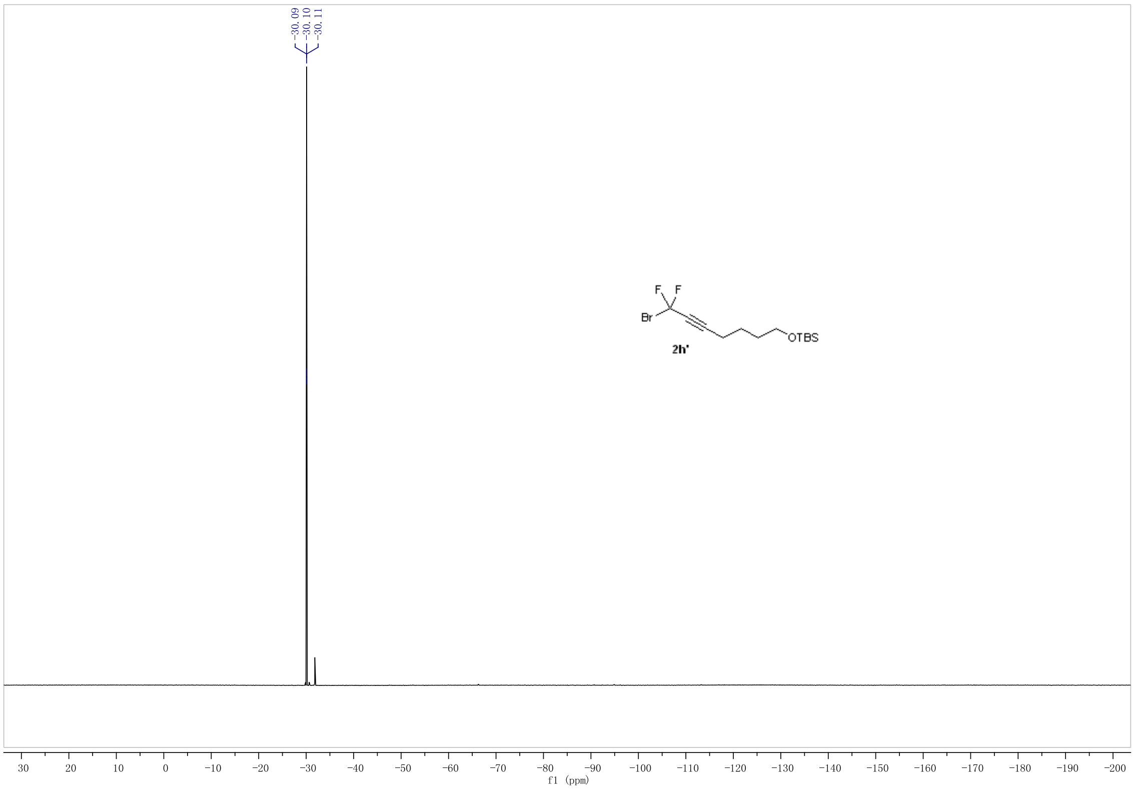

Supplementary Figure 8. ^{19}**F NMR of ((7-bromo-7,7-difluorohept-5-yn-1-yl)oxy)(*tert*-butyl)dimethylsilane (2h′)**

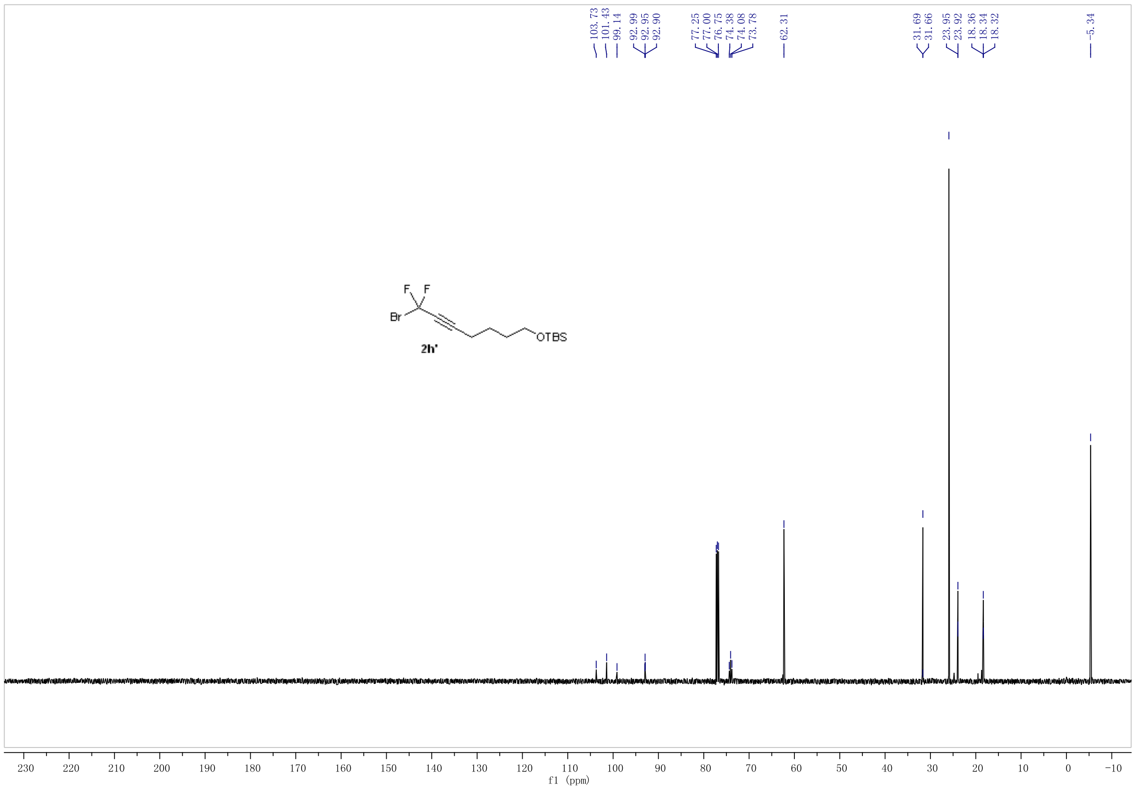

Supplementary Figure 9. ^{13}C NMR of ((7-bromo-7,7-difluorohept-5-yn-1-yl)oxy)(*tert*-butyl)dimethylsilane (2h′)

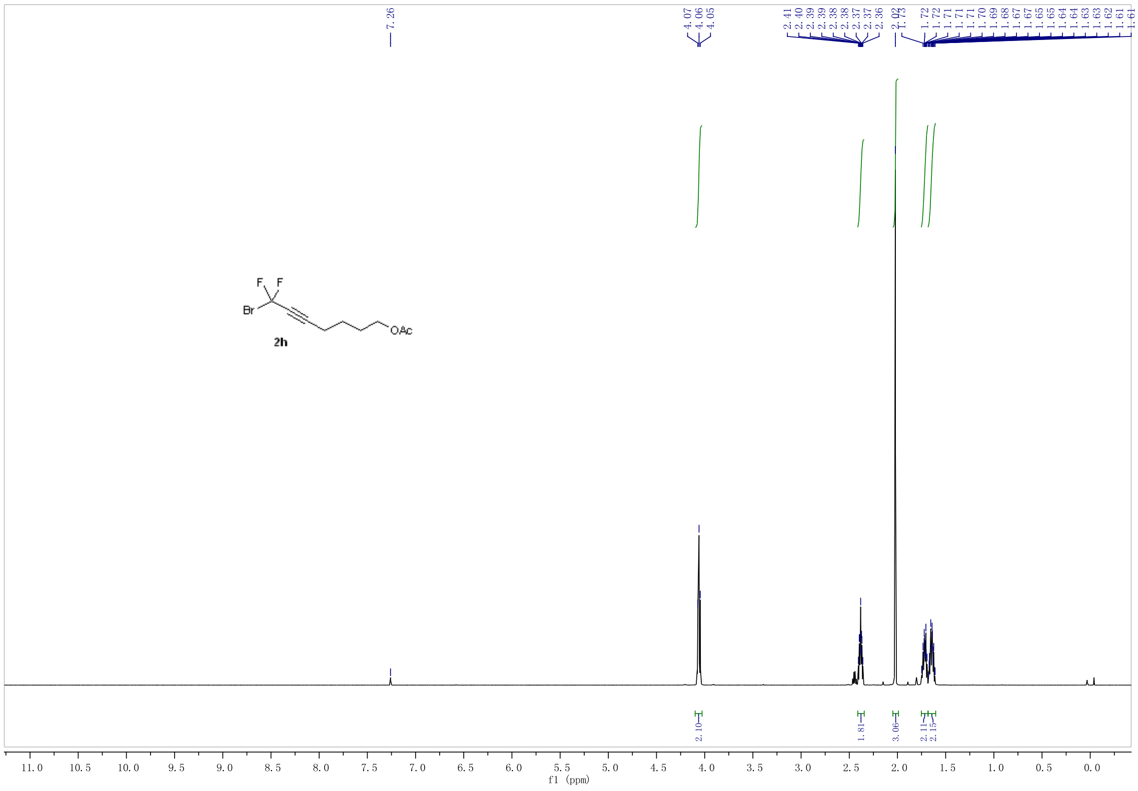

Supplementary Figure 10. ^{1}H NMR of 7-bromo-7,7-difluorohept-5-yn-1-yl acetate (2h)

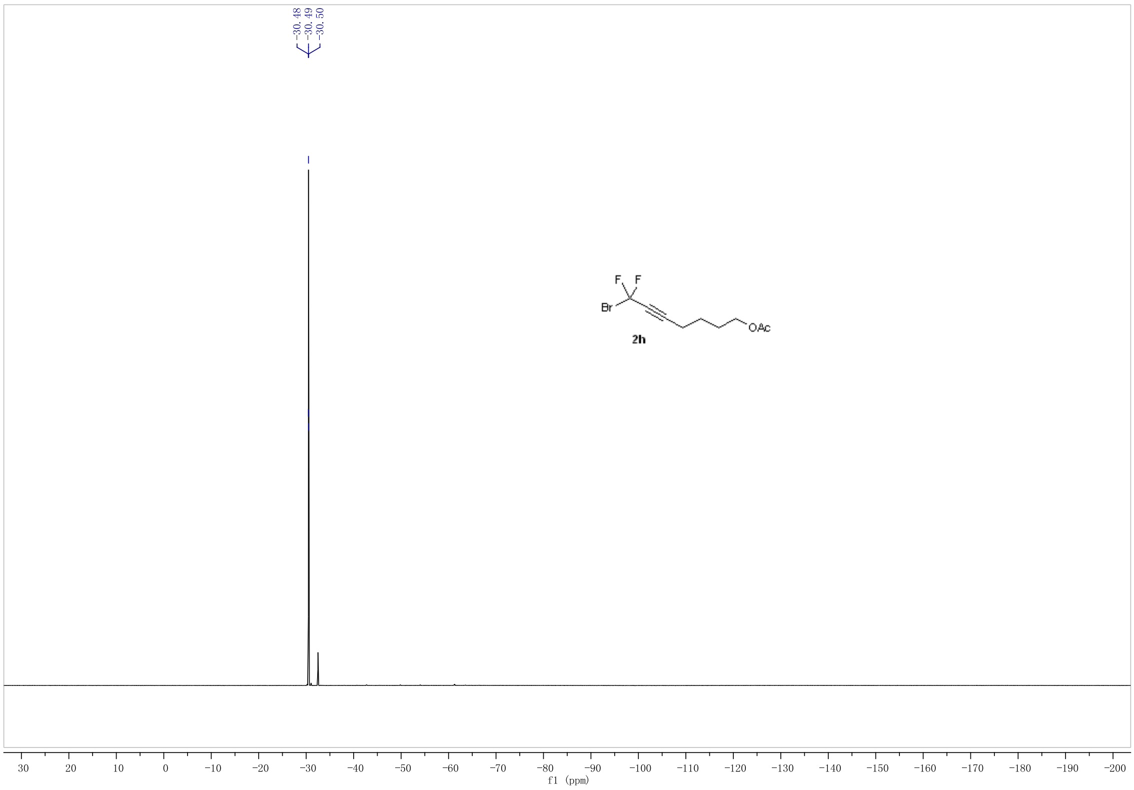

Supplementary Figure 11. ^{19}F NMR of 7-bromo-7,7-difluorohept-5-yn-1-yl acetate (2h)

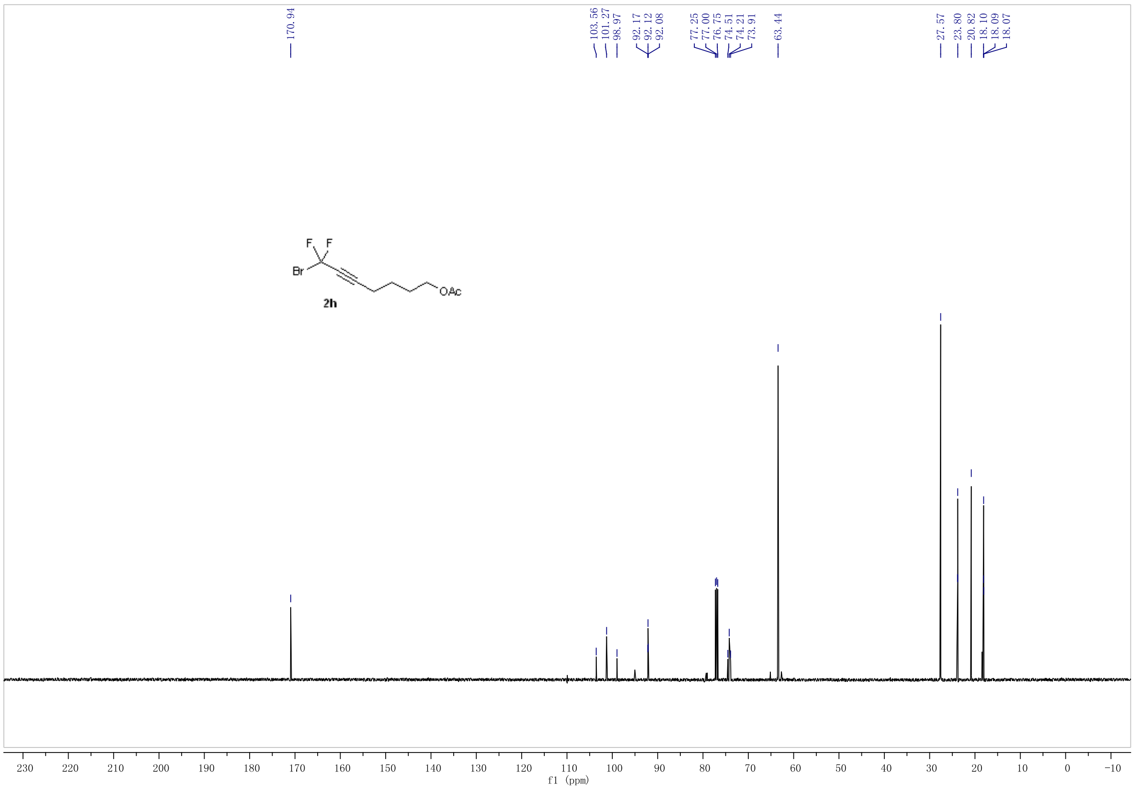

Supplementary Figure 12. ^{13}C NMR of 7-bromo-7,7-difluorohept-5-yn-1-yl acetate (2h)

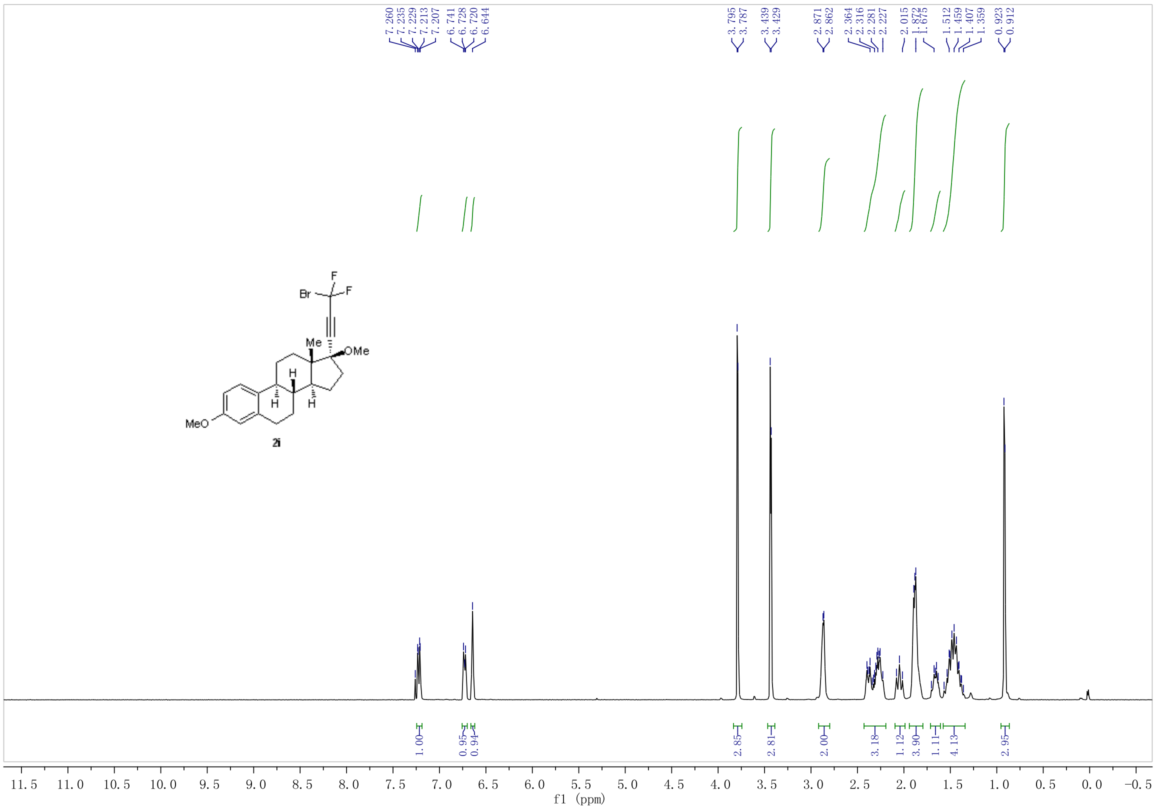

Supplementary Figure 13. ^{1}H NMR of product 2i

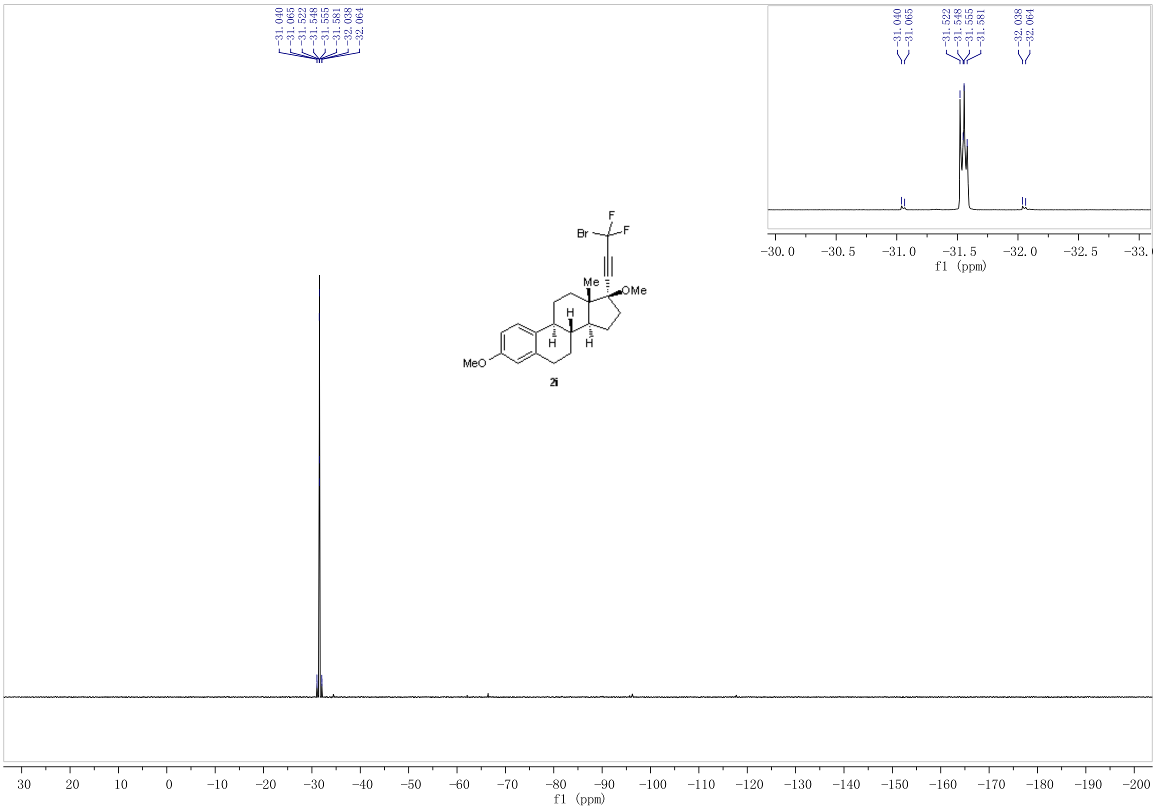

Supplementary Figure 14. ^{19}F NMR of product 2i

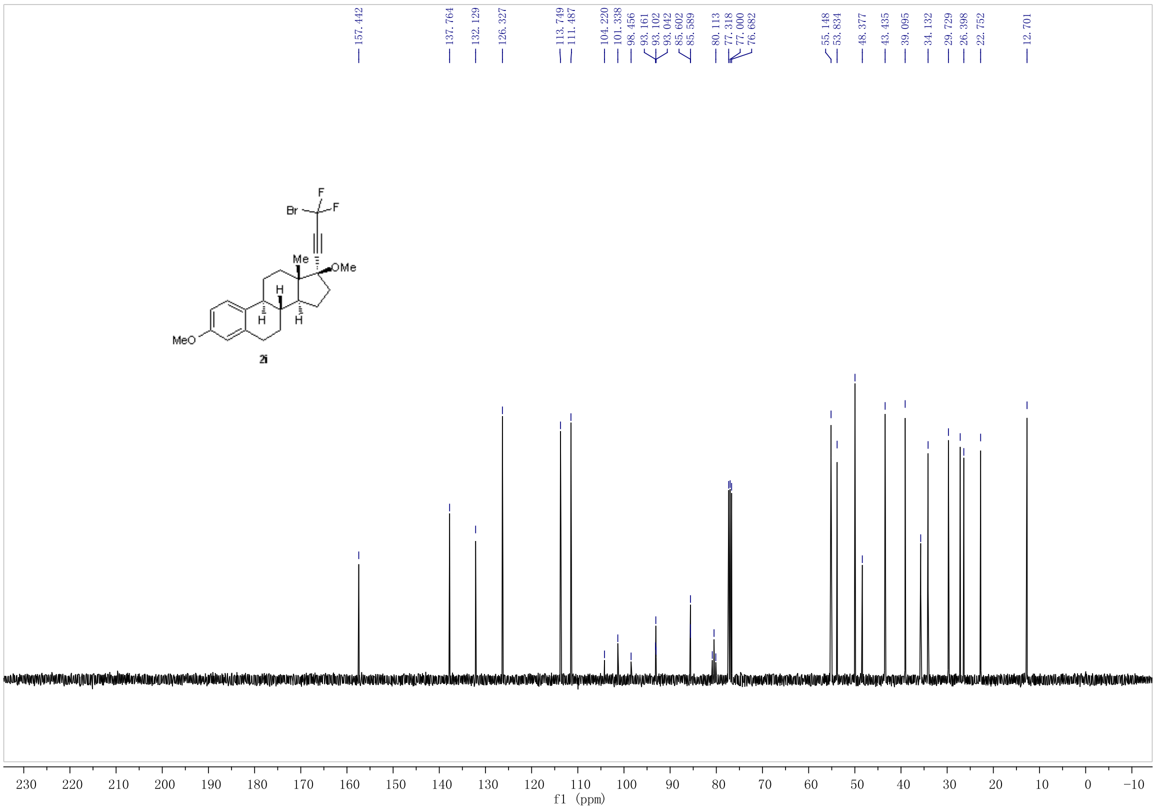

Supplementary Figure 15. ^{13}C NMR of product 2i

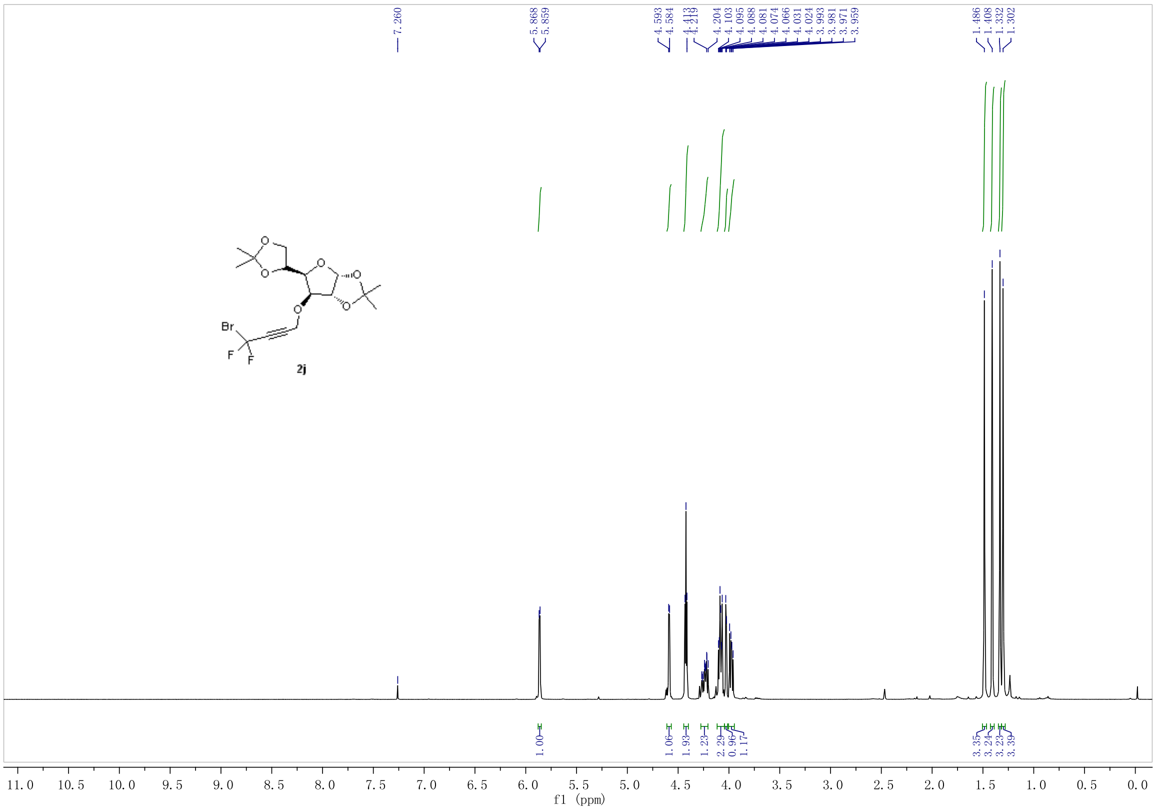

Supplementary Figure 16. ^{1}H NMR of product 2j

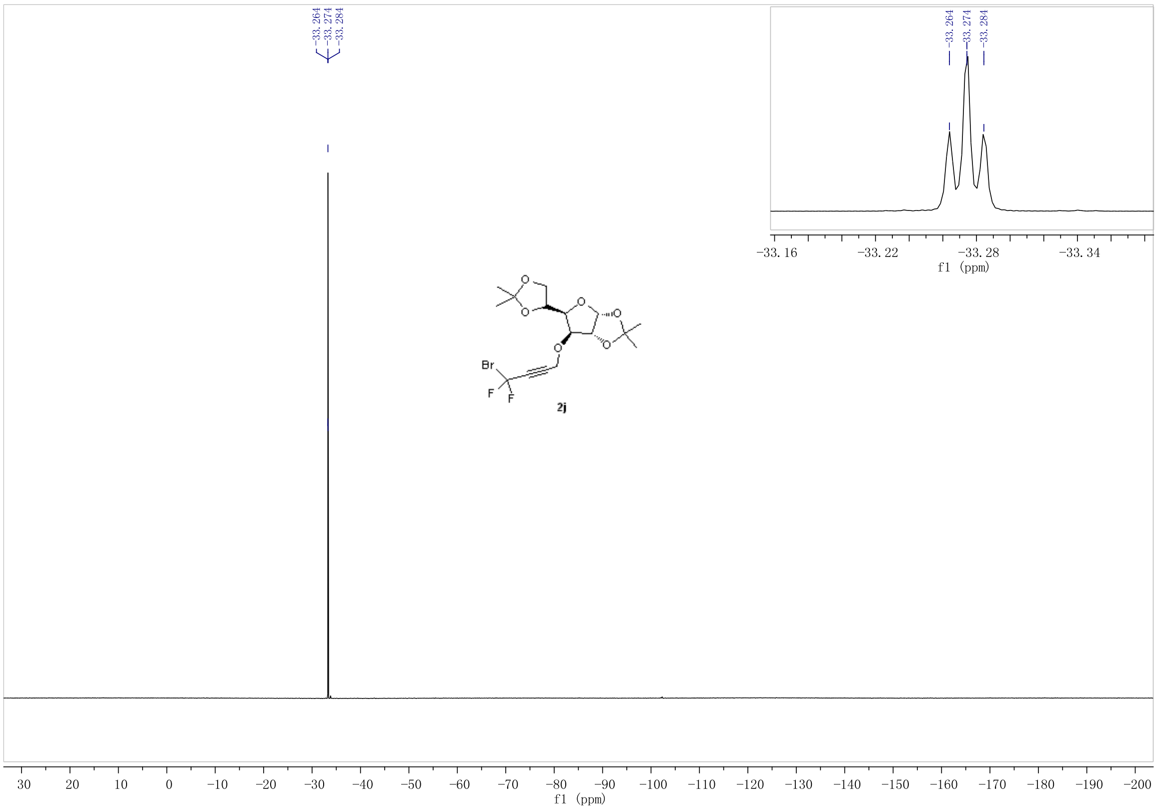

Supplementary Figure 17. ^{19}F NMR of product 2j

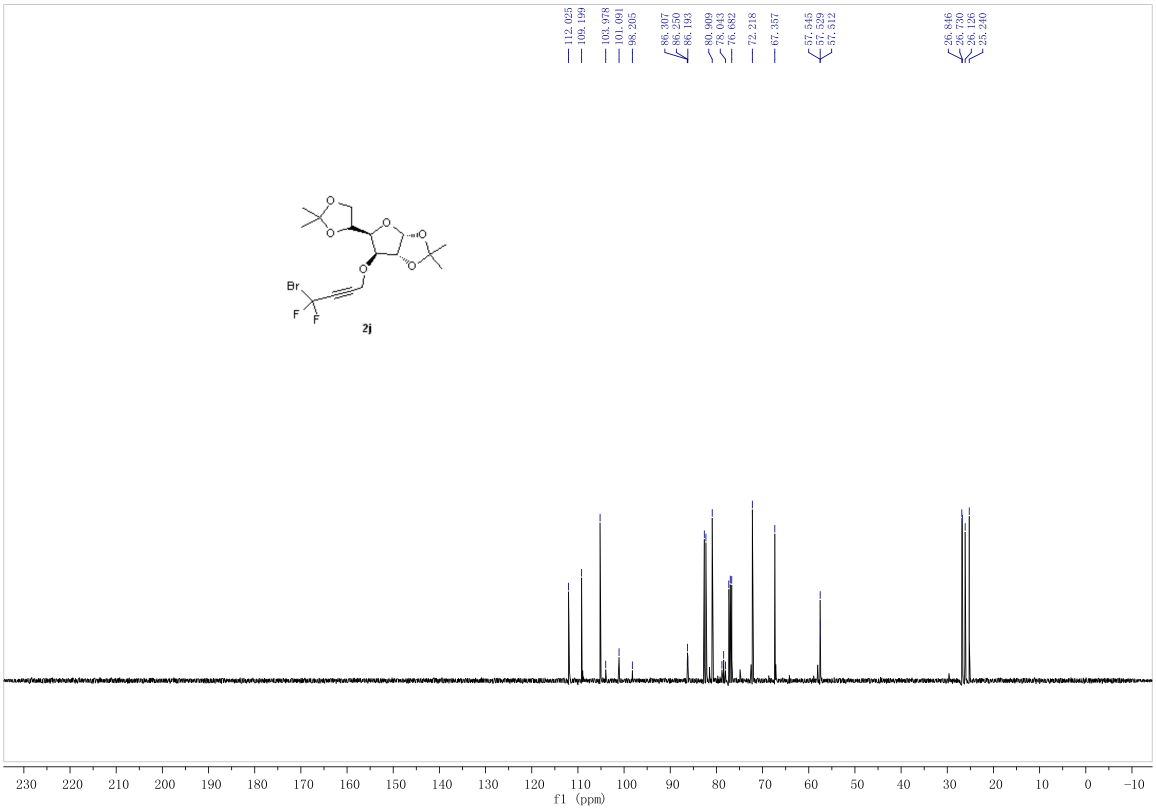

Supplementary Figure 18. ^{13}C NMR of product 2j

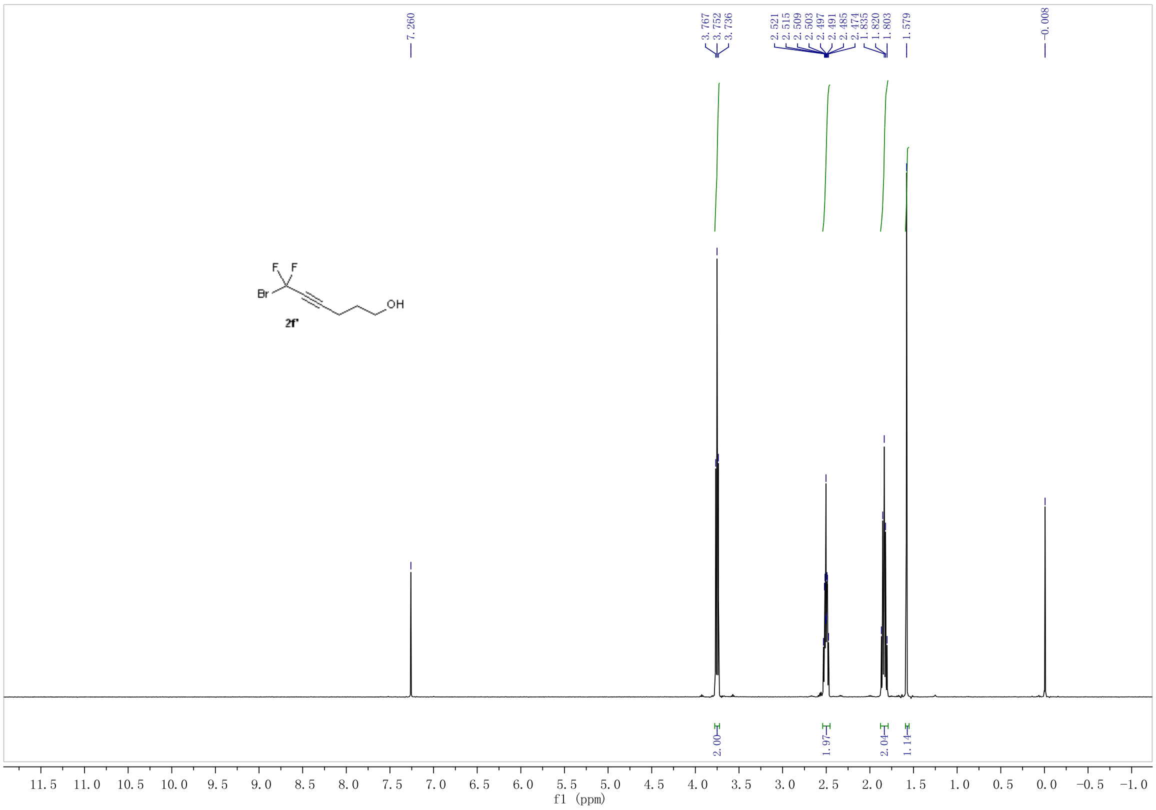

Supplementary Figure 19. ^{1}H NMR of 6-bromo-6,6-difluorohex-4-yn-1-ol (2f′)

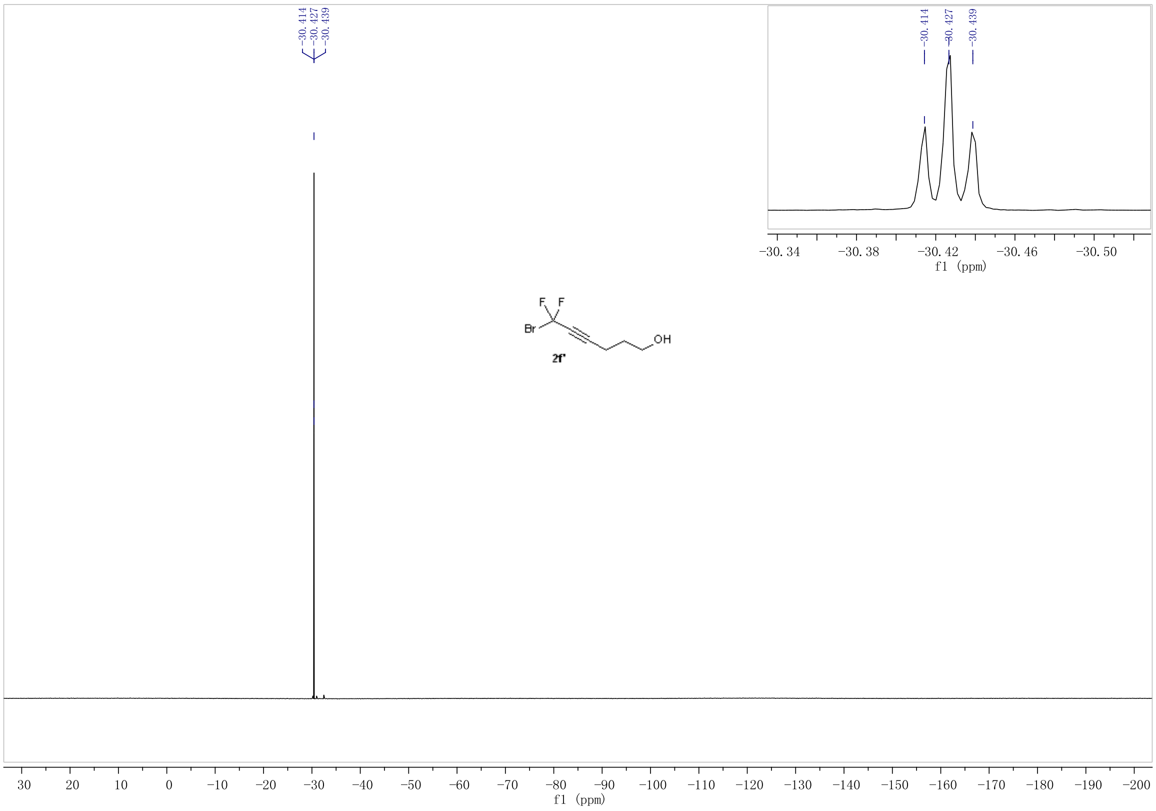

Supplementary Figure 20. ^{19}F NMR of 6-bromo-6,6-difluorohex-4-yn-1-ol (2f′)

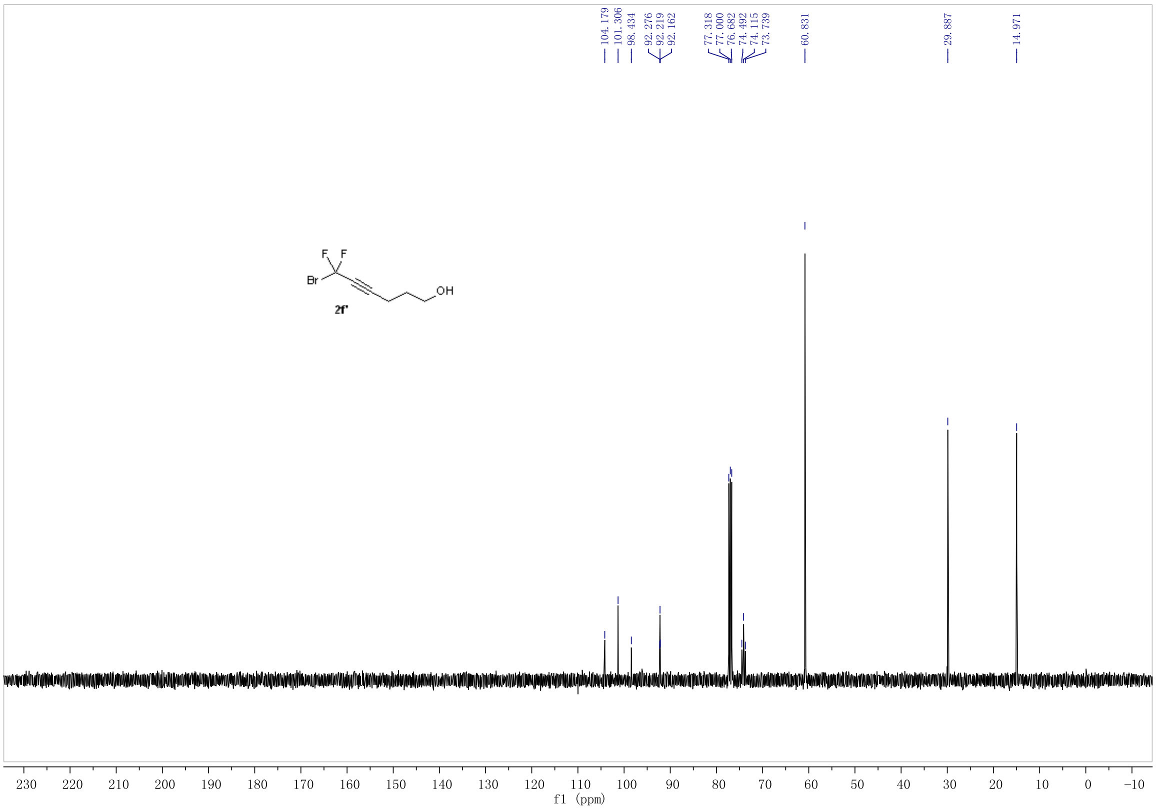

Supplementary Figure 21. ^{13}C NMR of 6-bromo-6,6-difluorohex-4-yn-1-ol (2f′)

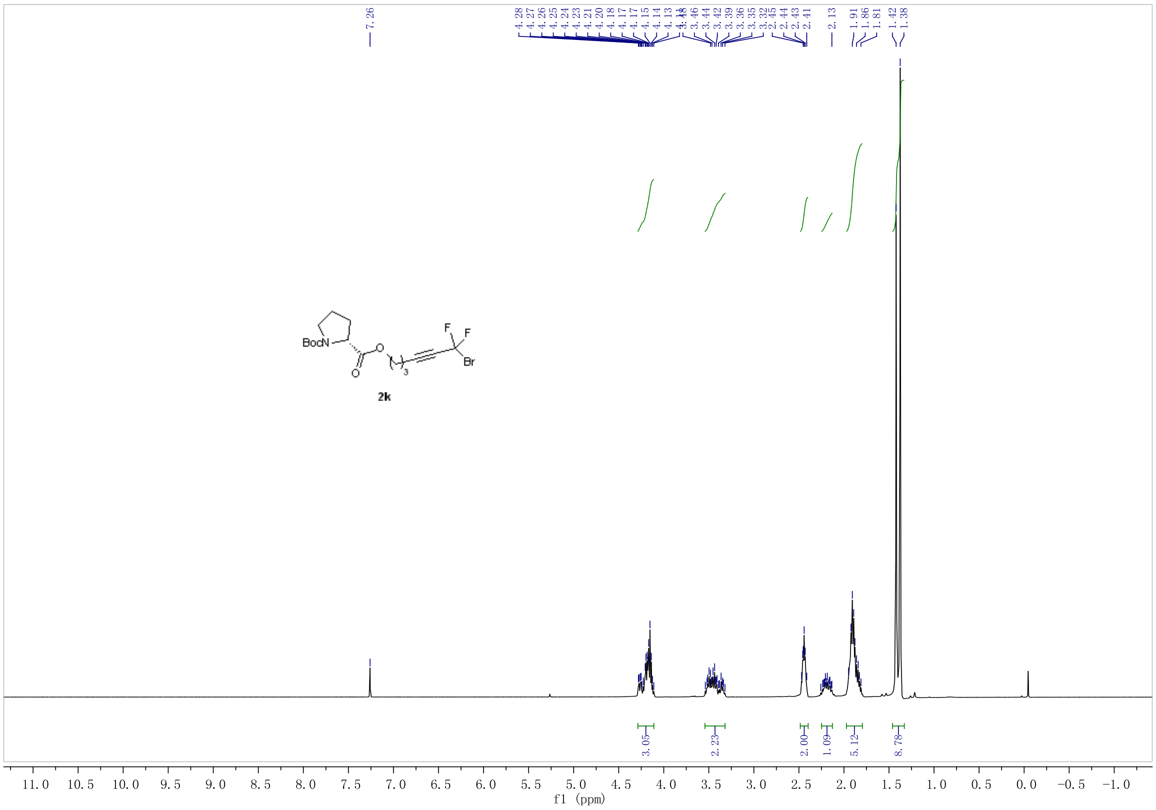

Supplementary Figure 22. ^{1}H NMR of (*R*)-2-(6-bromo-6,6-difluorohex-4-yn-1-yl) 1-*tert*-butyl pyrrolidine-1,2-dicarboxylate (2k)

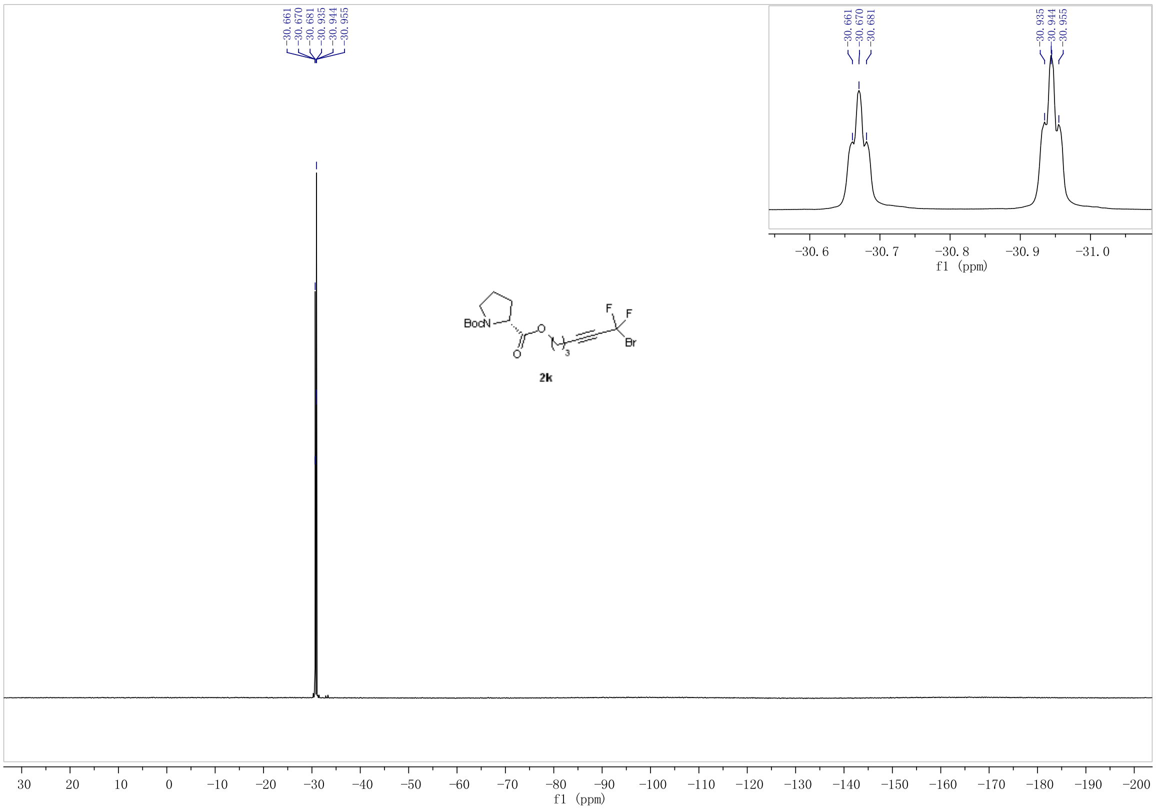

Supplementary Figure 23. ^{19}F NMR of (*R*)-2-(6-bromo-6,6-difluorohex-4-yn-1-yl) 1-*tert*-butyl pyrrolidine-1,2-dicarboxylate

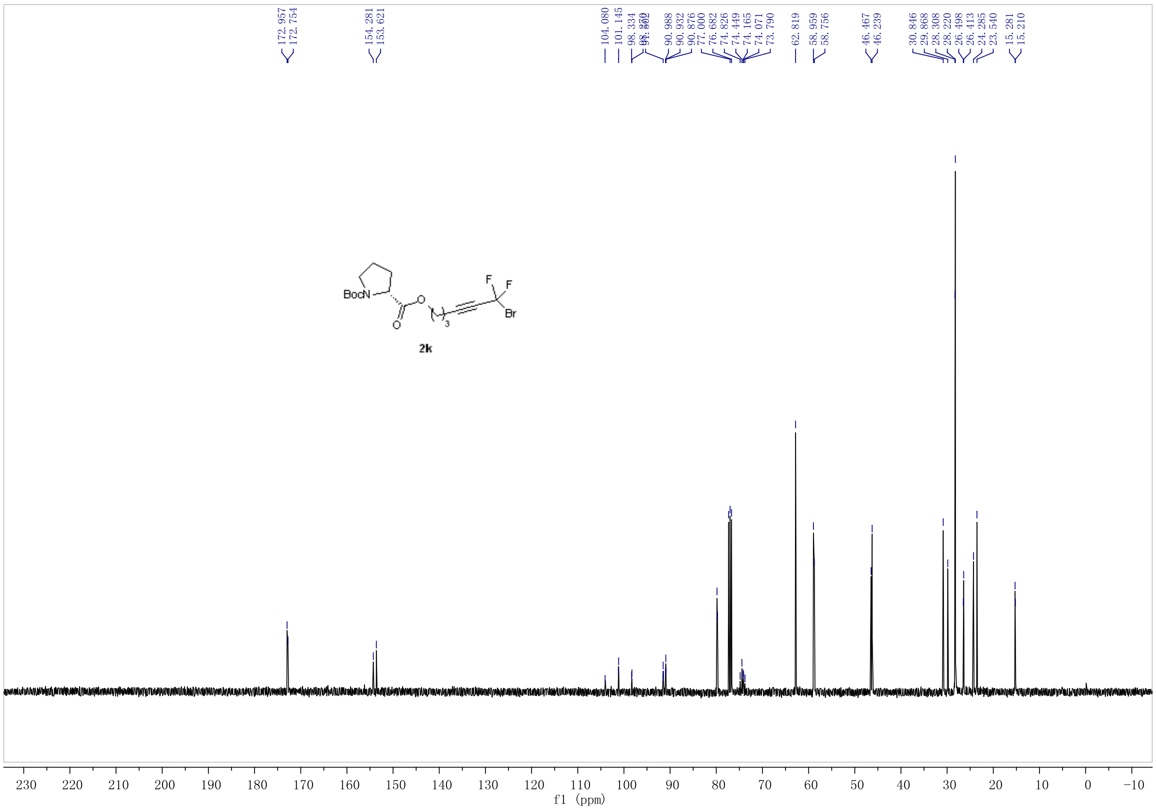

Supplementary Figure 24. ^{13}C NMR of (*R*)-2-(6-bromo-6,6-difluorohex-4-yn-1-yl) 1-*tert*-butyl pyrrolidine-1,2-dicarboxylate

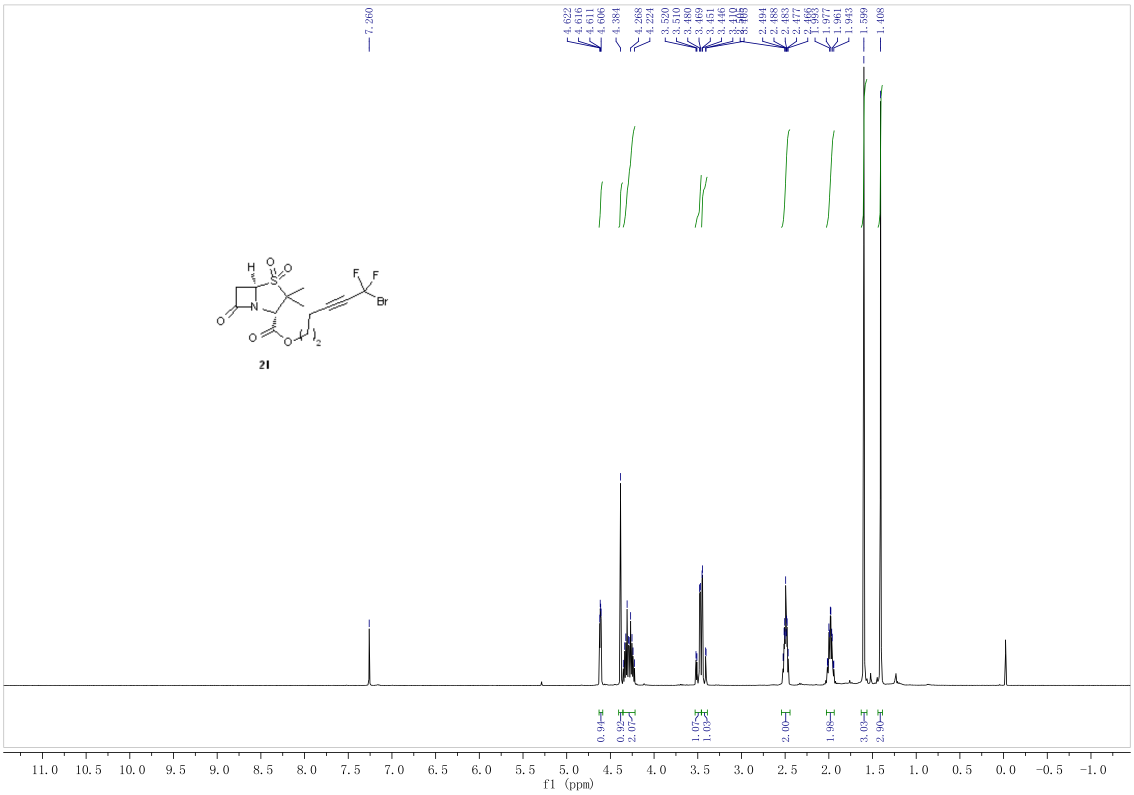

Supplementary Figure 25. ^{1}H NMR of product 2l

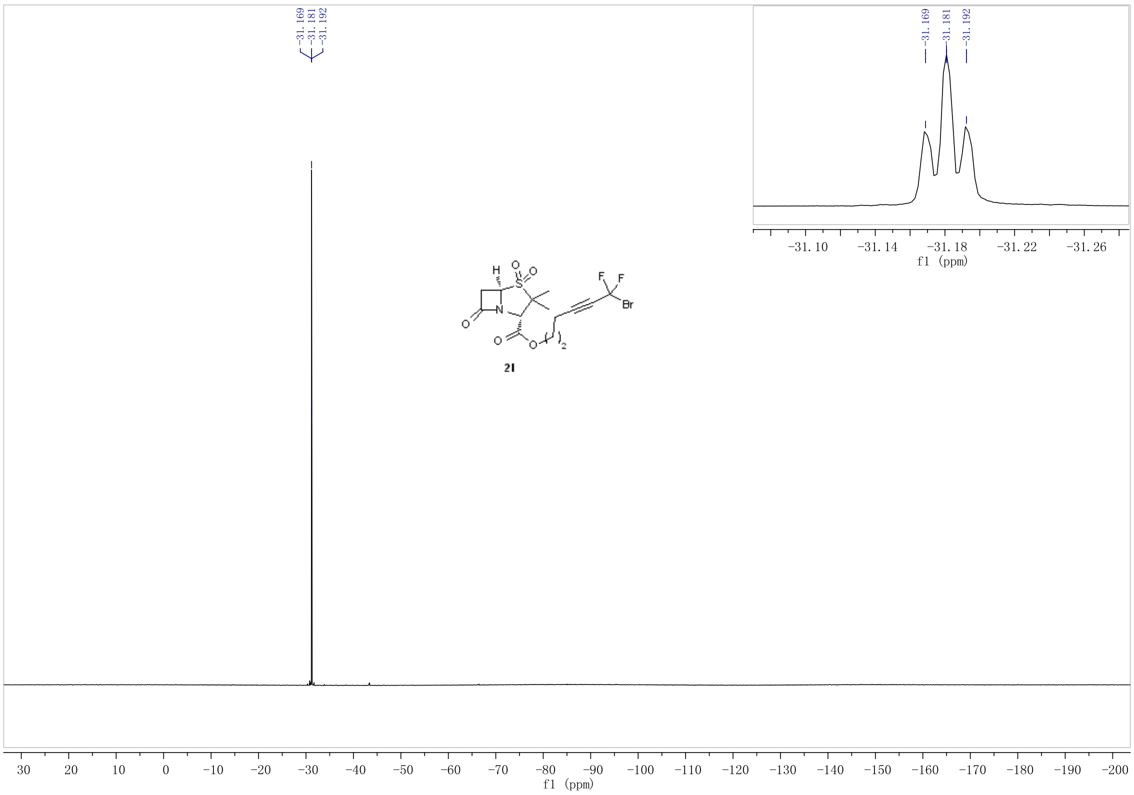

Supplementary Figure 26. ^{19}F NMR of product 2l

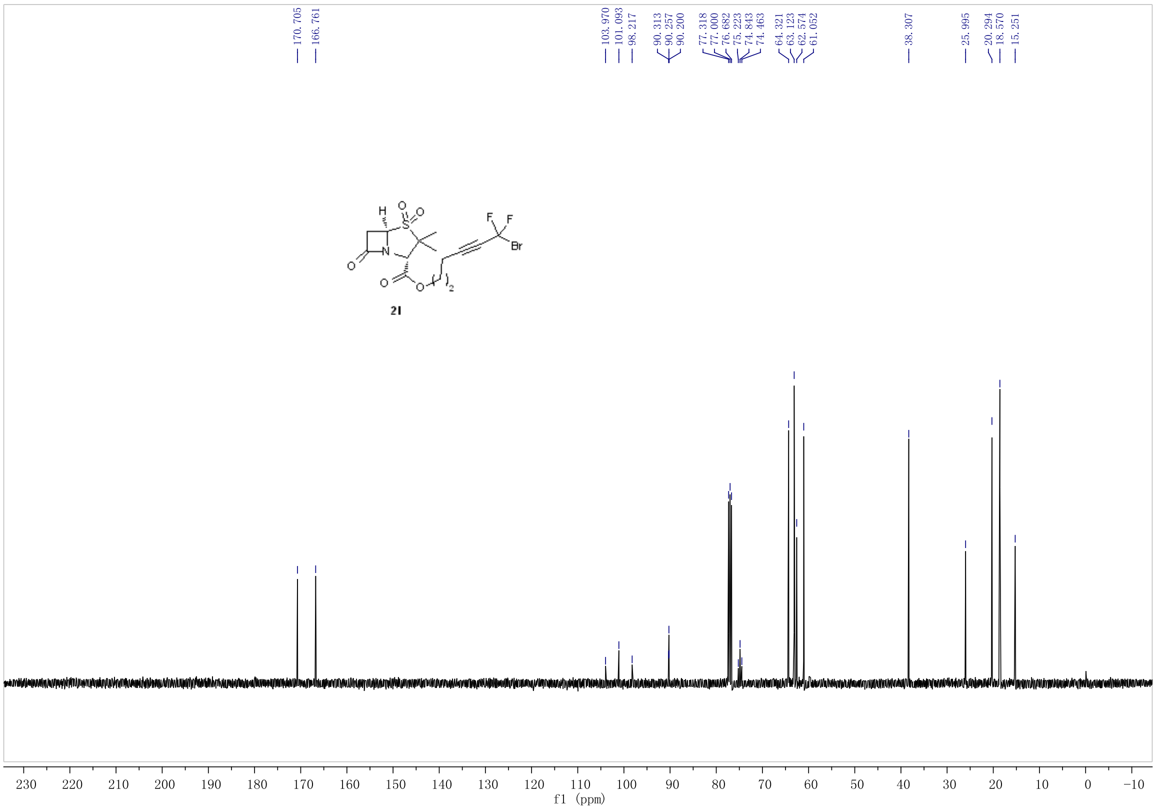

Supplementary Figure 27. ^{13}**C NMR of product 2l**

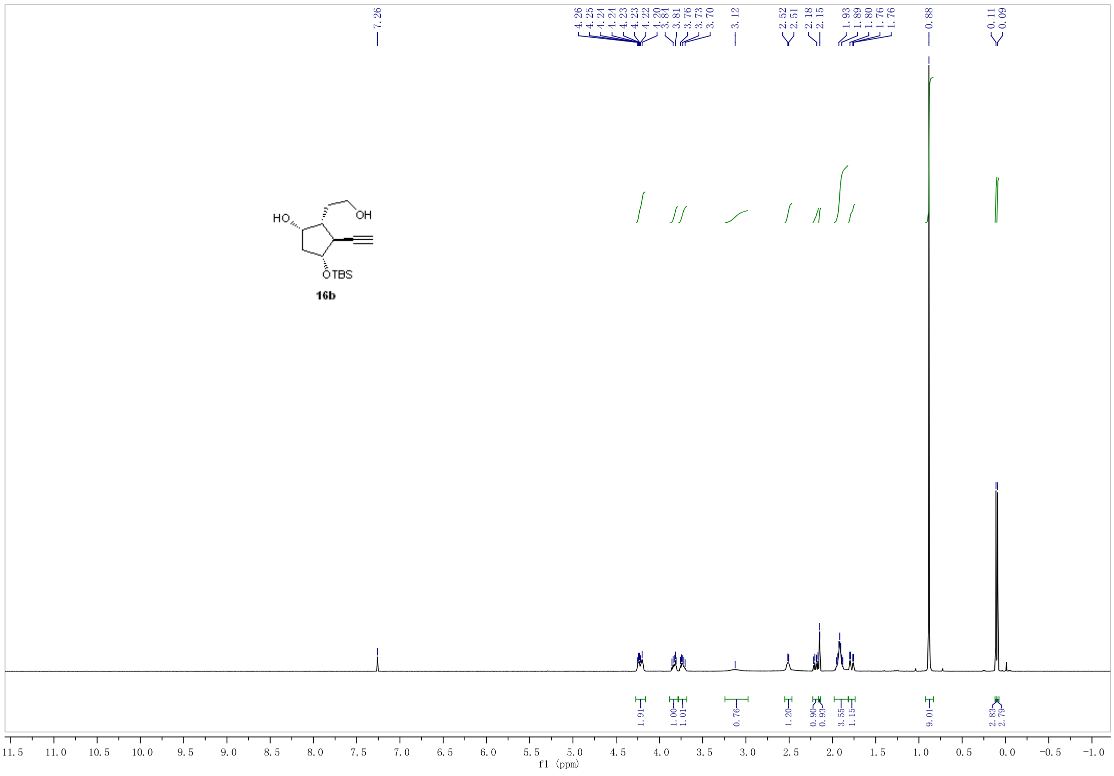

Supplementary Figure 28. ^{1}**H NMR of**

(1*S*,2*R*,3*S*,4*R*)-4-((*tert*-butyldimethylsilyl)oxy)-3-ethynyl-2-(2-hydroxyethyl)cyclopentanol (16b)

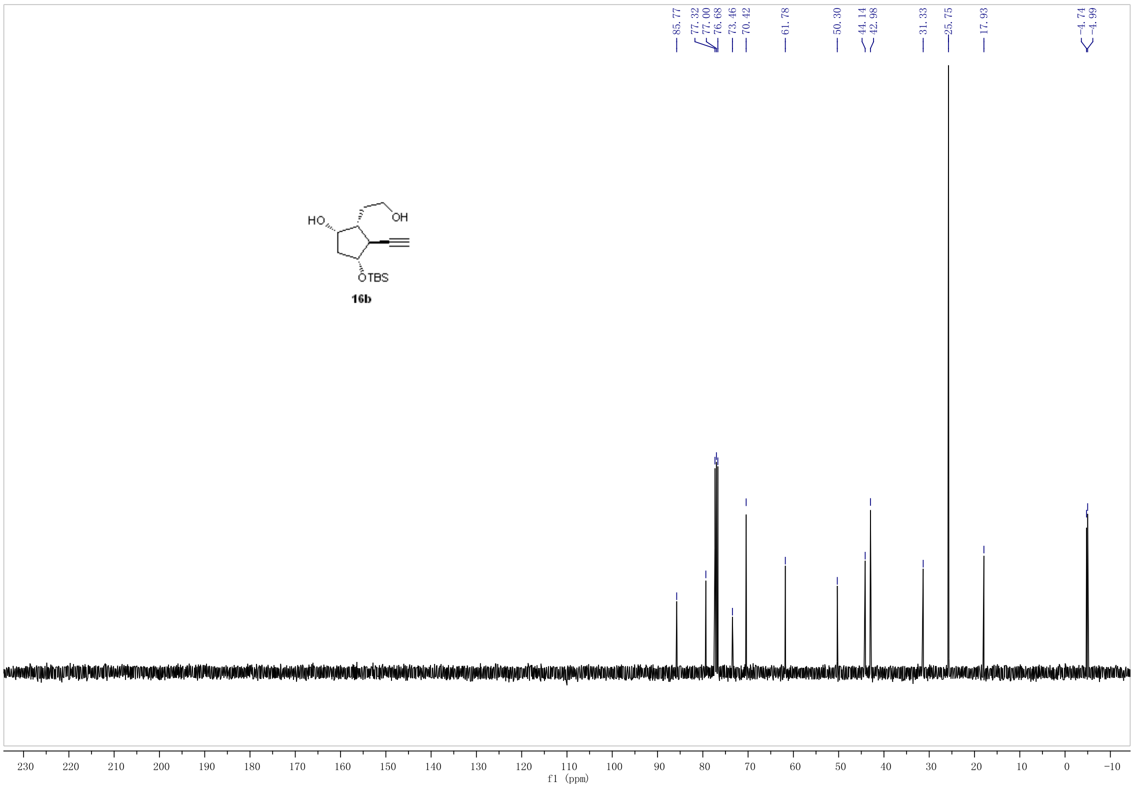

Supplementary Figure 29. ^{13}C NMR of

(1*S*,2*R*,3*S*,4*R*)-4-((*tert*-butyldimethylsilyl)oxy)-3-ethynyl-2-(2-hydroxyethyl)cyclopentanol (16b)

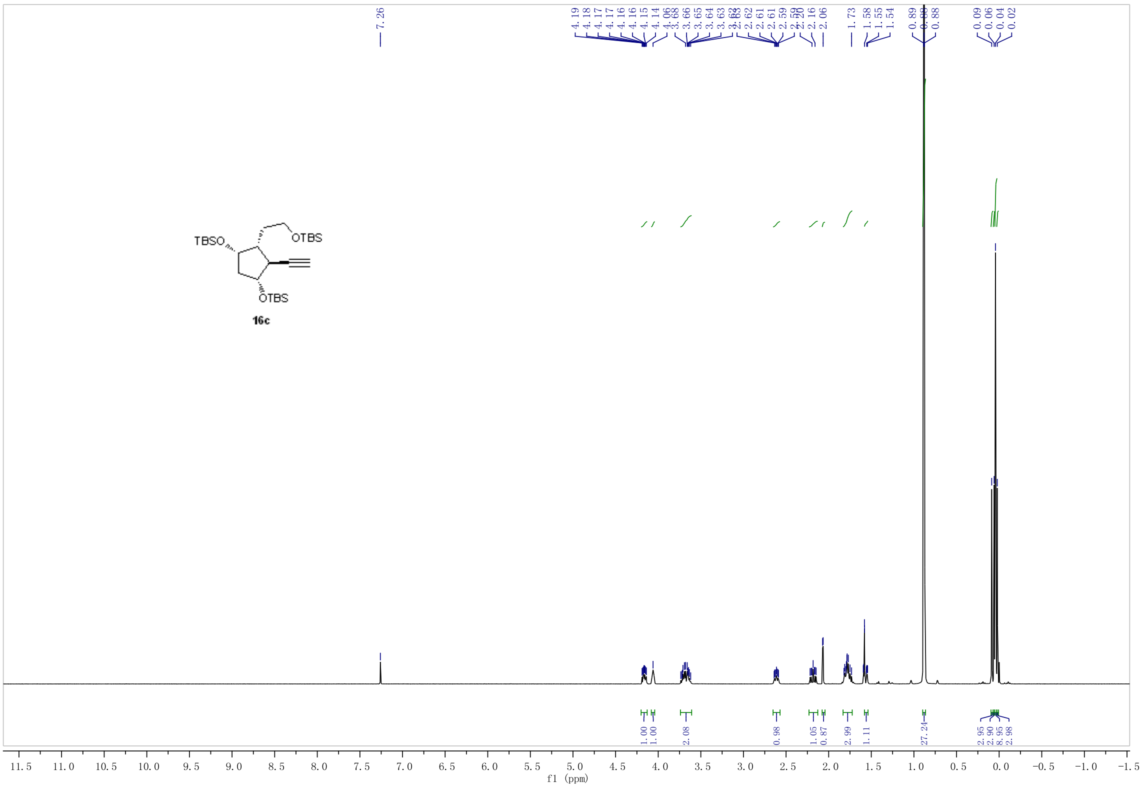

Supplementary Figure 30. ^{1}H NMR of product 16c

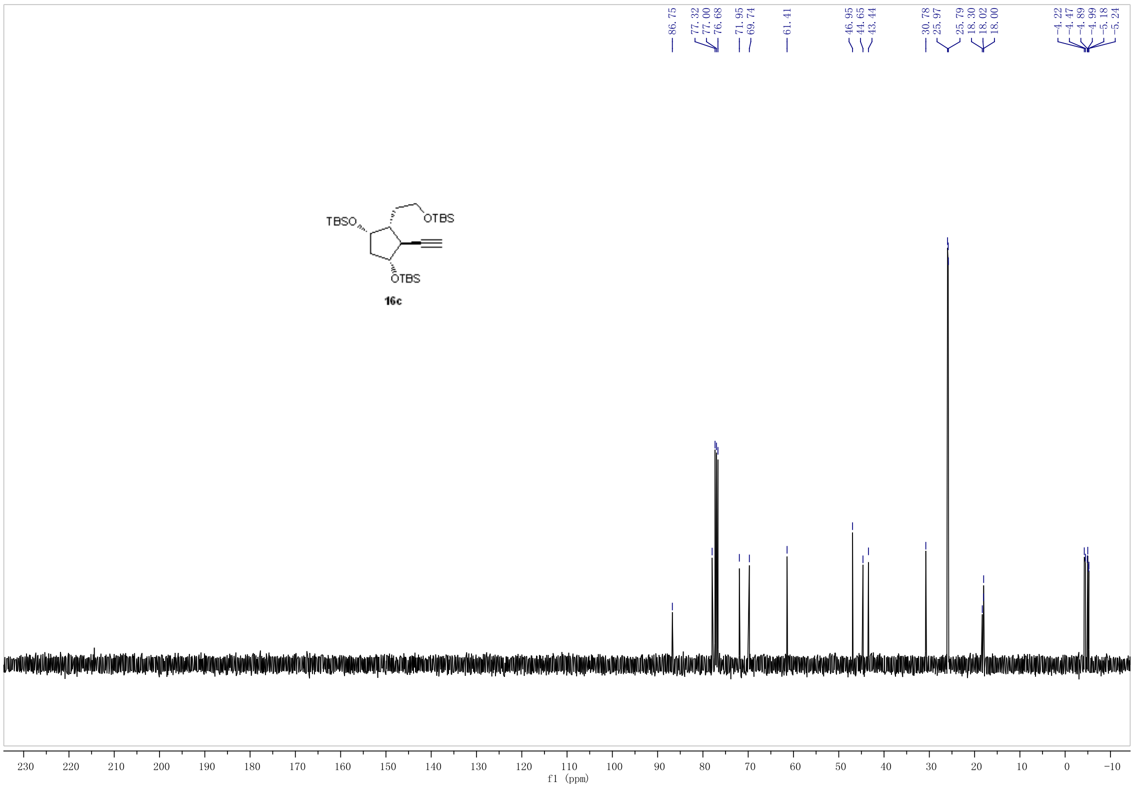

Supplementary Figure 31. ^{13}C NMR of product 16c

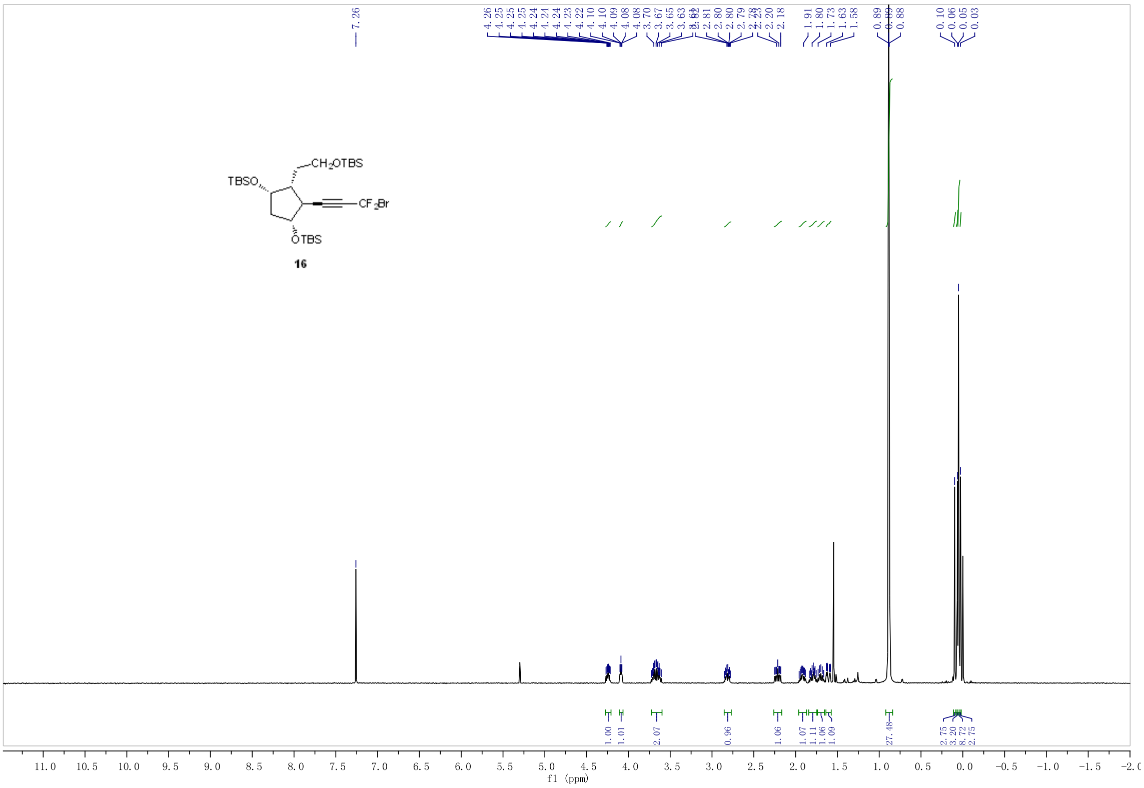

Supplementary Figure 32. ^{1}H NMR of product 16

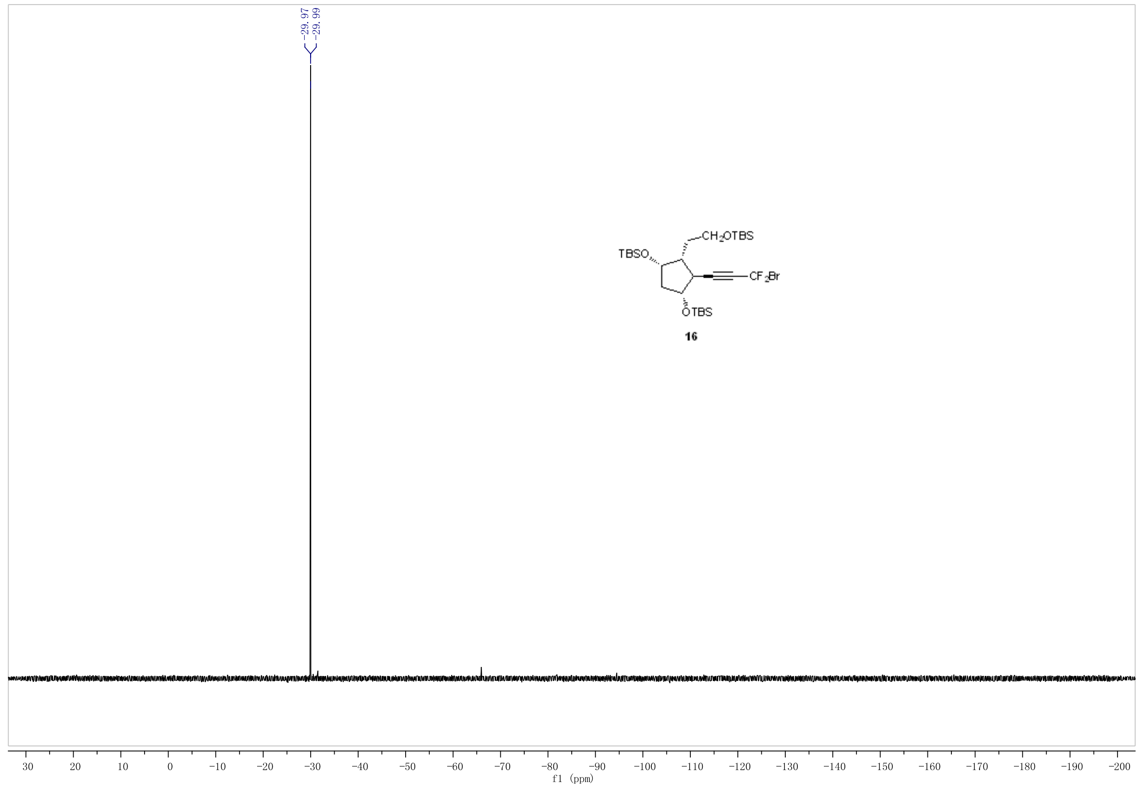

Supplementary Figure 33. ^{19}F NMR of product 16

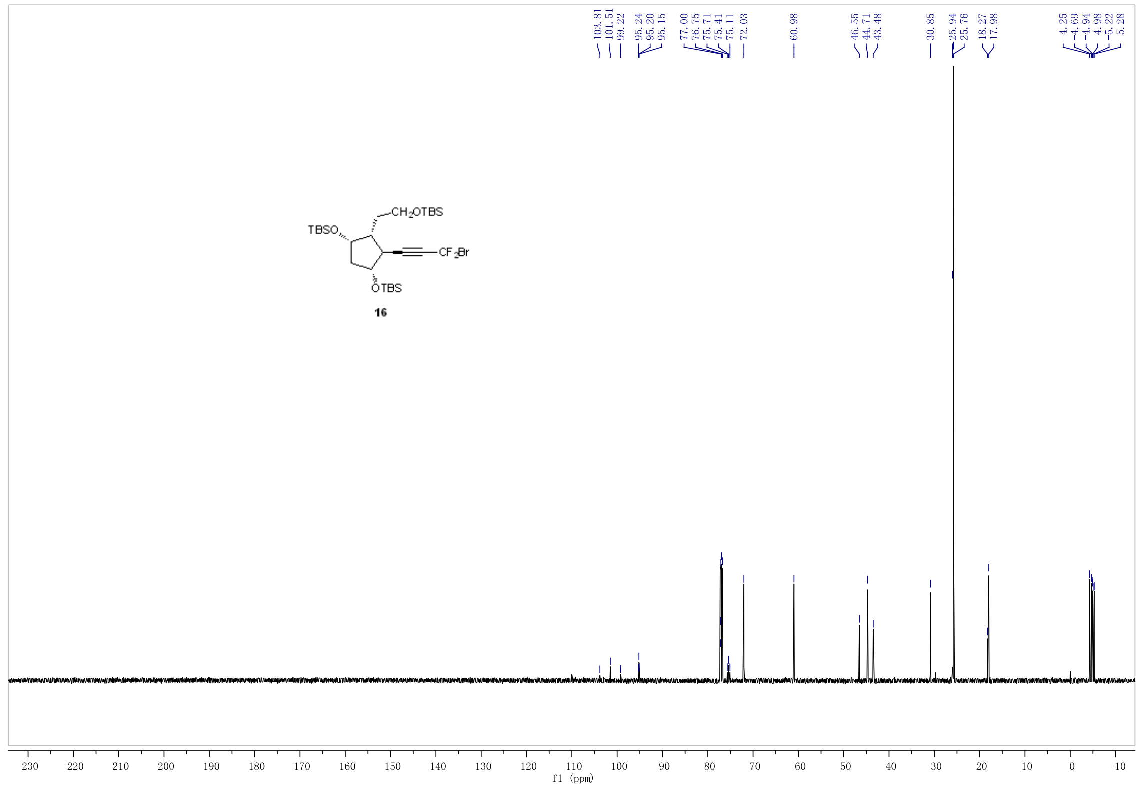

Supplementary Figure 34. ^{13}C NMR of product 16

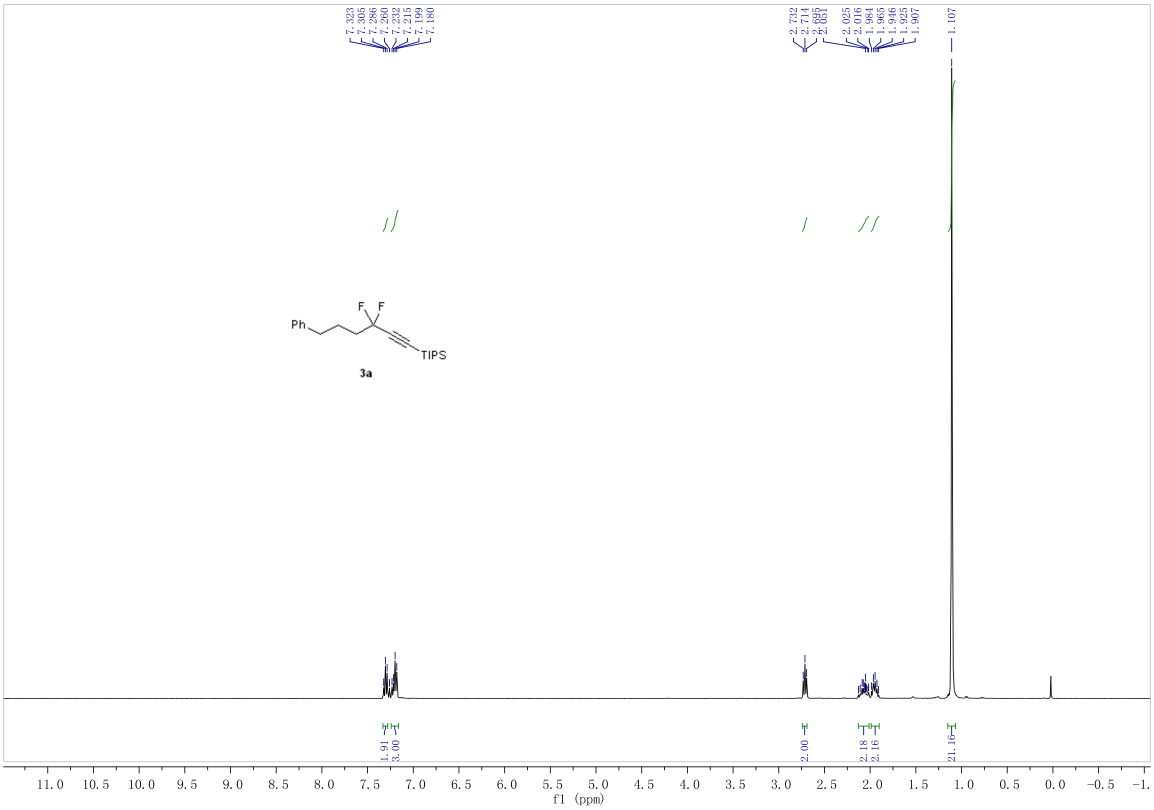

Supplementary Figure 35. ^{1}H NMR of (3, 3-difluoro-6-phenylhex-1-yn-1-yl)triisopropylsilane (3a)

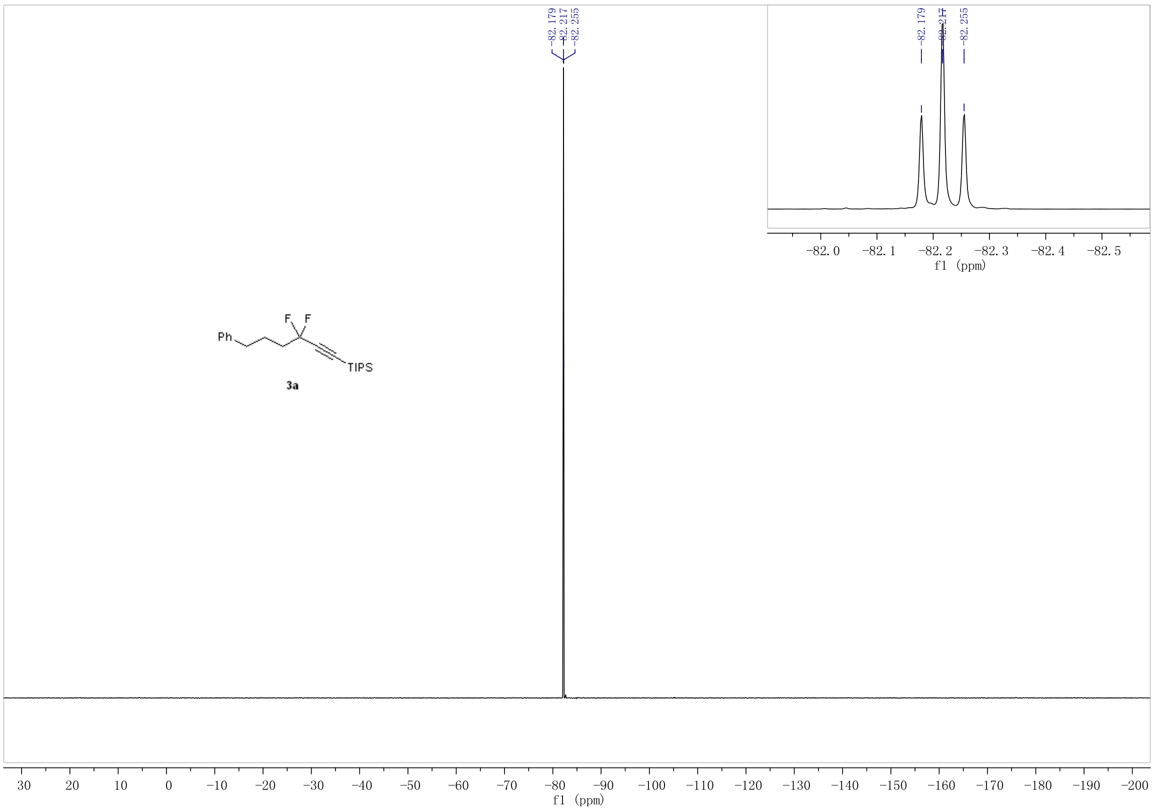

Supplementary Figure 36. ^{19}F NMR of (3, 3-difluoro-6-phenylhex-1-yn-1-yl)triisopropylsilane (3a)

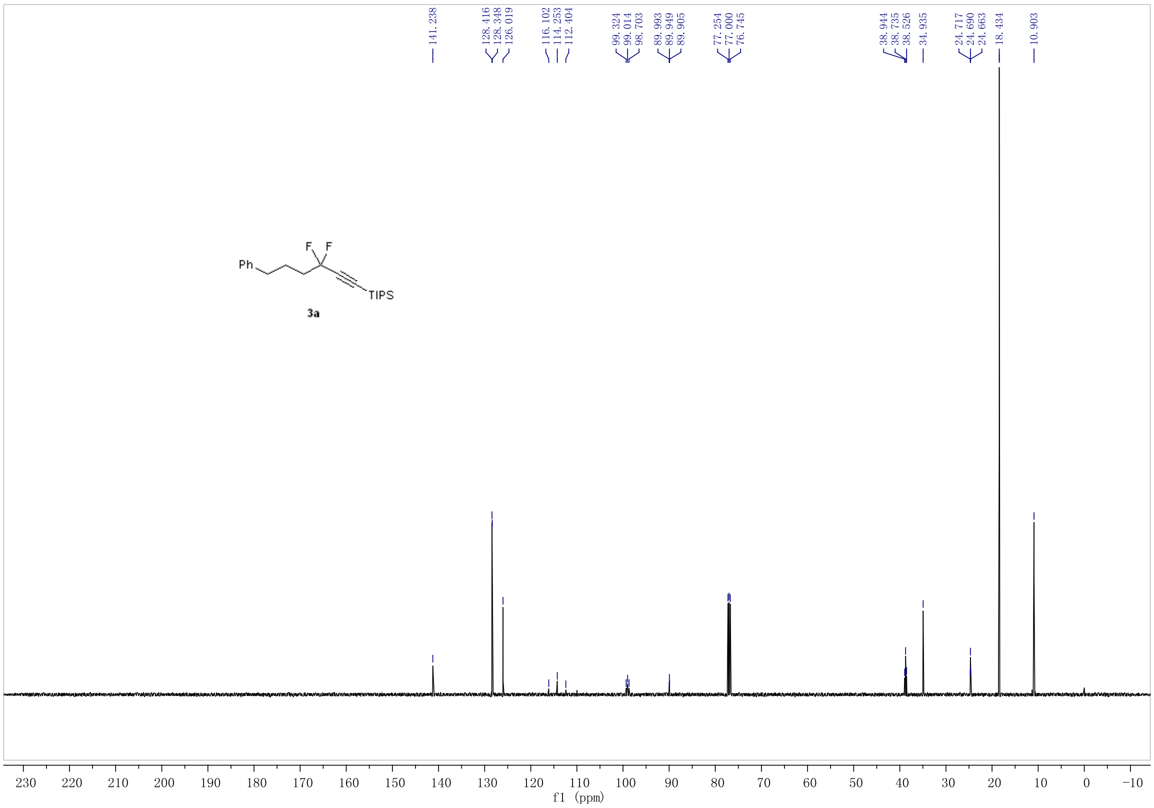

Supplementary Figure 37. **^{13}C NMR of (3, 3-difluoro-6-phenylhex-1-yn-1-yl)triisopropylsilane (3a)**

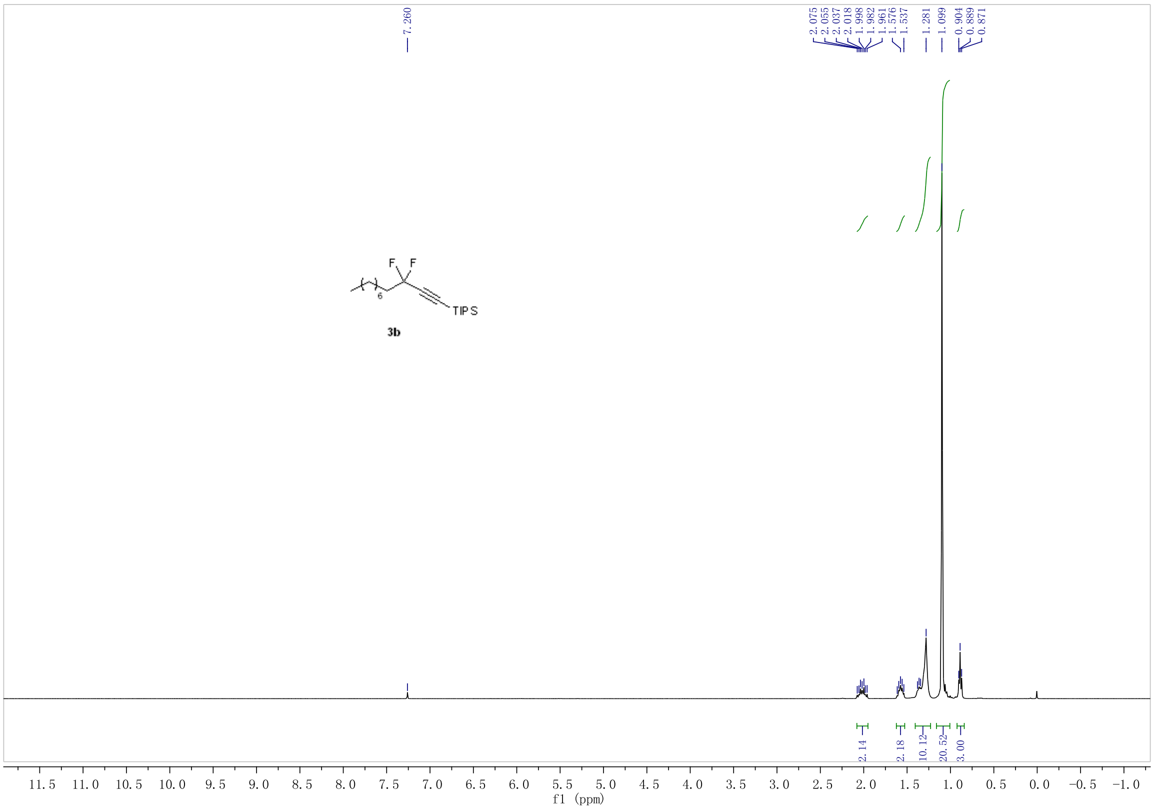

Supplementary Figure 38. **^{1}H NMR of (3,3-difluoroundec-1-yn-1-yl)triisopropylsilane (3b)**

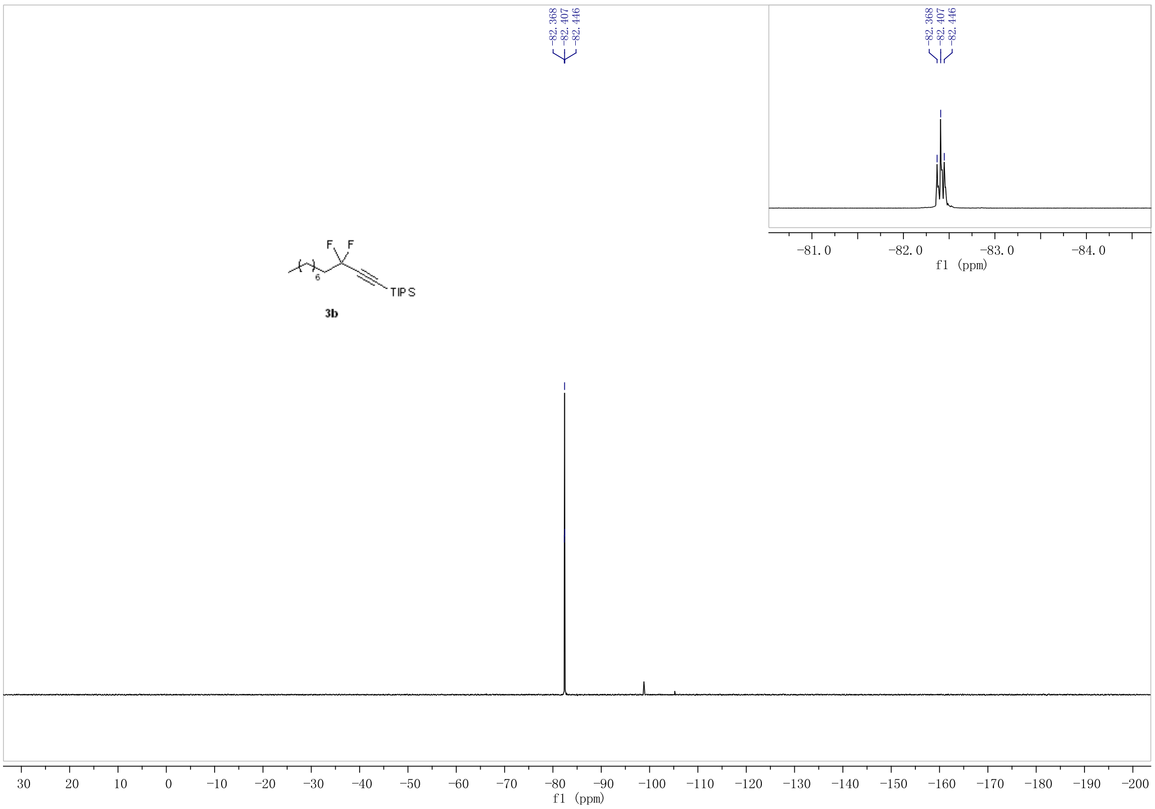

Supplementary Figure 39. ^{19}F NMR of (3,3-difluoroundec-1-yn-1-yl)triisopropylsilane (3b)

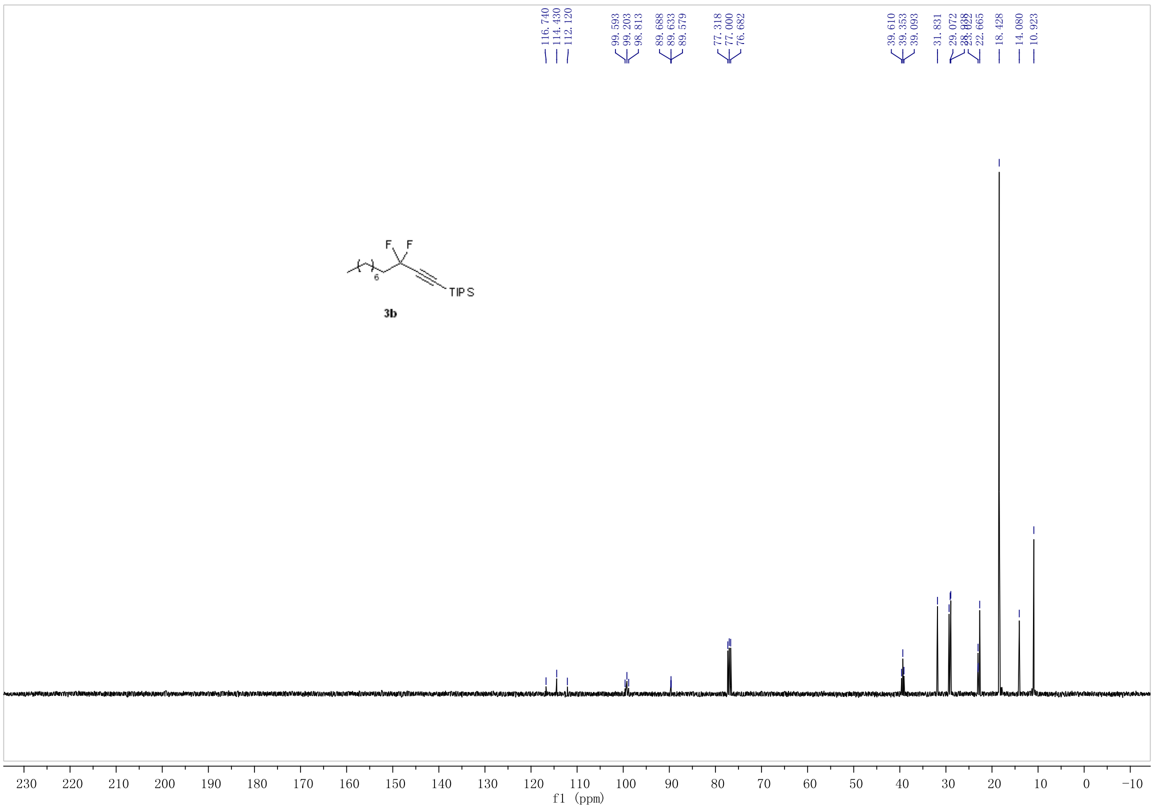

Supplementary Figure 40. ^{13}C NMR of (3,3-difluoroundec-1-yn-1-yl)triisopropylsilane (3b)

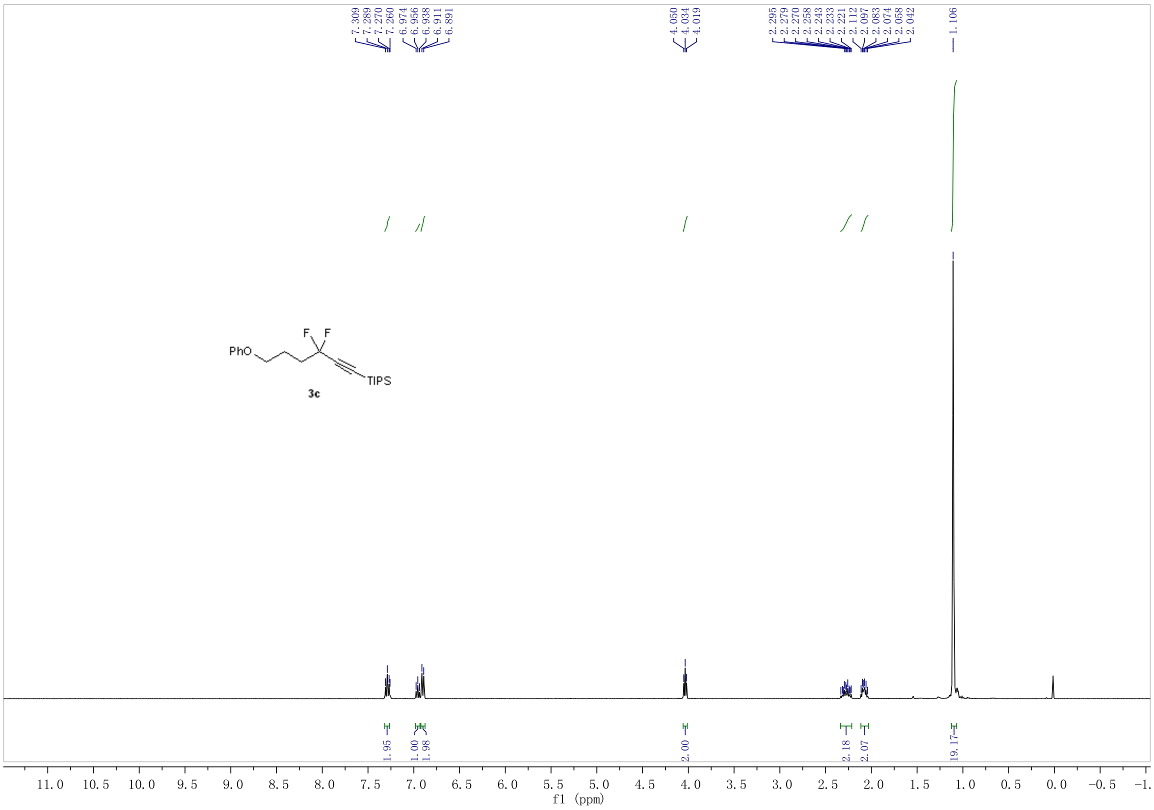

Supplementary Figure 41. ^{1}H NMR of (3, 3-difluoro-6-phenoxyhex-1-yn-1-yl)triisopropylsilane (3c)

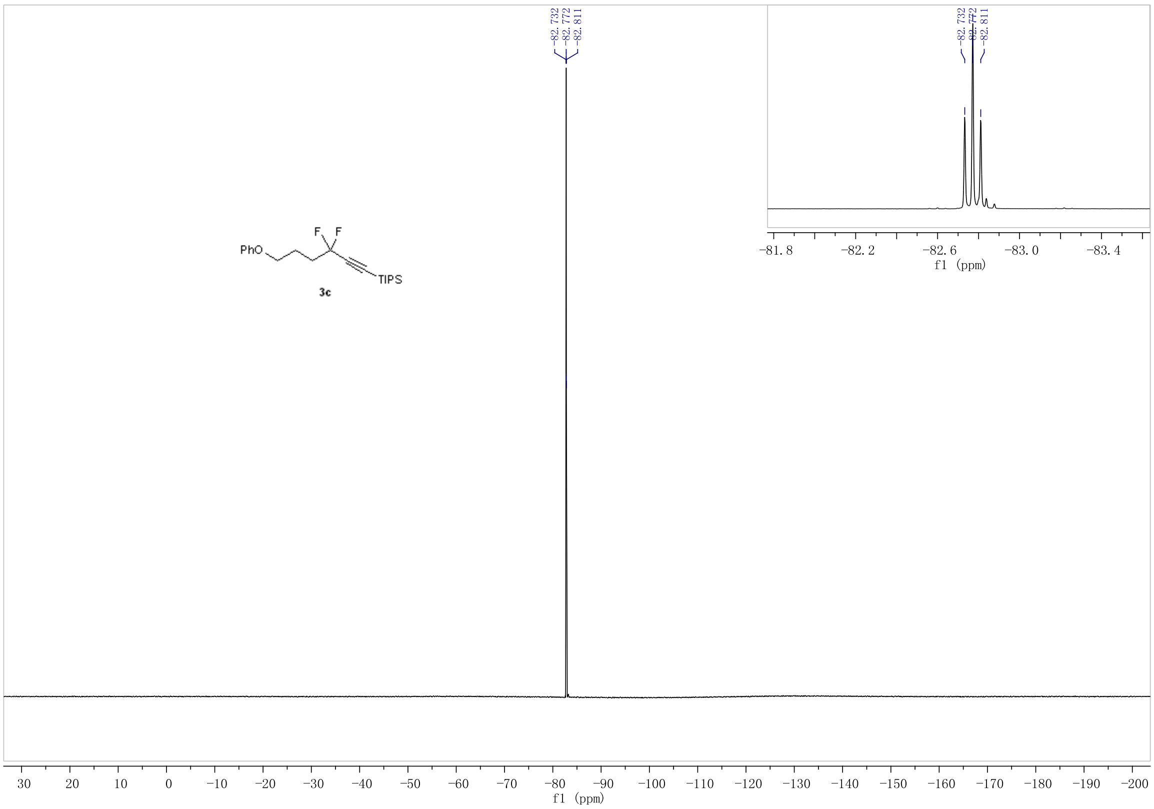

Supplementary Figure 42. ^{19}F NMR of (3, 3-difluoro-6-phenoxyhex-1-yn-1-yl)triisopropylsilane (3c)

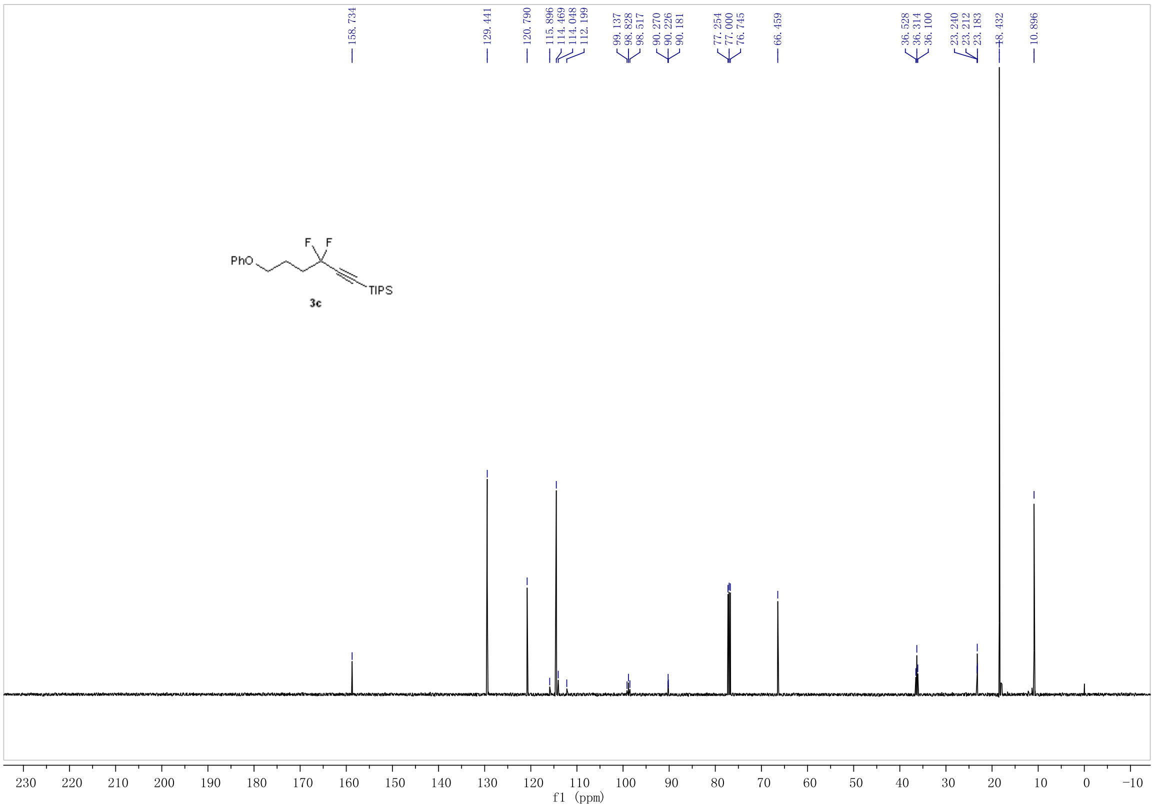

Supplementary Figure 43. ^{13}C NMR of (3, 3-difluoro-6-phenoxyhex-1-yn-1-yl)triisopropylsilane (3c)

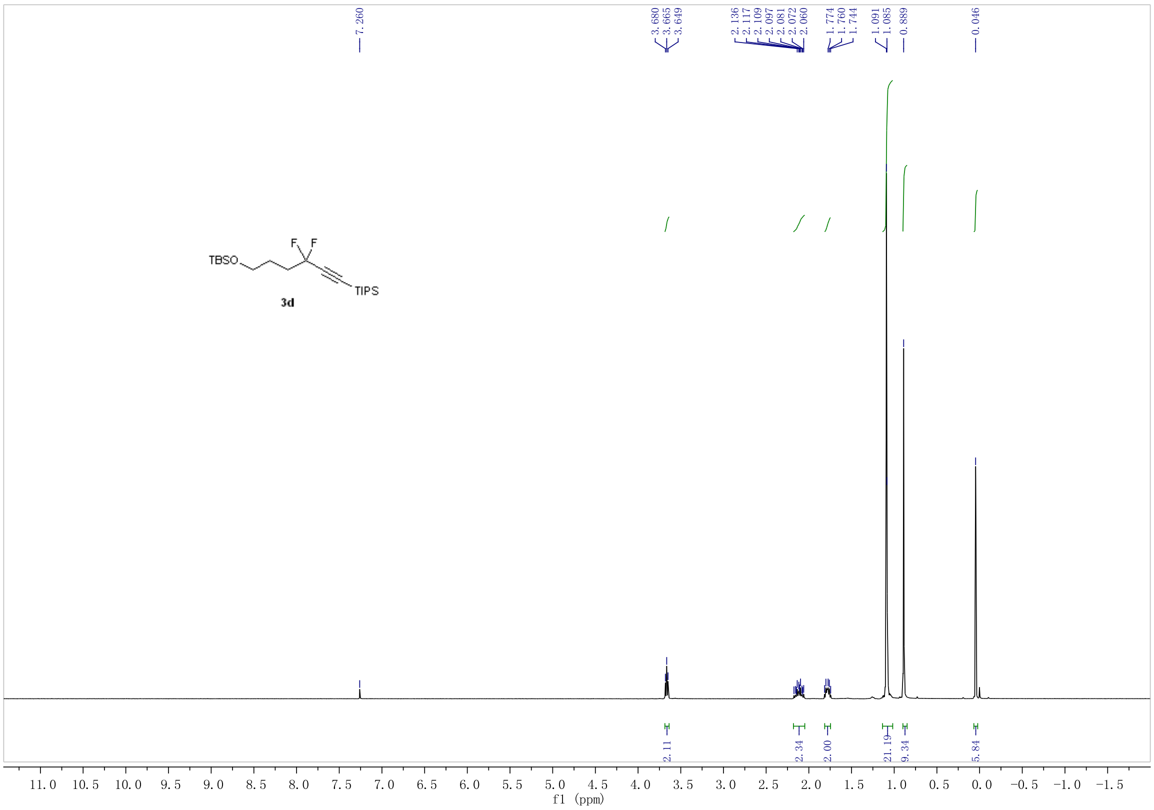

Supplementary Figure 44. ^{1}H NMR of *tert*-butyl((4,4-difluoro-6-(triisopropylsilyl)hex-5-yn-1-yl)oxy)dimethylsilane (3d)

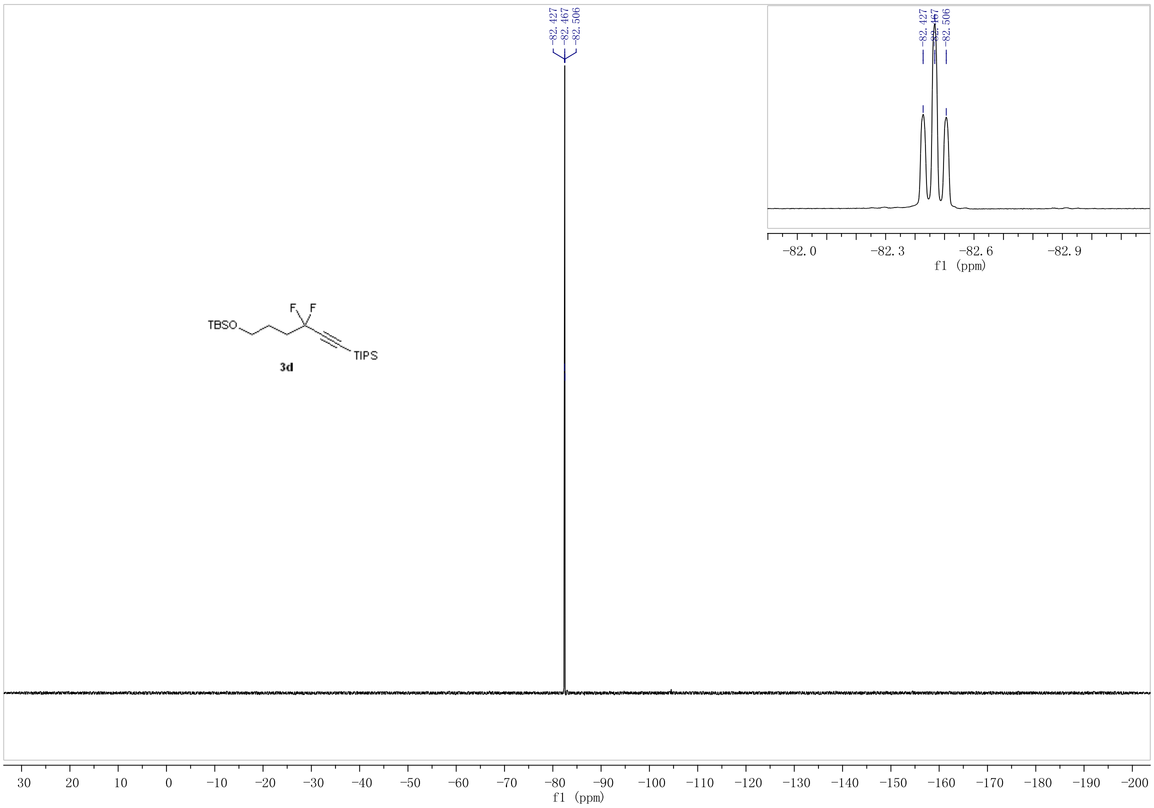

Supplementary Figure 45. ^{19}F NMR of *tert*-butyl((4,4-difluoro-6-(triisopropylsilyl)hex-5-yn-1-yl)oxy)dimethylsilane (3d)

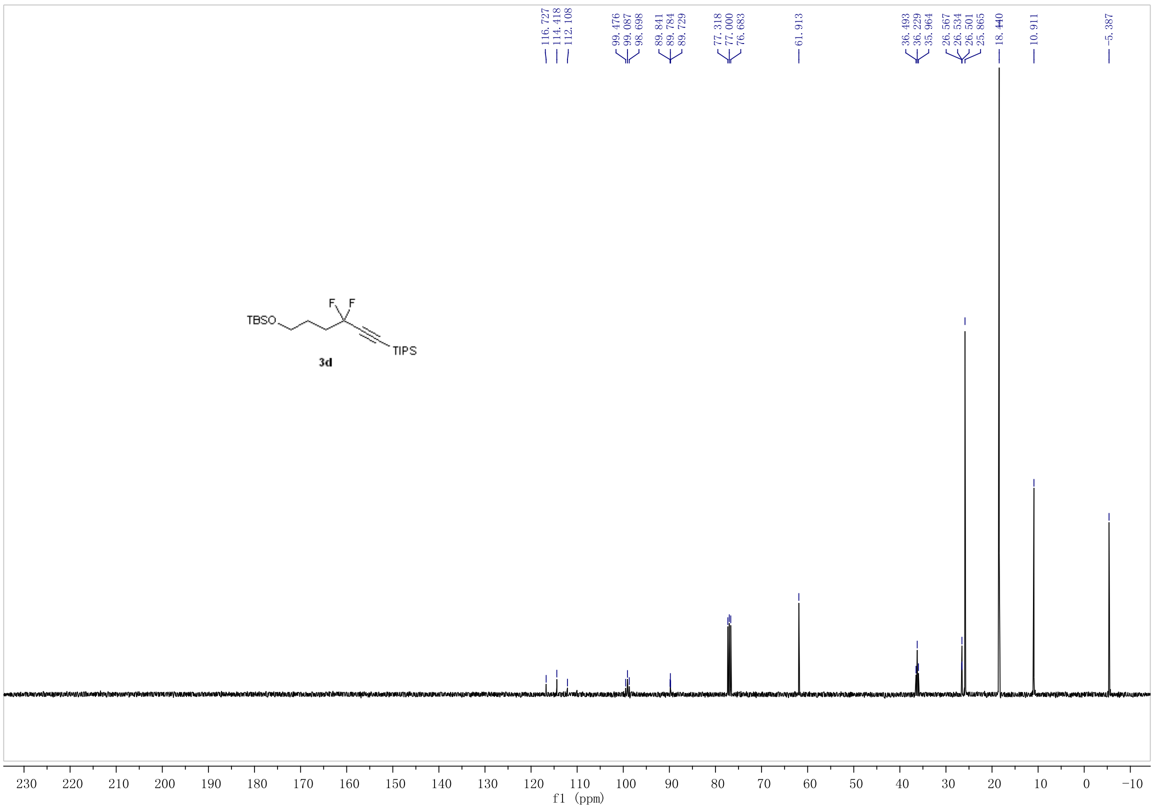

Supplementary Figure 46. ^{13}C NMR of *tert*-butyl((4,4-difluoro-6-(triisopropylsilyl)hex-5-yn-1-yl)oxy)dimethylsilane (3d)

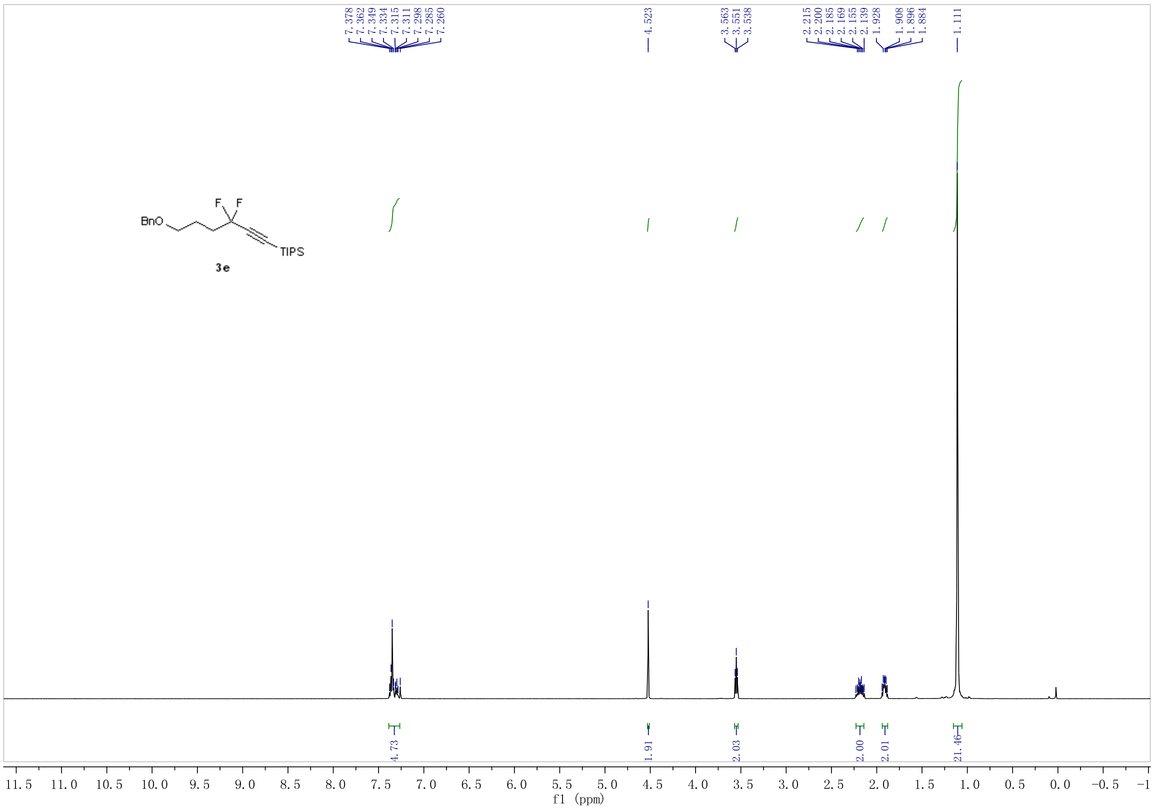

Supplementary Figure 47. ^{1}H NMR of (6-(benzyloxy)-3,3-difluorohex-1-yn-1-yl)triisopropylsilane (3e)

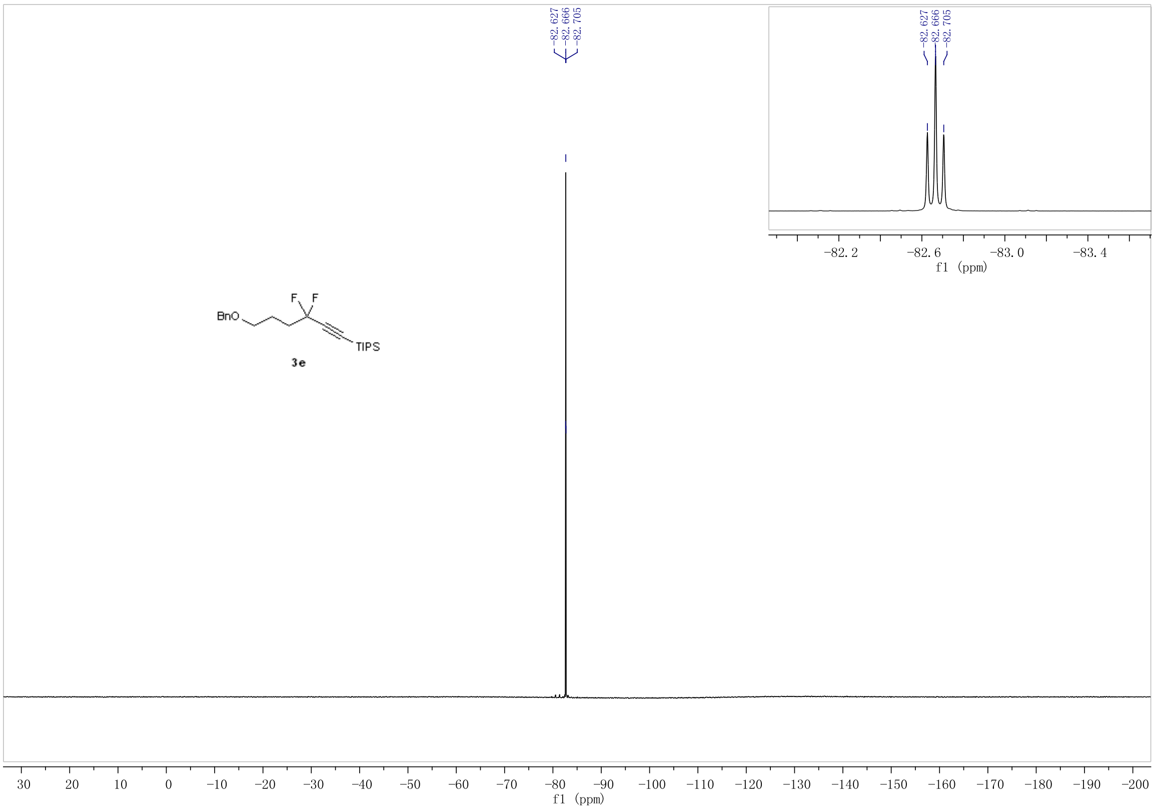

Supplementary Figure 48. ^{19}F NMR of (6-(benzyloxy)-3,3-difluorohex-1-yn-1-yl)triisopropylsilane (3e)

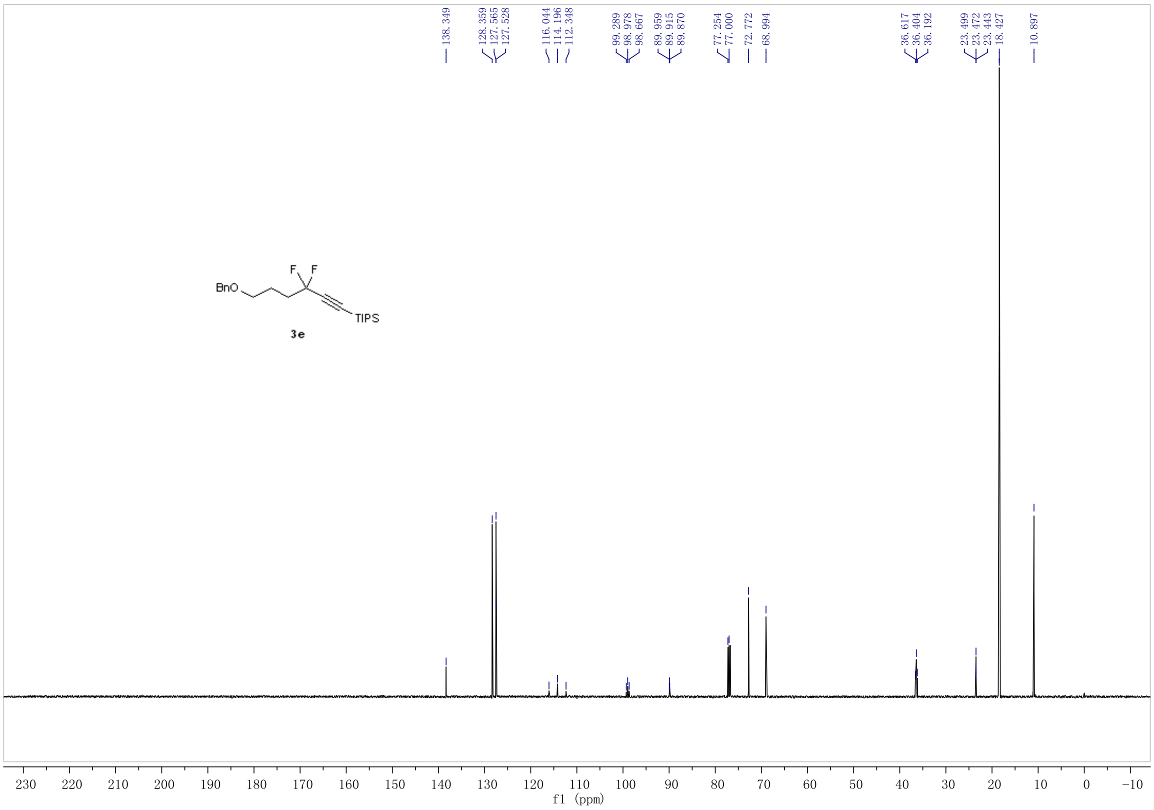

Supplementary Figure 49. ^{13}C NMR of (6-(benzyloxy)-3,3-difluorohex-1-yn-1-yl)triisopropylsilane (3e)

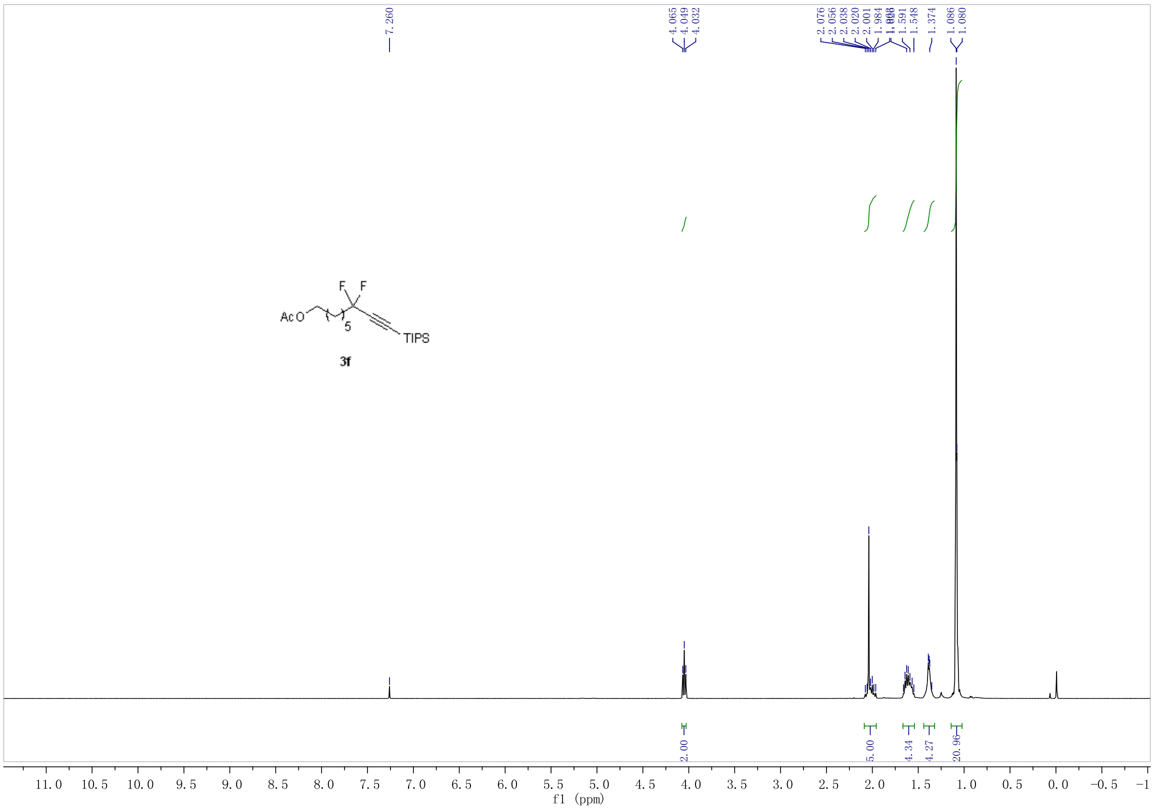

Supplementary Figure 50. ^{1}H NMR of 7, 7-difluoro-9-(triisopropylsilyl)non-8-yn-1-yl acetate (3f)

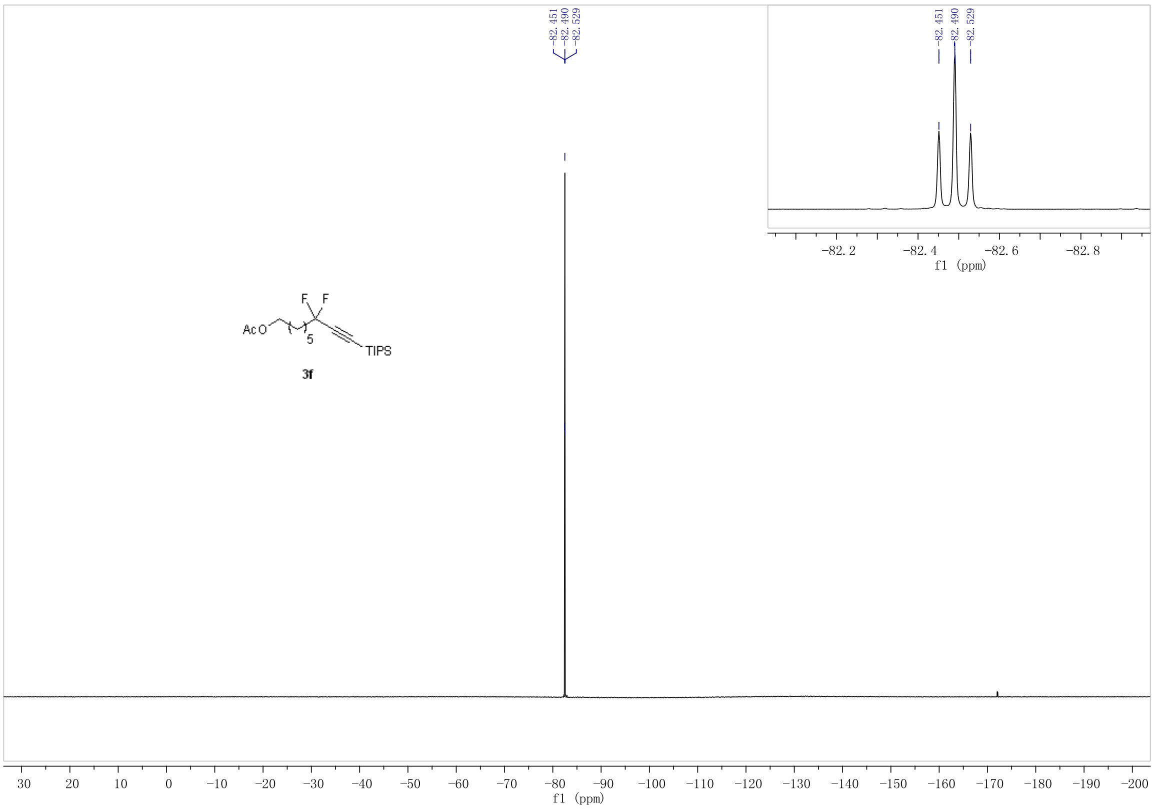

Supplementary Figure 51. ^{19}F NMR of 7, 7-difluoro-9-(triisopropylsilyl)non-8-yn-1-yl acetate (3f)

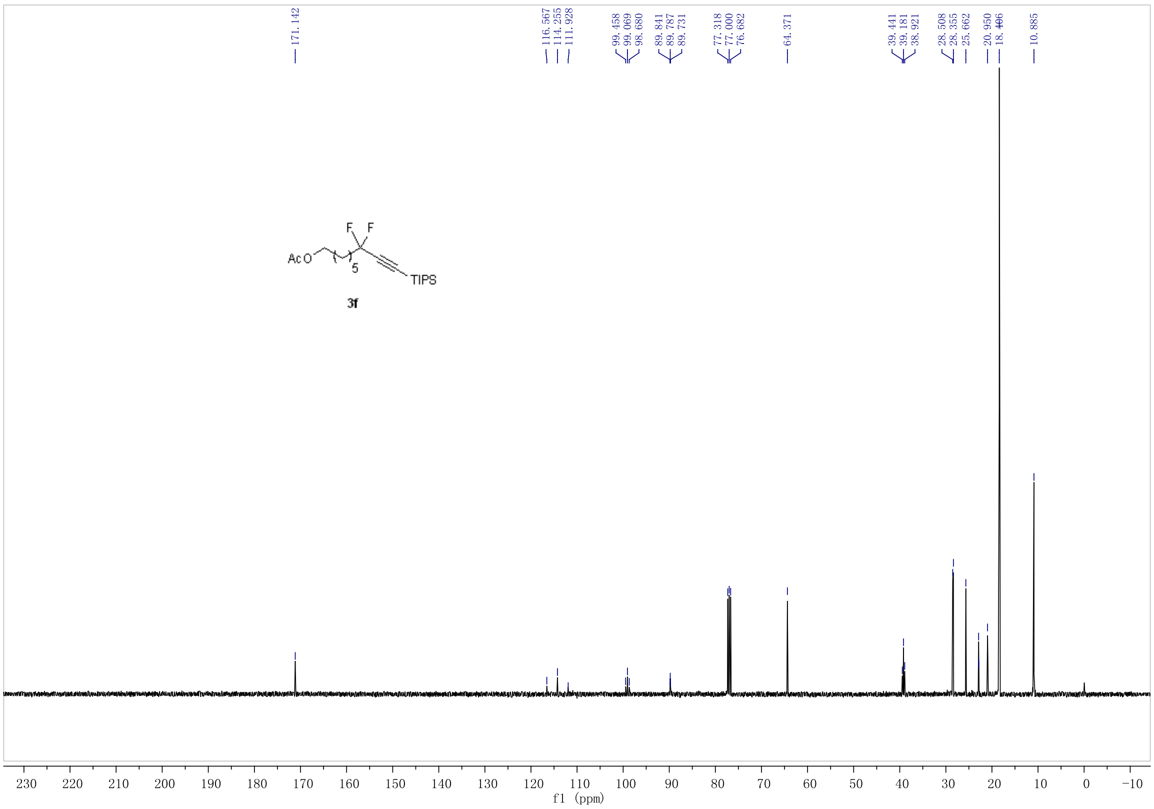

Supplementary Figure 52. ^{13}C NMR of 7, 7-difluoro-9-(triisopropylsilyl)non-8-yn-1-yl acetate (3f)

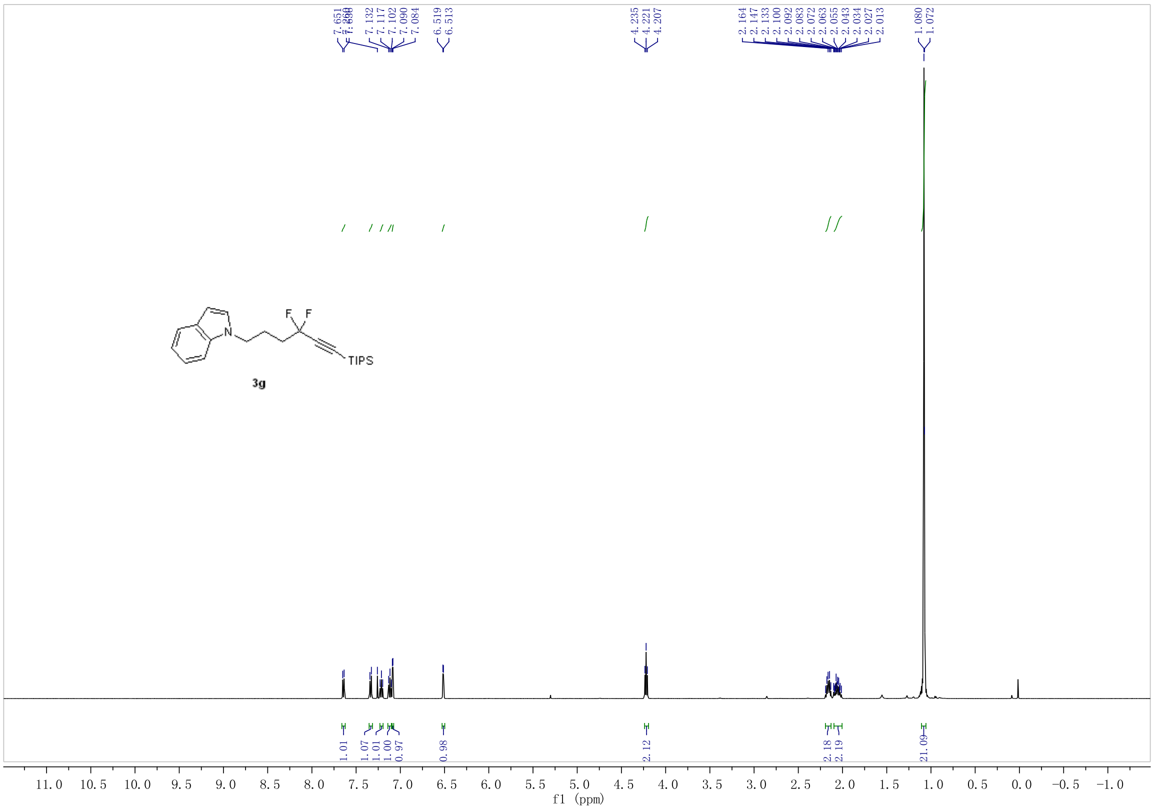

Supplementary Figure 53. ^{1}H NMR of 1-(4,4-difluoro-6-(triisopropylsilyl)hex-5-yn-1-yl)-1H-indole (3g)

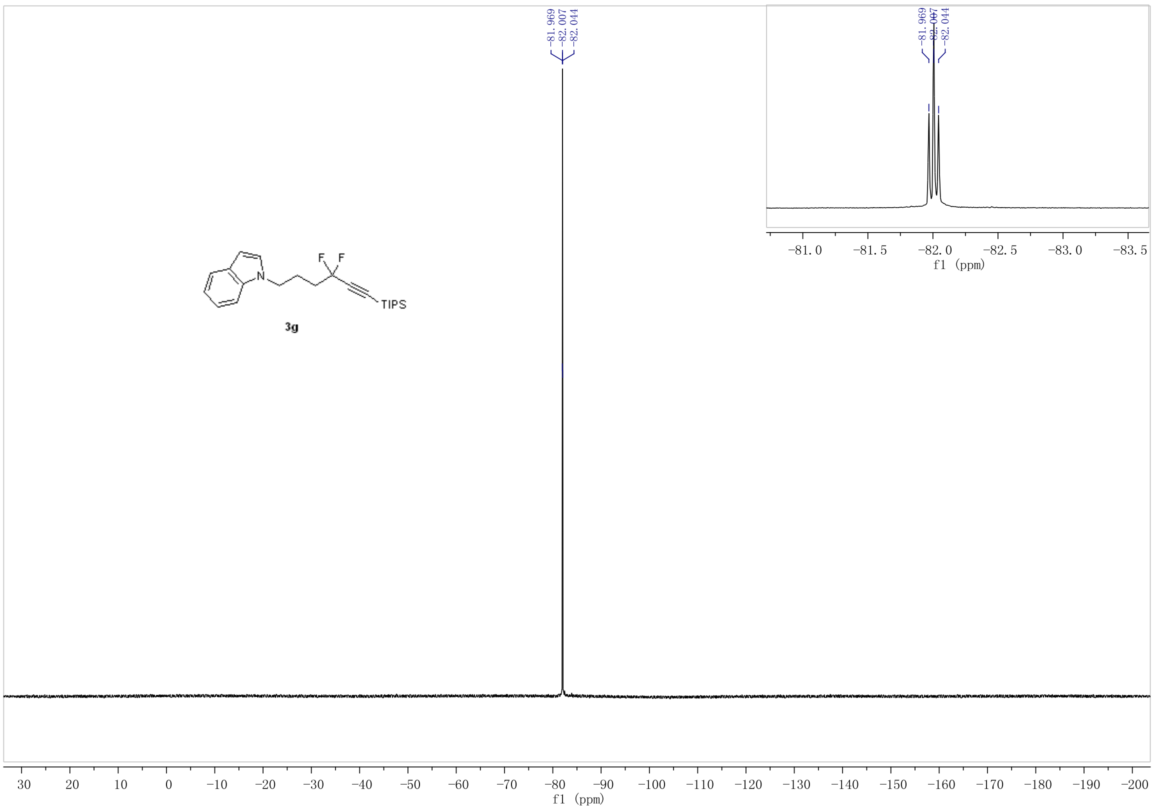

Supplementary Figure 54. ^{19}F NMR of 1-(4,4-difluoro-6-(triisopropylsilyl)hex-5-yn-1-yl)-1H-indole (3g)

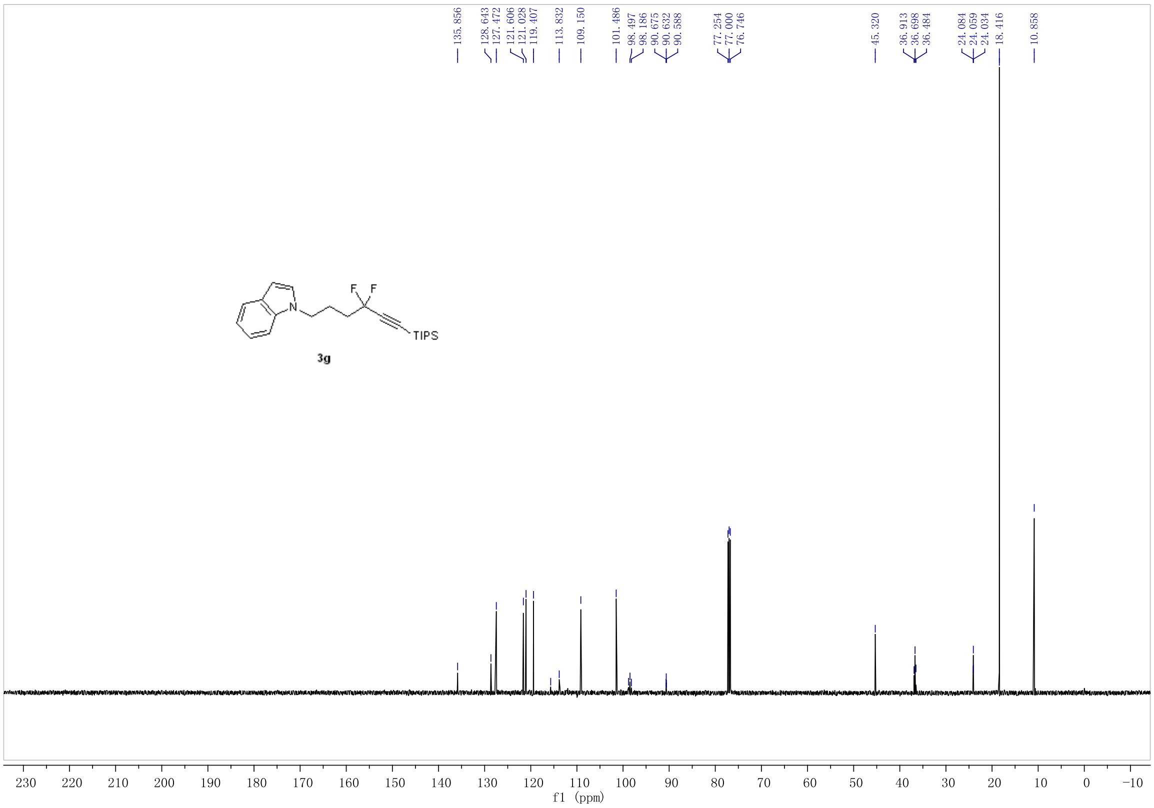

Supplementary Figure 55. ^{13}C NMR of 1-(4,4-difluoro-6-(triisopropylsilyl)hex-5-yn-1-yl)-1H-indole (3g)

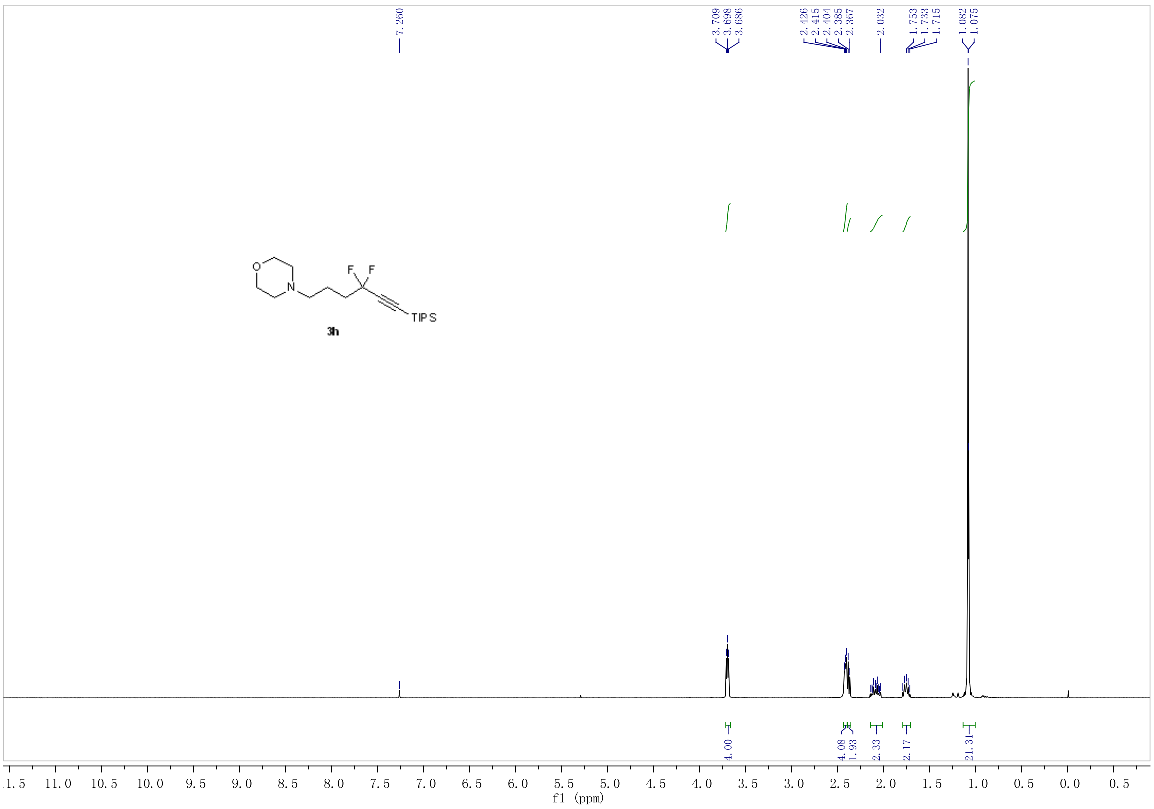

Supplementary Figure 56. ^{1}H NMR of 4-(4,4-difluoro-6-(triisopropylsilyl)hex-5-yn-1-yl)morpholine (3h)

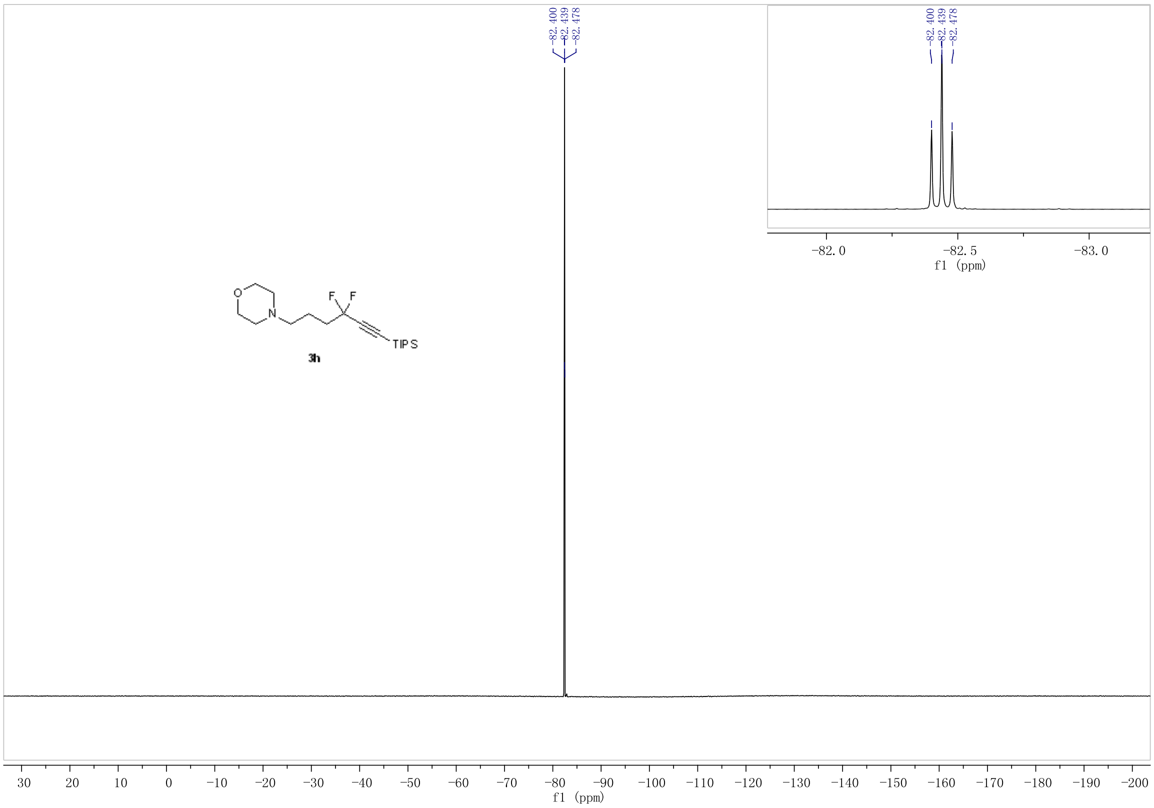

Supplementary Figure 57. ^{19}F NMR of 4-(4,4-difluoro-6-(triisopropylsilyl)hex-5-yn-1-yl)morpholine (3h)

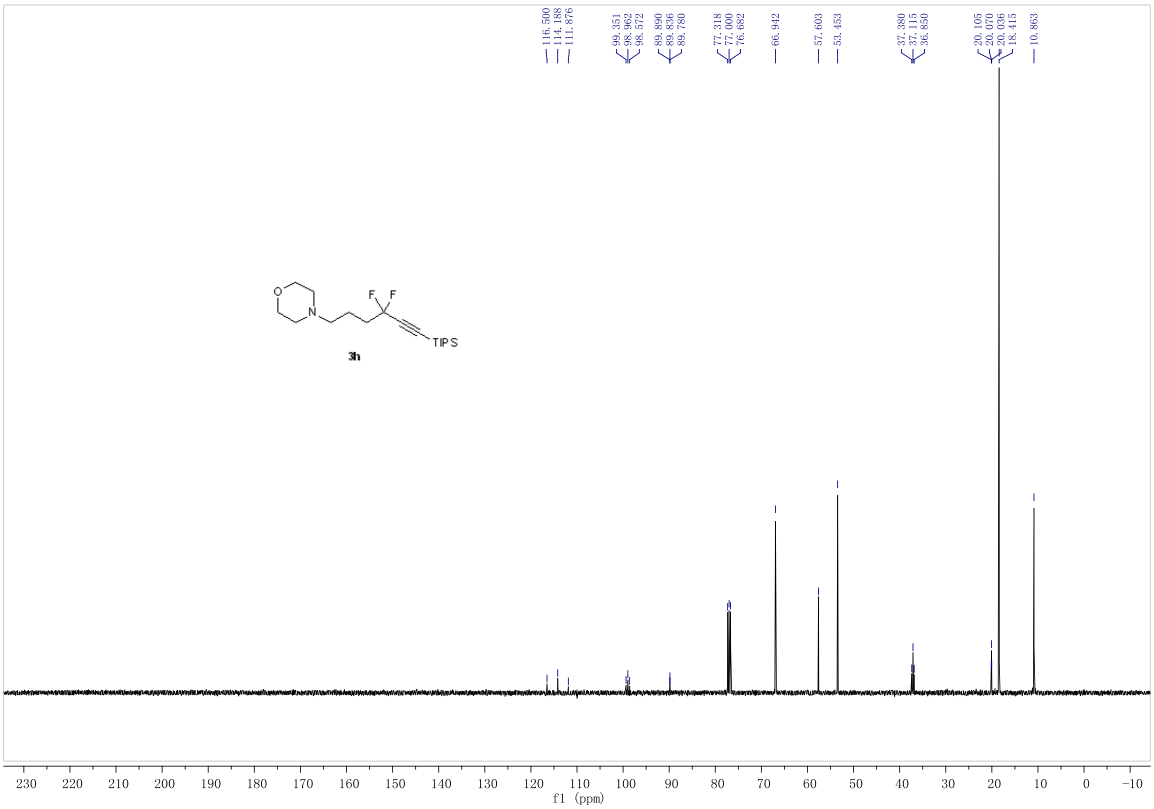

Supplementary Figure 58. ^{13}C NMR of 4-(4,4-difluoro-6-(triisopropylsilyl)hex-5-yn-1-yl)morpholine (3h)

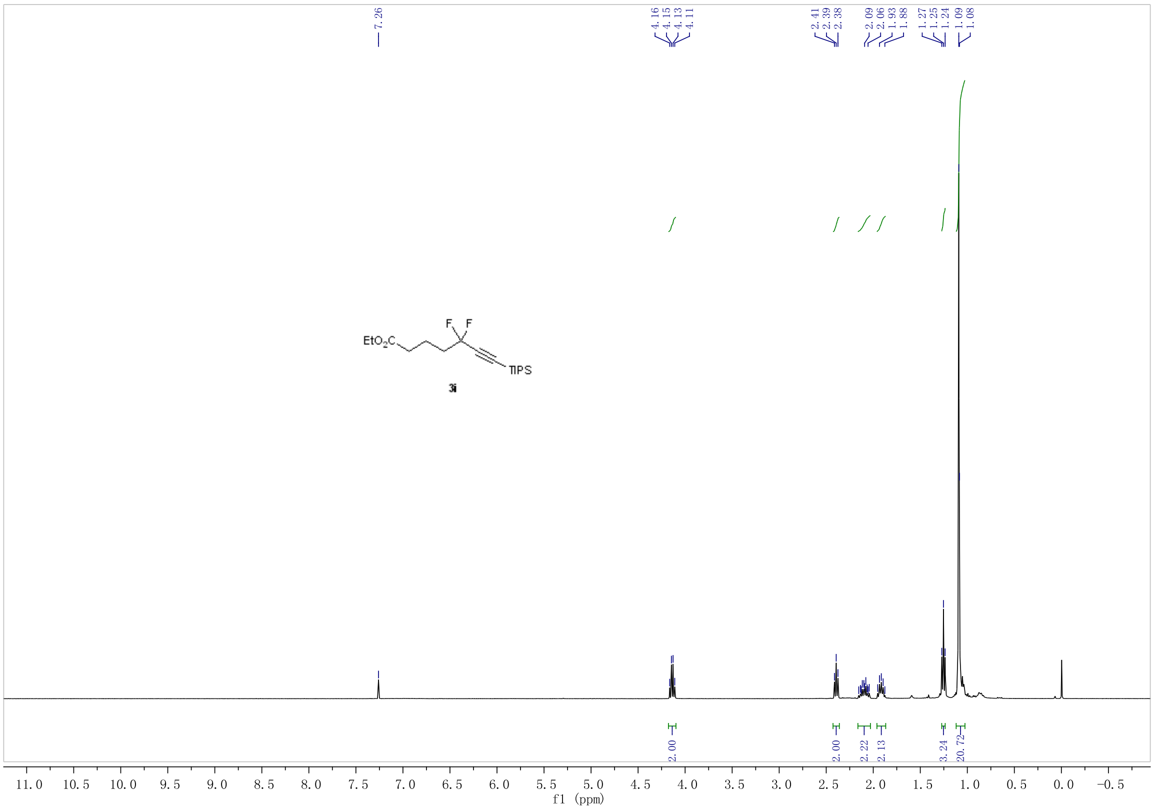

Supplementary Figure 59. ^{1}H NMR of ethyl 5, 5-difluoro-7-(triisopropylsilyl)hept-6-ynoate (3i)

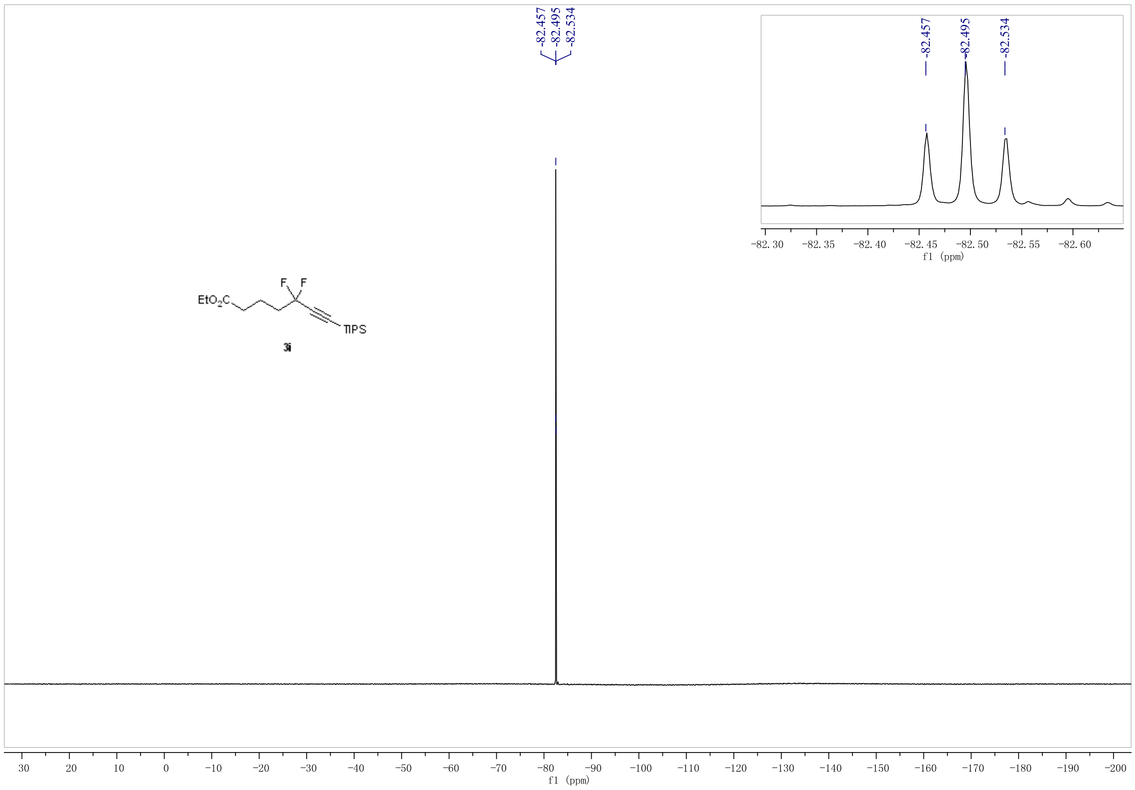

Supplementary Figure 60. ^{19}F NMR of ethyl 5, 5-difluoro-7-(triisopropylsilyl)hept-6-ynoate (3i)

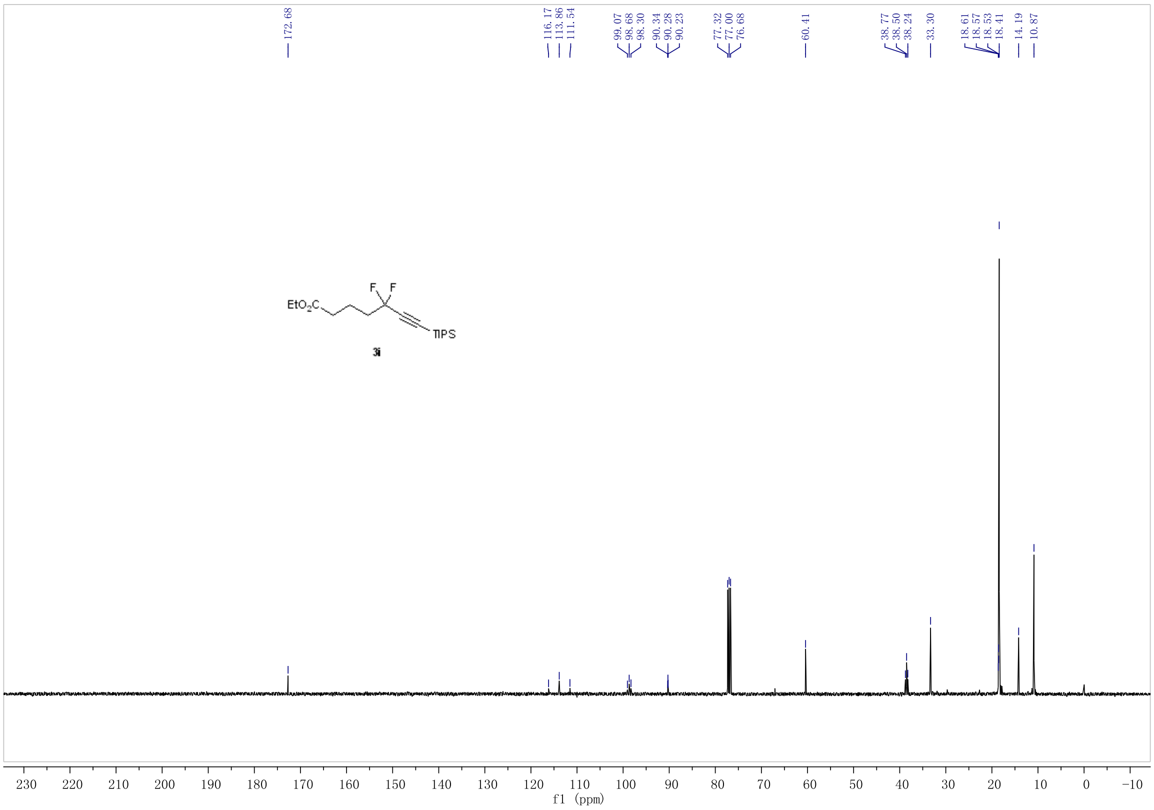

Supplementary Figure 61. ^{13}**C NMR of ethyl 5, 5-difluoro-7-(triisopropylsilyl)hept-6-ynoate (3i)**

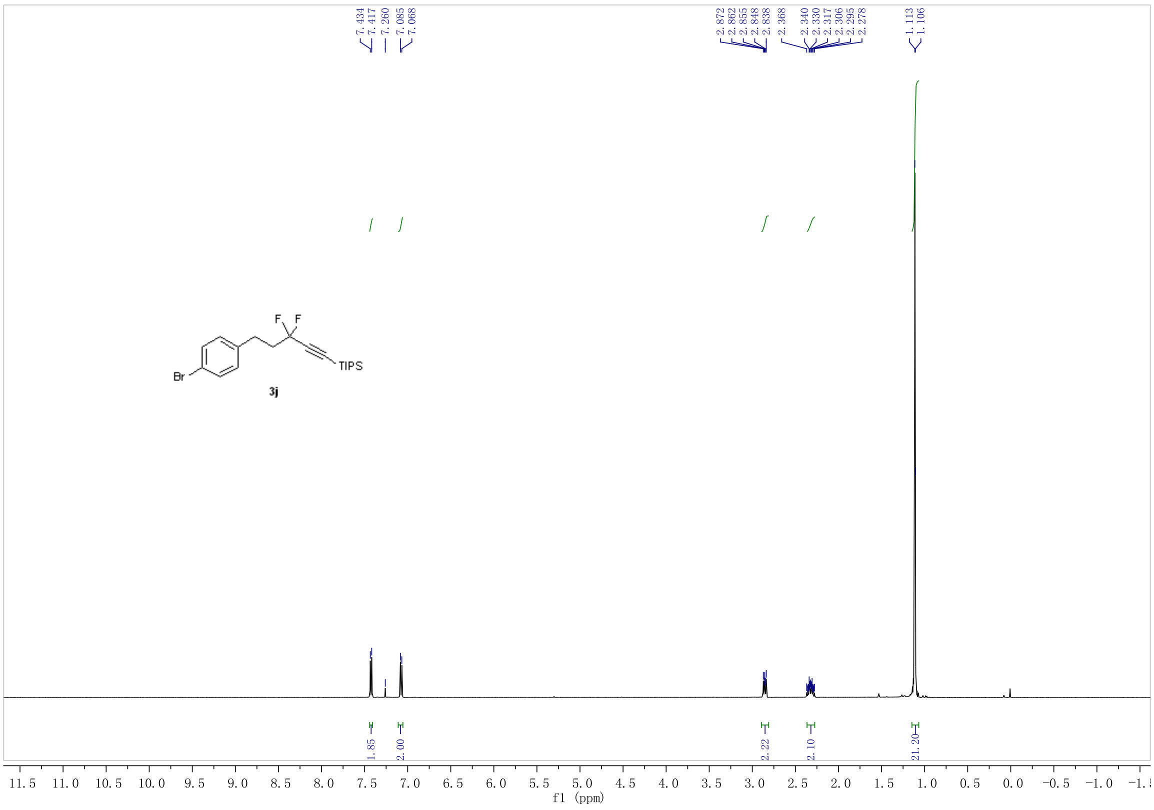

Supplementary Figure 62. ^{1}**H NMR of (5-(4-bromophenyl)-3,3-difluoropent-1-yn-1-yl)triisopropylsilane (3j)**

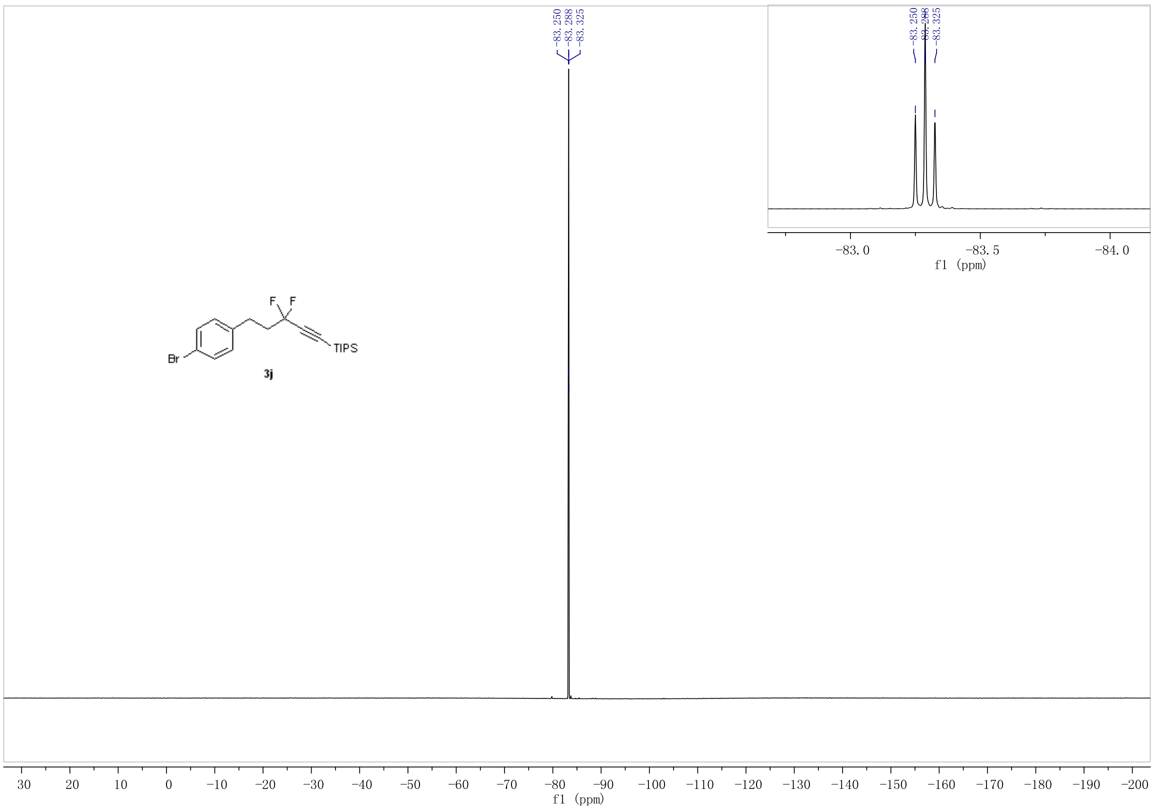

Supplementary Figure 63. ^{19}F NMR of (5-(4-bromophenyl)-3,3-difluoropent-1-yn-1-yl)triisopropylsilane (3j)

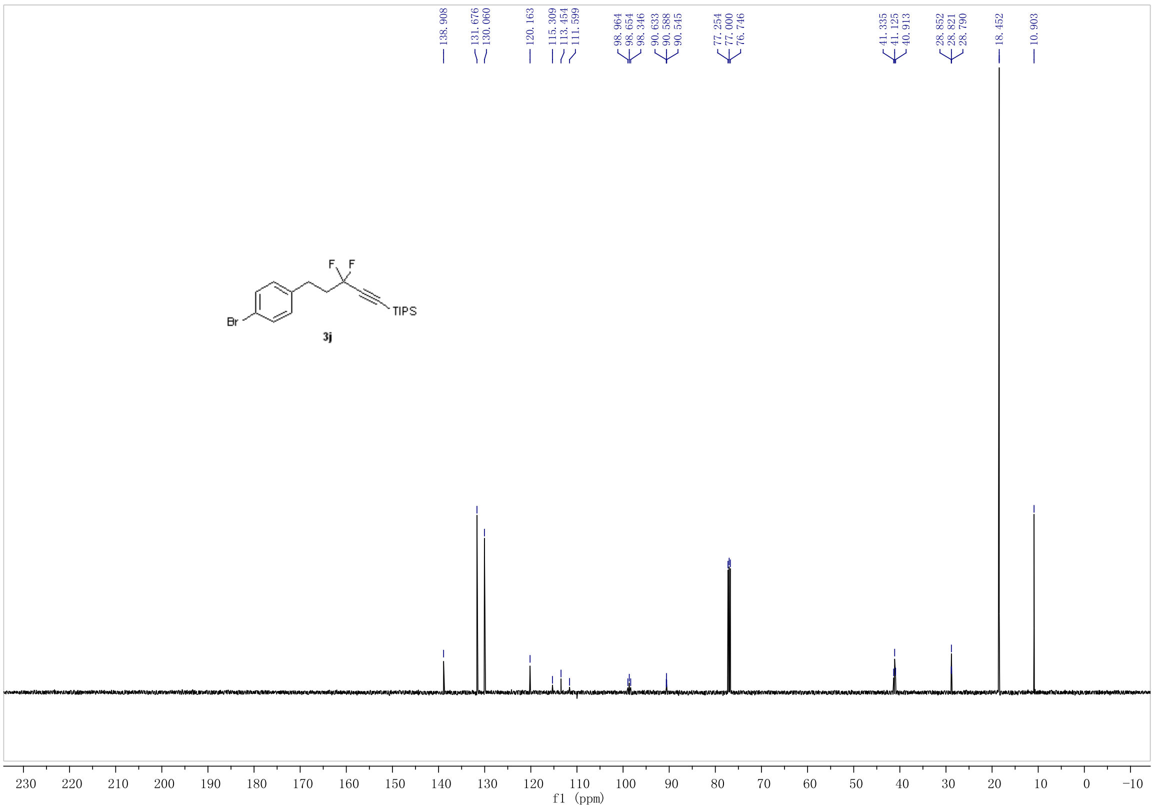

Supplementary Figure 64. ^{13}C NMR of (5-(4-bromophenyl)-3,3-difluoropent-1-yn-1-yl)triisopropylsilane (3j)

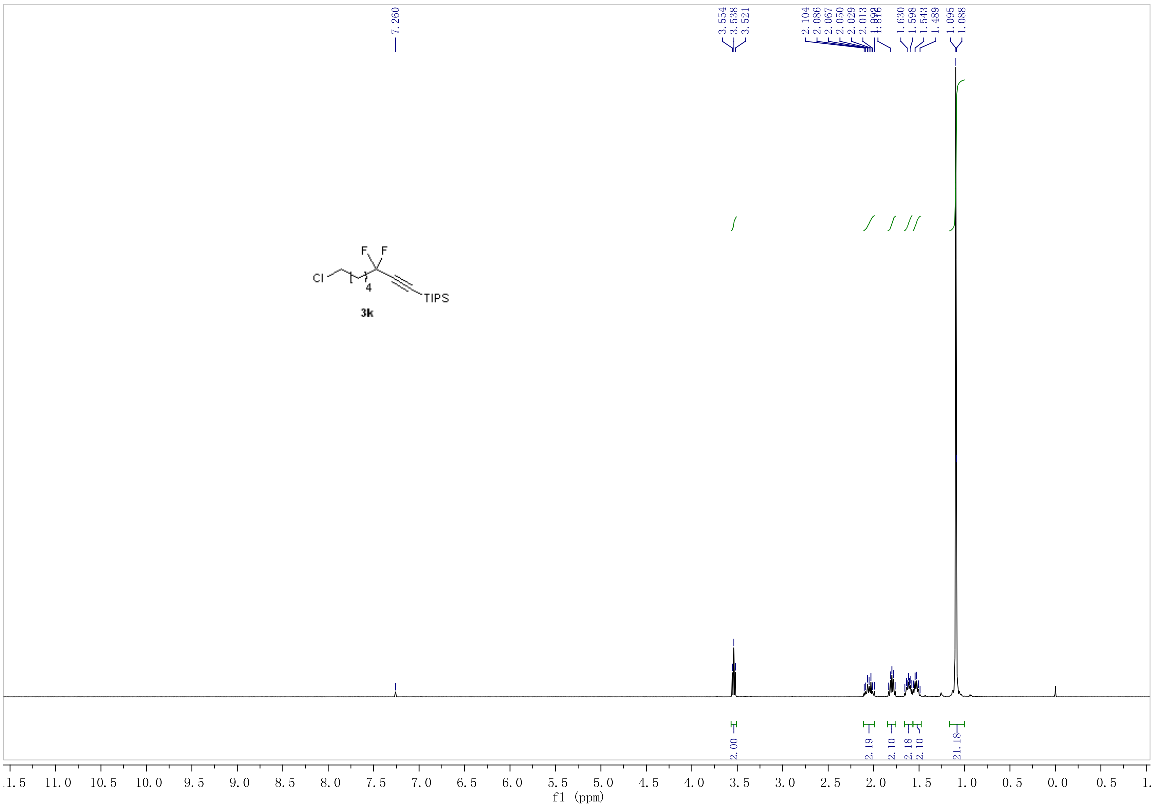

Supplementary Figure 65. ^{1}H NMR of (8-chloro-3,3-difluorooct-1-yn-1-yl)triisopropylsilane (3k)

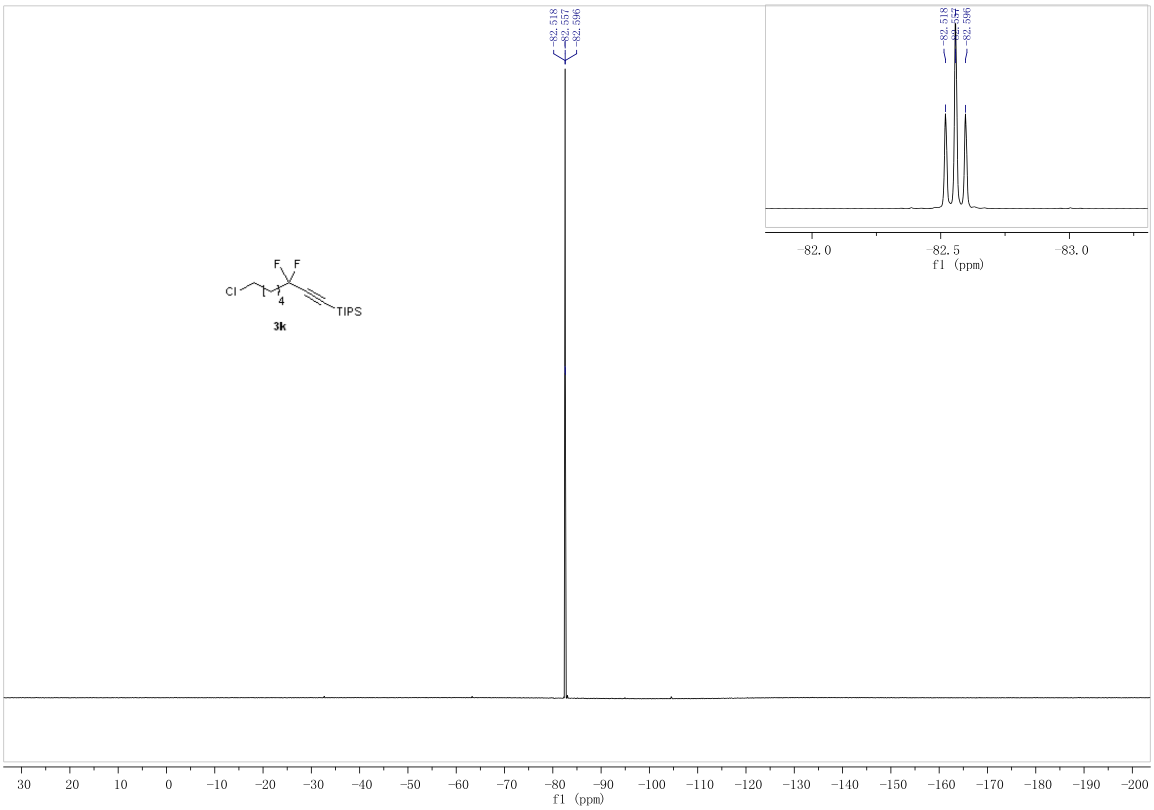

Supplementary Figure 66. ^{19}F NMR of (8-chloro-3,3-difluorooct-1-yn-1-yl)triisopropylsilane (3k)

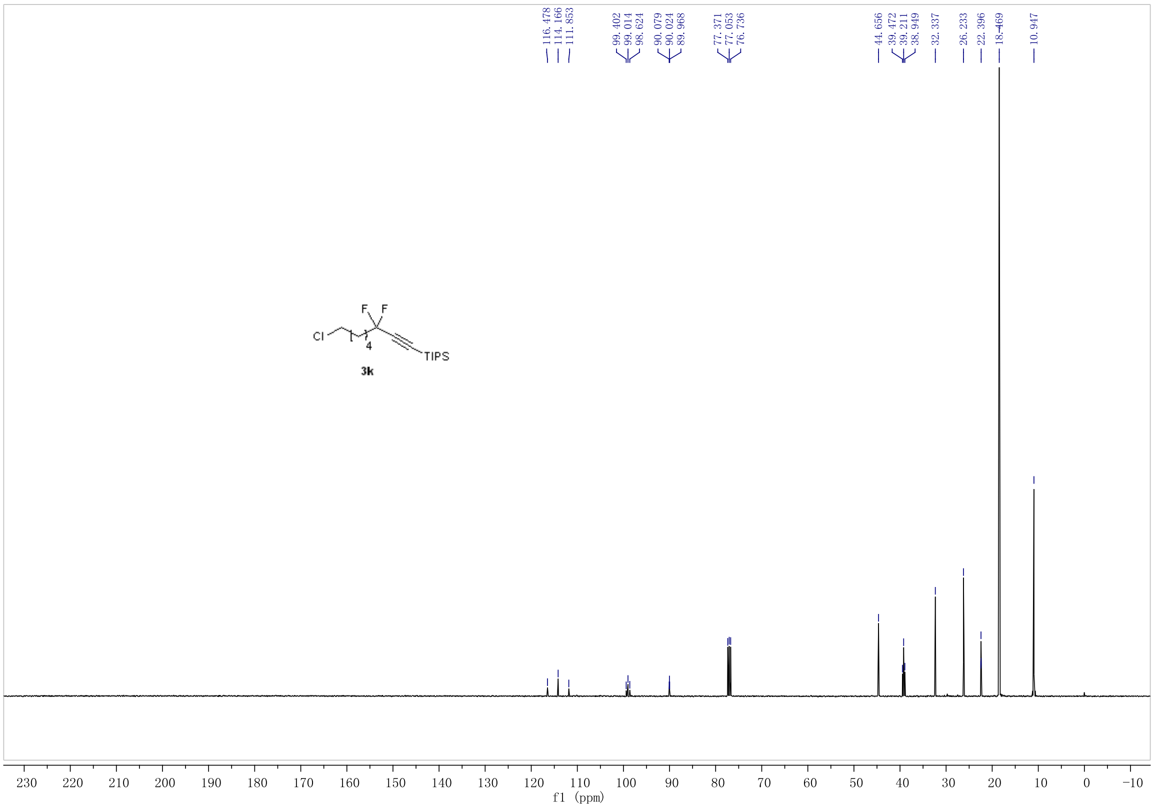

Supplementary Figure 67. ^{13}C NMR of (8-chloro-3,3-difluorooct-1-yn-1-yl)triisopropylsilane (3k)

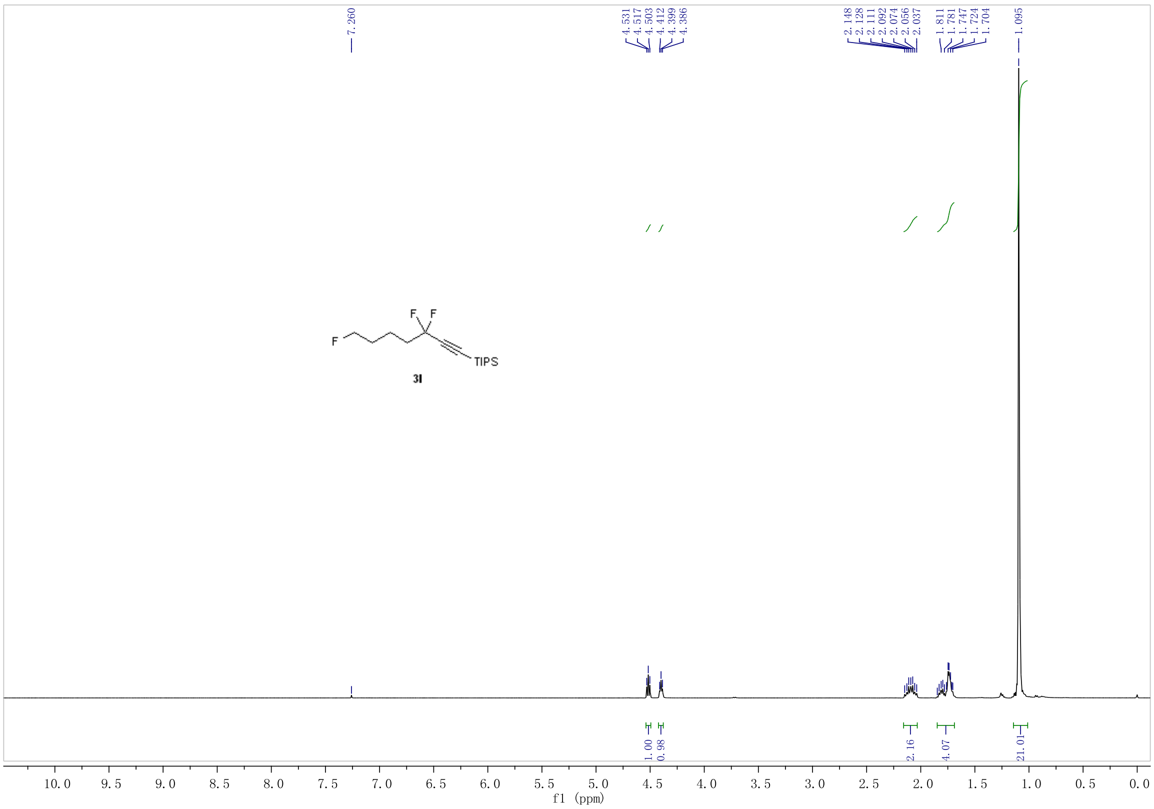

Supplementary Figure 68. ^{1}H NMR of triisopropyl(3,3,7-trifluorohept-1-yn-1-yl)silane (3l)

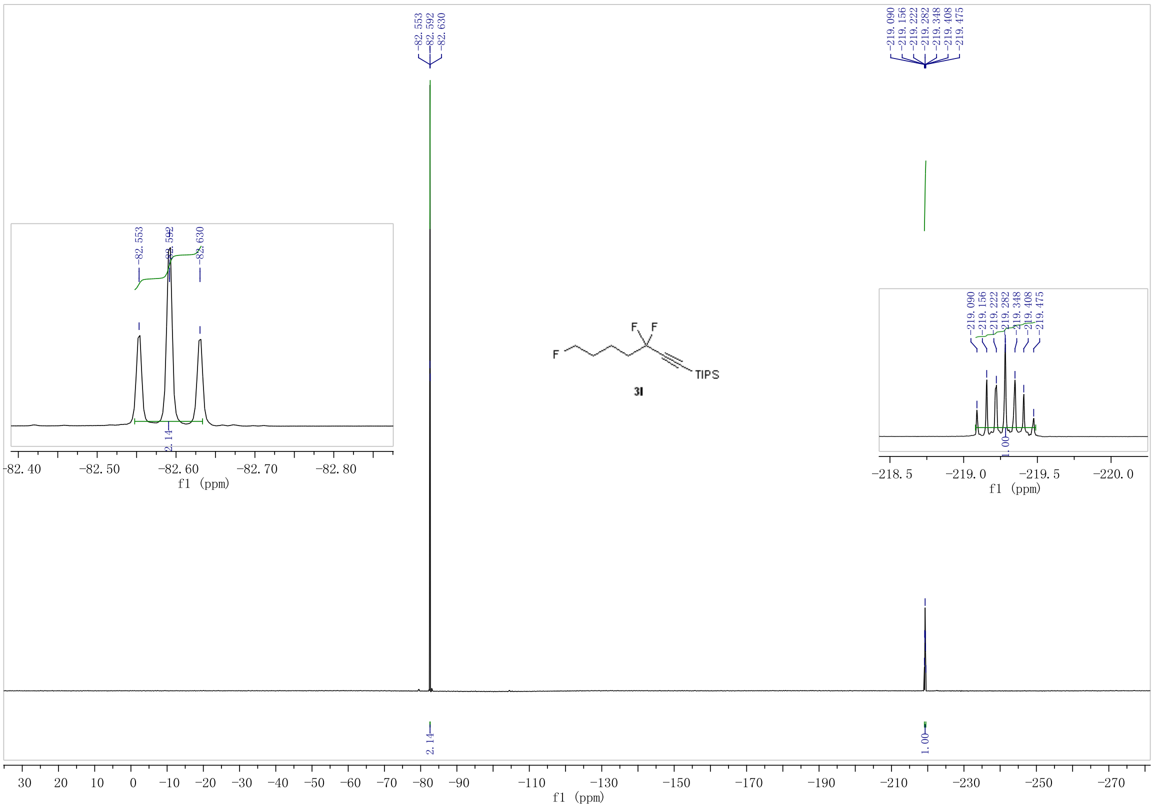

Supplementary Figure 69. ^{19}F NMR of triisopropyl(3,3,7-trifluorohept-1-yn-1-yl)silane (3l)

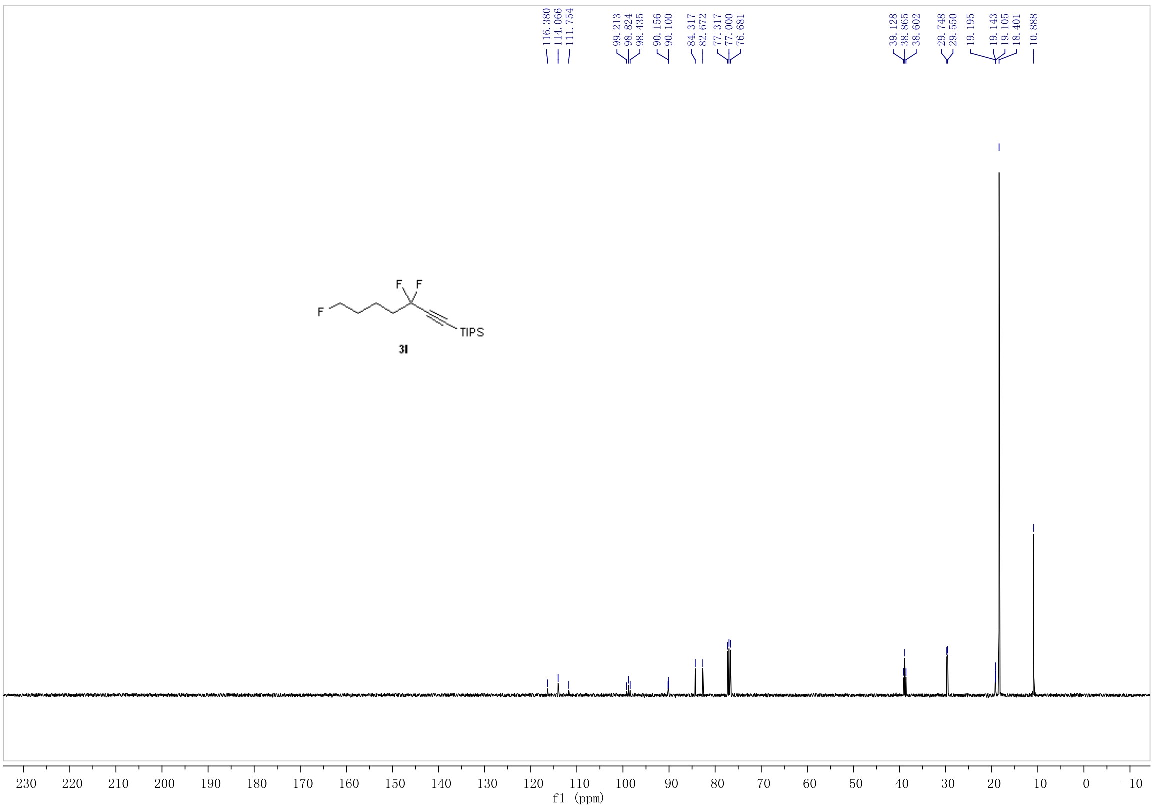

Supplementary Figure 70. ^{13}C NMR of triisopropyl(3,3,7-trifluorohept-1-yn-1-yl)silane (3l)

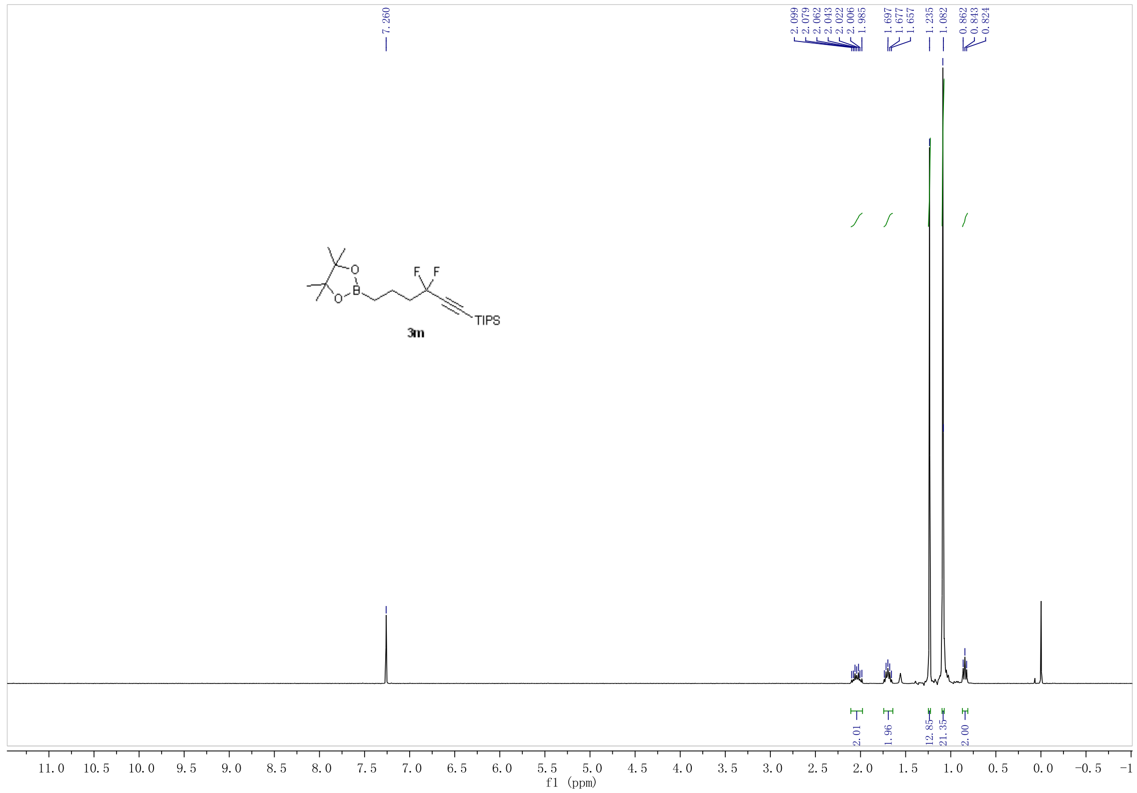

Supplementary Figure 71. ^{1}H NMR of

(3,3-difluoro-6-(4,4,5,5-tetramethyl-1,3,2-dioxaborolan-2-yl)hex-1-yn-1-yl)triisopropylsilane (3m)

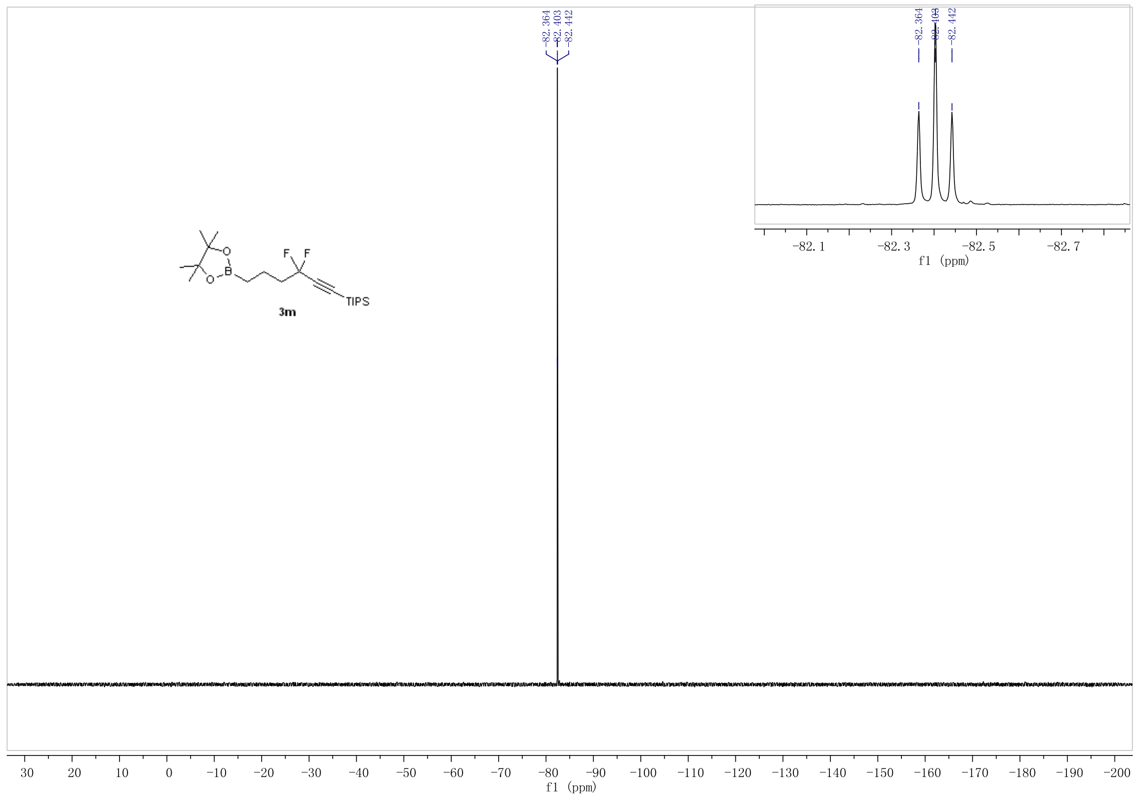

Supplementary Figure 72. ^{19}F NMR of

(3,3-difluoro-6-(4,4,5,5-tetramethyl-1,3,2-dioxaborolan-2-yl)hex-1-yn-1-yl)triisopropylsilane (3m)

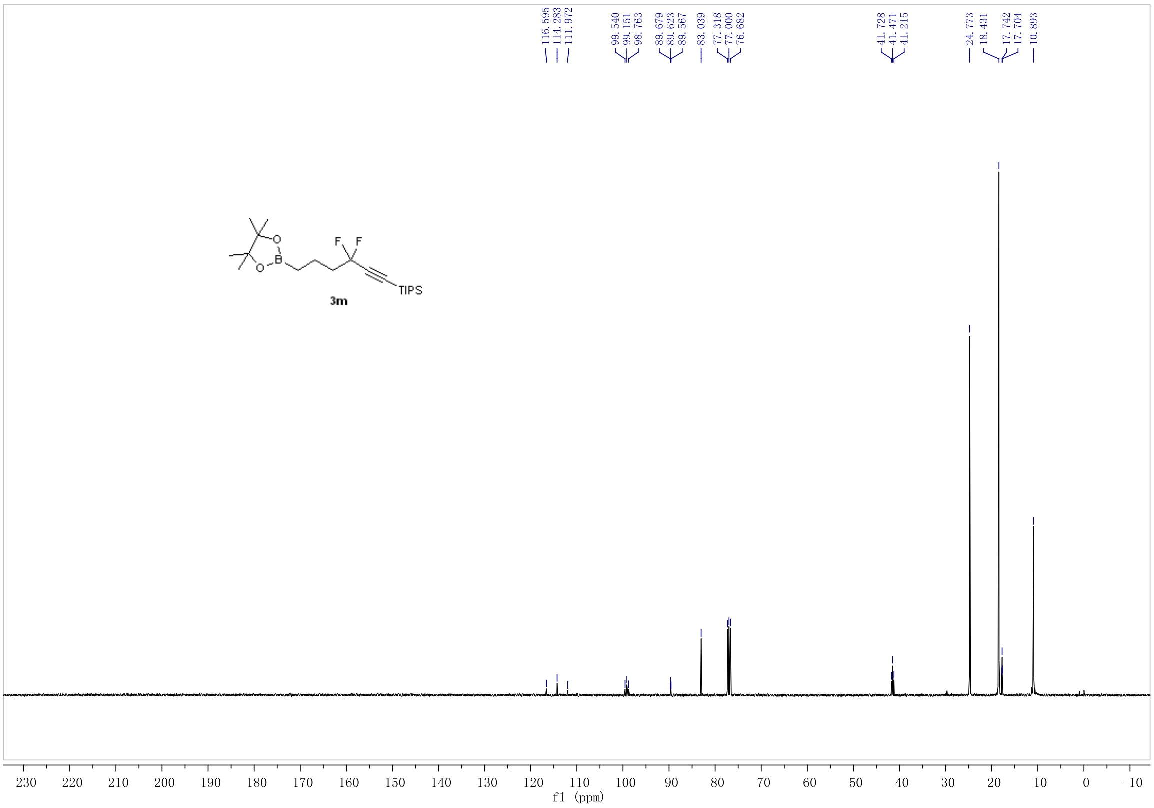

Supplementary Figure 73. ^{13}C NMR of (3,3-difluoro-6-(4,4,5,5-tetramethyl-1,3,2-dioxaborolan-2-yl)hex-1-yn-1-yl)triisopropylsilane (3m)

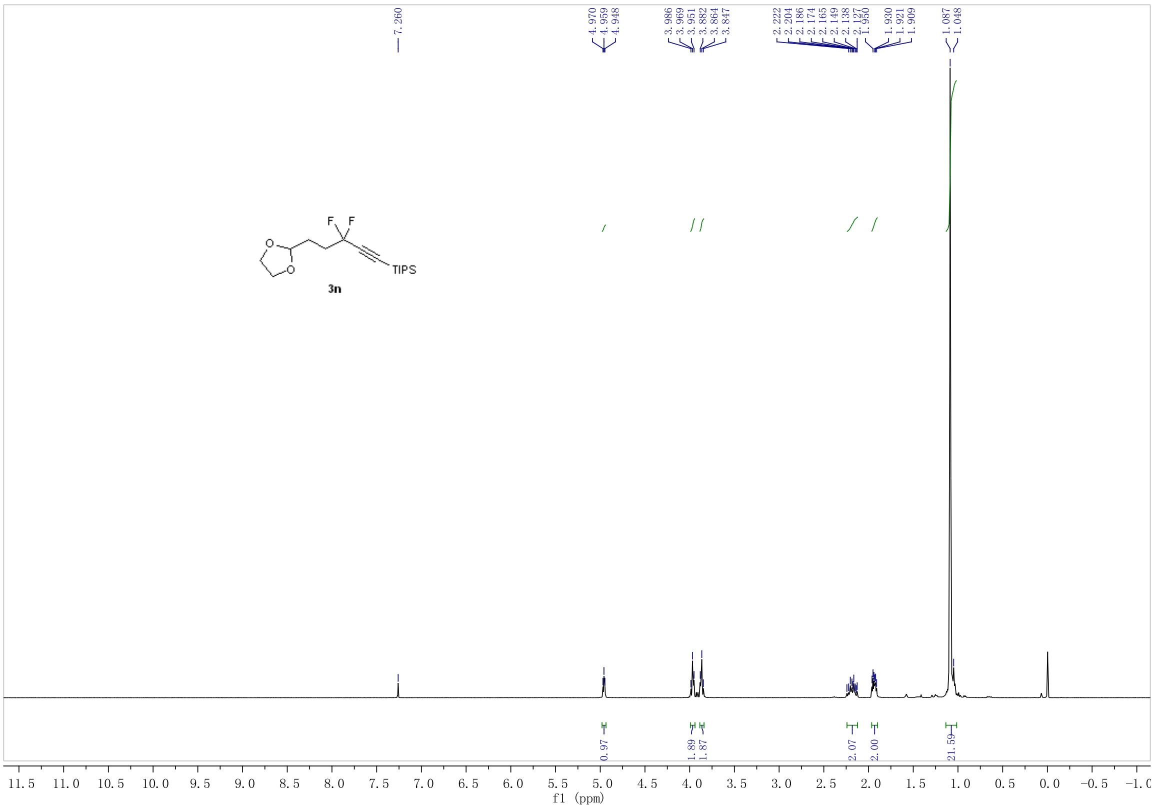

Supplementary Figure 74. ^{1}H NMR of (5-(1, 3-dioxolan-2-yl)-3,3-difluoropent-1-yn-1-yl)triisopropylsilane (3n)

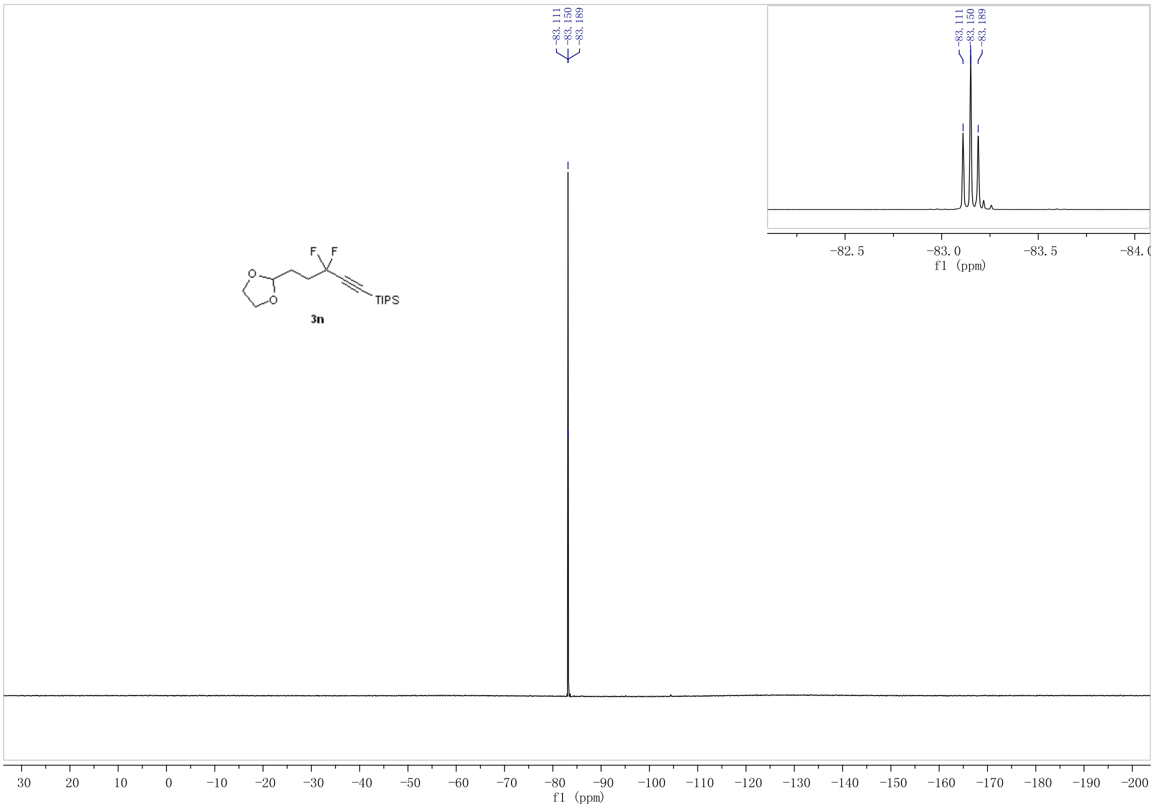

Supplementary Figure 75. **^{19}F NMR of (5-(1, 3-dioxolan-2-yl)-3,3-difluoropent-1-yn-1-yl)triisopropylsilane (3n)**

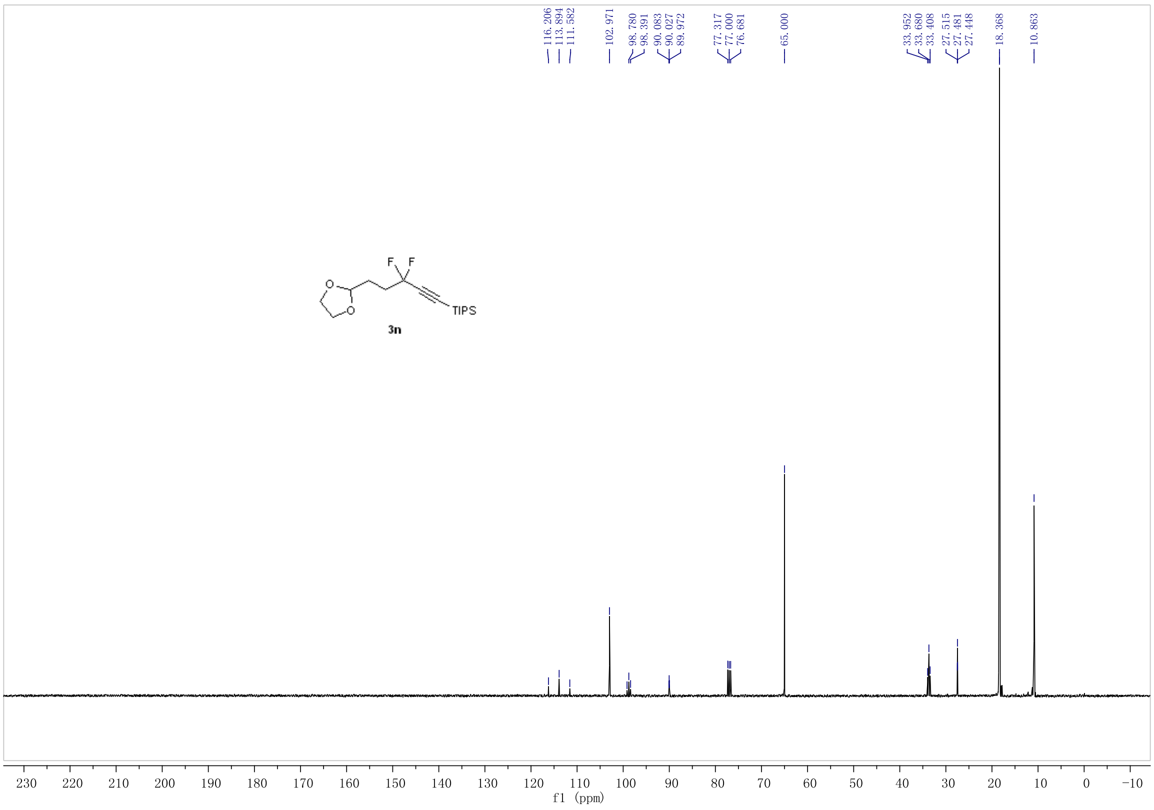

Supplementary Figure 76. **^{13}C NMR of (5-(1, 3-dioxolan-2-yl)-3,3-difluoropent-1-yn-1-yl)triisopropylsilane (3n)**

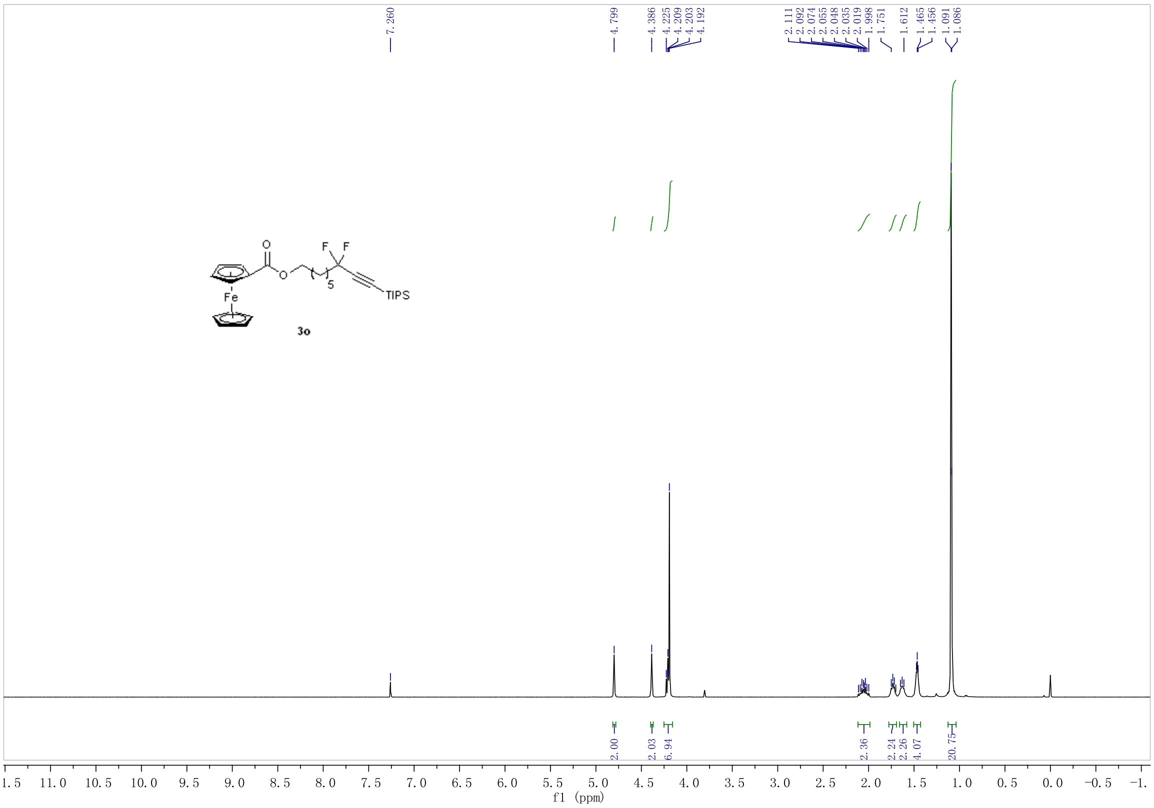

Supplementary Figure 77. ^1^H NMR of 7,7-difluoro-9-(triisopropylsilyl)non-8-yn-1-yl ferrocene formate (3o)

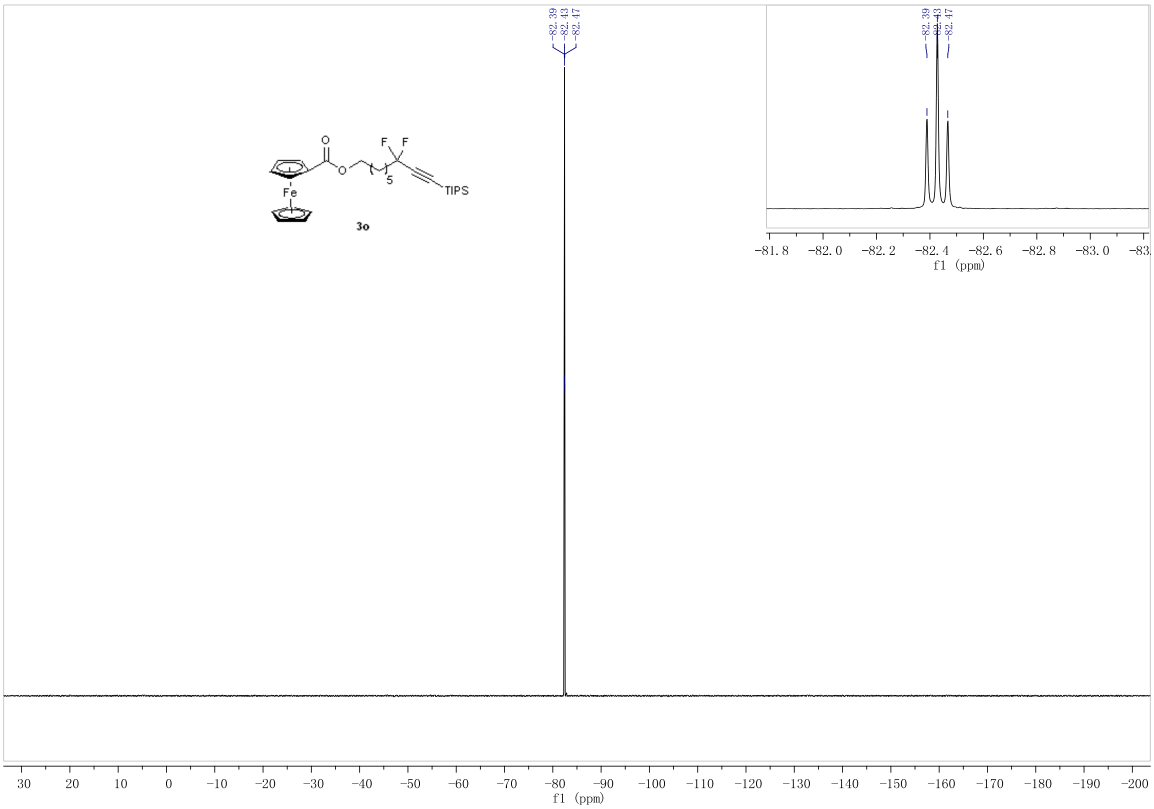

Supplementary Figure 78. ^19^F NMR of 7,7-difluoro-9-(triisopropylsilyl)non-8-yn-1-yl ferrocene formate (3o)

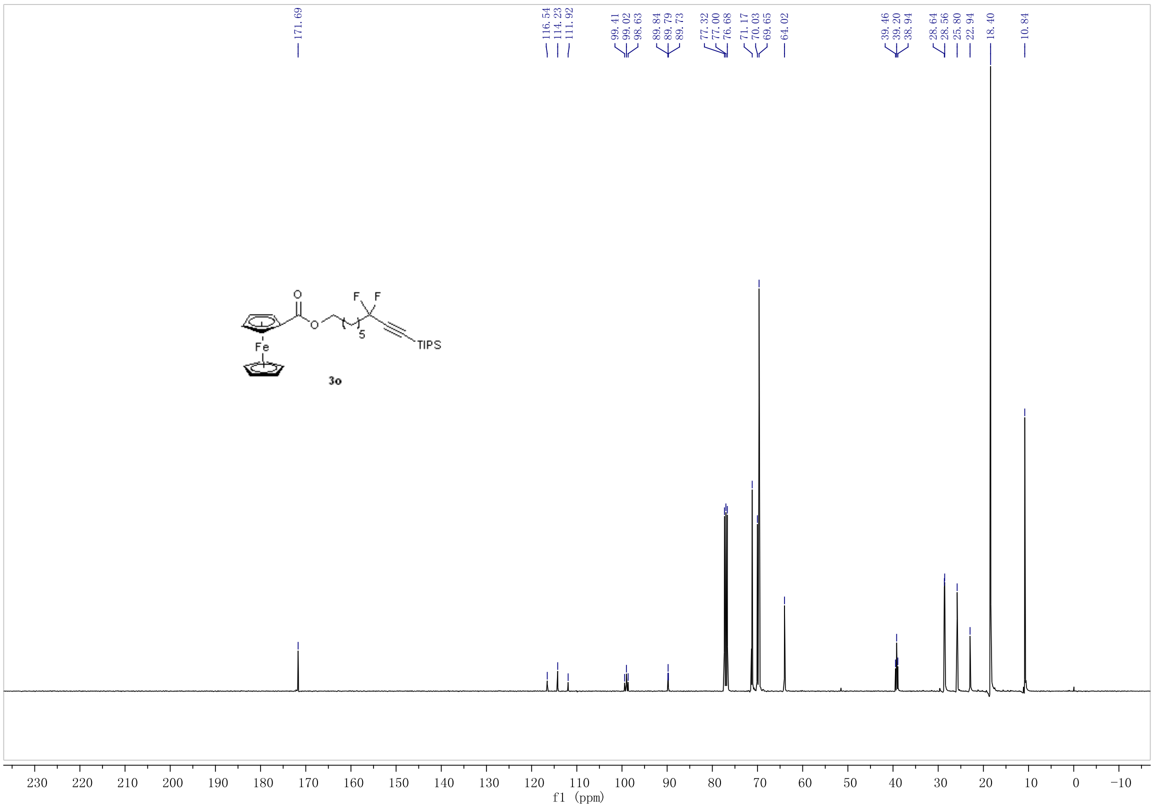

Supplementary Figure 79. ^{13}C NMR of 7,7-difluoro-9-(triisopropylsilyl)non-8-yn-1-yl ferrocene formate (3o)

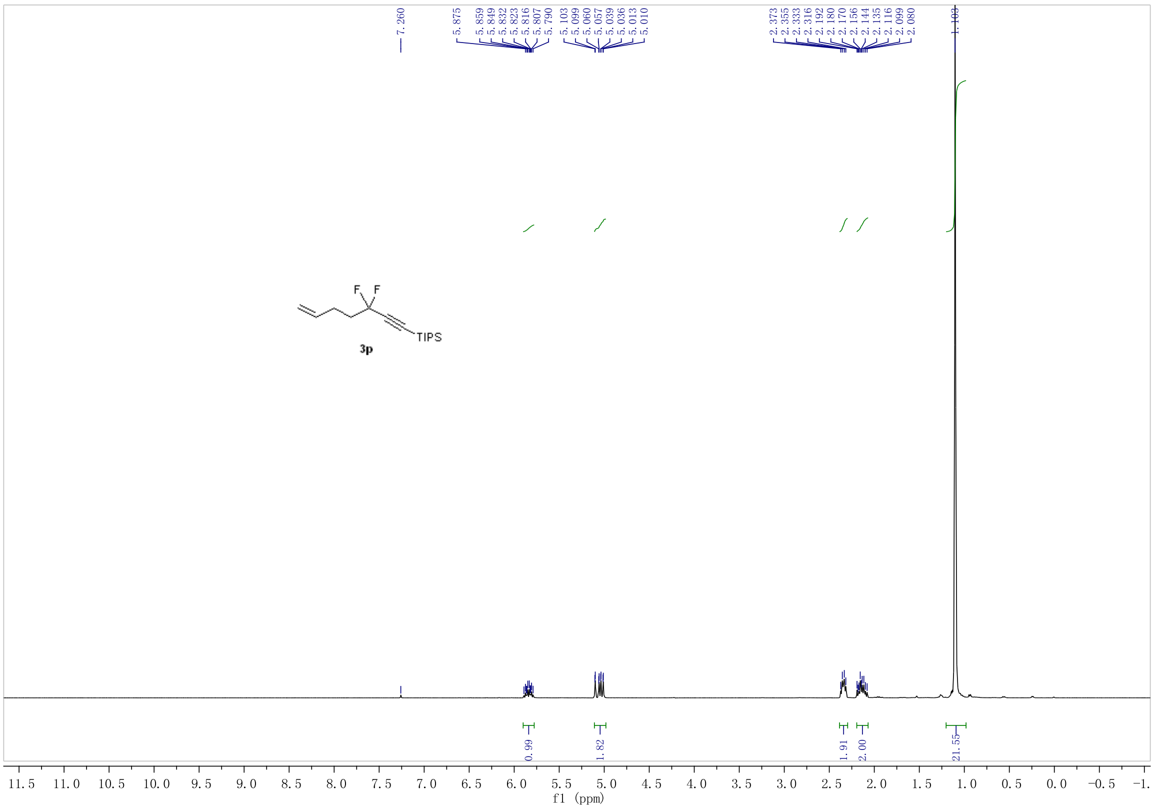

Supplementary Figure 80. ^{1}H NMR of (3,3-difluorohept-6-en-1-yn-1-yl)triisopropylsilane (3p)

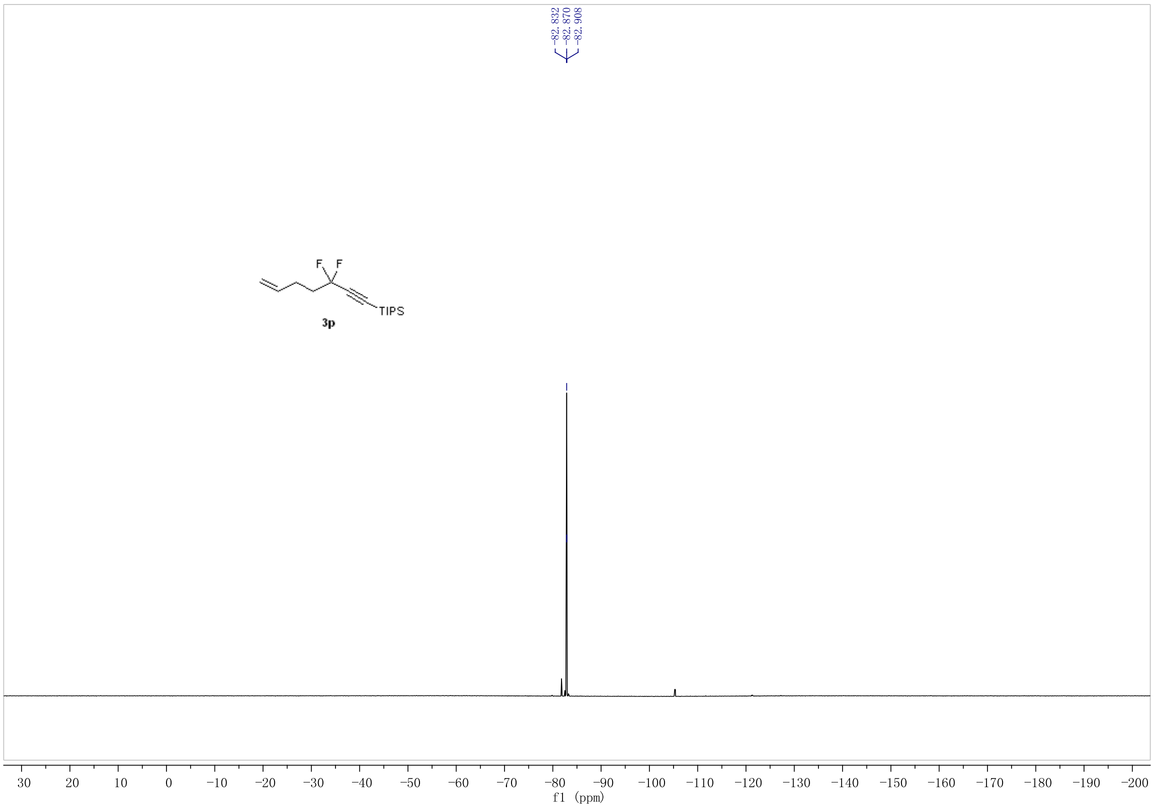

Supplementary Figure 81. ^{19}F NMR of (3,3-difluorohept-6-en-1-yn-1-yl)triisopropylsilane (3p)

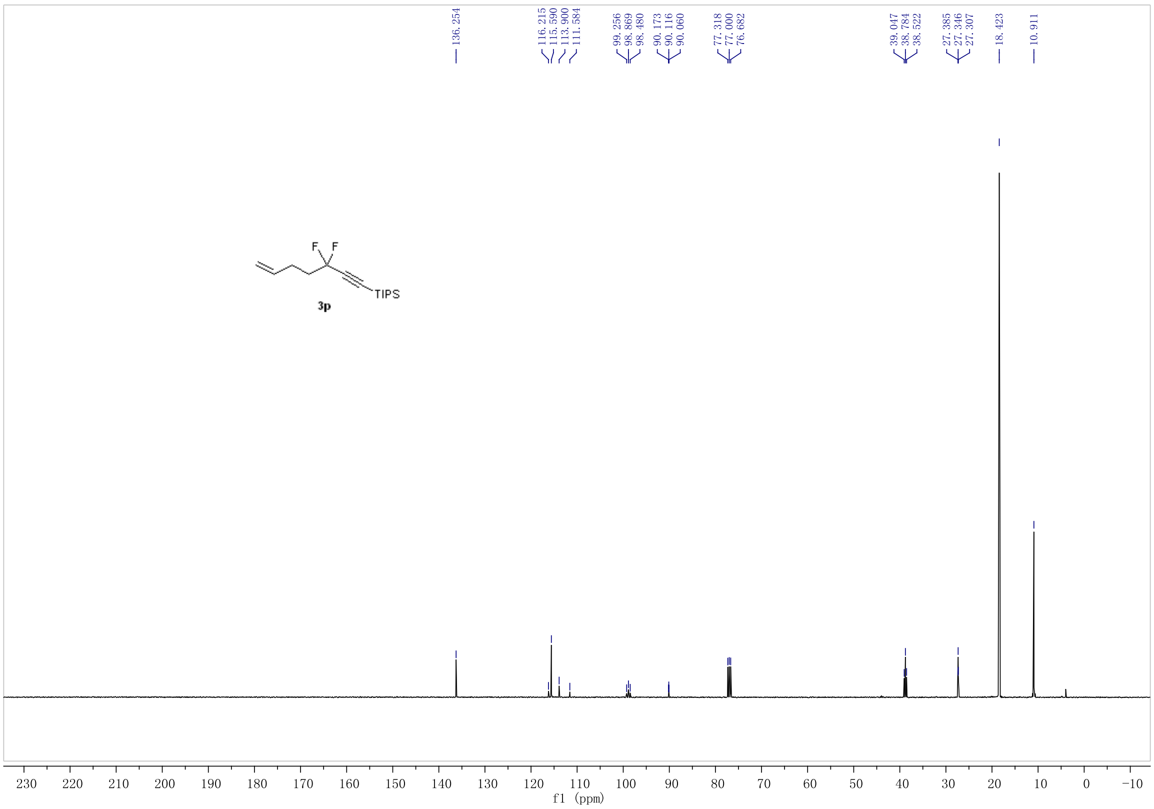

Supplementary Figure 82. ^{13}C NMR of (3,3-difluorohept-6-en-1-yn-1-yl)triisopropylsilane (3p)

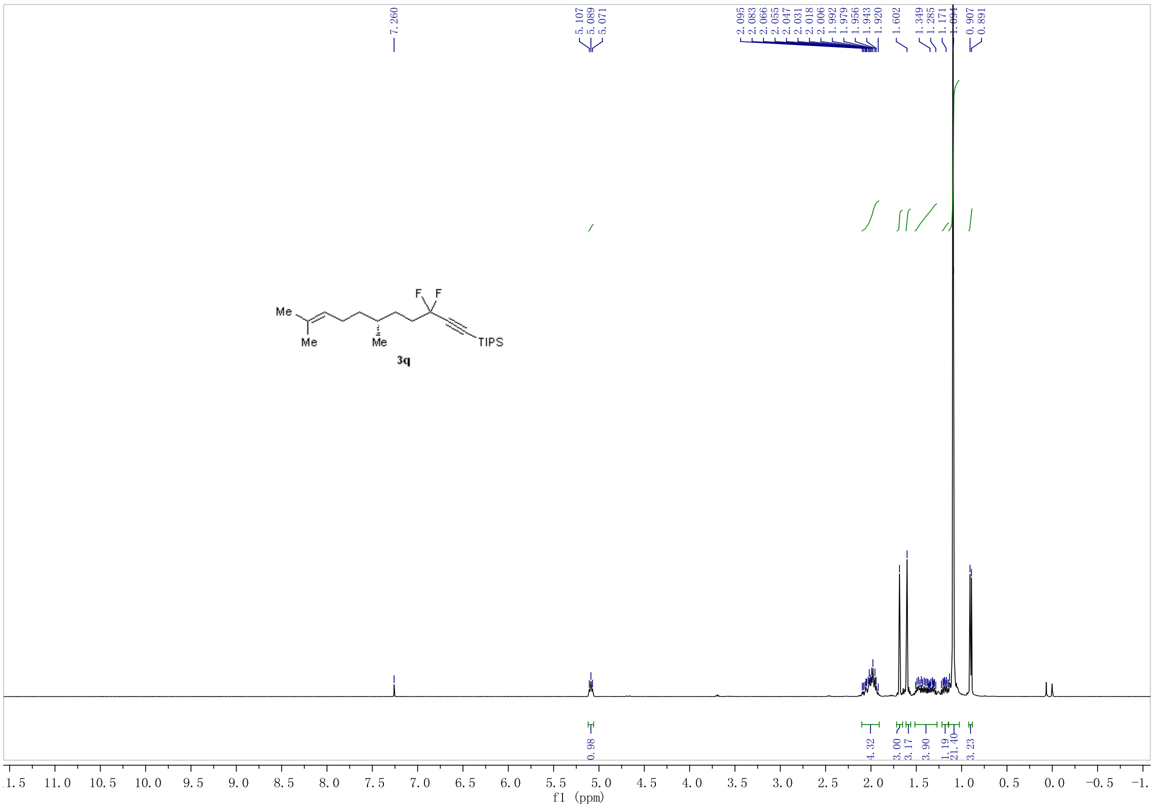

Supplementary Figure 83. ^{1}**H NMR of (*R*)-(3,3-difluoro-6,10-dimethylundec-9-en-1-yn-1-yl)triisopropylsilane (3q)**

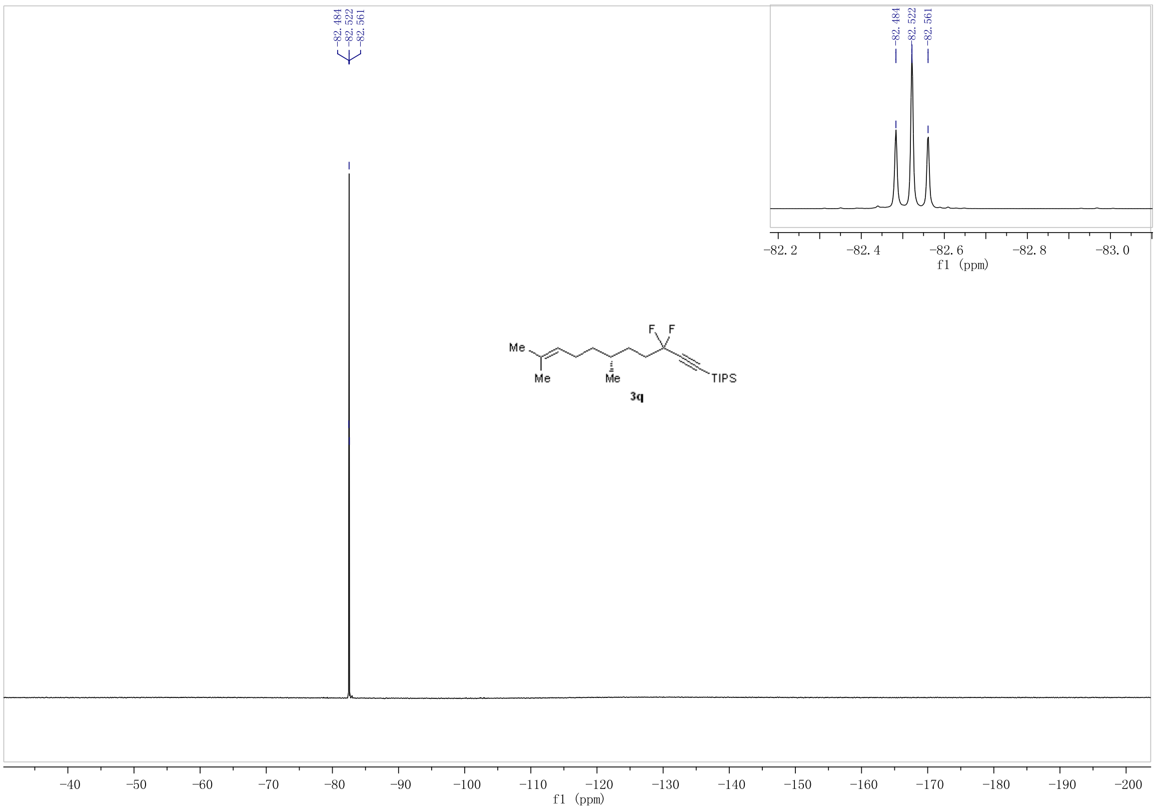

Supplementary Figure 84. ^{19}**F NMR of (*R*)-(3,3-difluoro-6,10-dimethylundec-9-en-1-yn-1-yl)triisopropylsilane (3q)**

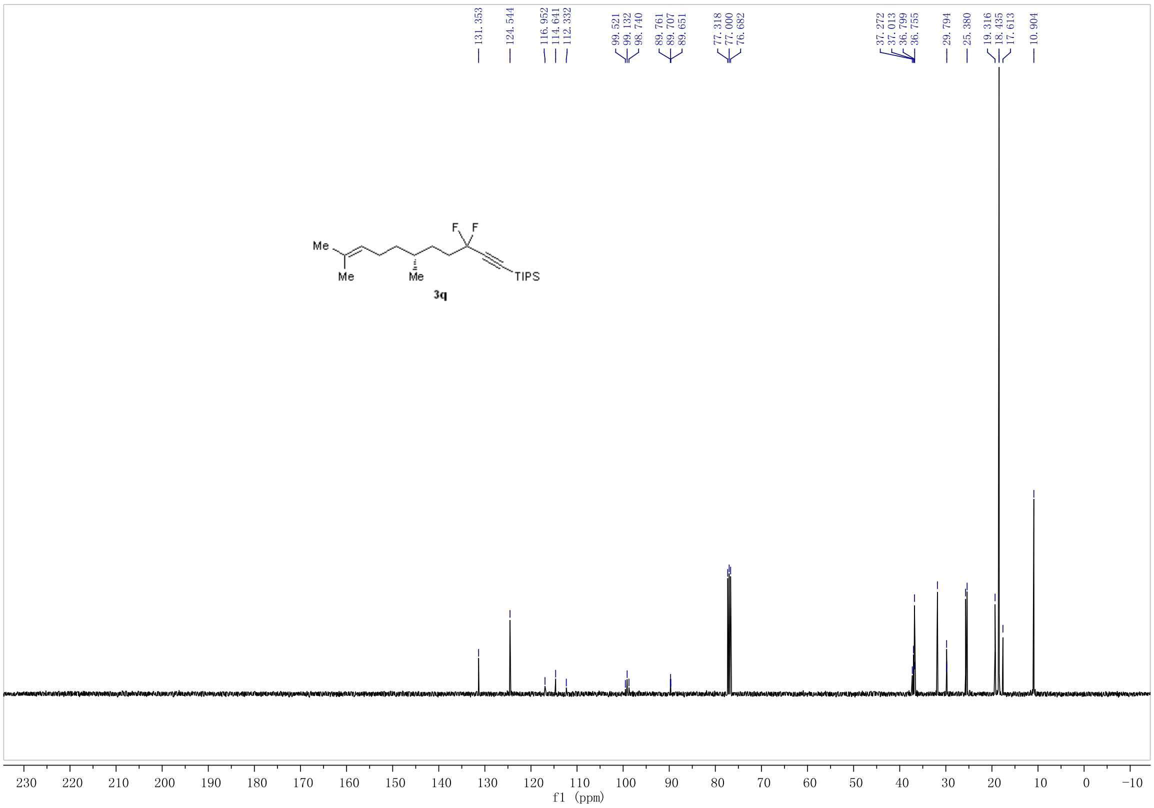

Supplementary Figure 85. ^{13}**C NMR of (*R*)-(3,3-difluoro-6,10-dimethylundec-9-en-1-yn-1-yl)triisopropylsilane (3q)**

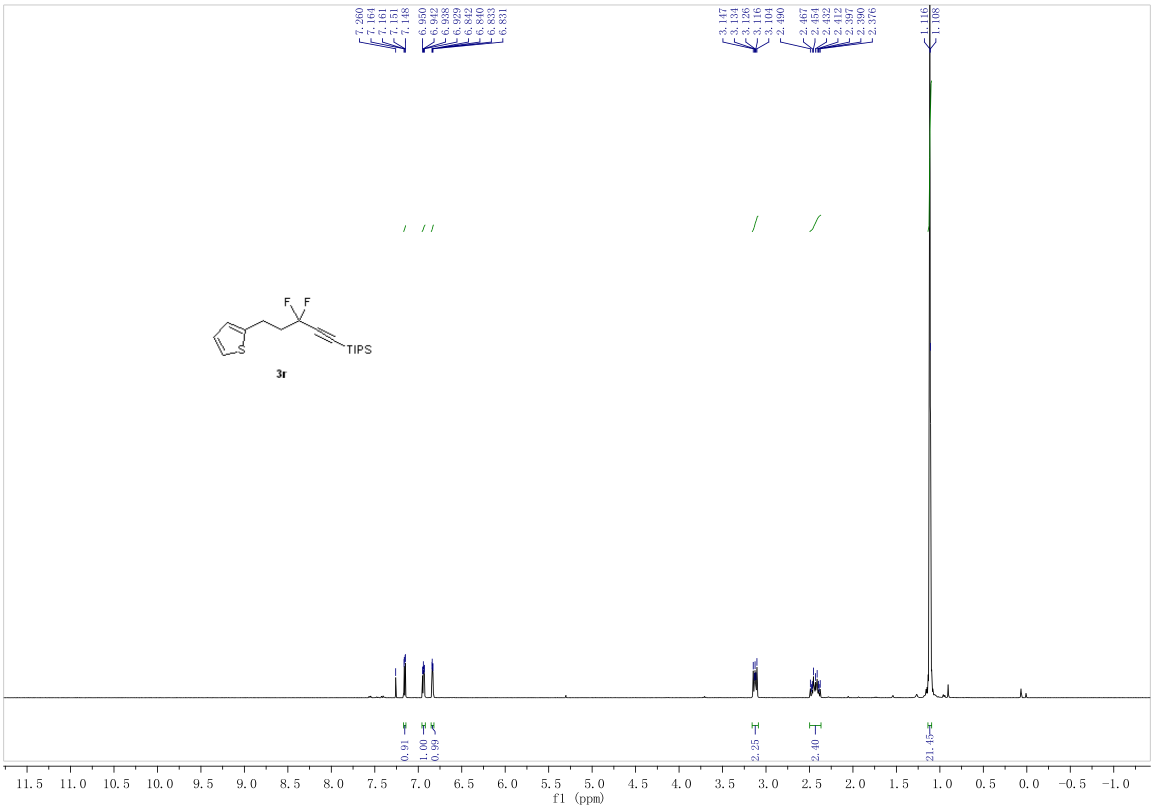

Supplementary Figure 86. ^{1}**H NMR of (3,3-difluoro-5-(thiophen-2-yl)pent-1-yn-1-yl)triisopropylsilane (3r)**

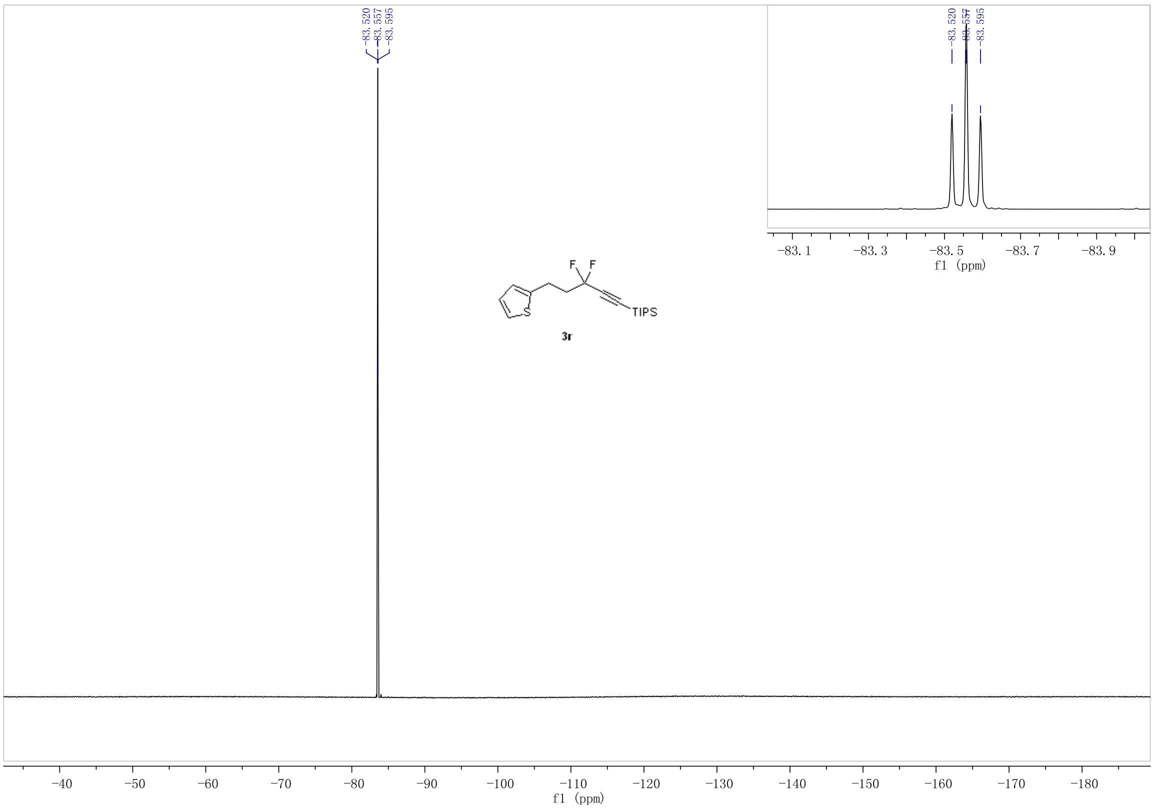

Supplementary Figure 87. ^{19}F NMR of (3,3-difluoro-5-(thiophen-2-yl)pent-1-yn-1-yl)triisopropylsilane (3r)

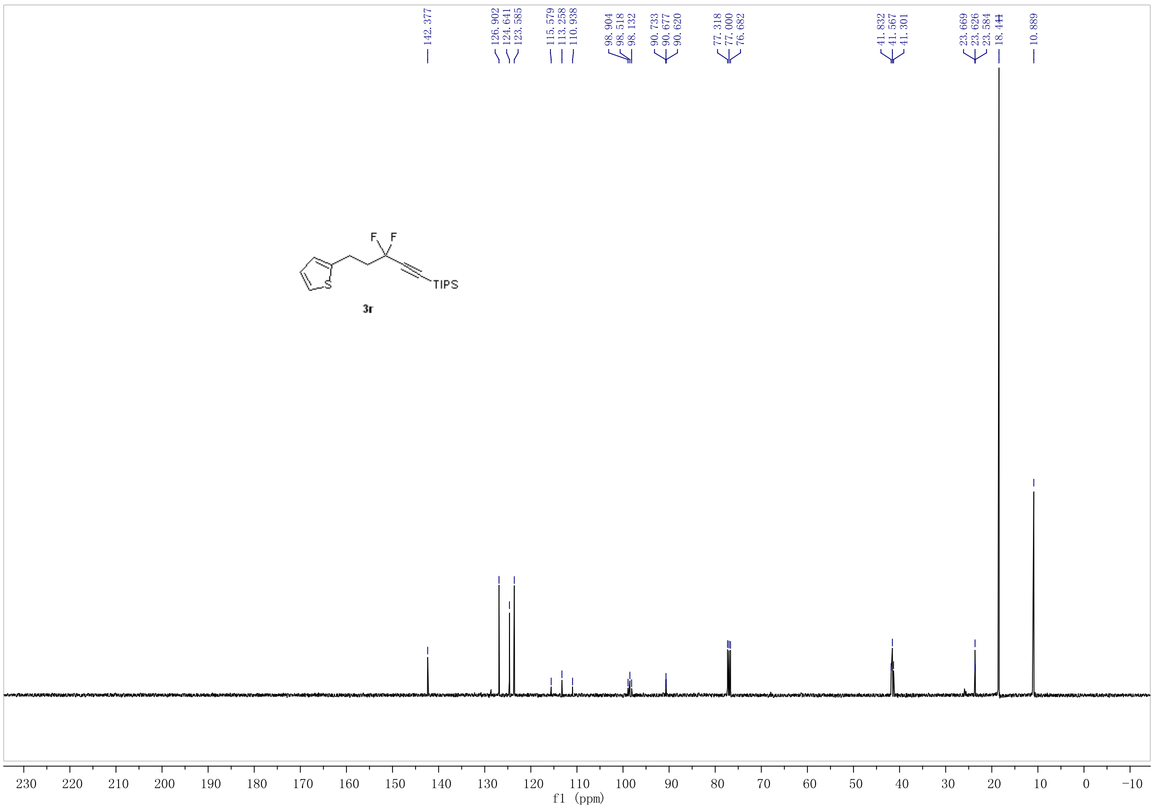

Supplementary Figure 88. ^{13}C NMR of (3,3-difluoro-5-(thiophen-2-yl)pent-1-yn-1-yl)triisopropylsilane (3r)

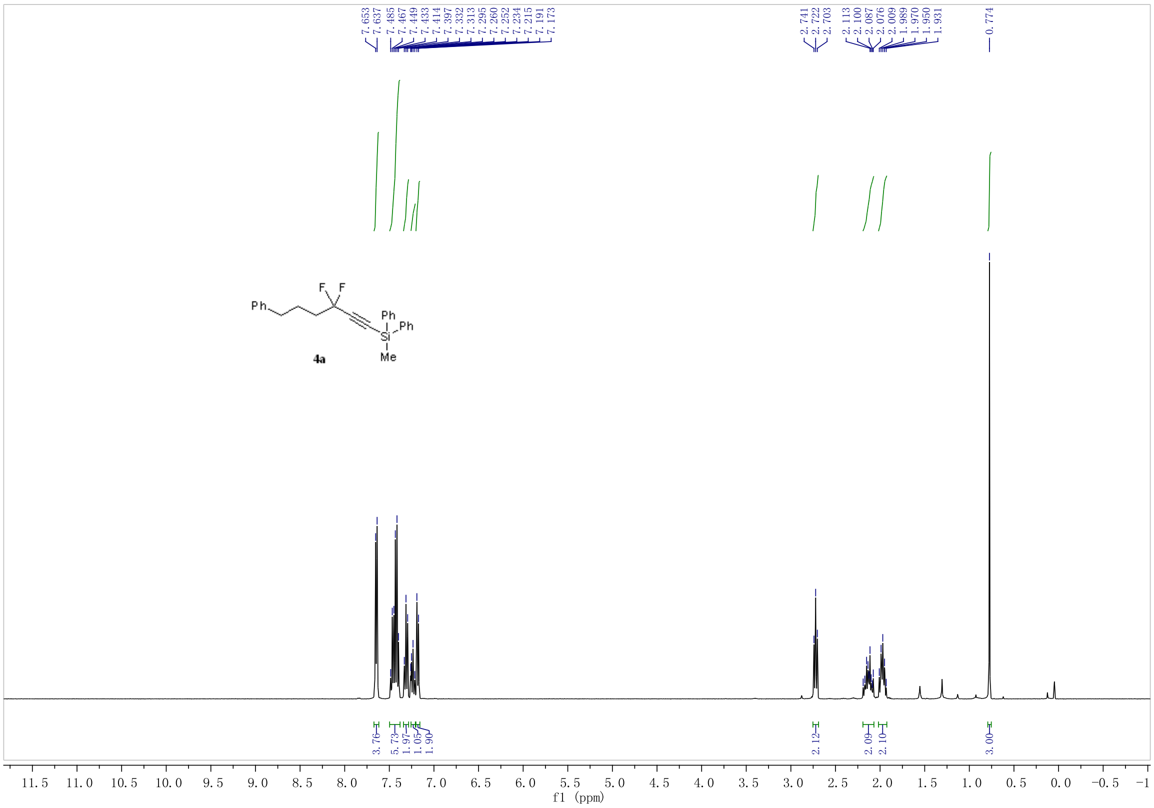

Supplementary Figure 89. ^{1}H NMR of (3,3-difluoro-6-phenylhex-1-yn-1-yl)(methyl)diphenylsilane (4a)

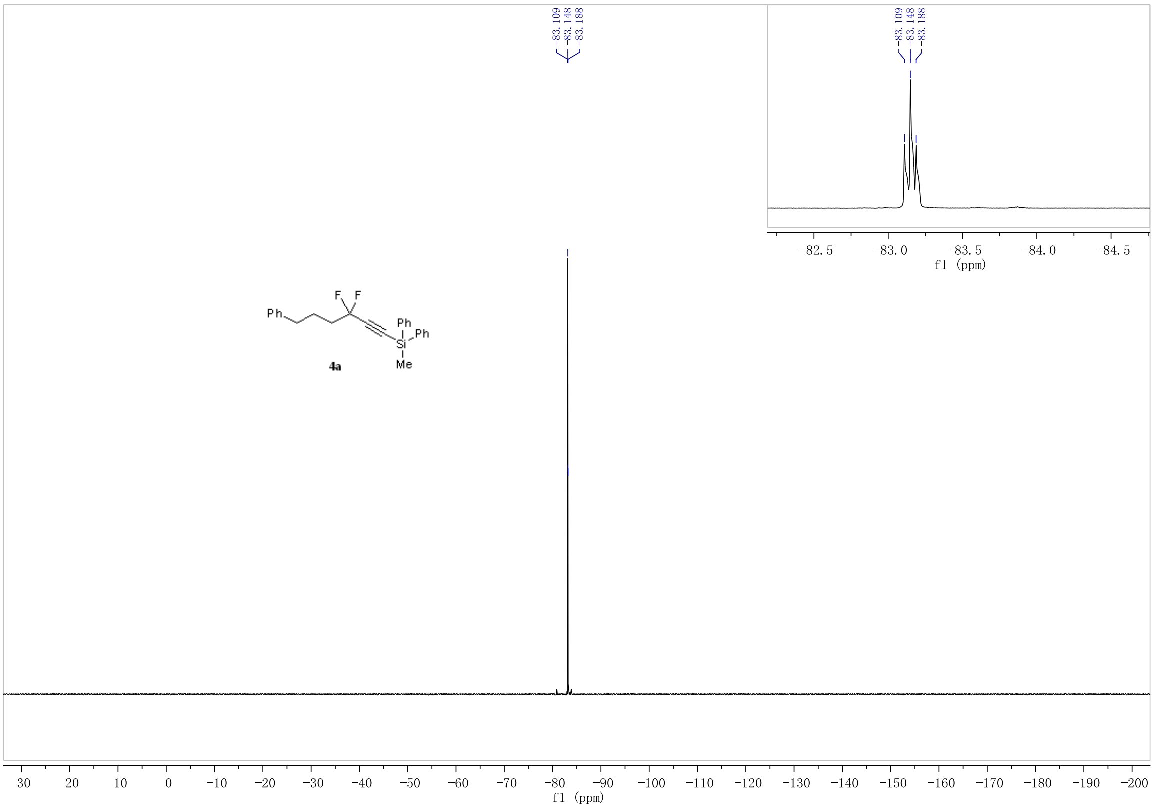

Supplementary Figure 90. ^{19}F NMR of (3,3-difluoro-6-phenylhex-1-yn-1-yl)(methyl)diphenylsilane (4a)

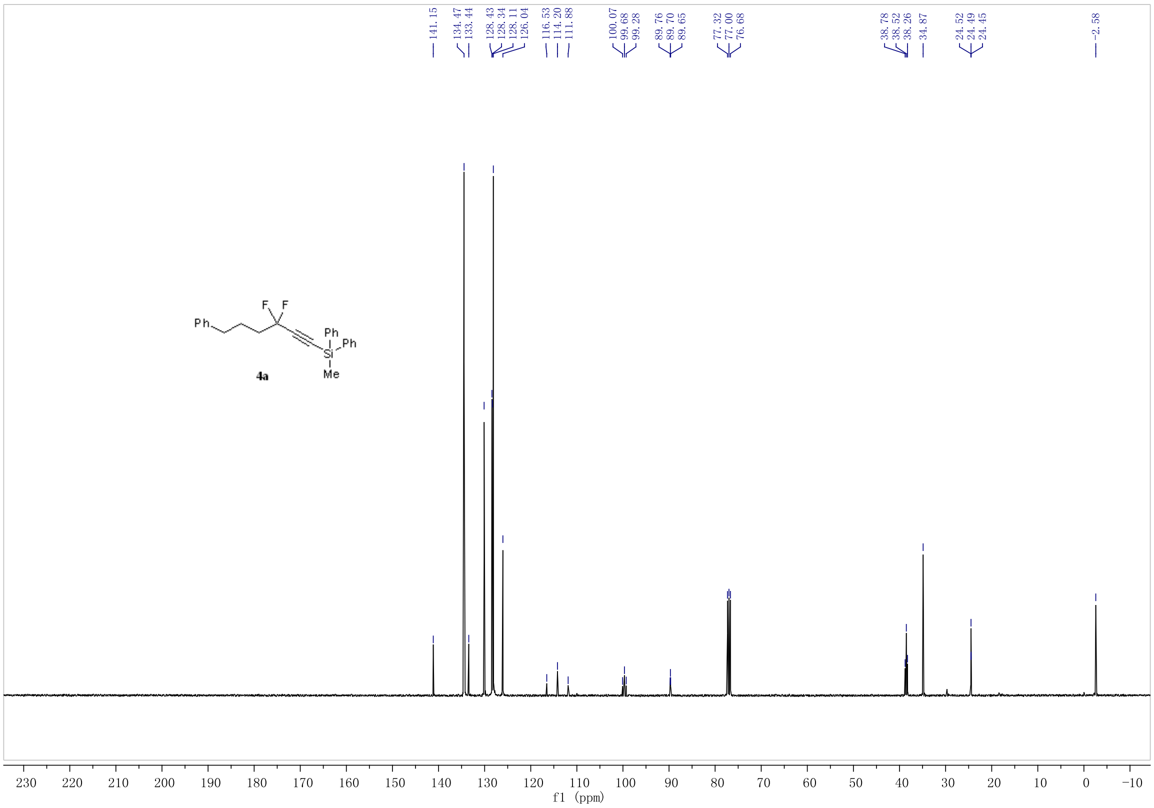

Supplementary Figure 91. ^{13}**C NMR of (3,3-difluoro-6-phenylhex-1-yn-1-yl)(methyl)diphenylsilane (4a)**

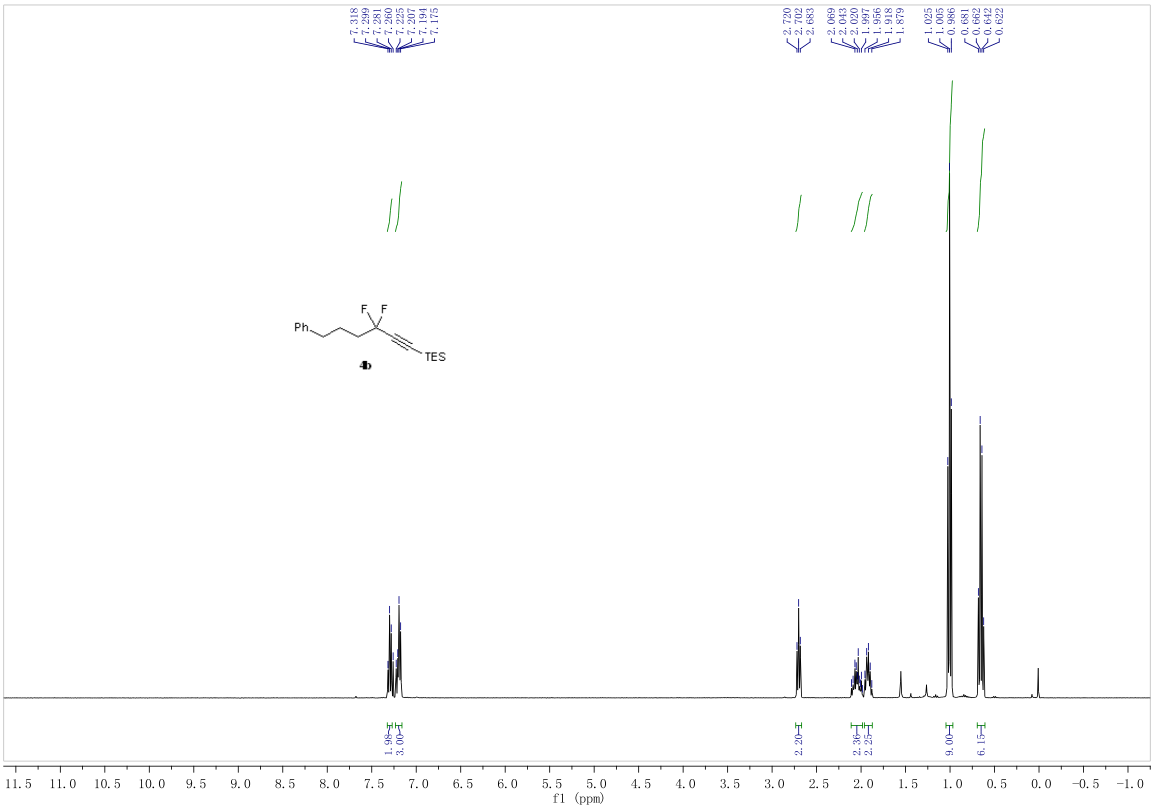

Supplementary Figure 92. ^{1}**H NMR of (3,3-difluoro-6-phenylhex-1-yn-1-yl)triethylsilane (4b)**

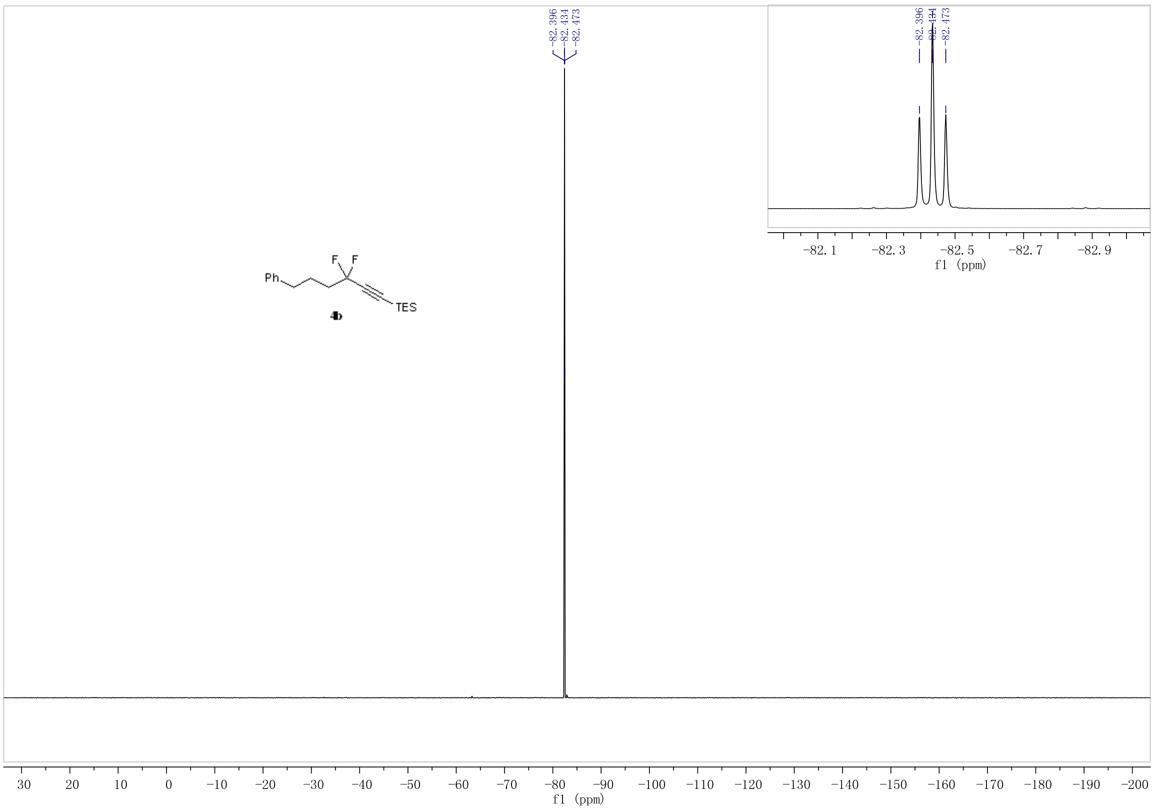

Supplementary Figure 93. ^{19}F NMR of (3,3-difluoro-6-phenylhex-1-yn-1-yl)triethylsilane (4b)

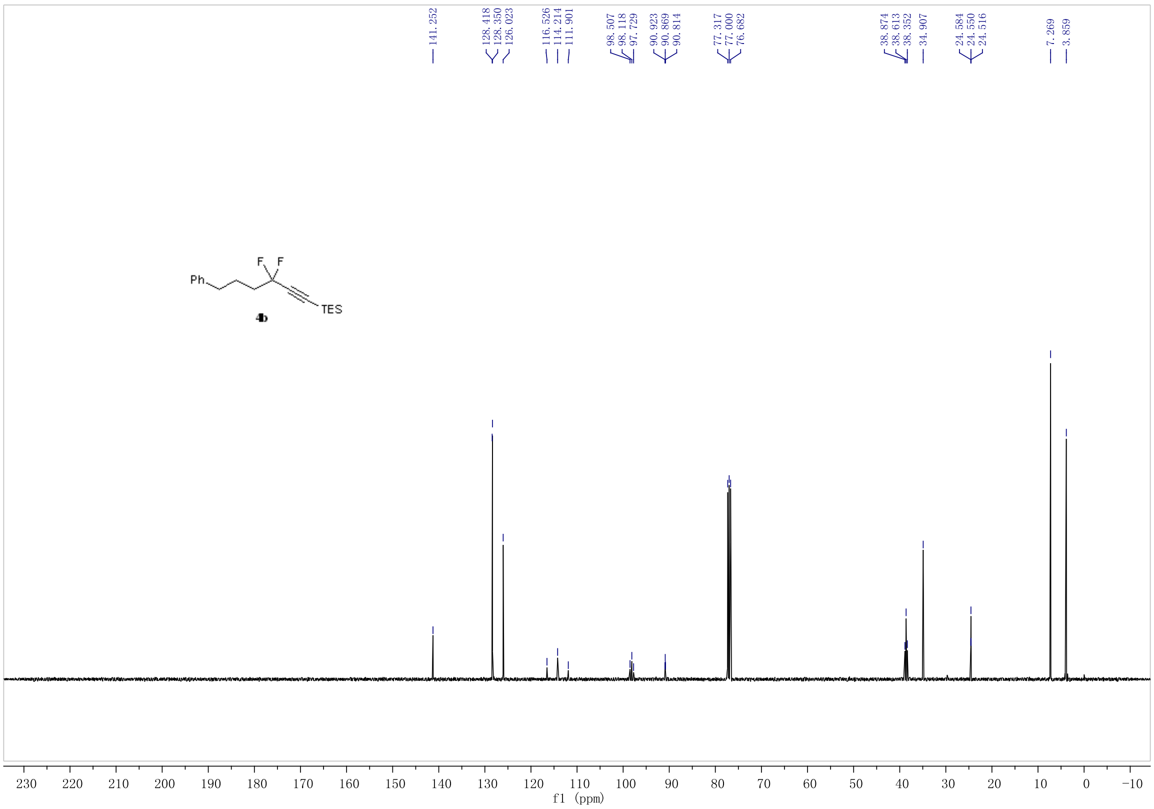

Supplementary Figure 94. ^{13}C NMR of (3,3-difluoro-6-phenylhex-1-yn-1-yl)triethylsilane (4b)

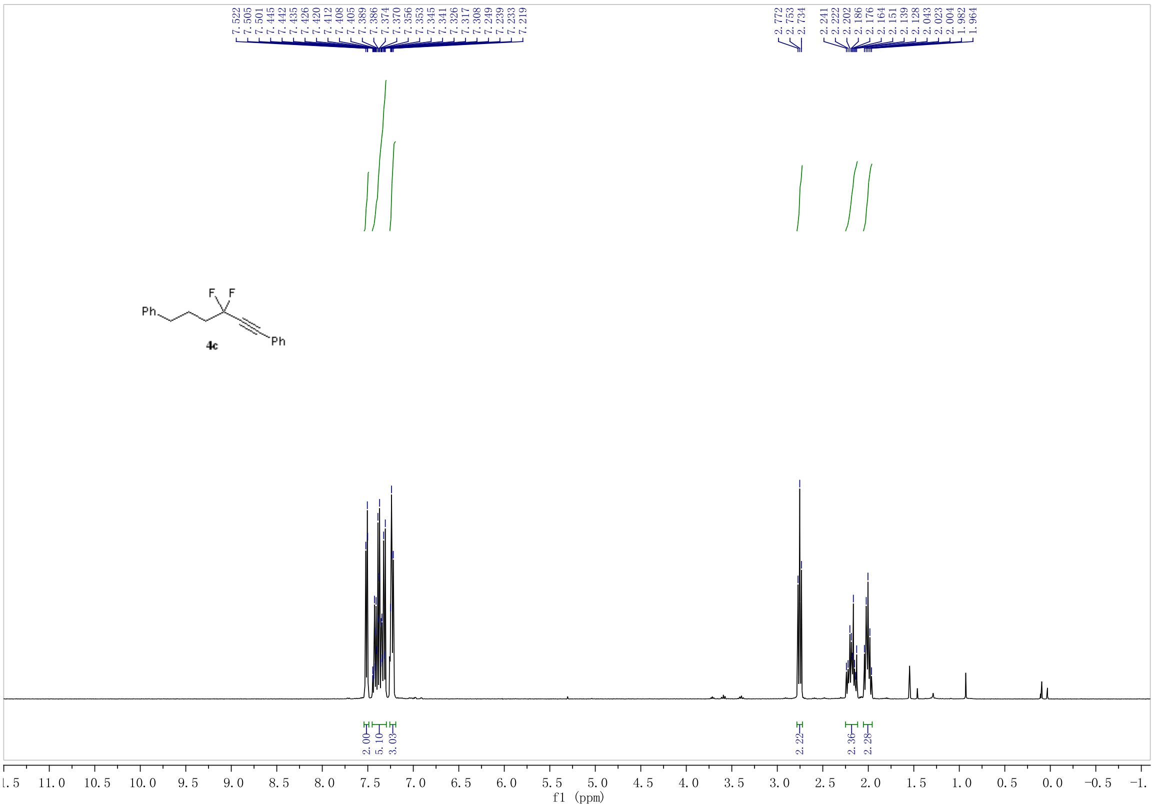

Supplementary Figure 95. ^{1}H NMR of (3,3-difluorohex-1-yne-1,6-diyl)dibenzene (4c)

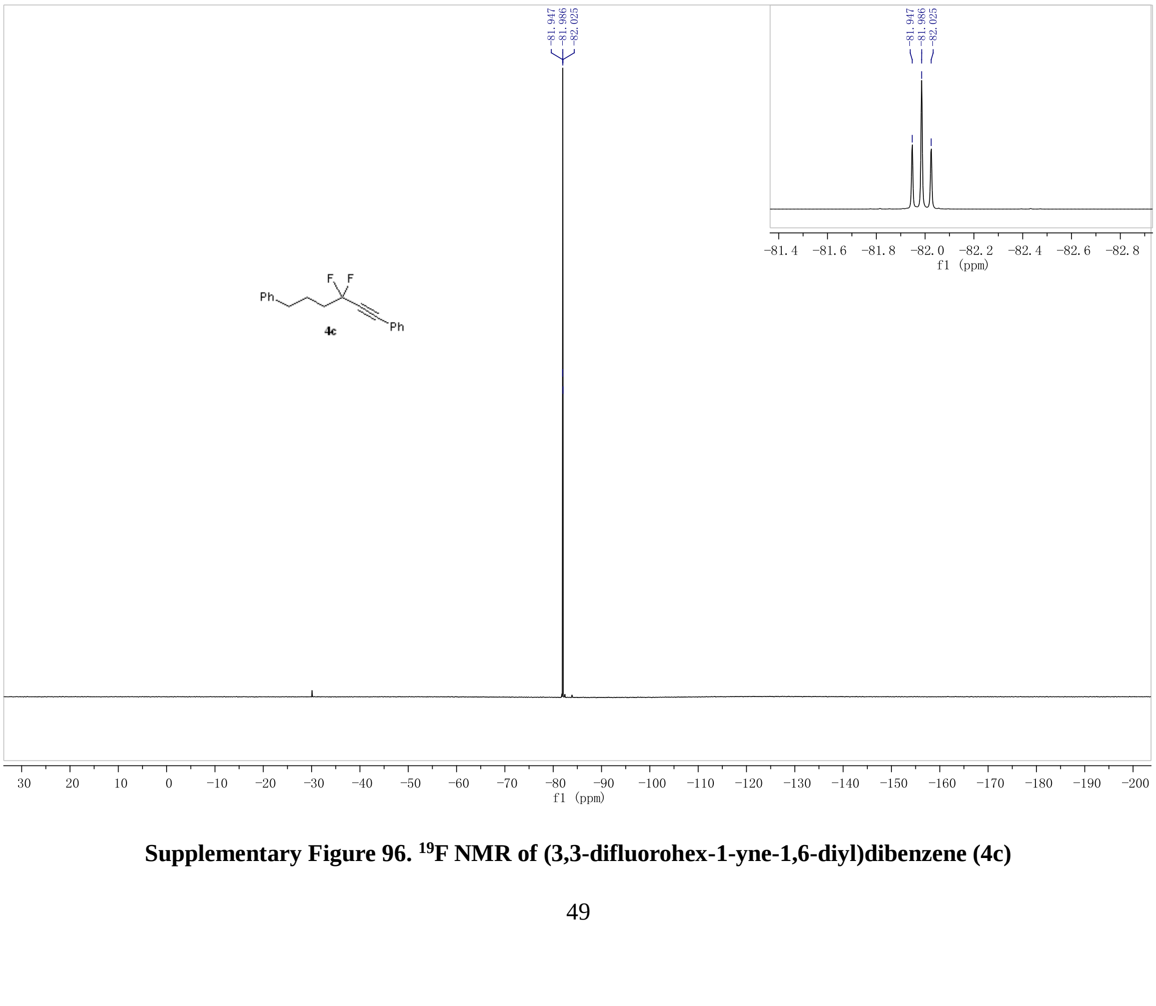

Supplementary Figure 96. ^{19}F NMR of (3,3-difluorohex-1-yne-1,6-diyl)dibenzene (4c)

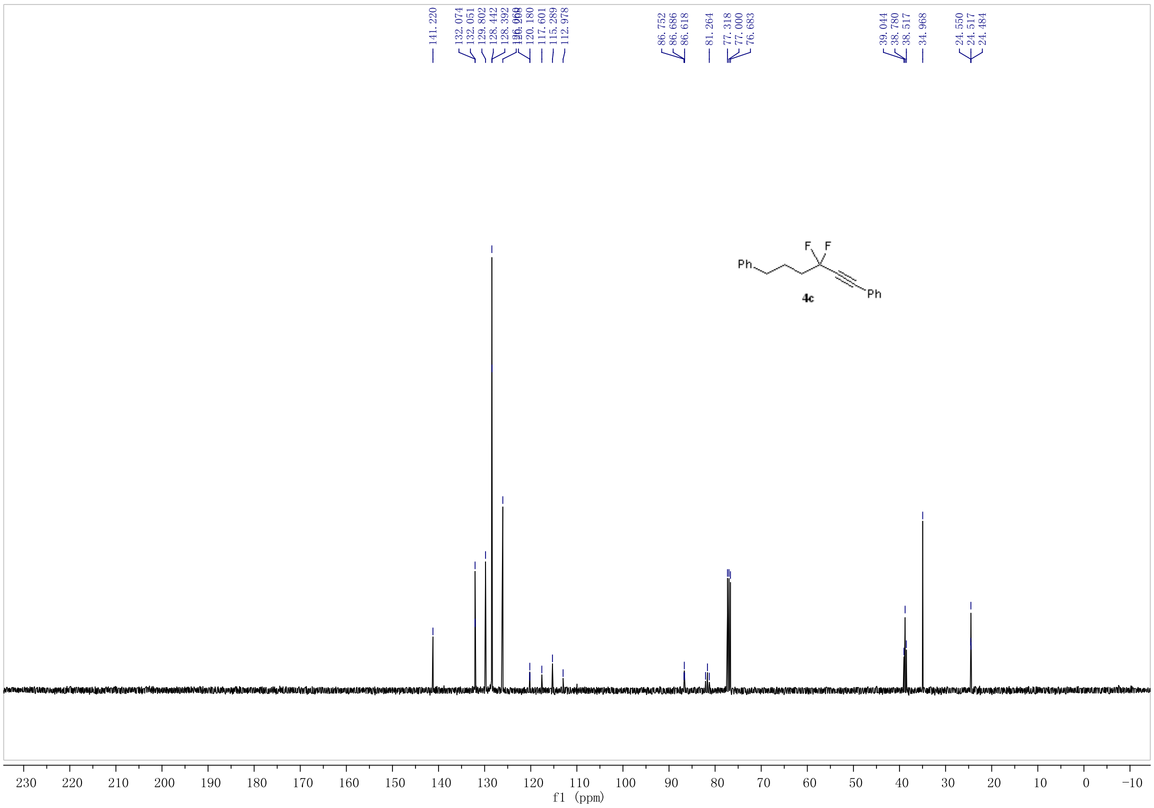

Supplementary Figure 97. ^{13}C NMR of (3,3-difluorohex-1-yne-1,6-diyl)dibenzene (4c)

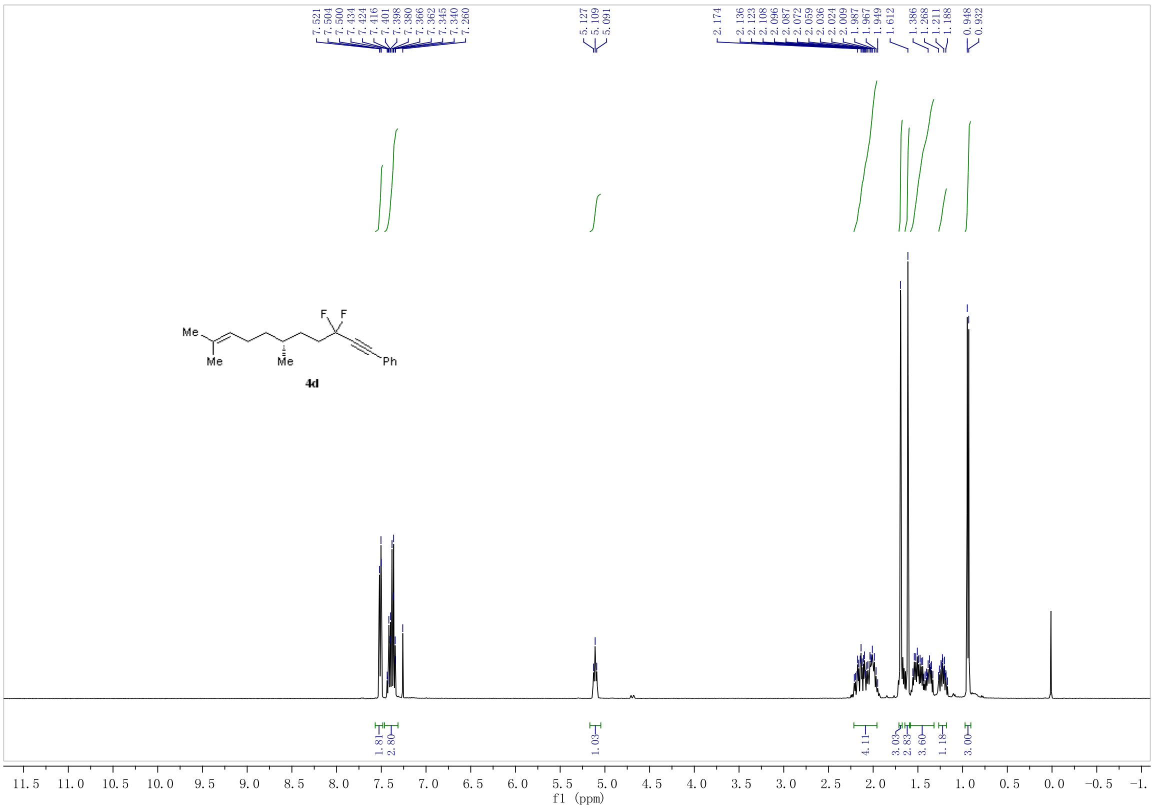

Supplementary Figure 98. ^{1}H NMR of (*R*)-(3,3-difluoro-6,10-dimethylundec-9-en-1-yn-1-yl)benzene (4d)

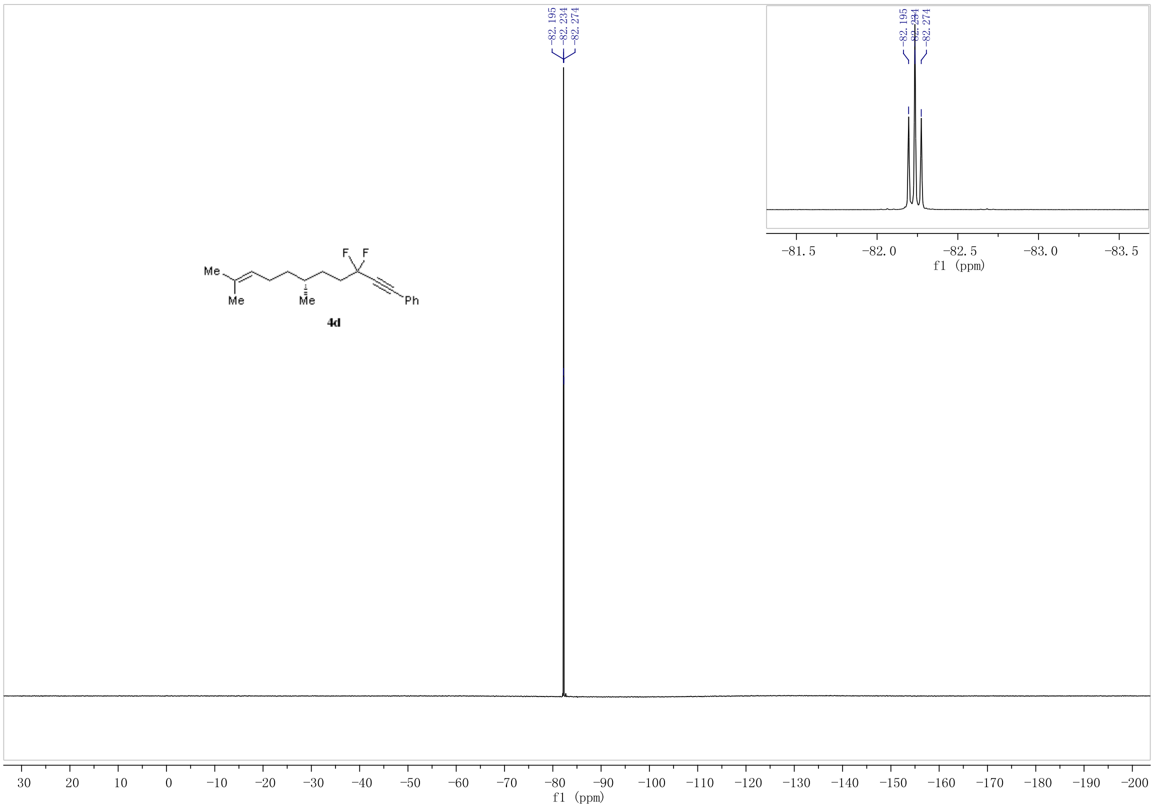

Supplementary Figure 99. ^{19}**F NMR of (*R*)-(3,3-difluoro-6,10-dimethylundec-9-en-1-yn-1-yl)benzene (4d)**

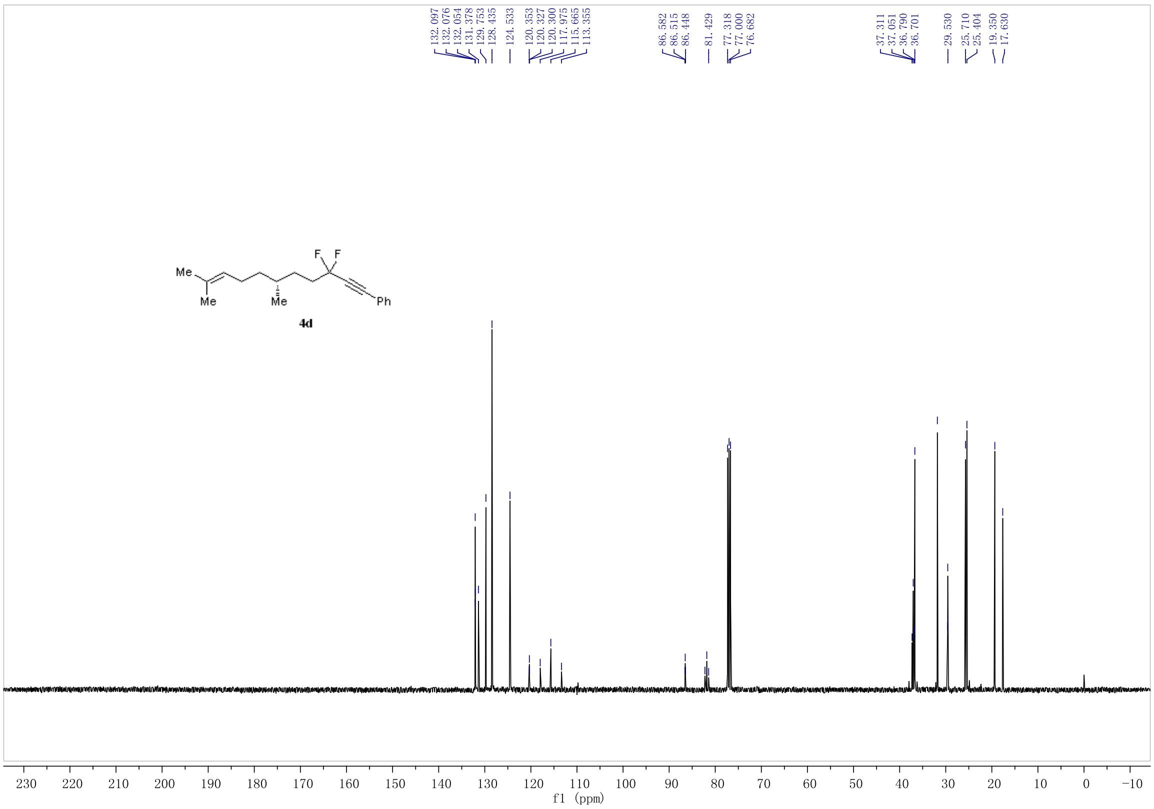

Supplementary Figure 100. ^{13}**C NMR of (*R*)-(3,3-difluoro-6,10-dimethylundec-9-en-1-yn-1-yl)benzene (4d)**

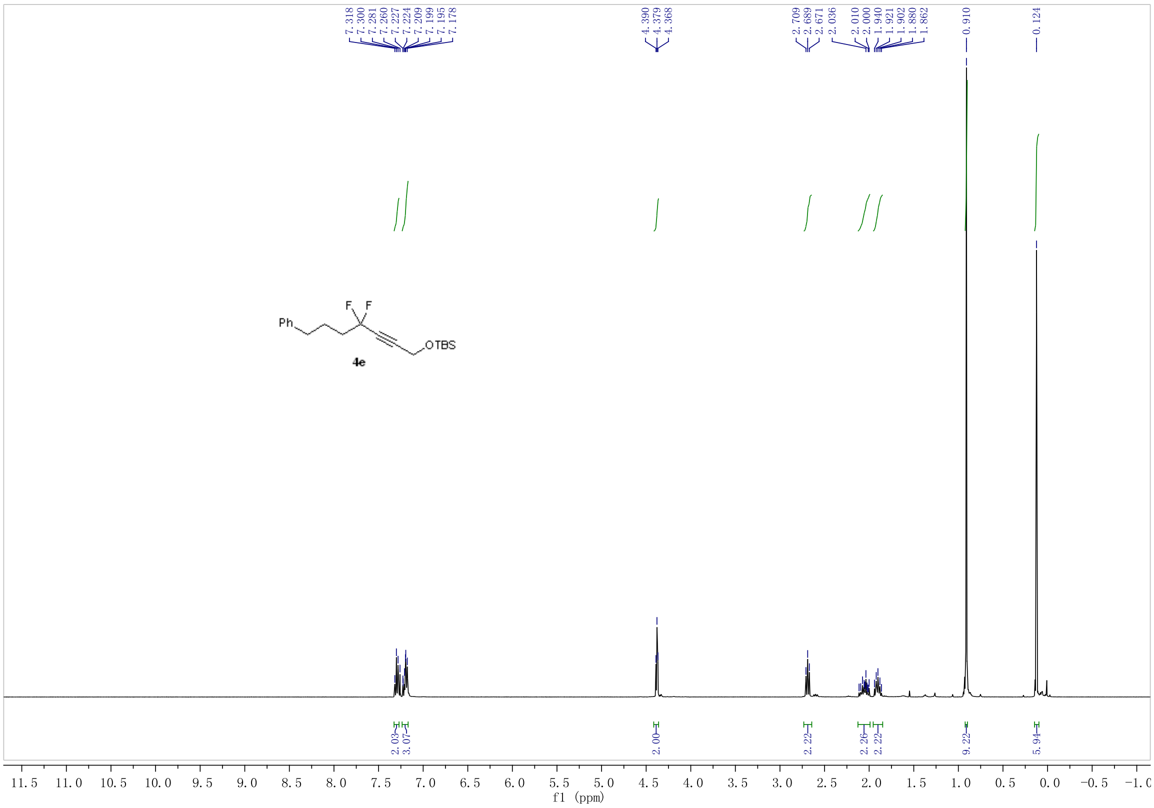

Supplementary Figure 101. ^{1}**H NMR of** ***tert*****-butyl((4,4-difluoro-7-phenylhept-2-yn-1-yl)oxy)dimethylsilane (4e)**

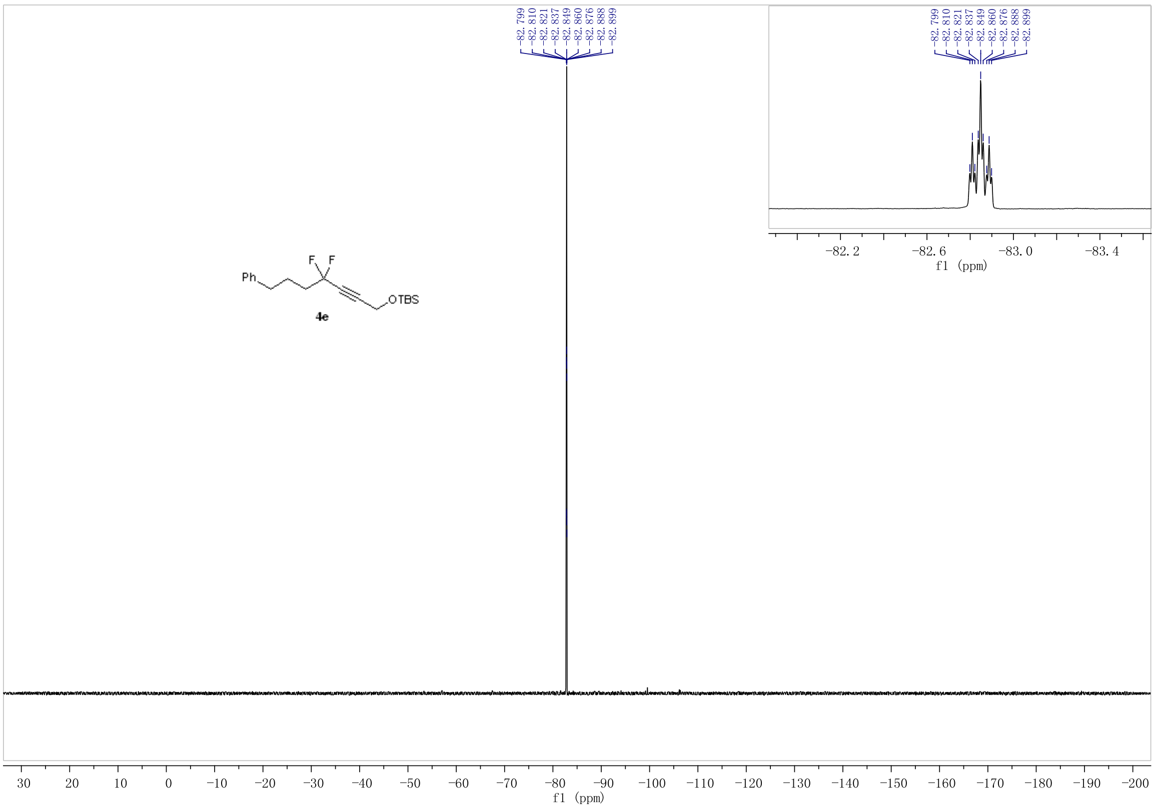

Supplementary Figure 102. ^{19}**F NMR of** ***tert*****-butyl((4,4-difluoro-7-phenylhept-2-yn-1-yl)oxy)dimethylsilane (4e)**

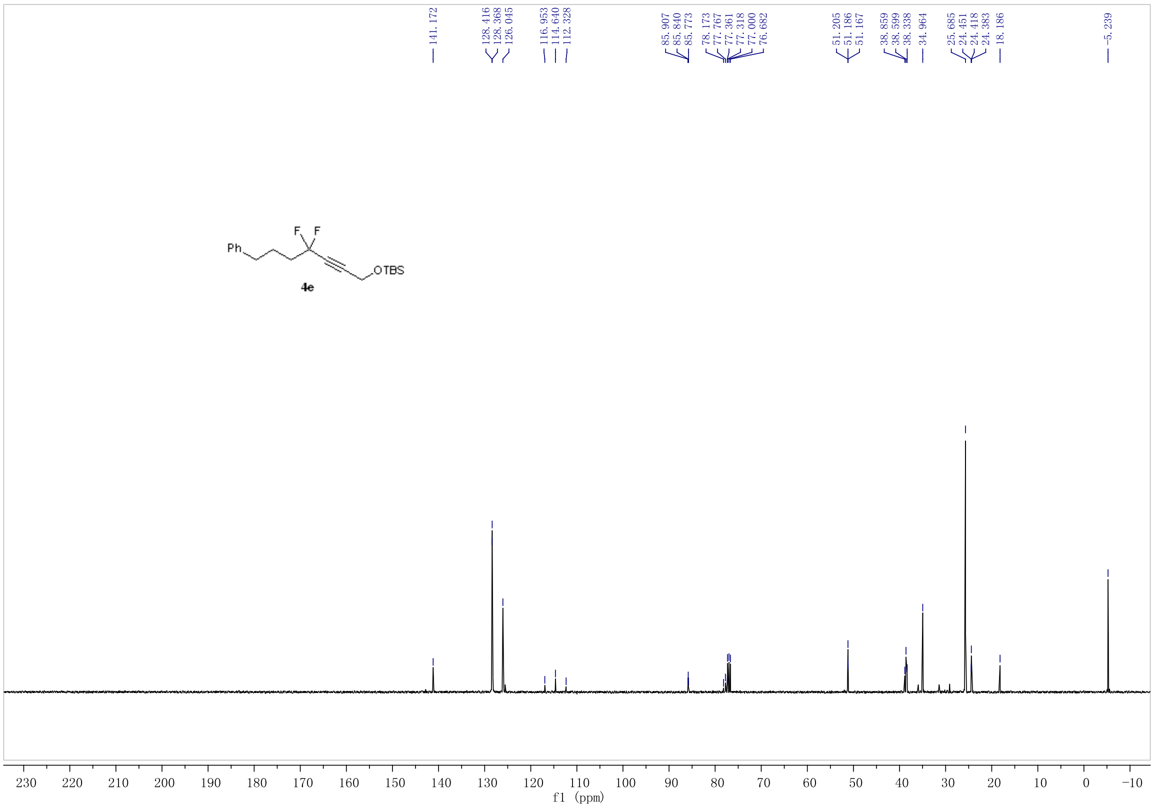

Supplementary Figure 103. **^{13}C NMR of *tert*-butyl((4,4-difluoro-7-phenylhept-2-yn-1-yl)oxy)dimethylsilane (4e)**

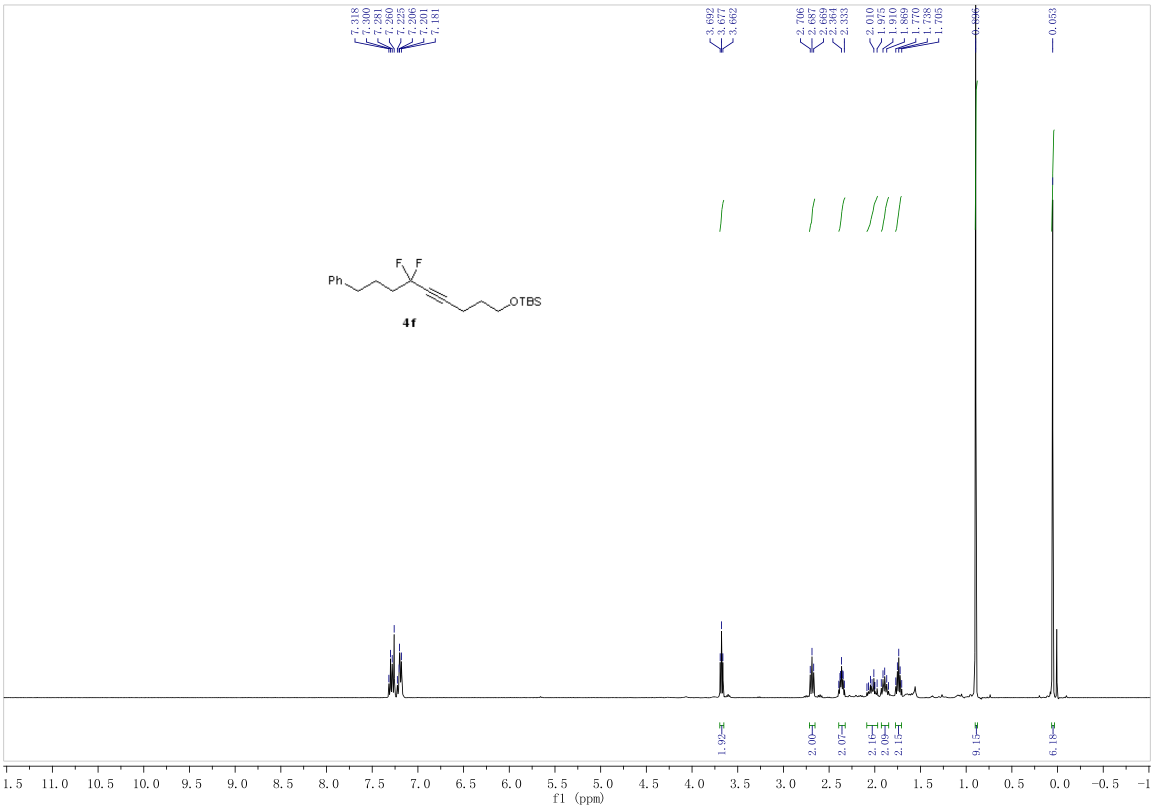

Supplementary Figure 104. **^{1}H NMR of *tert*-butyl((6,6-difluoro-9-phenylnon-4-yn-1-yl)oxy)dimethylsilane (4f)**

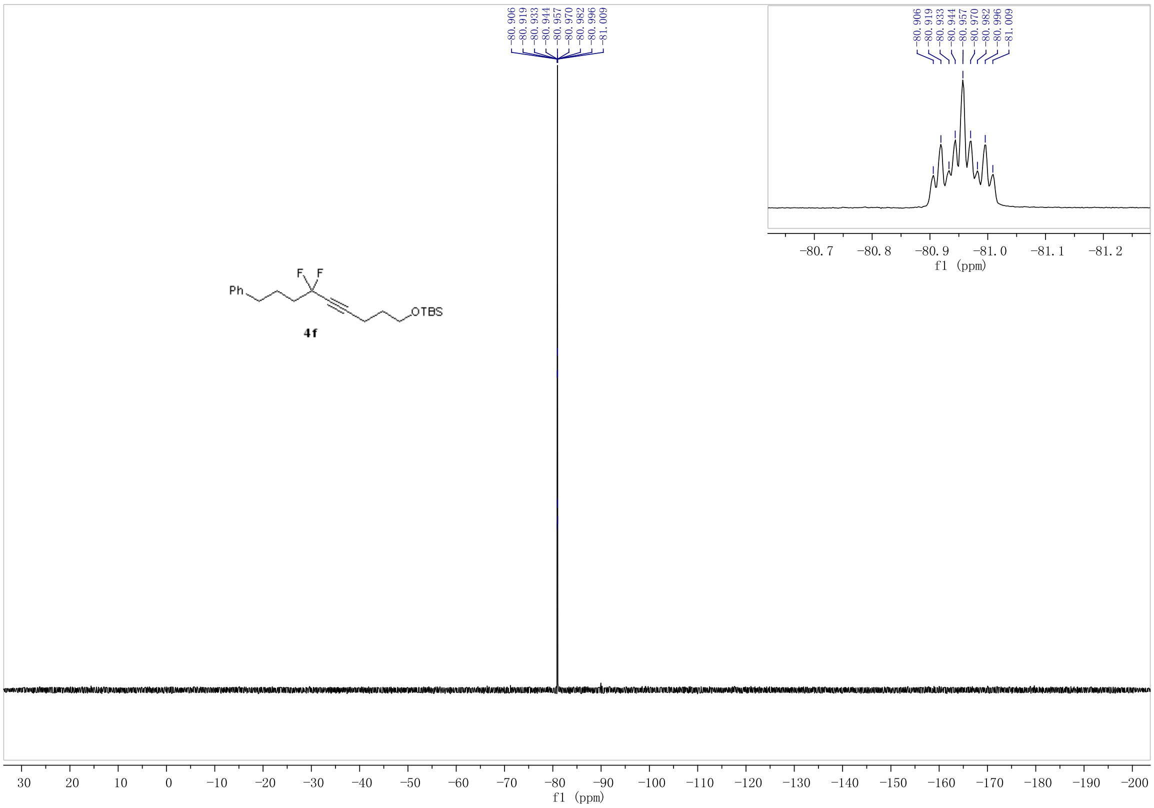

Supplementary Figure 105. ^{19}F NMR of *tert*-butyl((6,6-difluoro-9-phenylnon-4-yn-1-yl)oxy)dimethylsilane (4f)

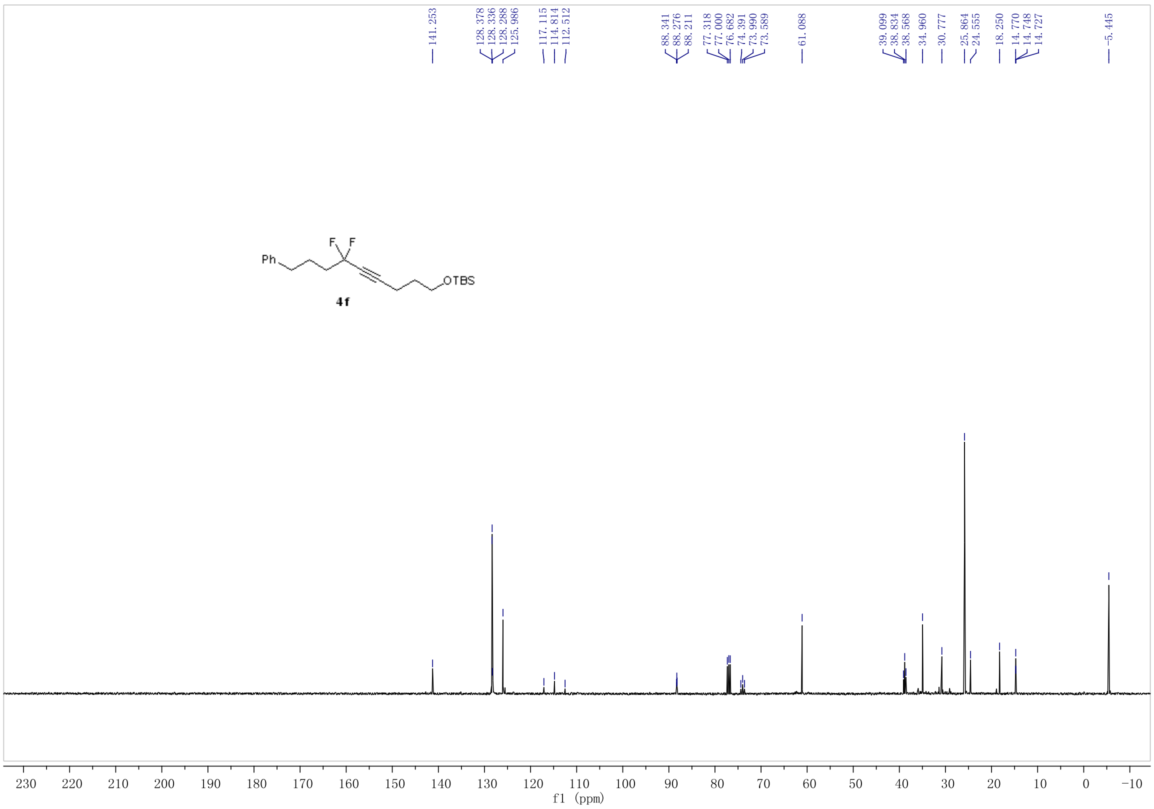

Supplementary Figure 106. ^{13}C NMR of *tert*-butyl((6,6-difluoro-9-phenylnon-4-yn-1-yl)oxy)dimethylsilane (4f)

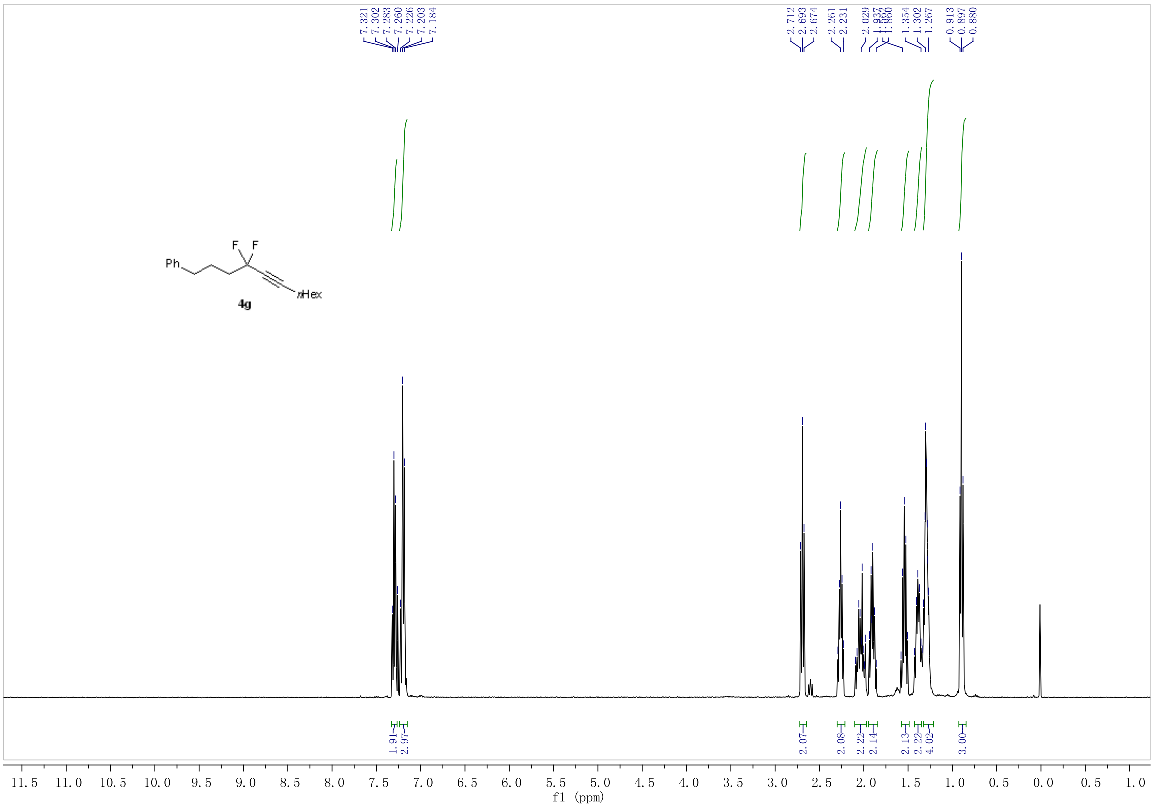

Supplementary Figure 107. ^{1}H NMR of (4,4-difluorododec-5-yn-1-yl)benzene (4g)

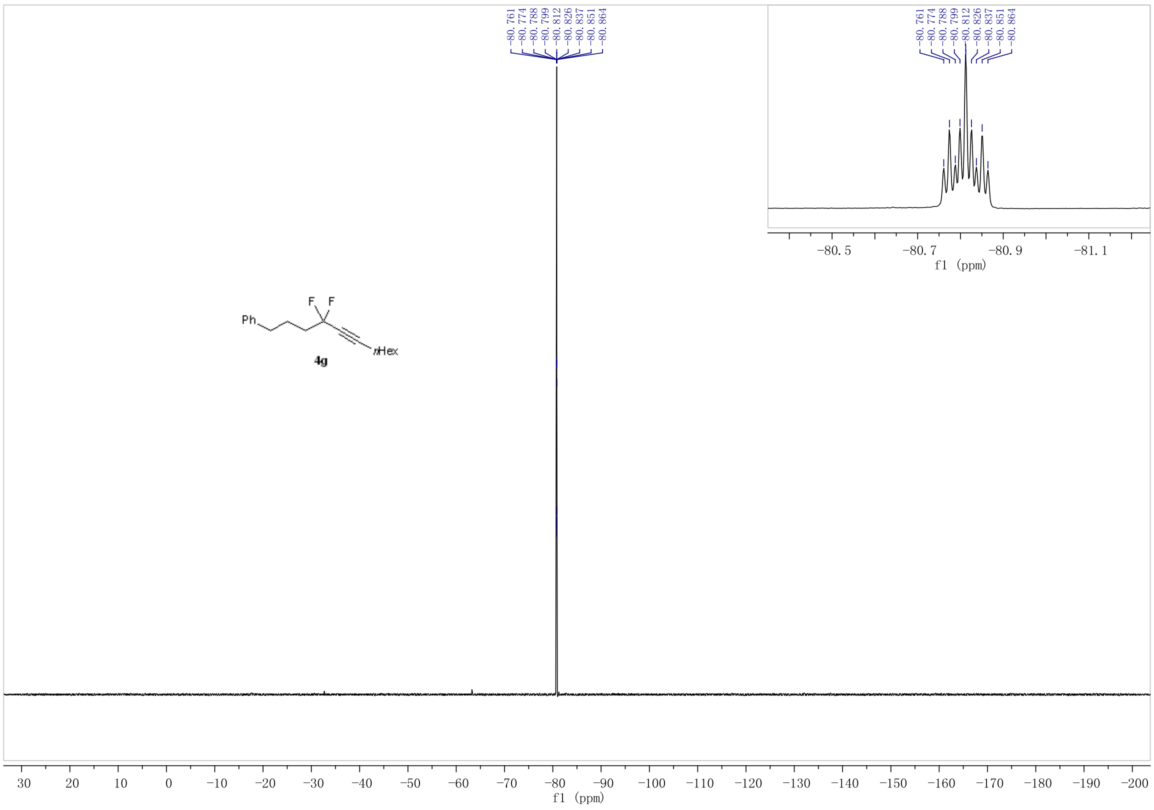

Supplementary Figure 108. ^{19}F NMR of (4,4-difluorododec-5-yn-1-yl)benzene (4g)

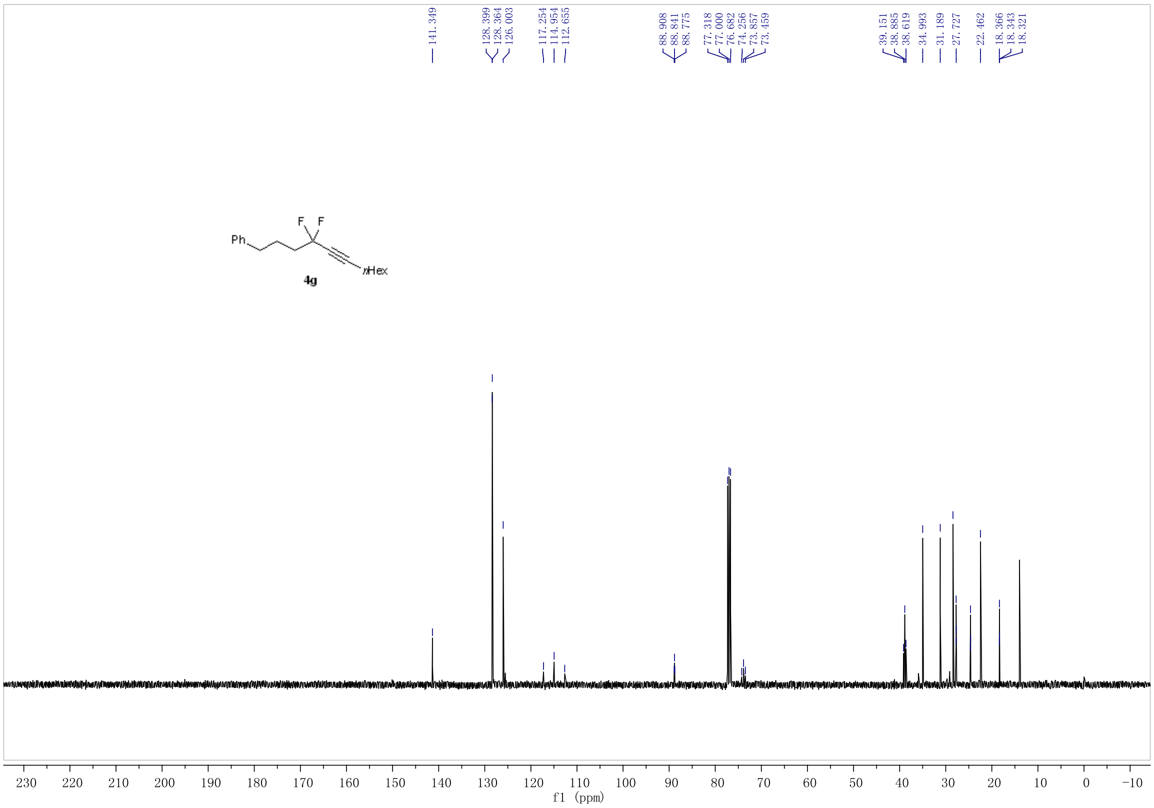

Supplementary Figure 109. ^{13}C NMR of (4,4-difluorododec-5-yn-1-yl)benzene (4g)

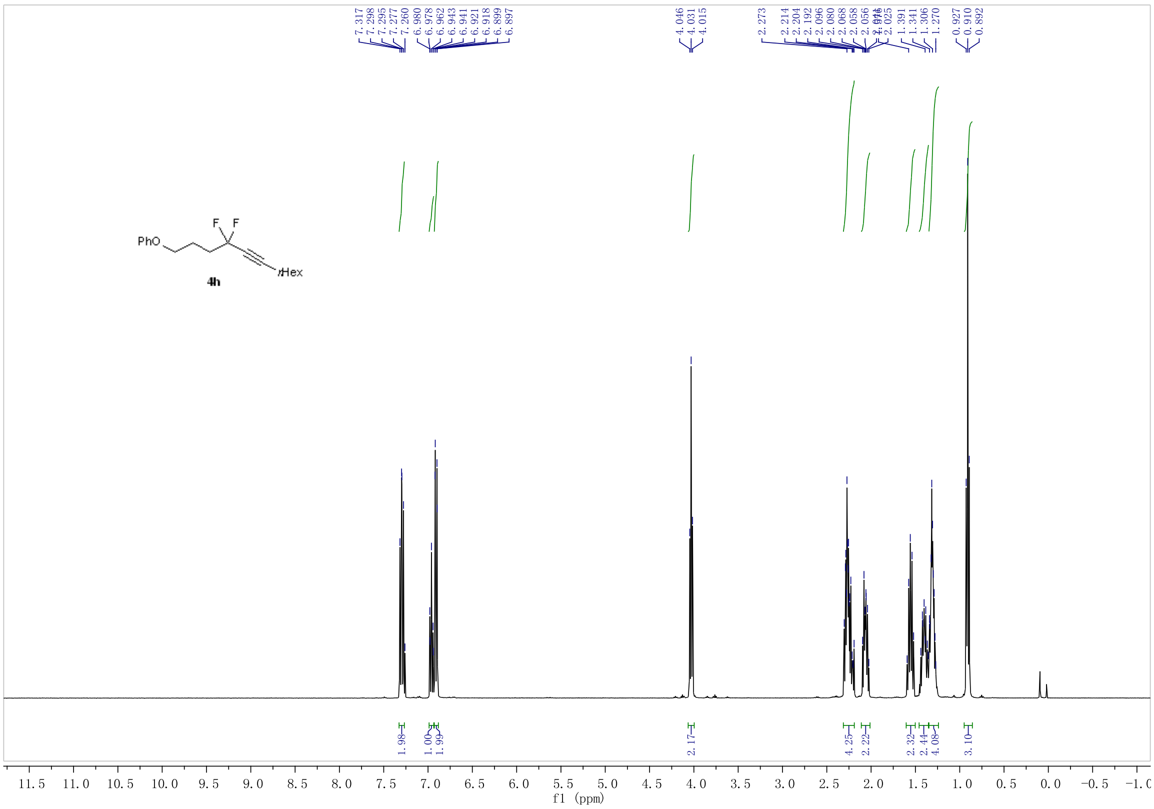

Supplementary Figure 110. ^{1}H NMR of ((4,4-difluorododec-5-yn-1-yl)oxy)benzene (4h)

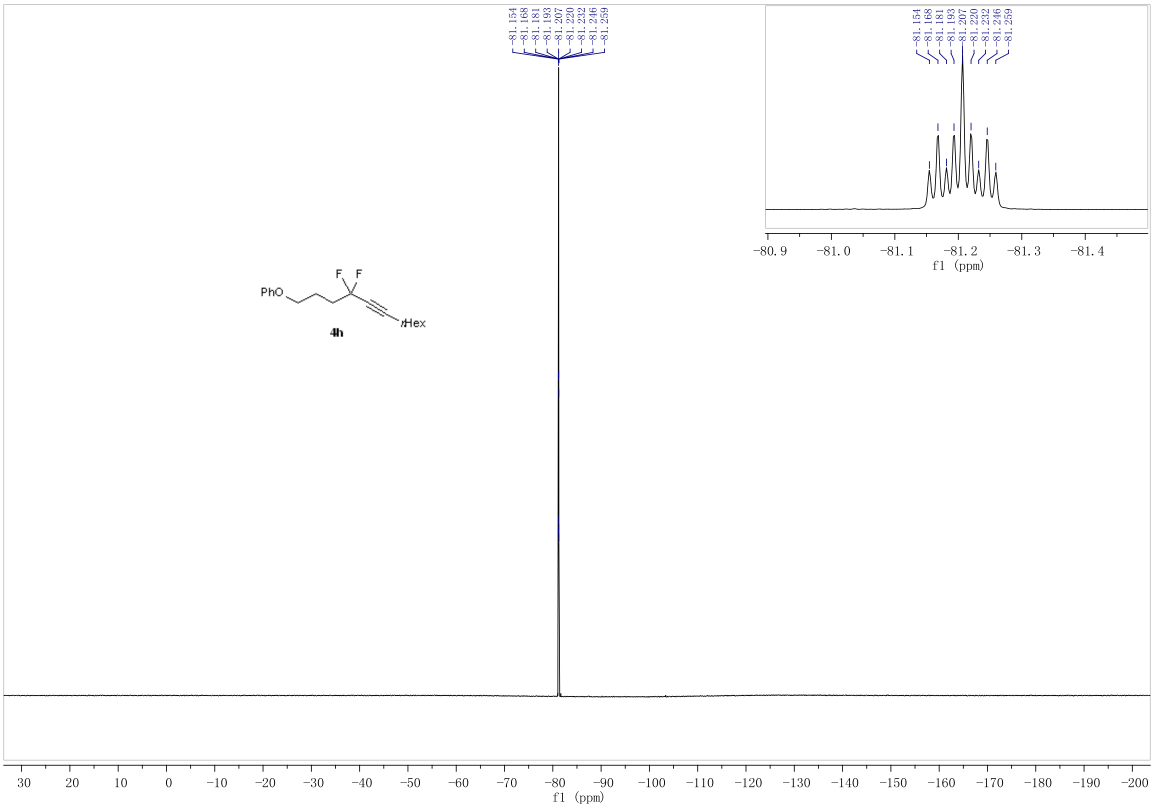

Supplementary Figure 111. ^{19}**F NMR of ((4,4-difluorododec-5-yn-1-yl)oxy)benzene (4h)**

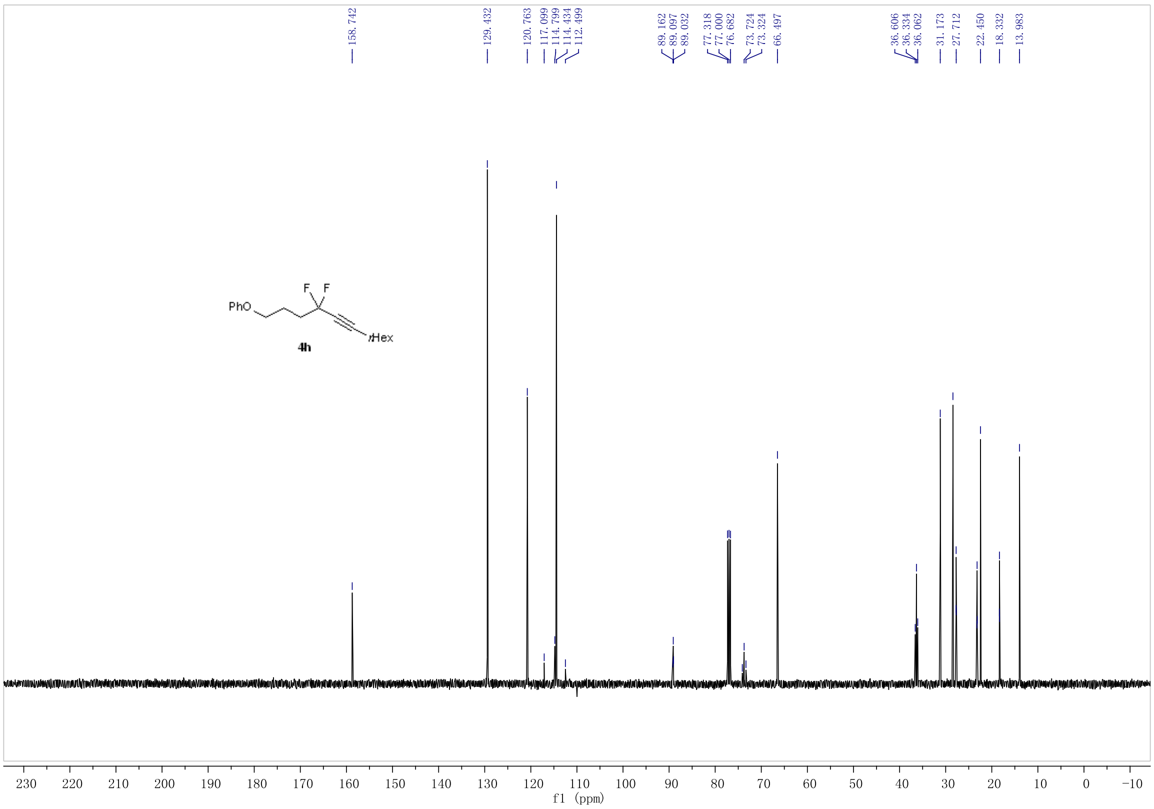

Supplementary Figure 112. ^{13}**C NMR of ((4,4-difluorododec-5-yn-1-yl)oxy)benzene (4h)**

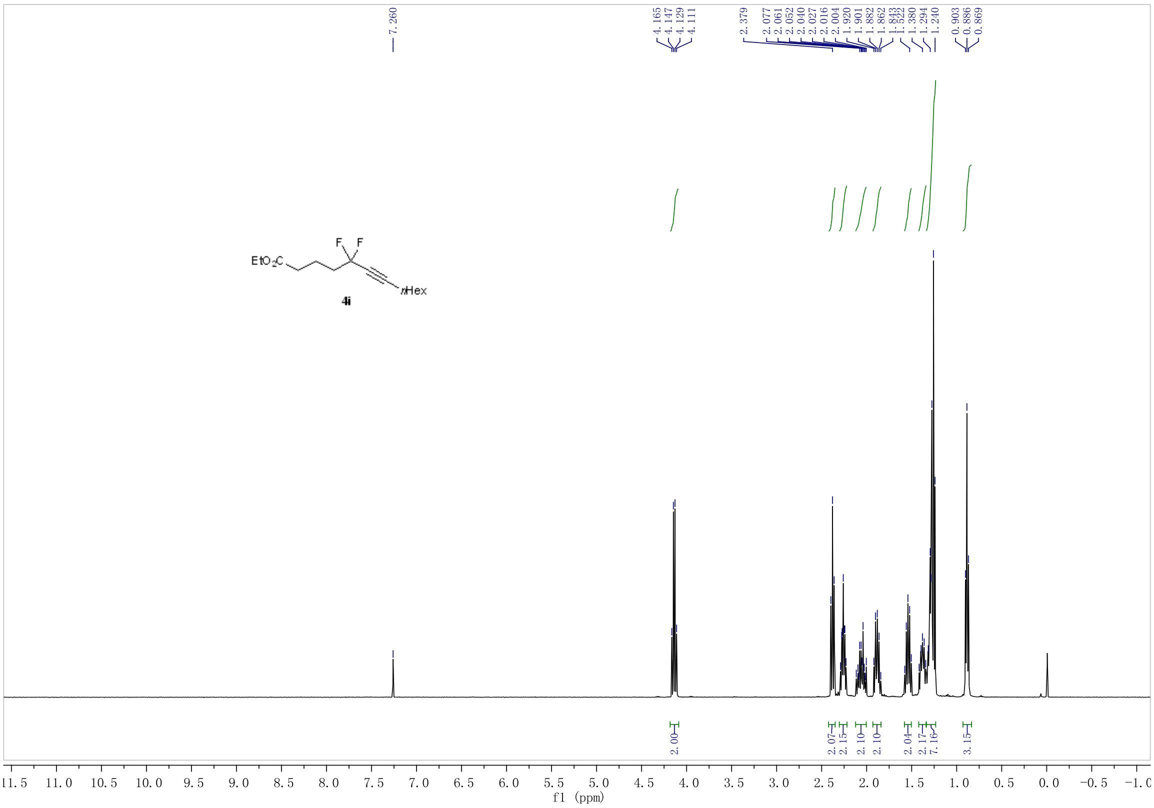

Supplementary Figure 113. ^{1}H NMR of ethyl-5,5-difluorotridec-6-ynoate (4i)

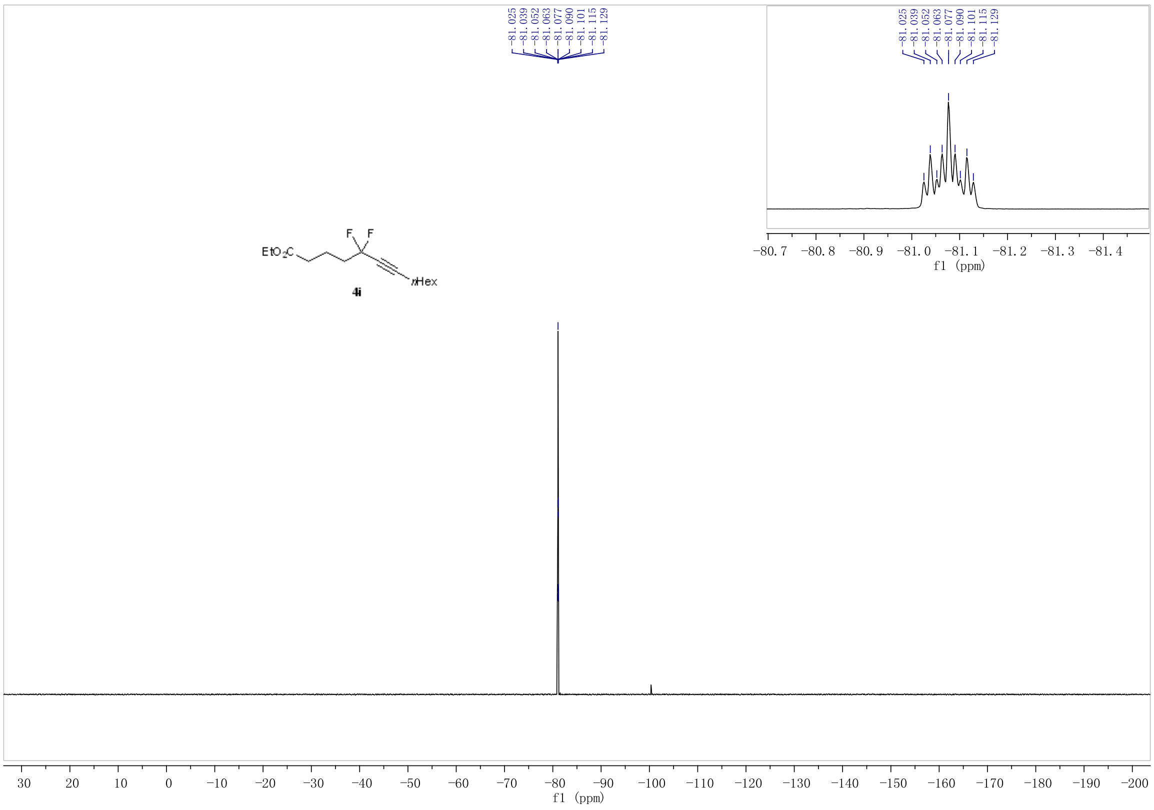

Supplementary Figure 114. ^{19}F NMR of ethyl-5,5-difluorotridec-6-ynoate (4i)

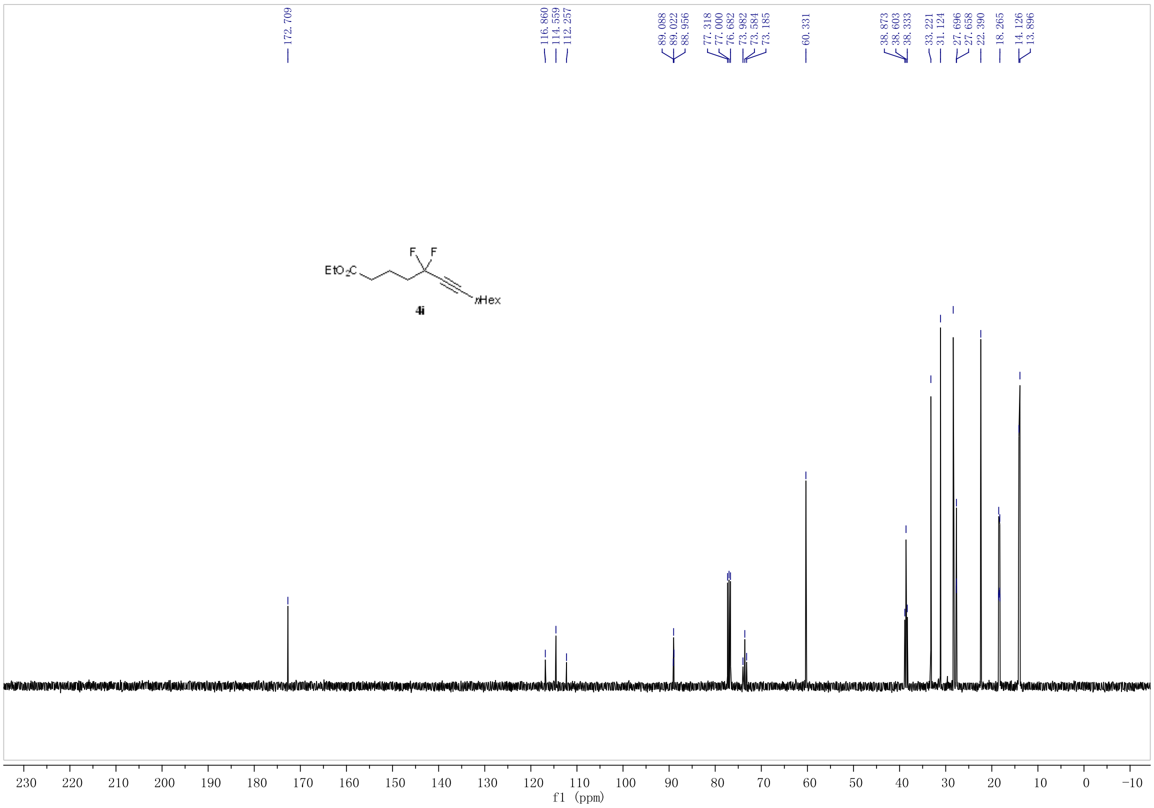

Supplementary Figure 115. ^{13}C NMR of ethyl-5,5-difluorotridec-6-ynoate (4i)

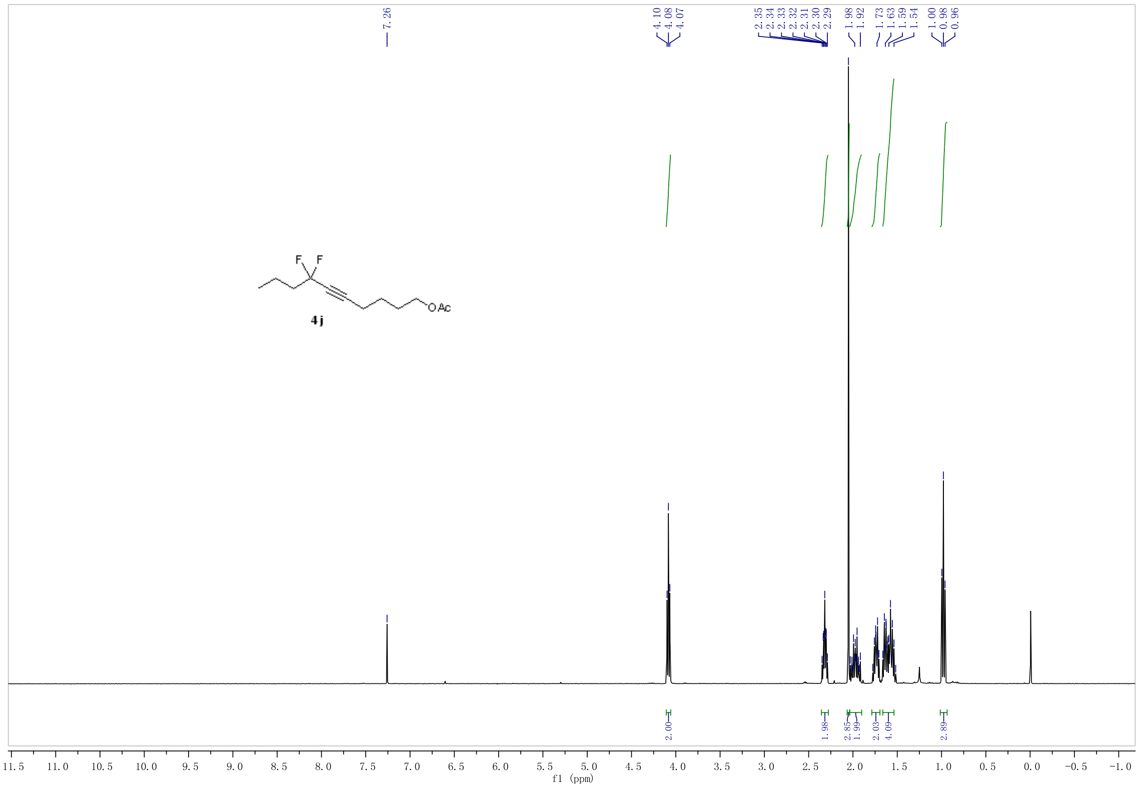

Supplementary Figure 116. ^{1}H NMR of 7,7-difluorodec-5-yn-1-yl acetate (4j)

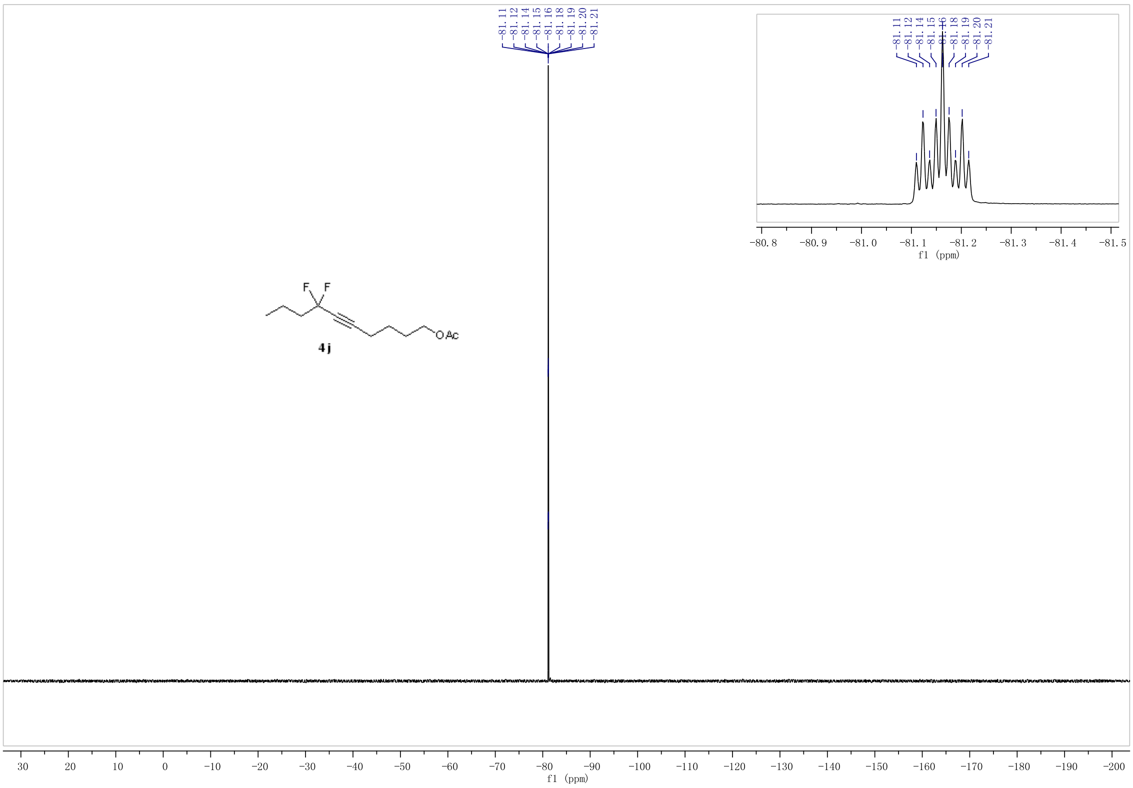

Supplementary Figure 117. ^{19}F NMR of 7,7-difluorodec-5-yn-1-yl acetate (4j)

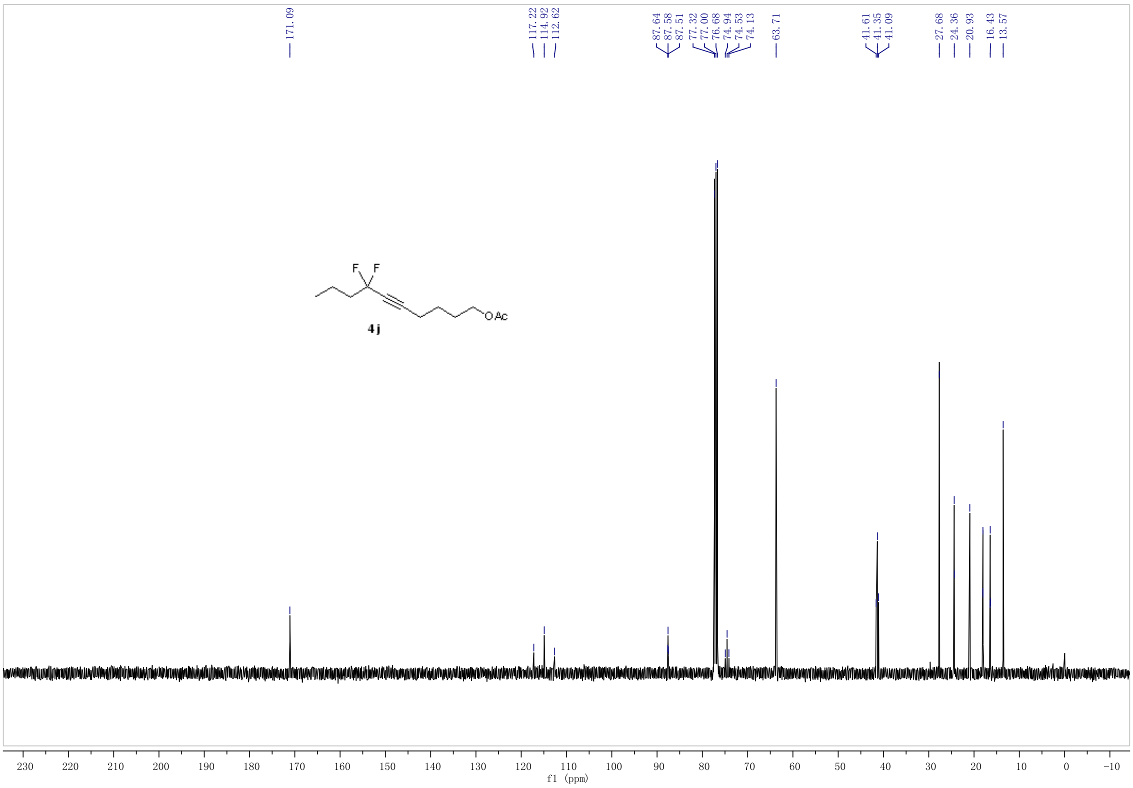

Supplementary Figure 118. ^{13}C NMR of 7,7-difluorodec-5-yn-1-yl acetate (4j)

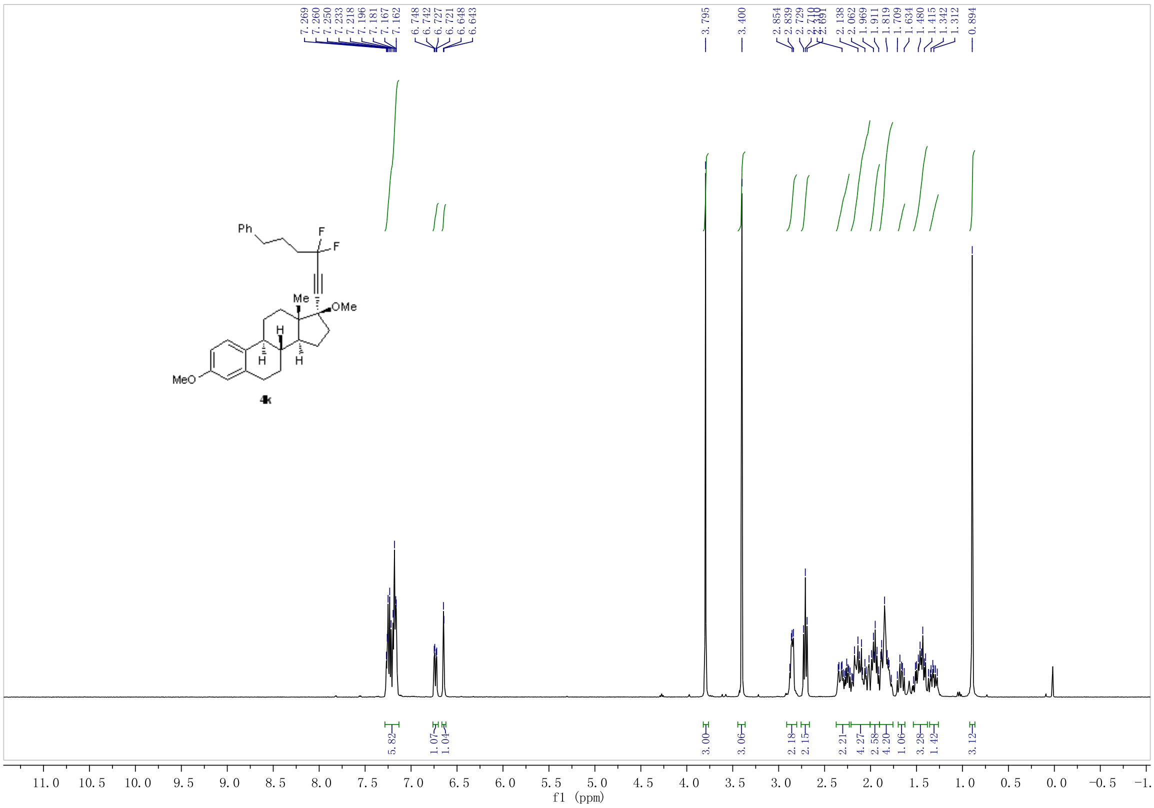

Supplementary Figure 119. ^{1}H NMR of product 4k

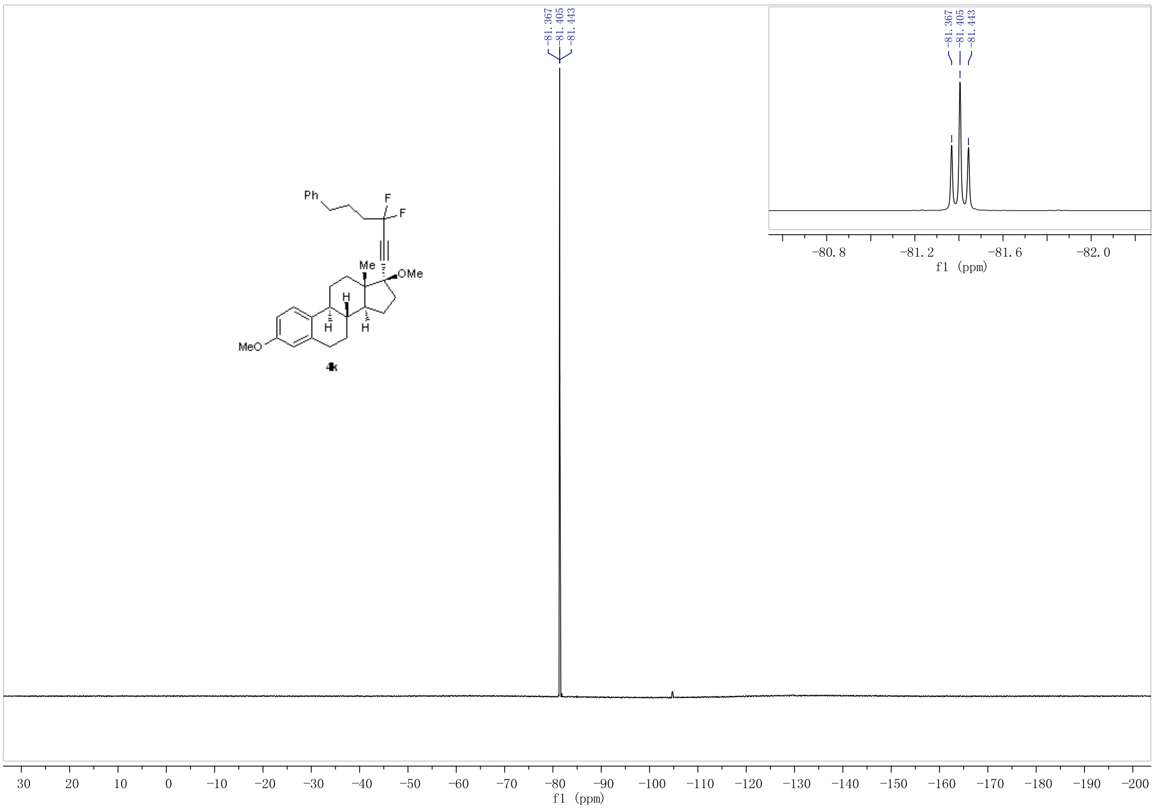

Supplementary Figure 120. ^{19}F NMR of product 4k

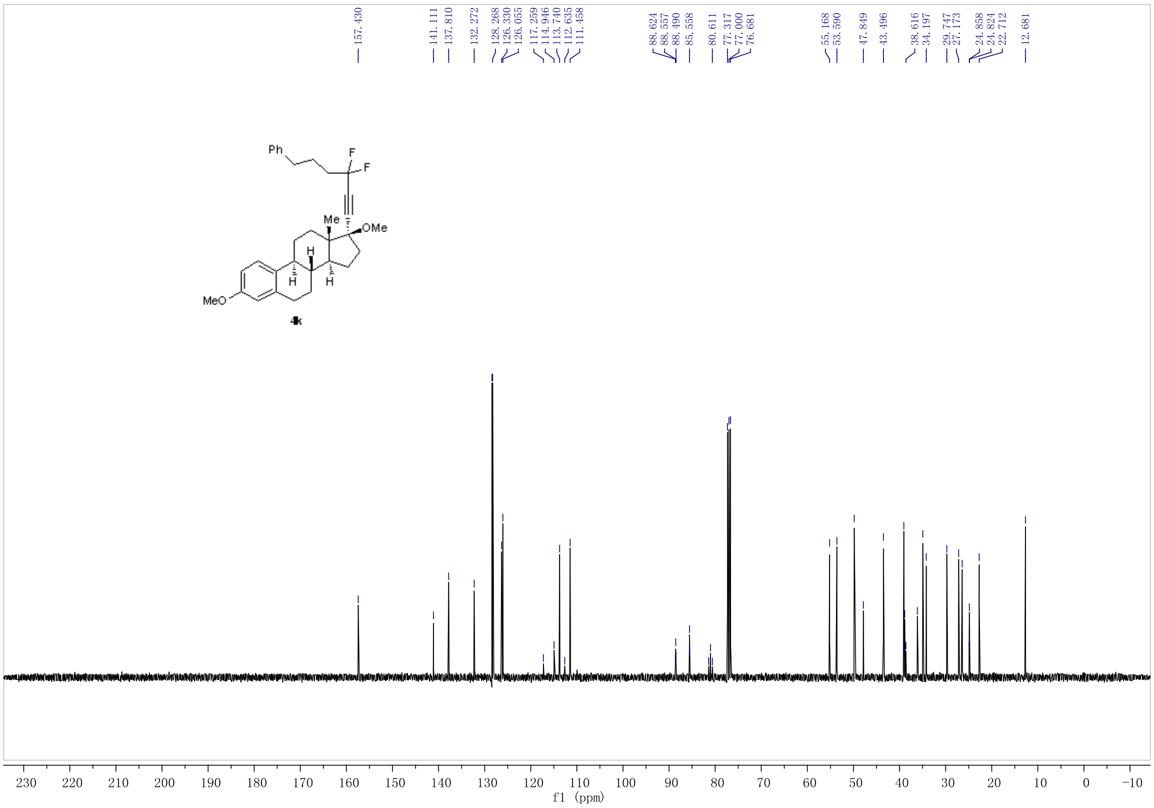

Supplementary Figure 121. ^{13}C NMR of product 4k

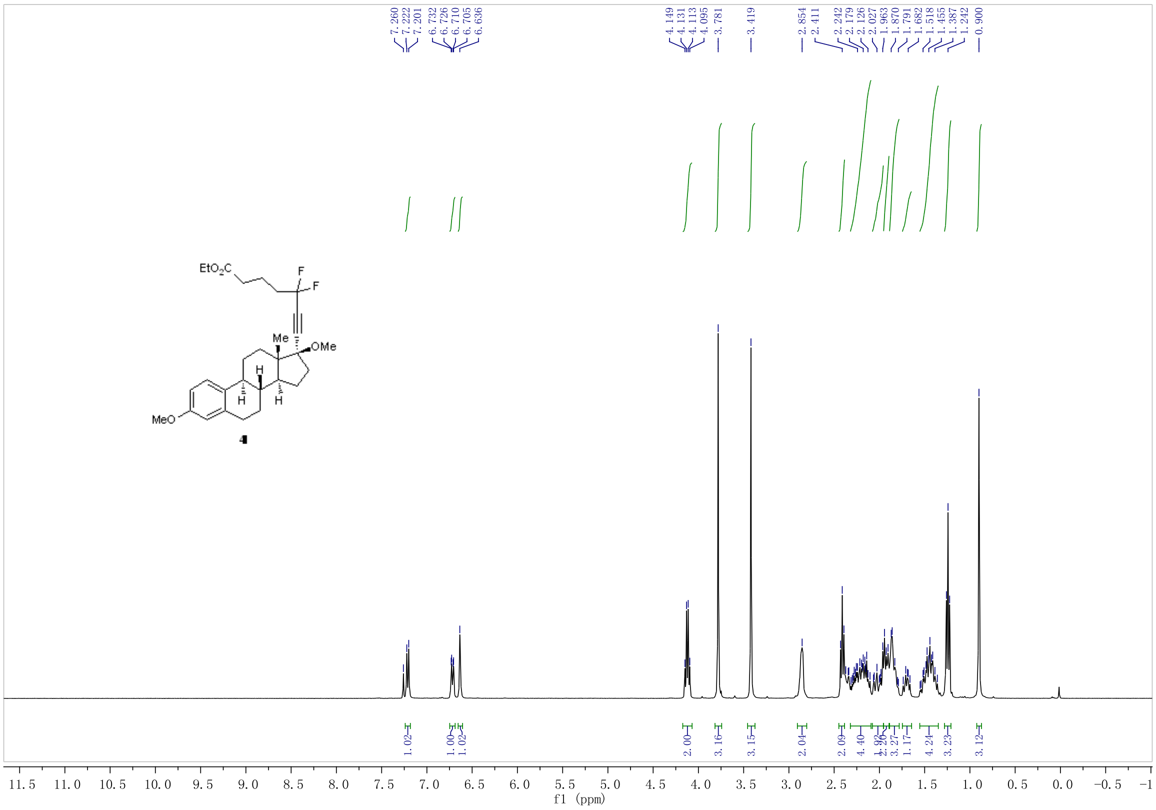

Supplementary Figure 122. ^{1}H NMR of product 4l

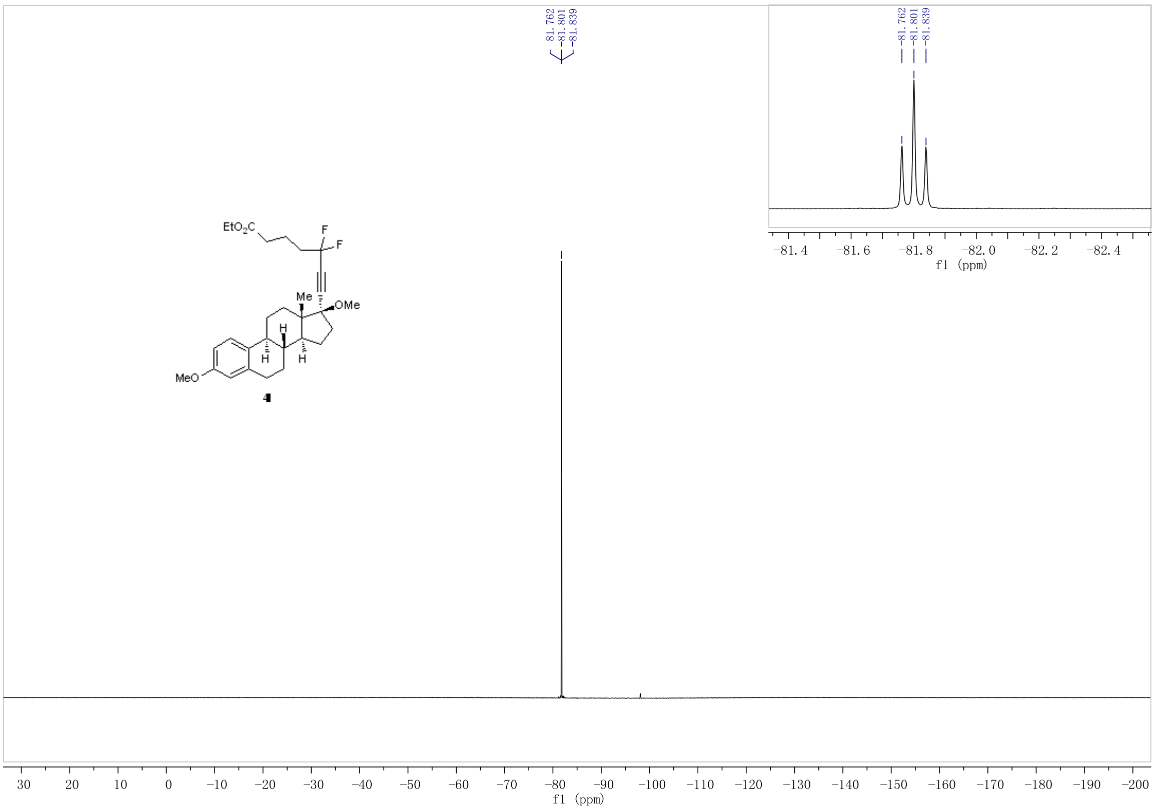

Supplementary Figure 123. ^{19}**F NMR of product 4l**

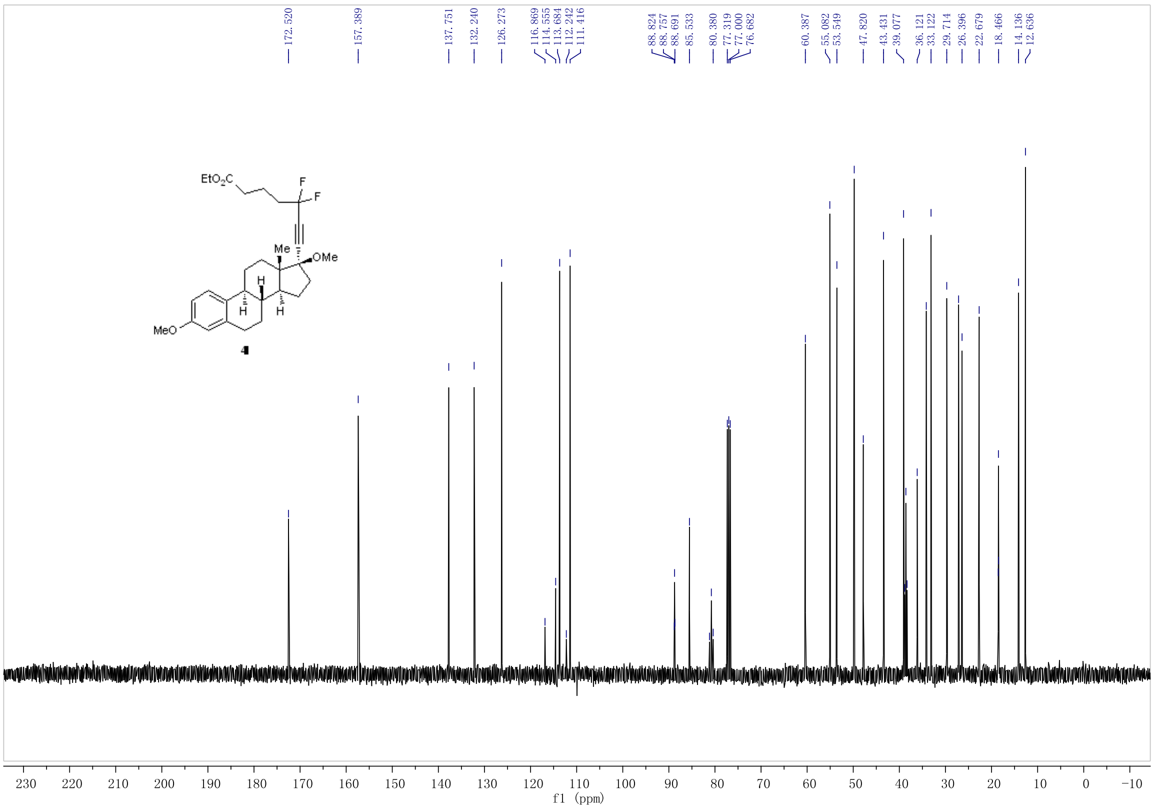

Supplementary Figure 124. ^{13}**C NMR of product 4l**

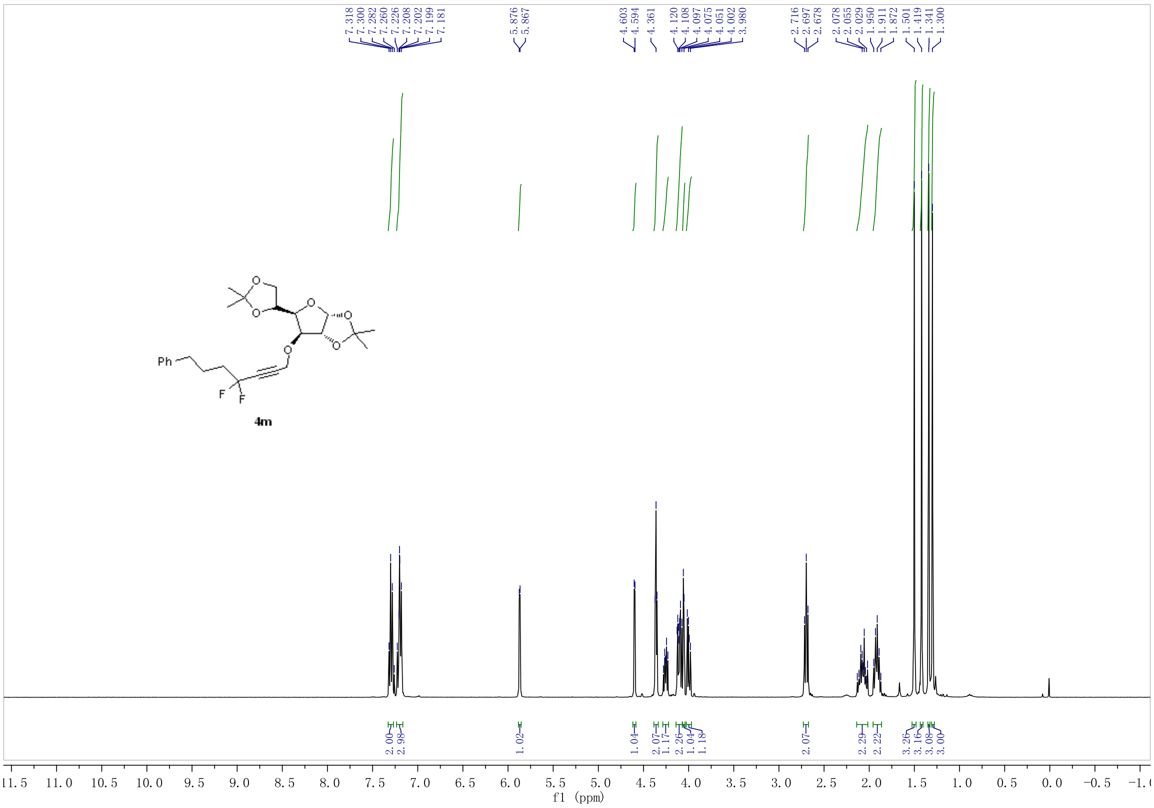

Supplementary Figure 125. ^{1}H NMR of product 4m

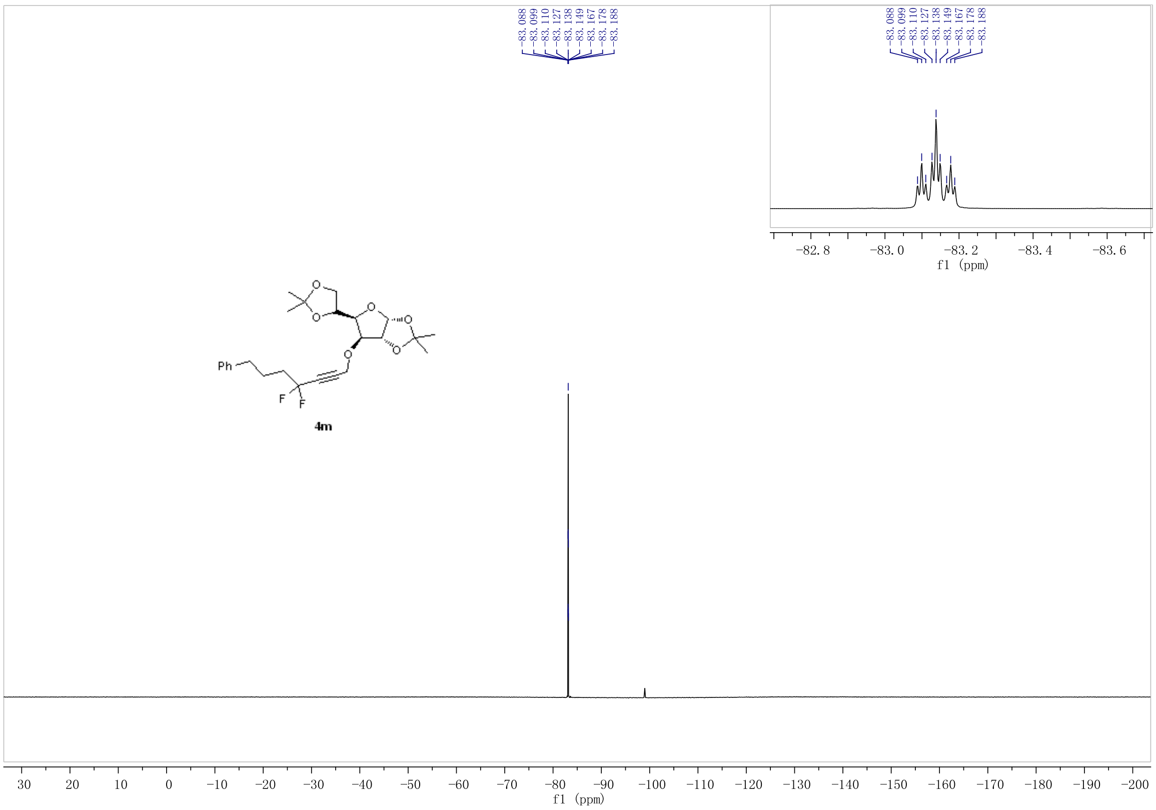

Supplementary Figure 126. ^{19}F NMR of product 4m

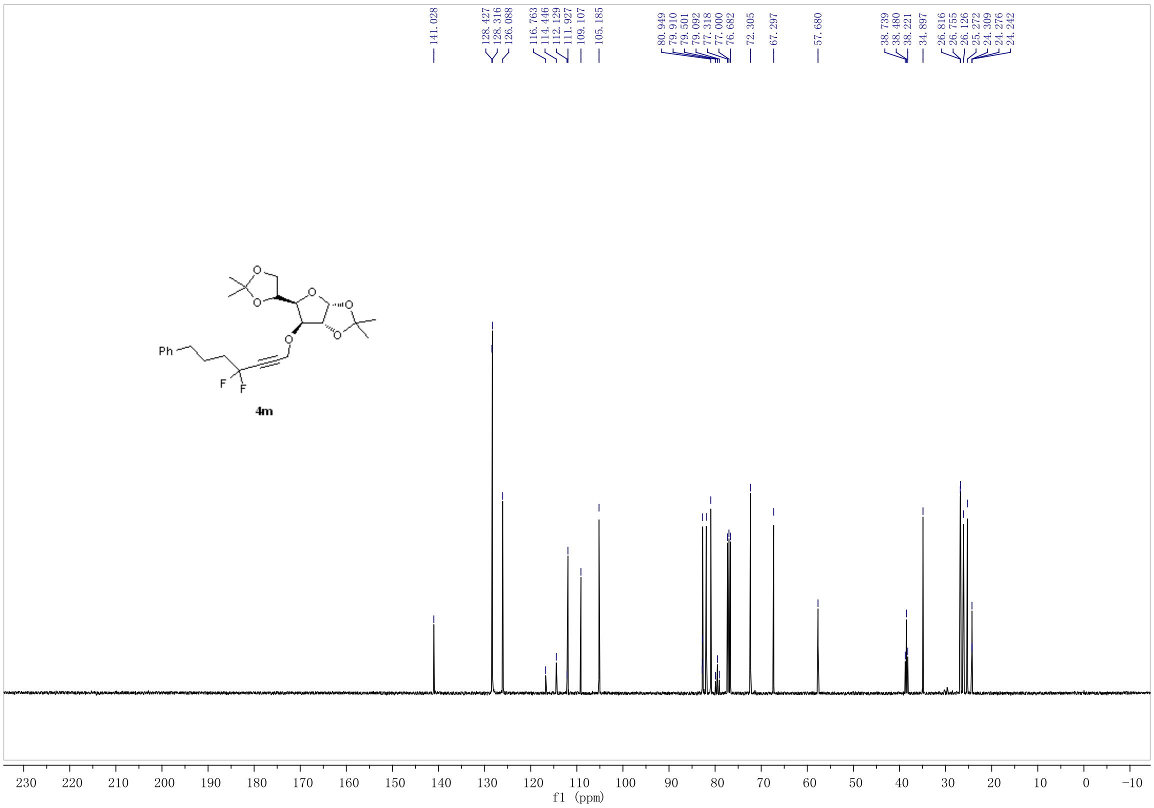

Supplementary Figure 127. ^{13}C NMR of product 4m

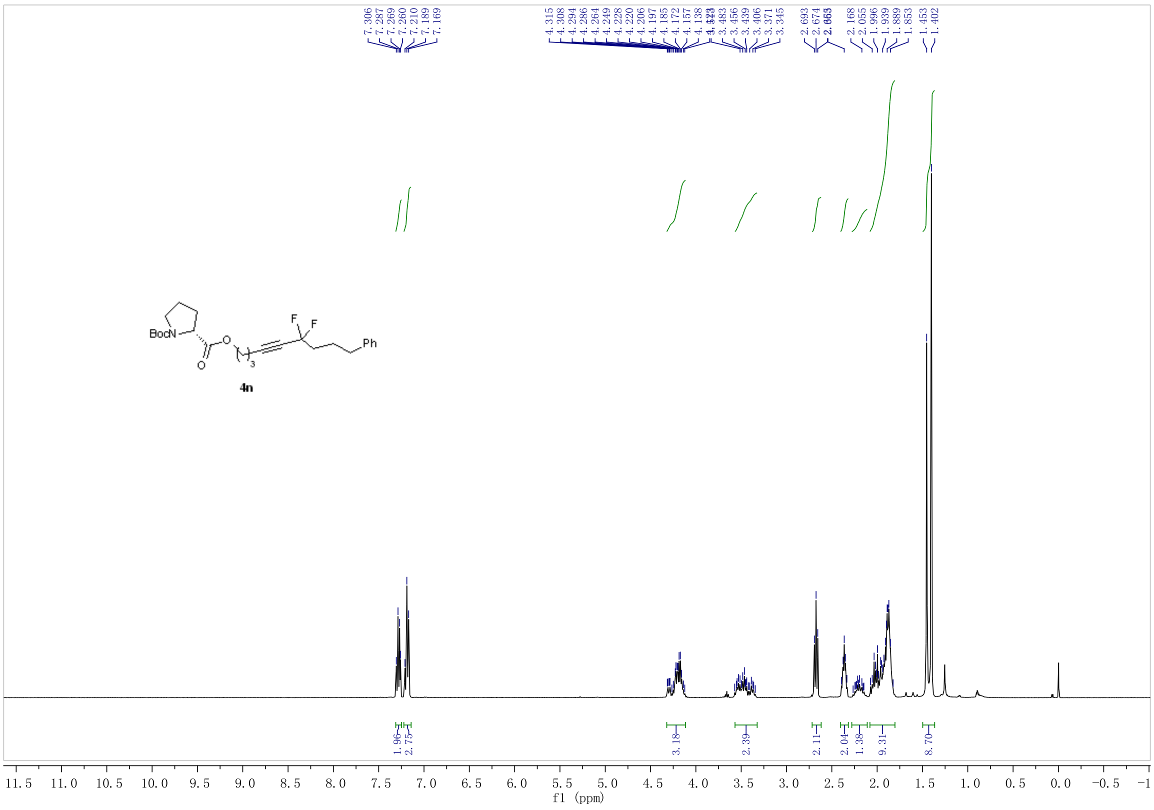

Supplementary Figure 128. ^{1}H NMR of 1-(*tert*-butyl)-2-(6,6-difluoro-9-phenylnon-4-yn-1-yl) pyrrolidine-1,2-dicarboxylate (4n)

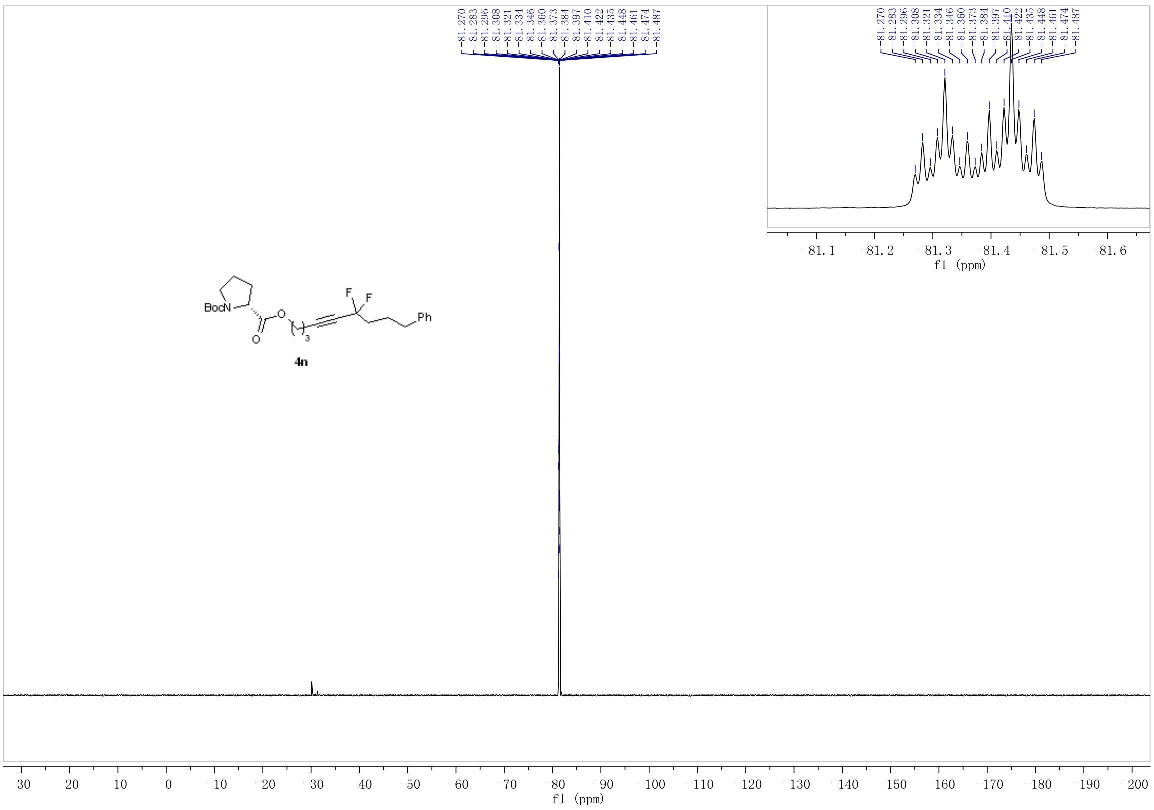

Supplementary Figure 129. ^{19}**F NMR of 1-(*tert*-butyl)-2-(6,6-difluoro-9-phenylnon-4-yn-1-yl) pyrrolidine-1,2-dicarboxylate (4n)**

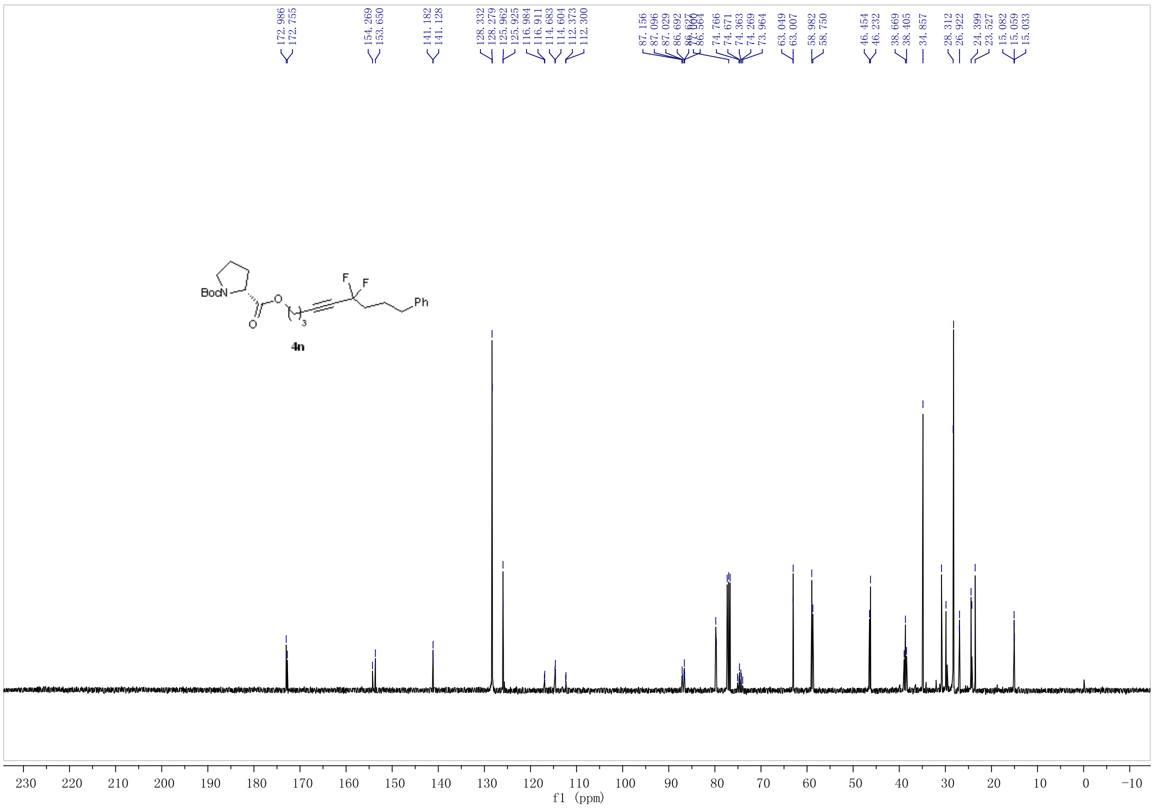

Supplementary Figure 130. ^{13}**C NMR of 1-(*tert*-butyl)-2-(6,6-difluoro-9-phenylnon-4-yn-1-yl) pyrrolidine-1,2-dicarboxylate (4n)**

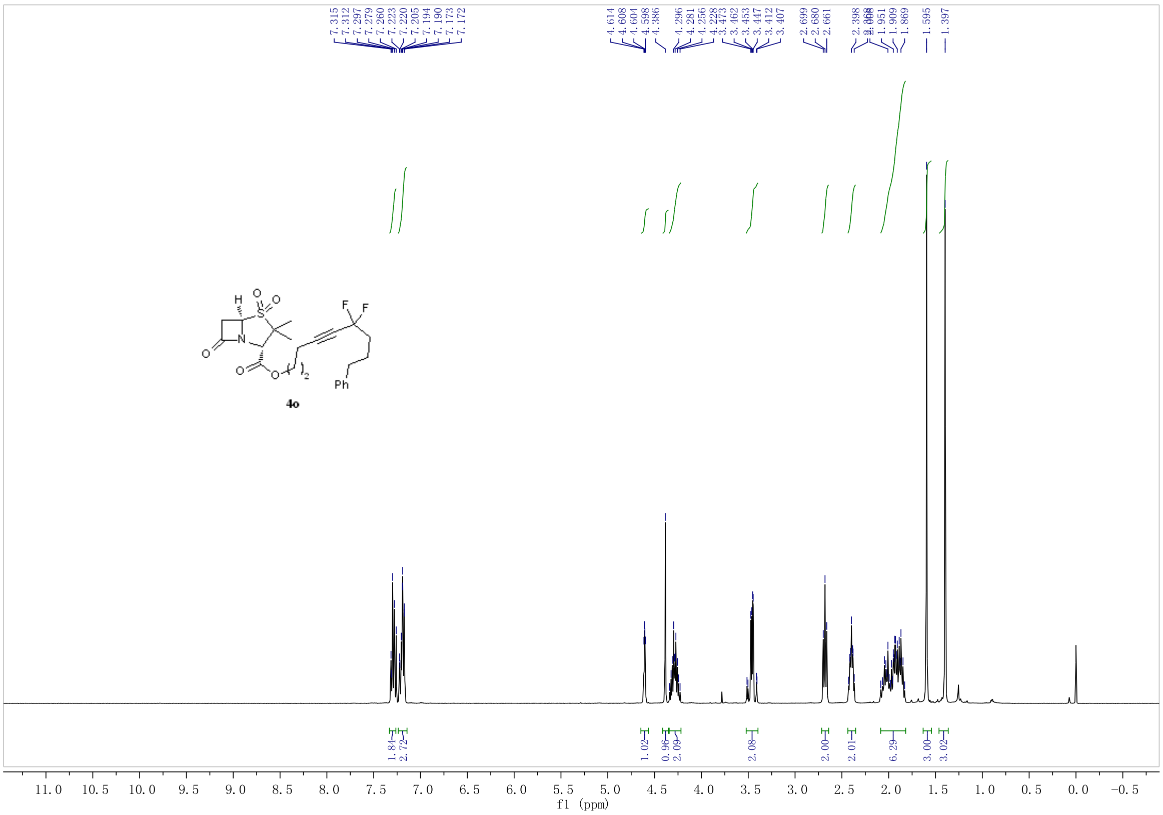

Supplementary Figure 131. ^{1}H NMR of product 4o

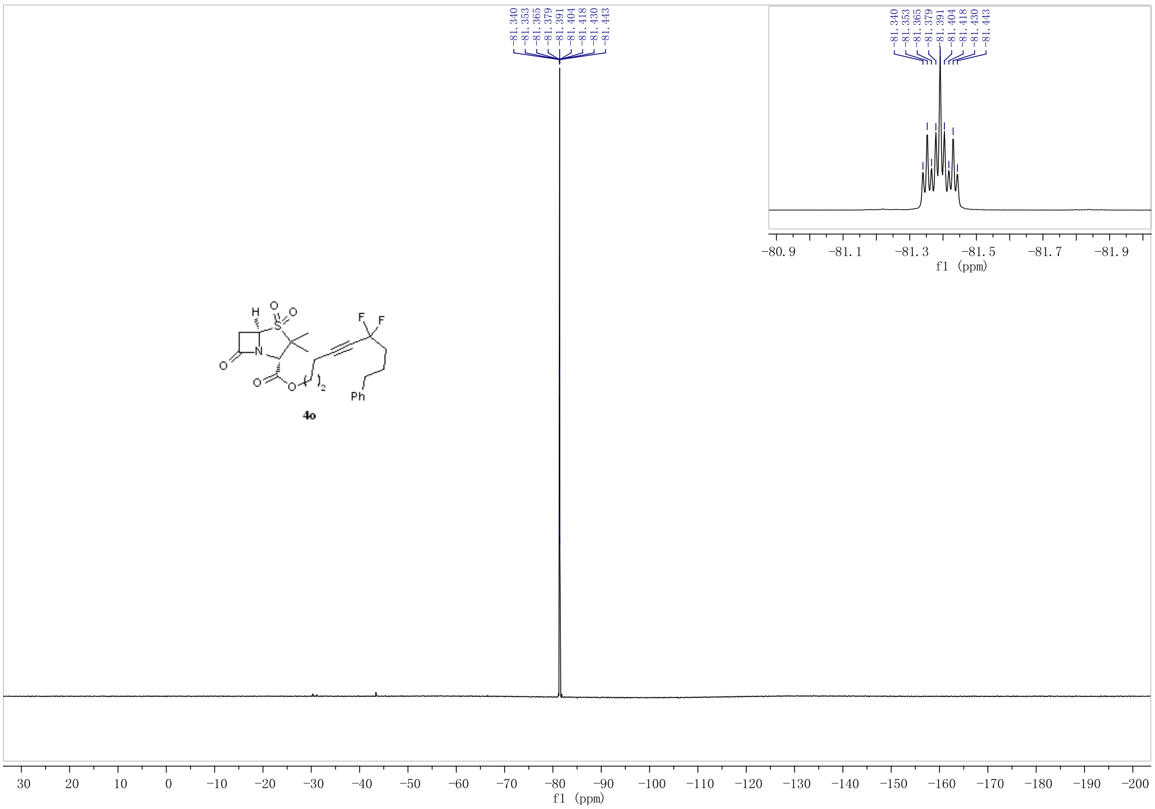

Supplementary Figure 132. ^{19}F NMR of product 4o

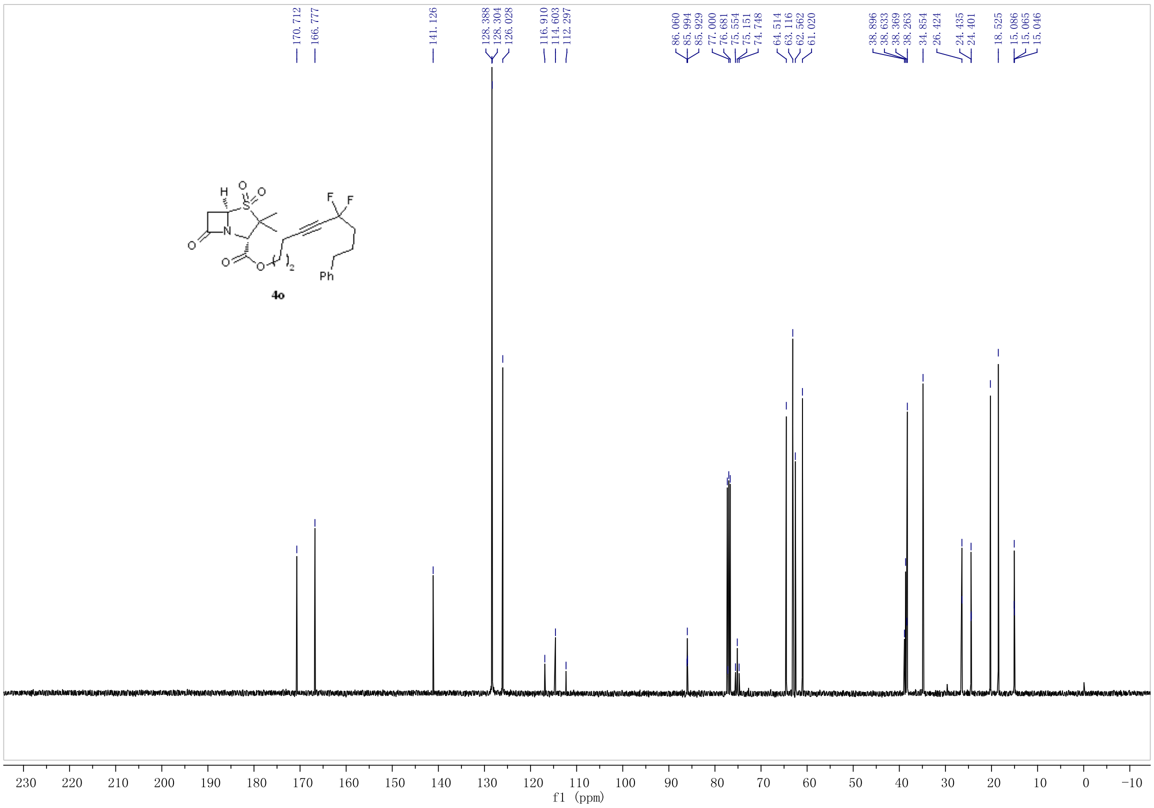

Supplementary Figure 133. ^{13}C NMR of product 4o

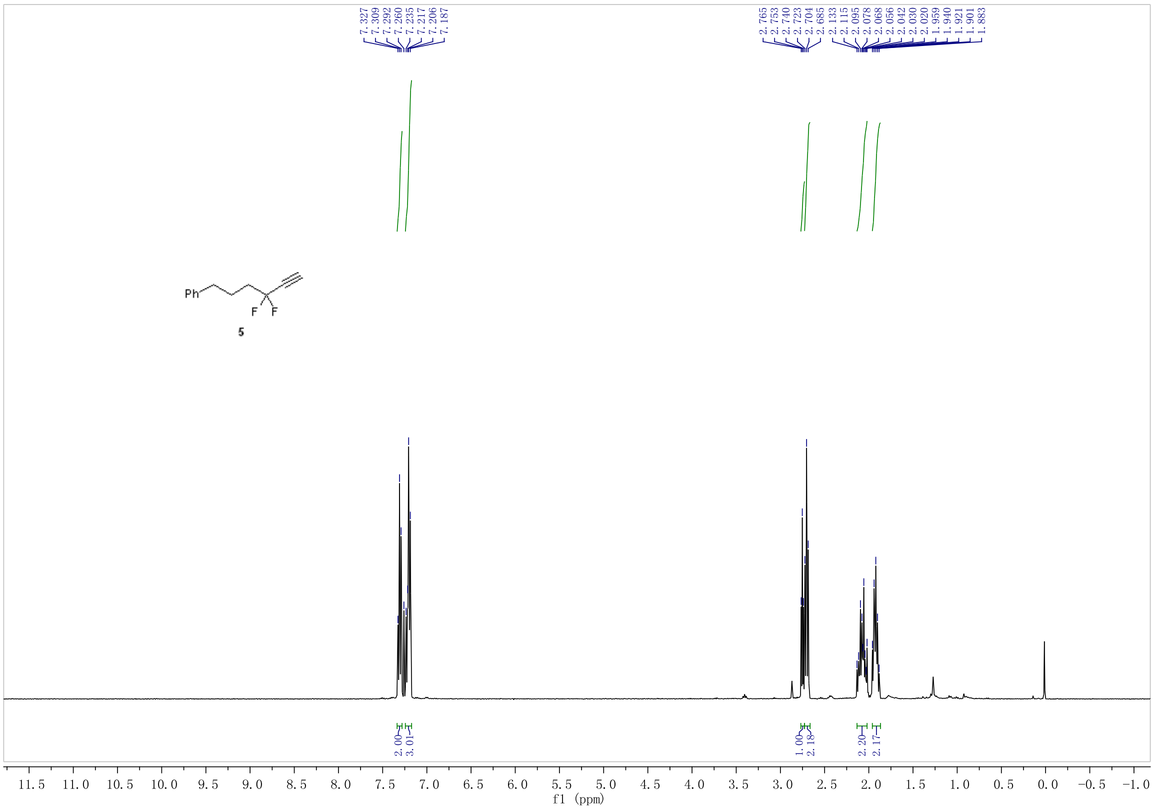

Supplementary Figure 134. ^{1}H NMR of (4,4-difluorohex-5-yn-1-yl)benzene (5)

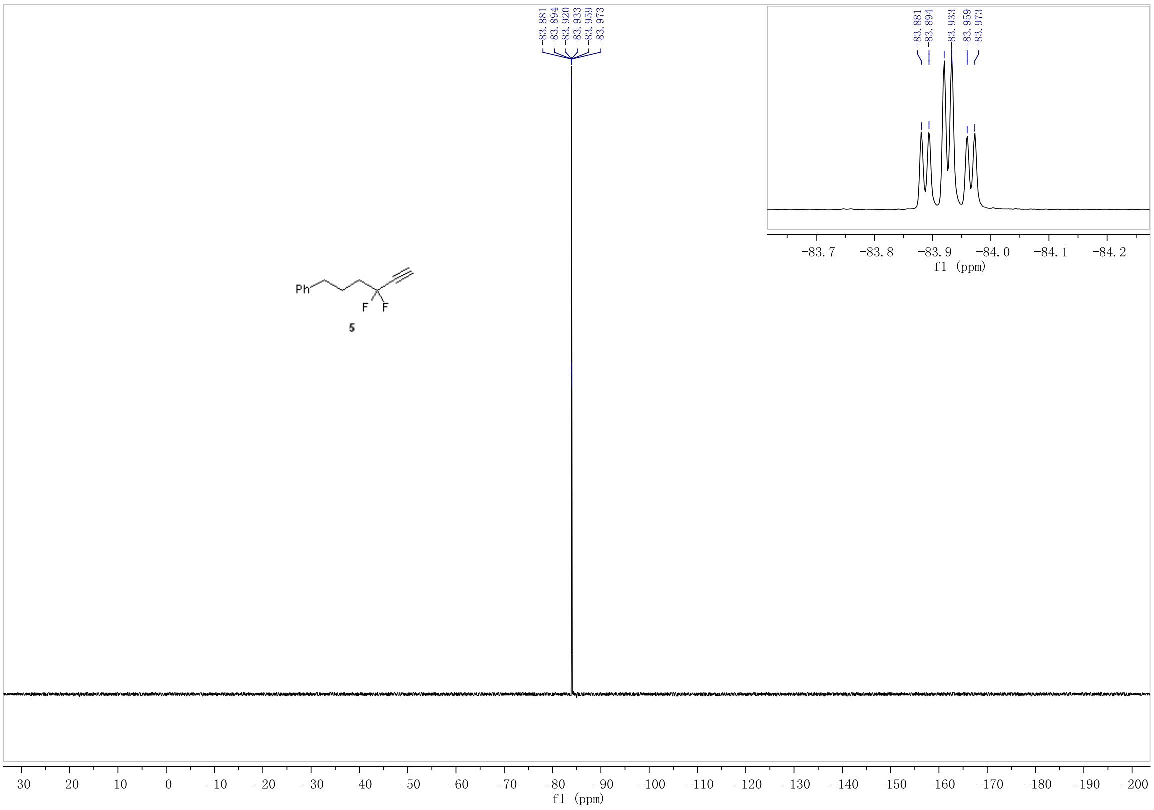

Supplementary Figure 135. ^{19}F NMR of (4,4-difluorohex-5-yn-1-yl)benzene (5)

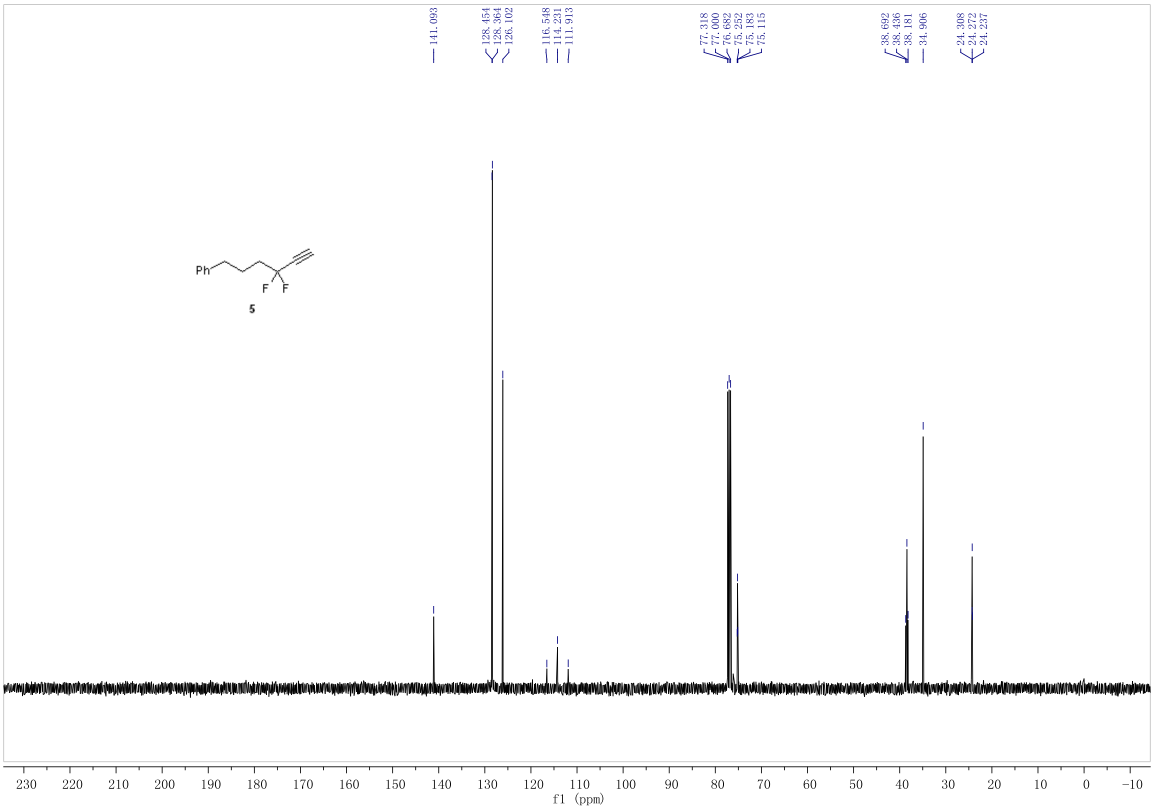

Supplementary Figure 136. ^{13}C NMR of (4,4-difluorohex-5-yn-1-yl)benzene (5)

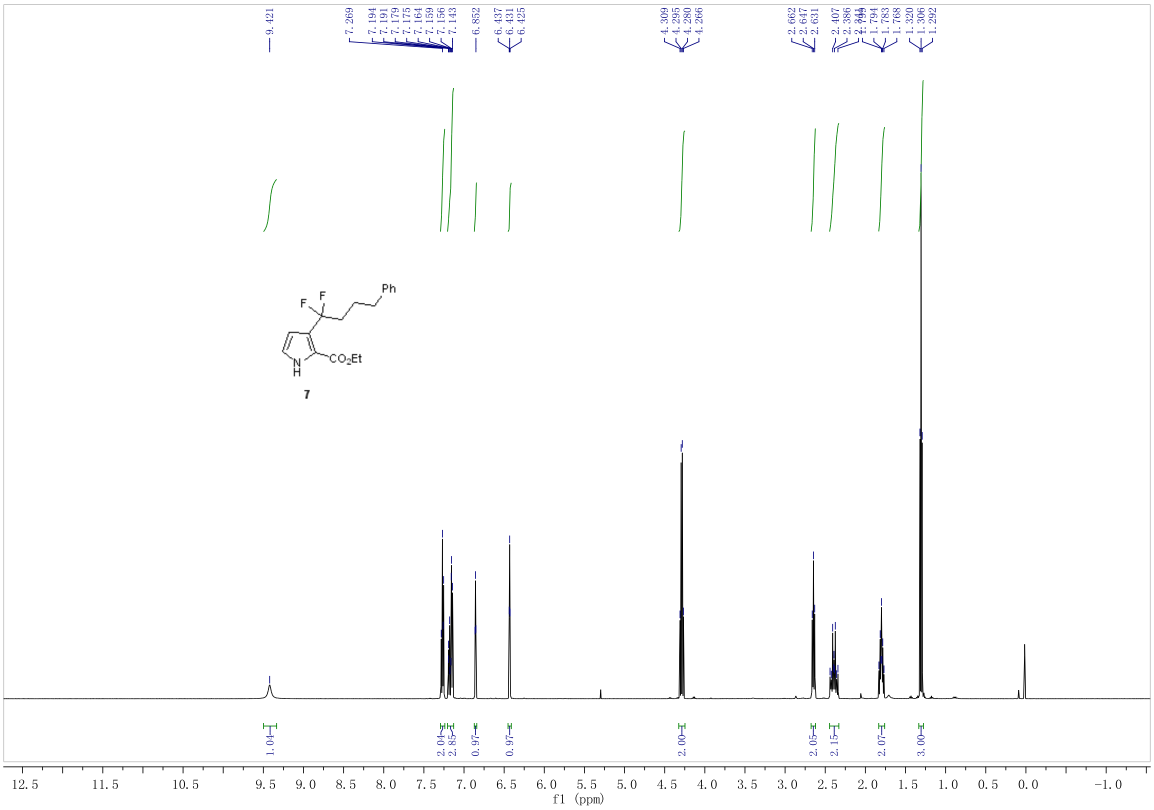

Supplementary Figure 137. ^{1}**H NMR of (ethyl 3-(1,1-difluoro-4-phenylbutyl)-1H-pyrrole-2-carboxylate (7)**

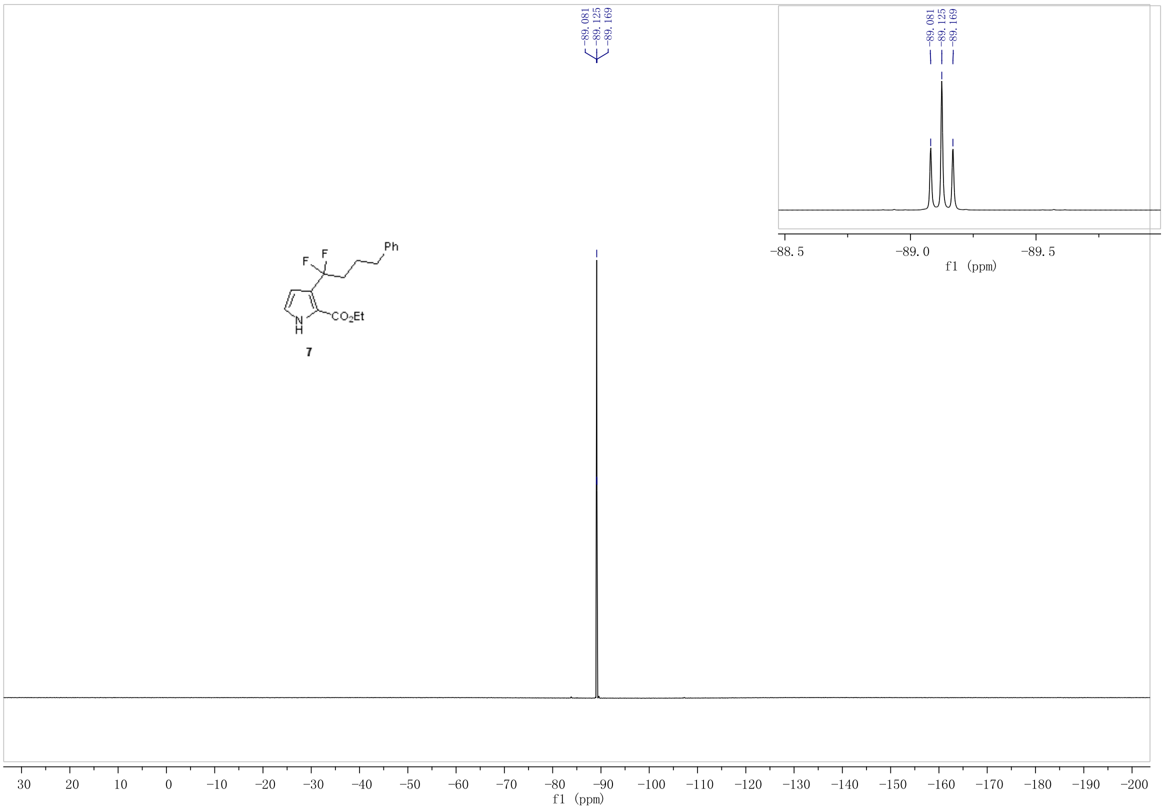

Supplementary Figure 138. ^{19}**F NMR of (ethyl 3-(1,1-difluoro-4-phenylbutyl)-1H-pyrrole-2-carboxylate (7)**

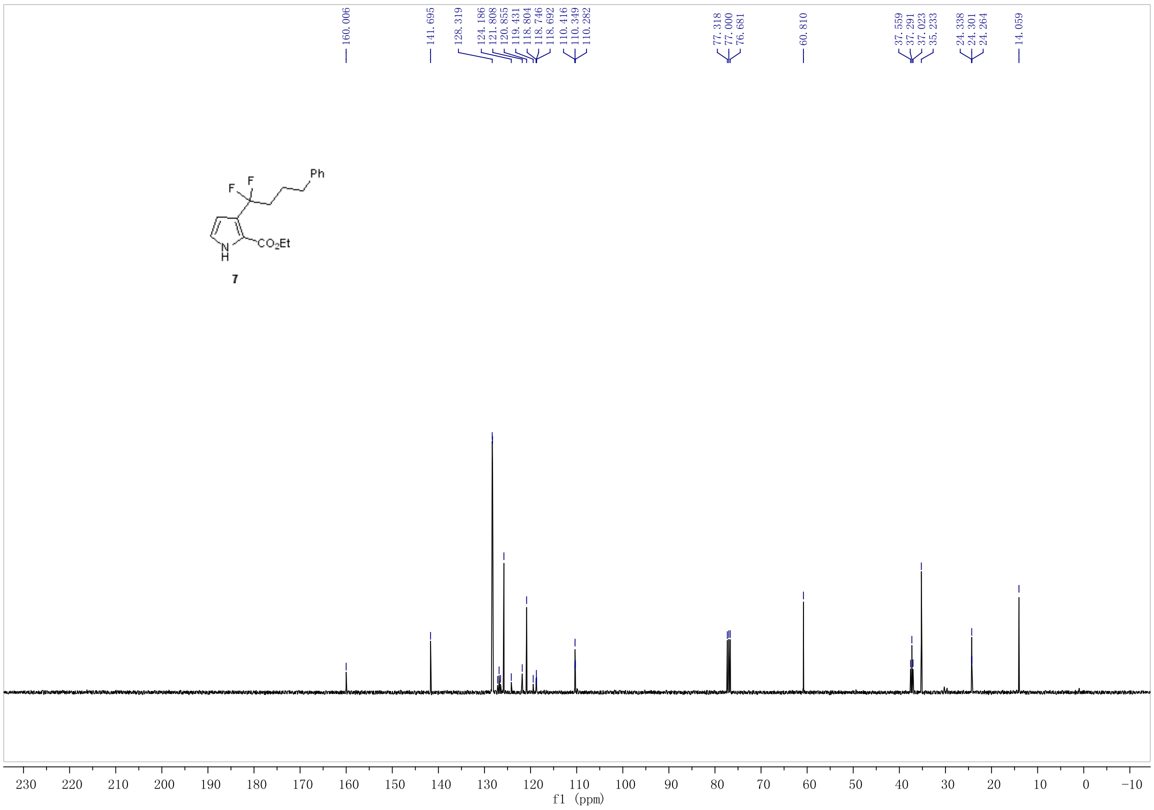

Supplementary Figure 139. ^{13}C NMR of (ethyl 3-(1,1-difluoro-4-phenylbutyl)-1H-pyrrole-2-carboxylate (7)

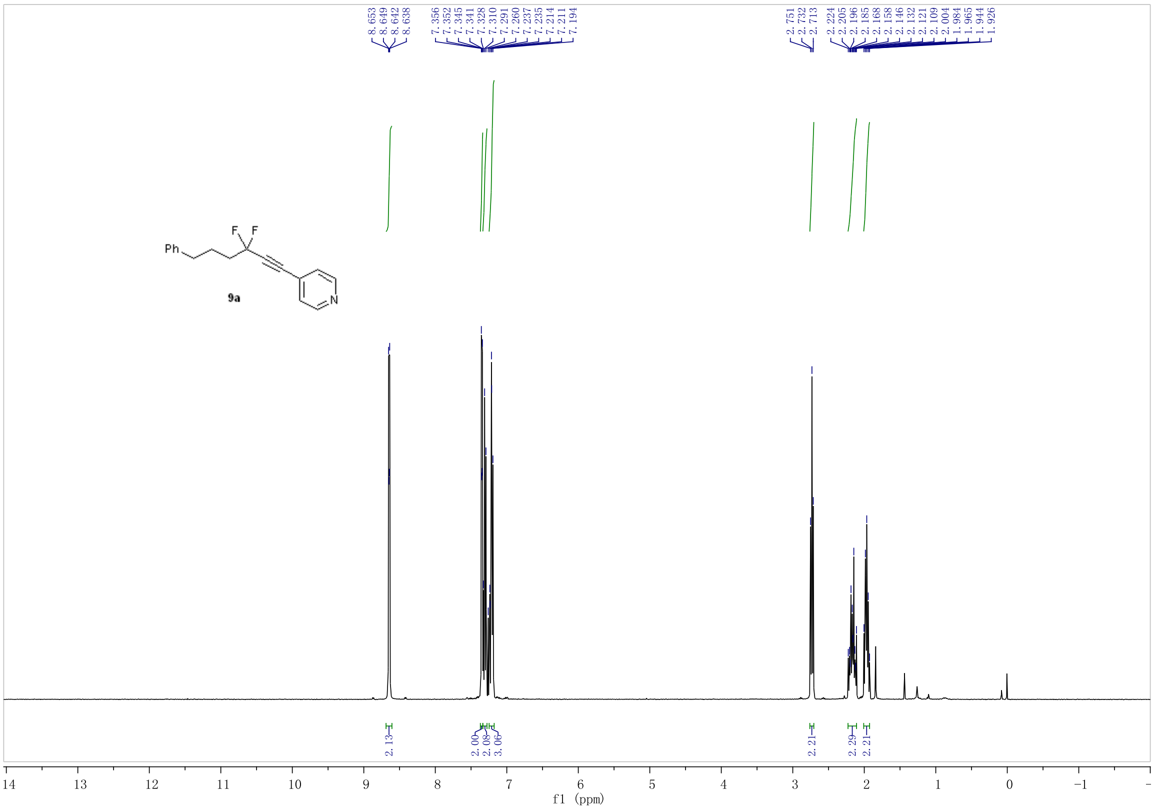

Supplementary Figure 140. ^{1}H NMR of 4-(3,3-difluoro-6-phenylhex-1-yn-1-yl)pyridine (9a)

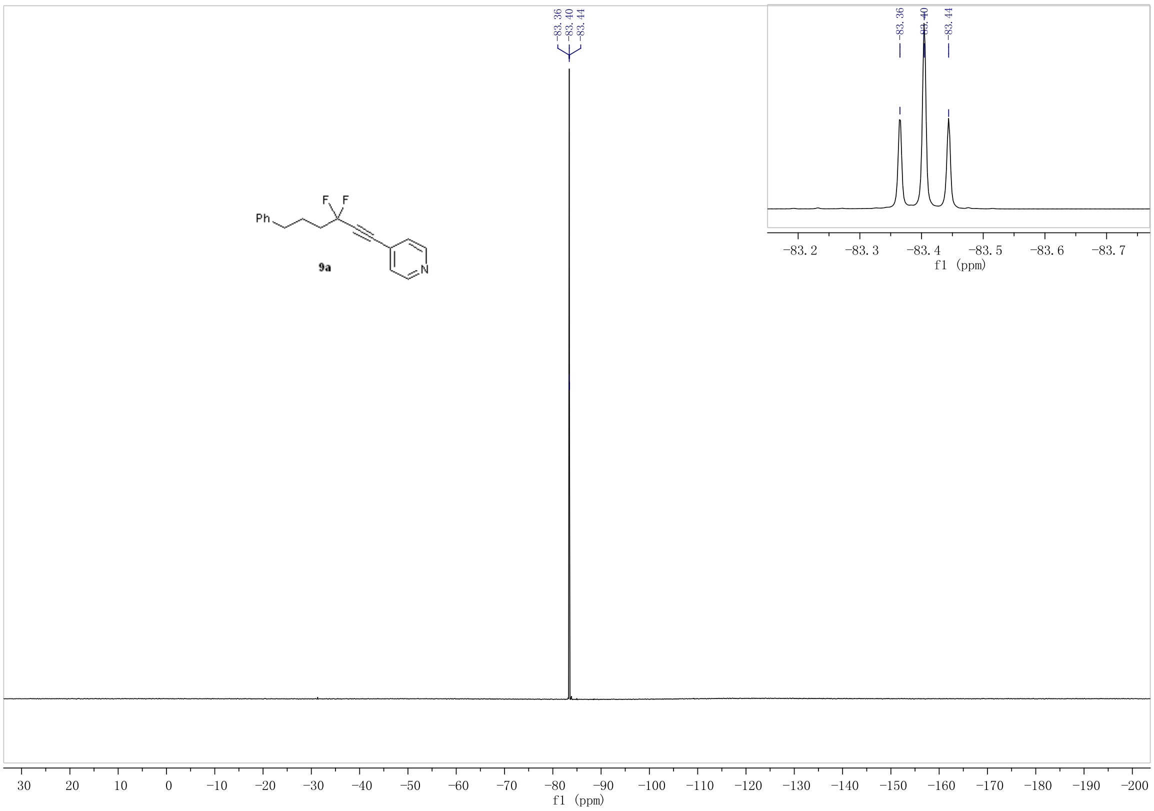

Supplementary Figure 141. ^{19}F NMR of 4-(3,3-difluoro-6-phenylhex-1-yn-1-yl)pyridine (9a)

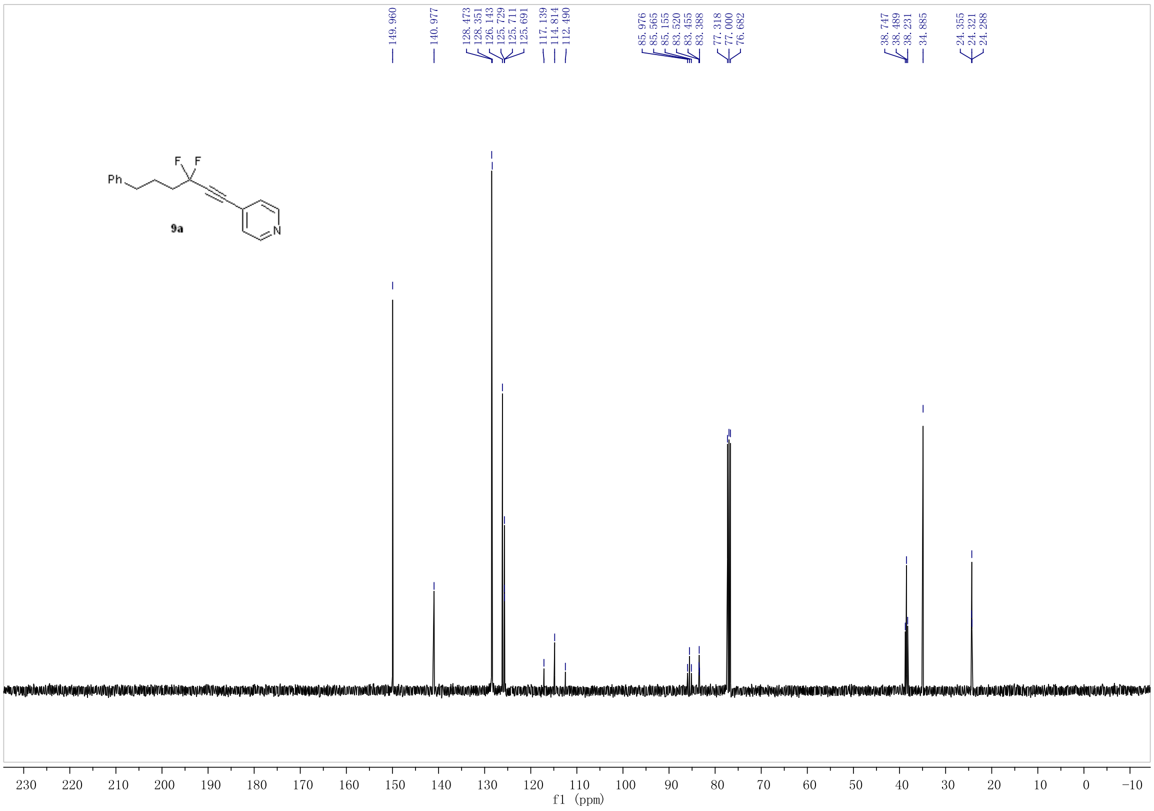

Supplementary Figure 142. ^{13}C NMR of 4-(3,3-difluoro-6-phenylhex-1-yn-1-yl)pyridine (9a)

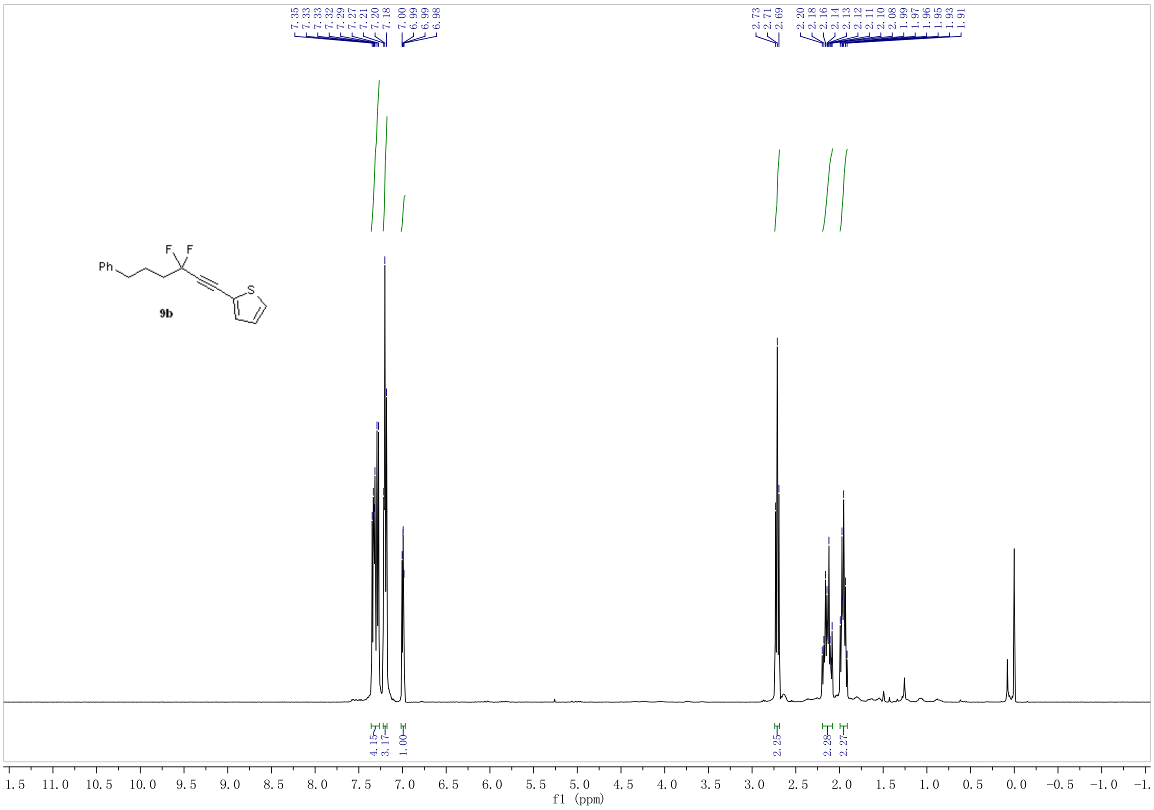

Supplementary Figure 143. ^{1}H NMR of 2-(3,3-difluoro-6-phenylhex-1-yn-1-yl)thiophene (9b)

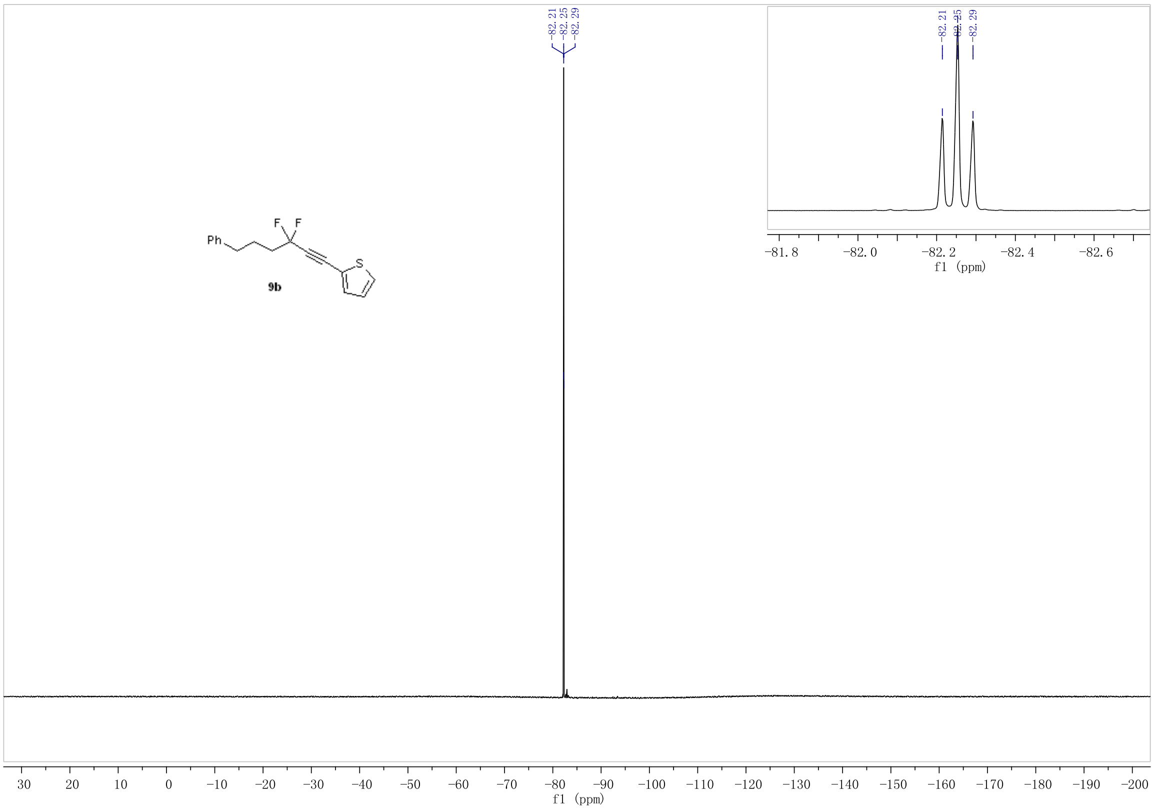

Supplementary Figure 144. ^{19}F NMR of 2-(3,3-difluoro-6-phenylhex-1-yn-1-yl)thiophene (9b)

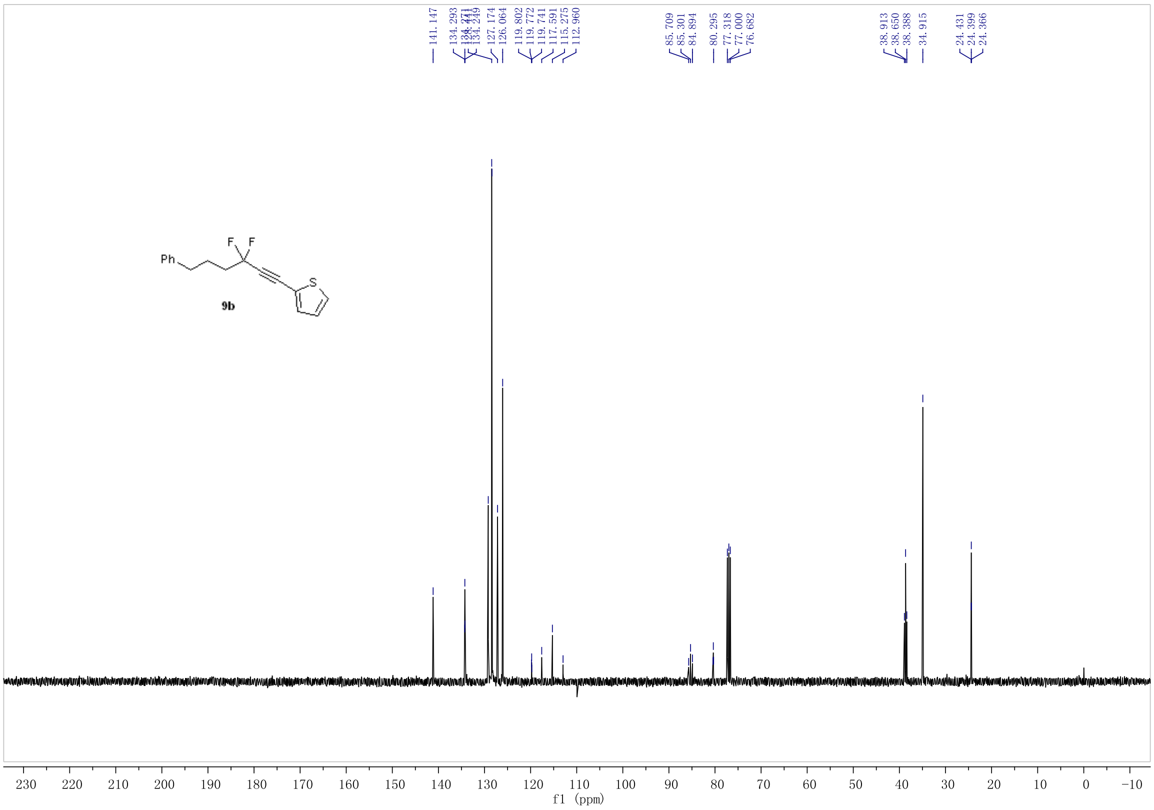

Supplementary Figure 145. ^{13}C NMR of 2-(3,3-difluoro-6-phenylhex-1-yn-1-yl)thiophene (9b)

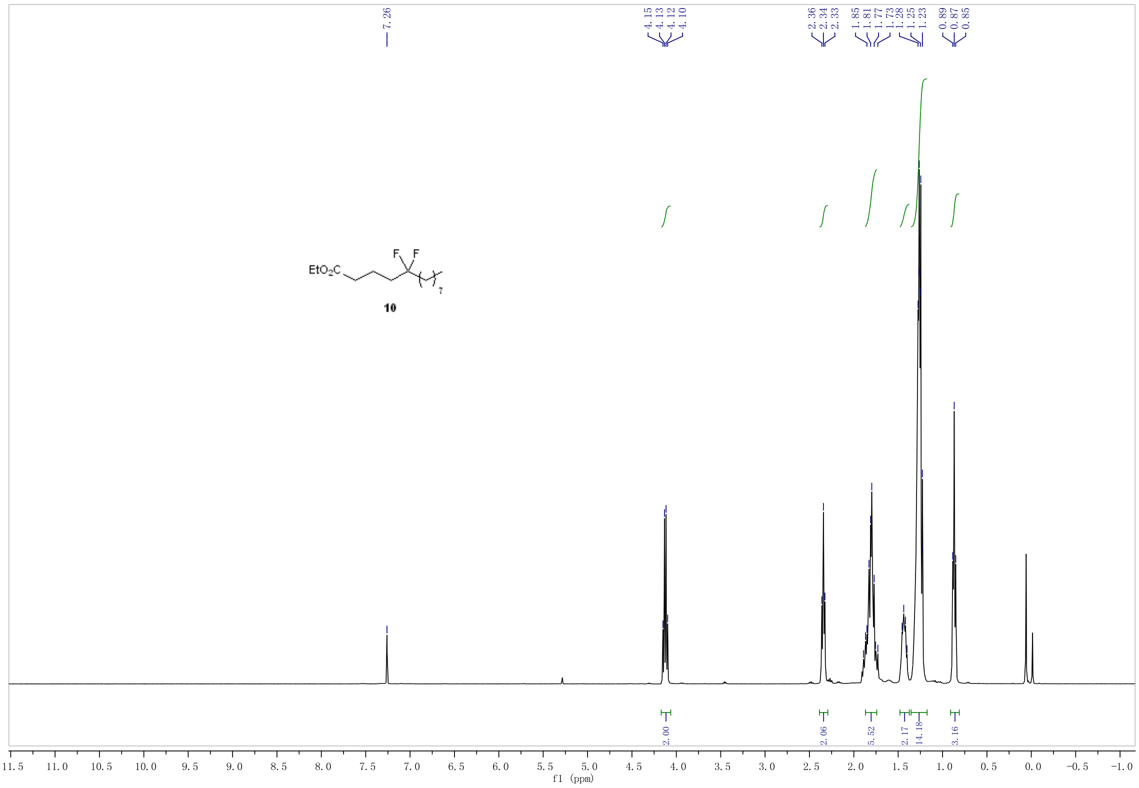

Supplementary Figure 146. ^{1}H NMR of ethyl 5,5-difluorotridecanoate (10)

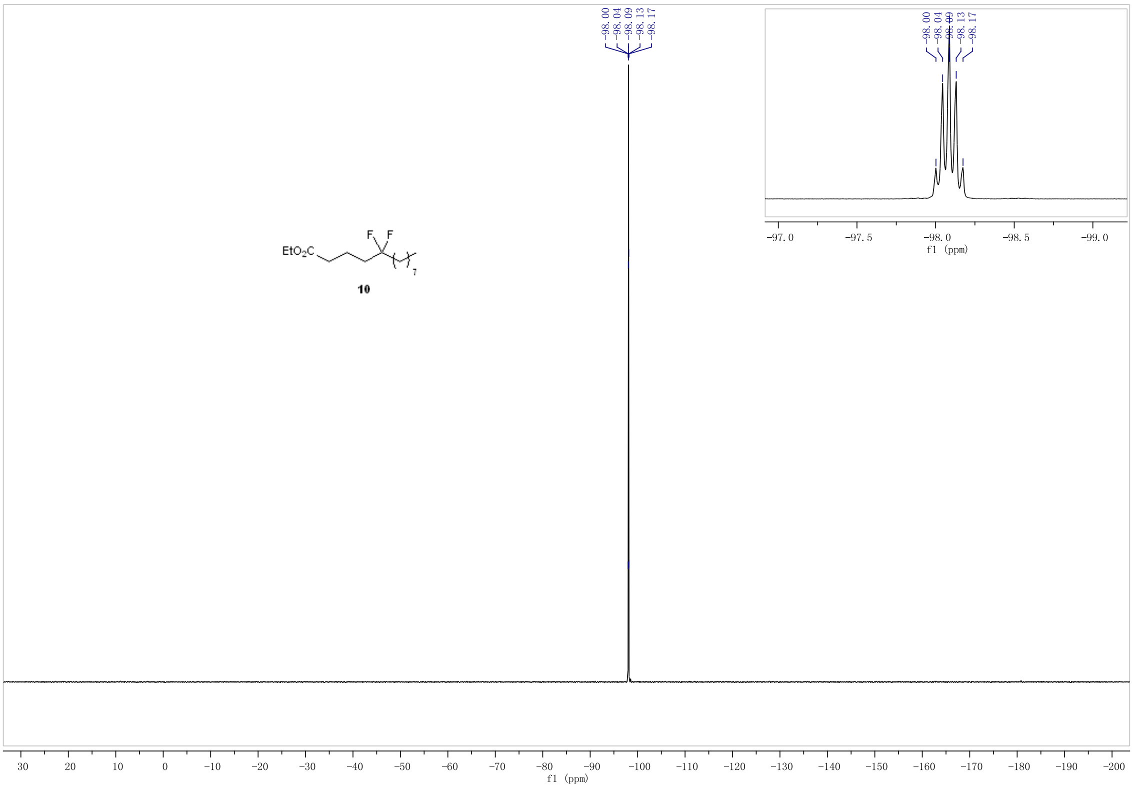

Supplementary Figure 147. ^{19}F NMR of ethyl 5,5-difluorotridecanoate (10)

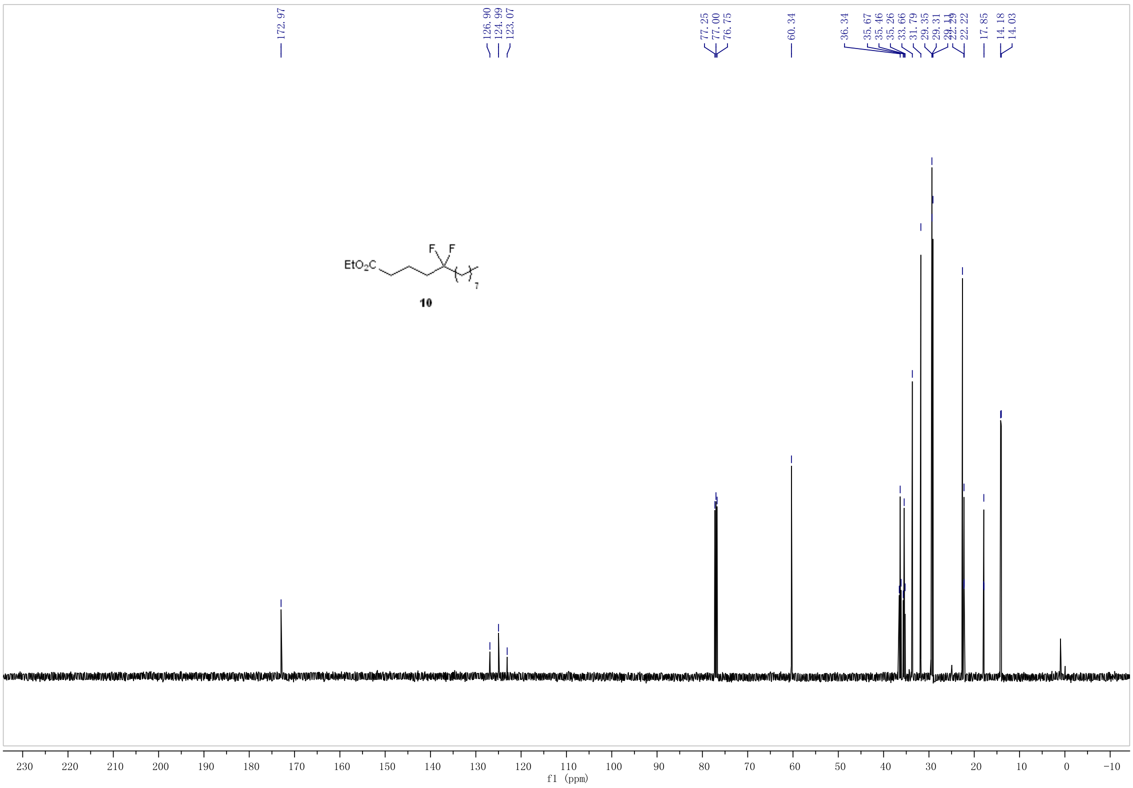

Supplementary Figure 148. ^{13}C NMR of ethyl 5,5-difluorotridecanoate (10)

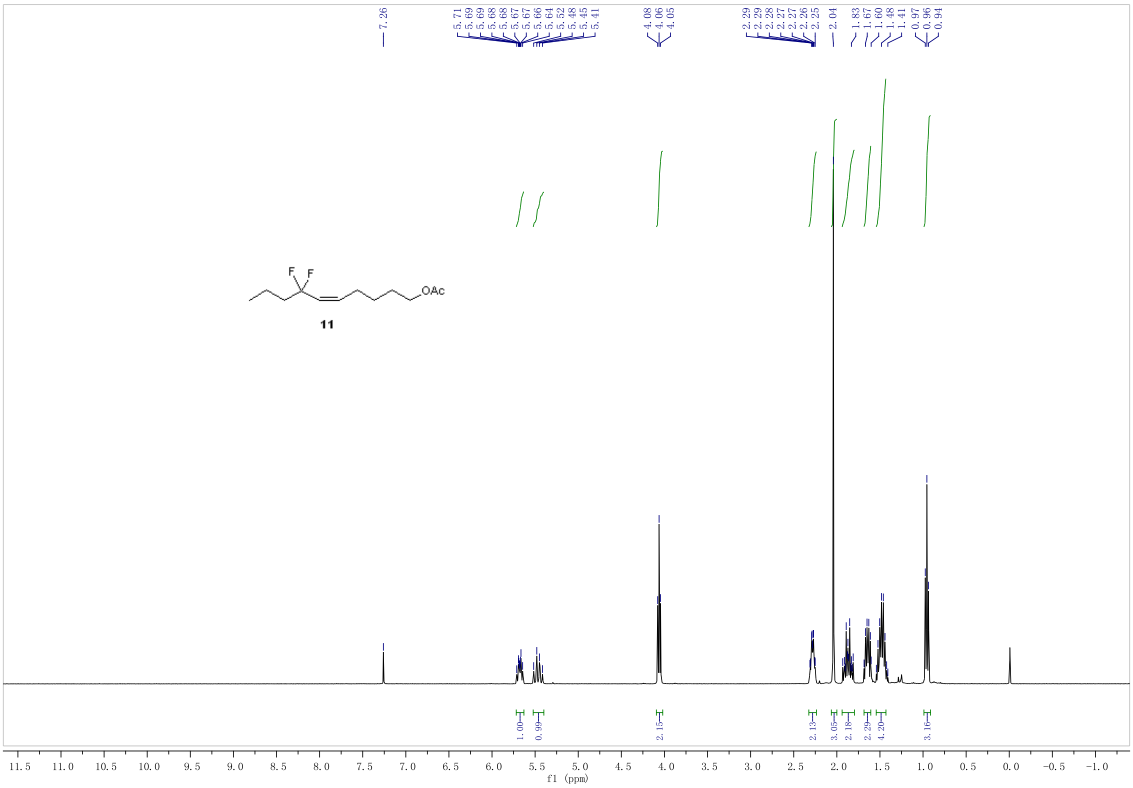

Supplementary Figure 149. ¹H NMR of (Z)-7,7-difluorodec-5-en-1-yl acetate (11)

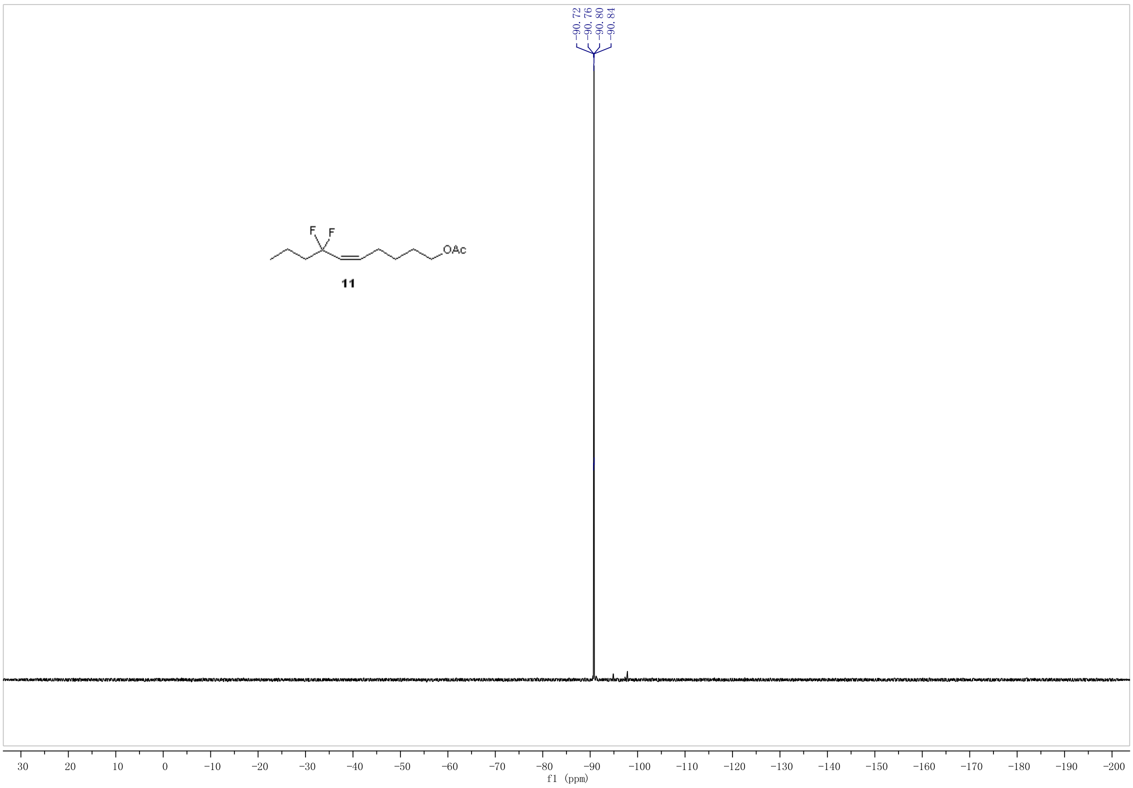

Supplementary Figure 150. ¹⁹F NMR of (Z)-7,7-difluorodec-5-en-1-yl acetate (11)

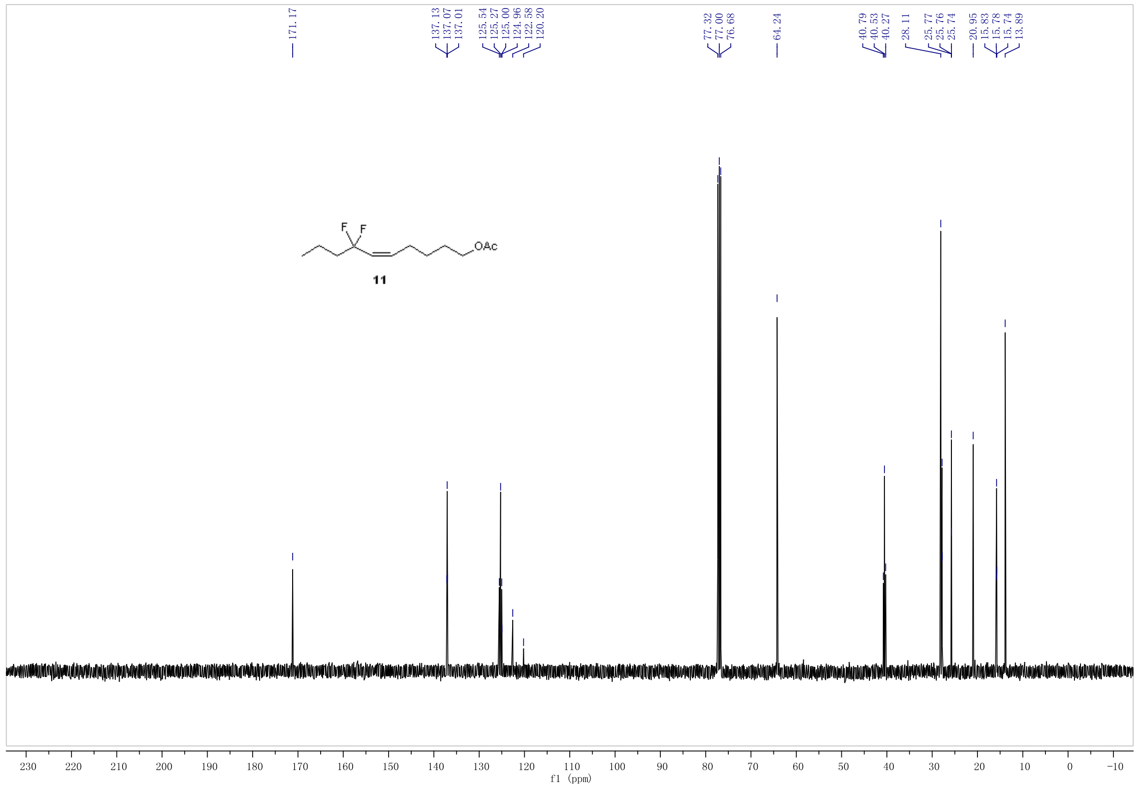

Supplementary Figure 151. ^{13}C NMR of (Z)-7,7-difluorodec-5-en-1-yl acetate (11)

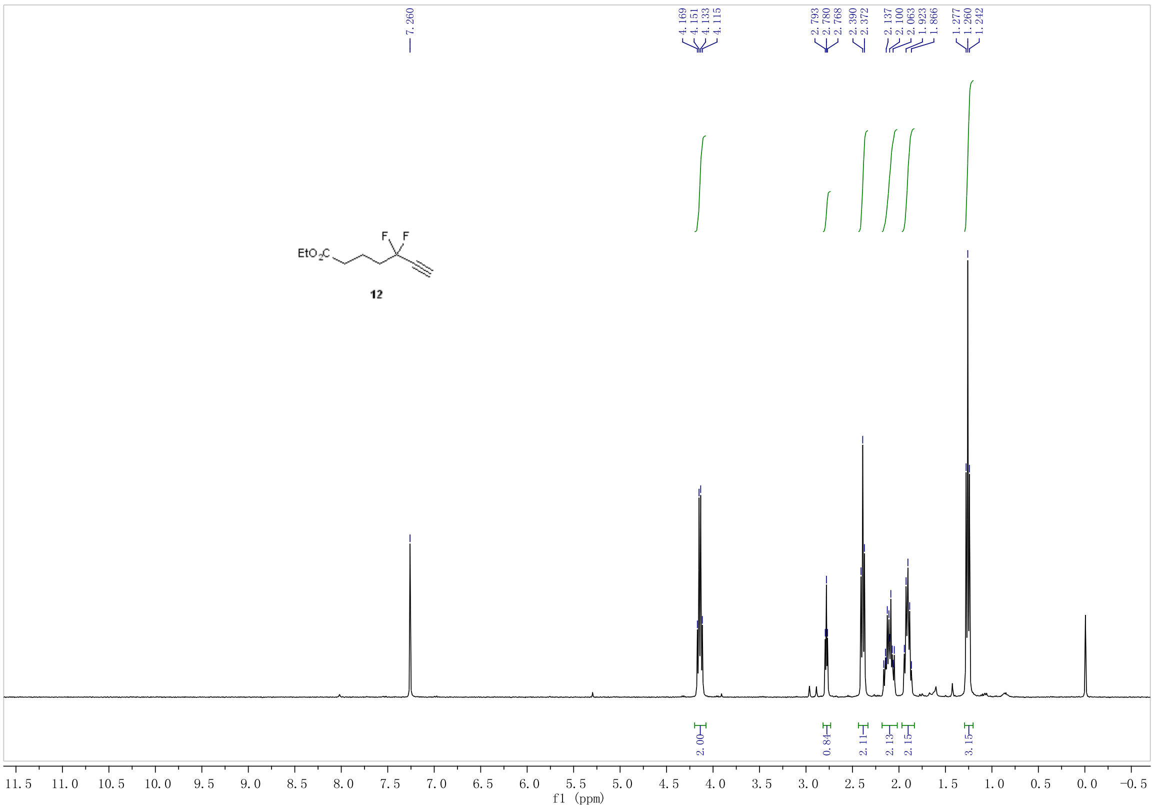

Supplementary Figure 152. ^{1}H NMR of ethyl 5,5-difluorohept-6-ynoate (12)

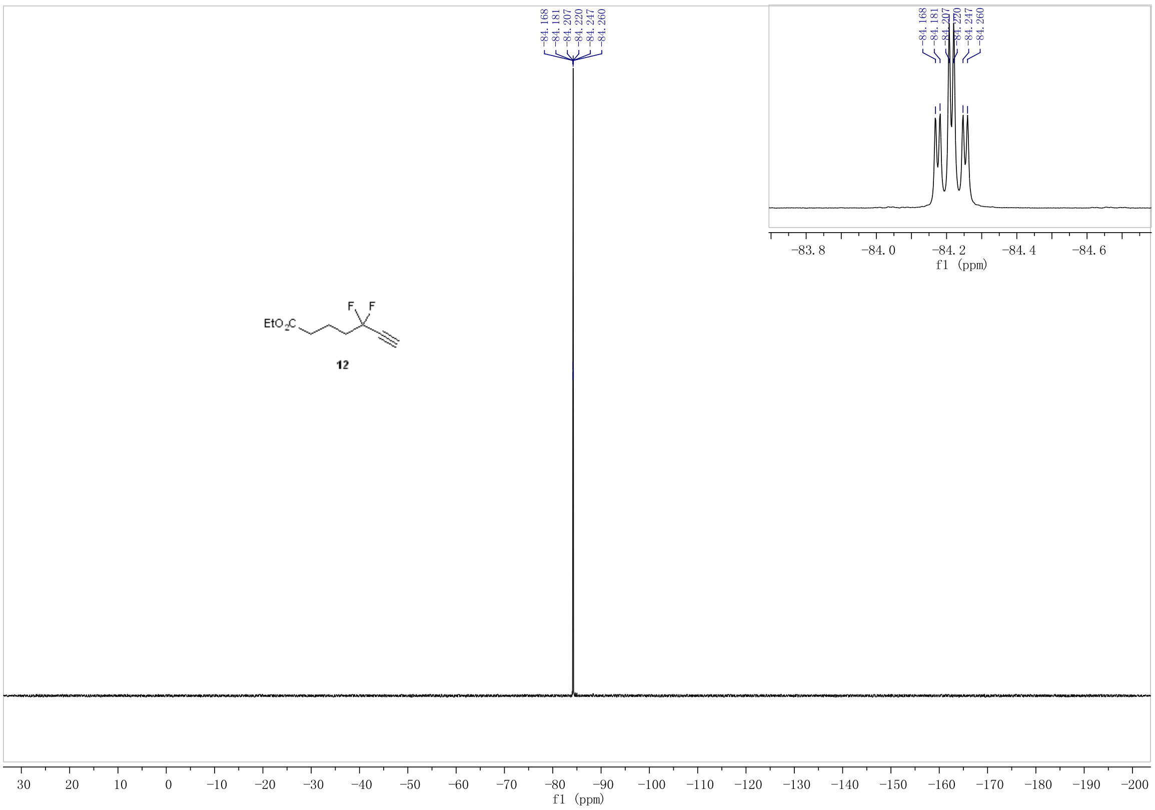

Supplementary Figure 153. ^{19}F NMR of ethyl 5,5-difluorohept-6-ynoate (12)

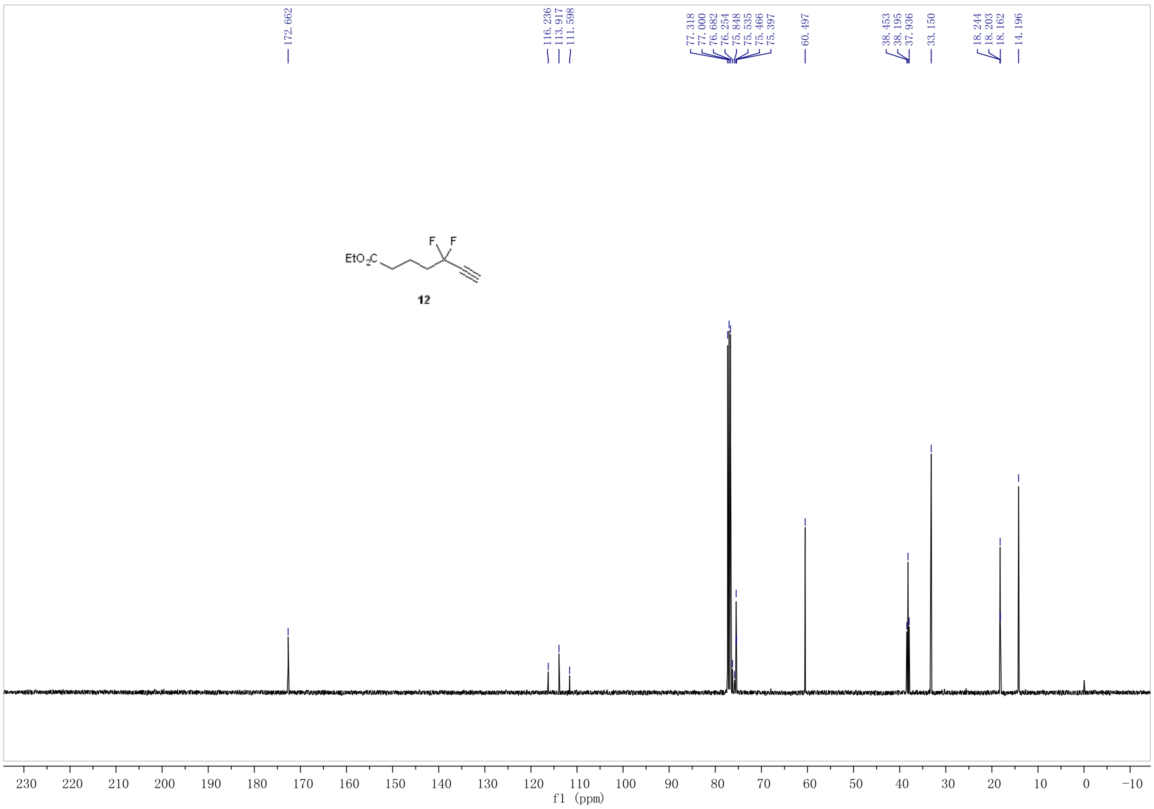

Supplementary Figure 154. ^{13}C NMR of ethyl 5,5-difluorohept-6-ynoate (12)

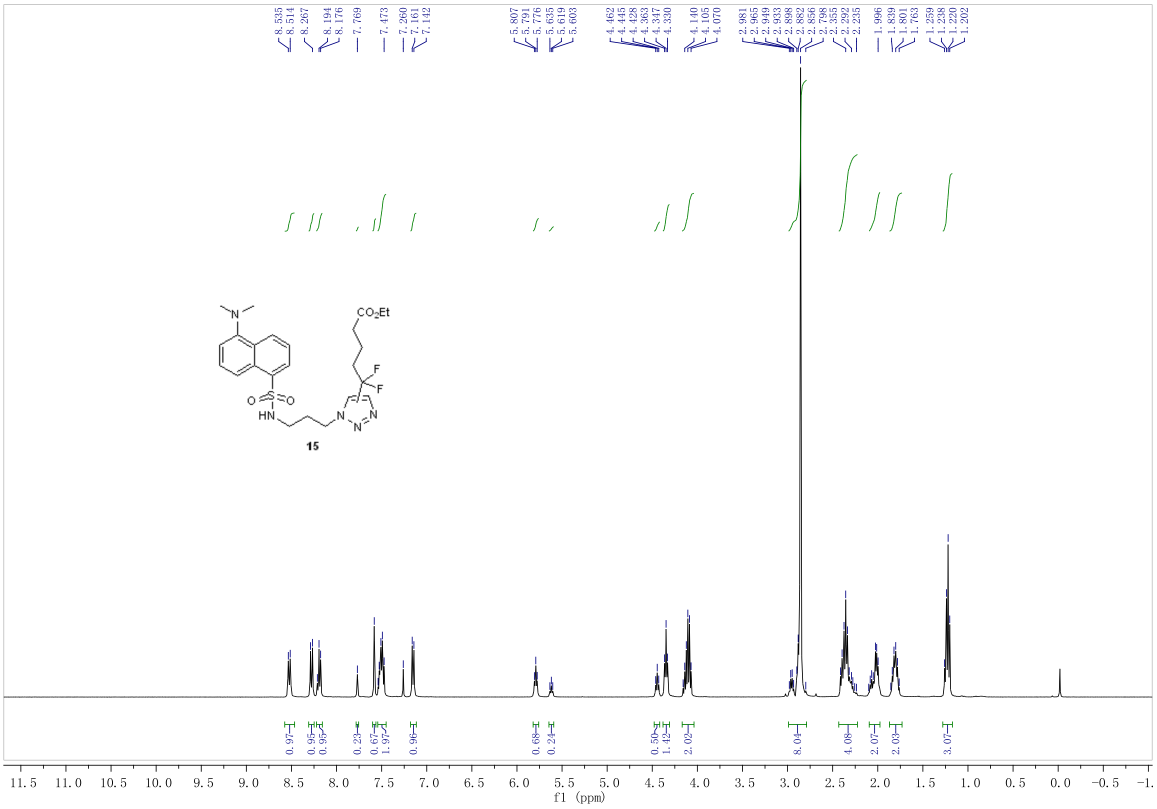

Supplementary Figure 155. ^{1}H NMR of product 15

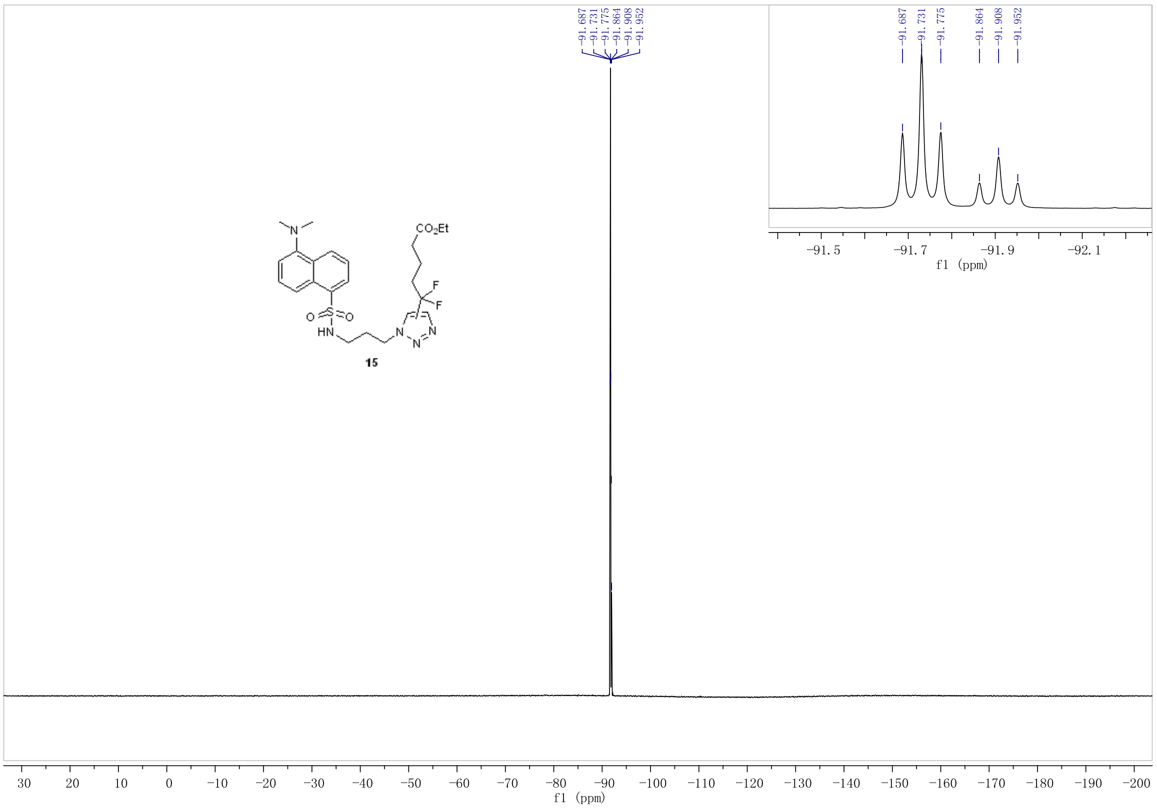

Supplementary Figure 156. ^{19}F NMR of product 15

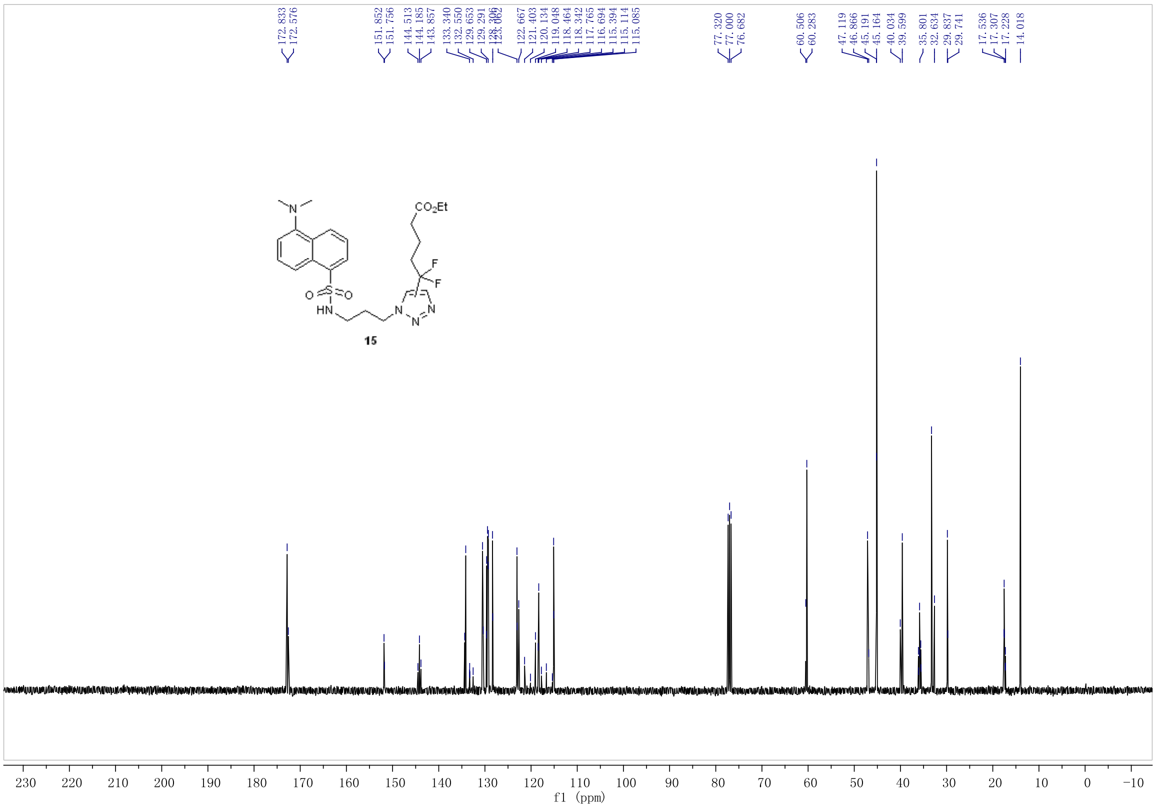

Supplementary Figure 157. ^{13}C NMR of product 15

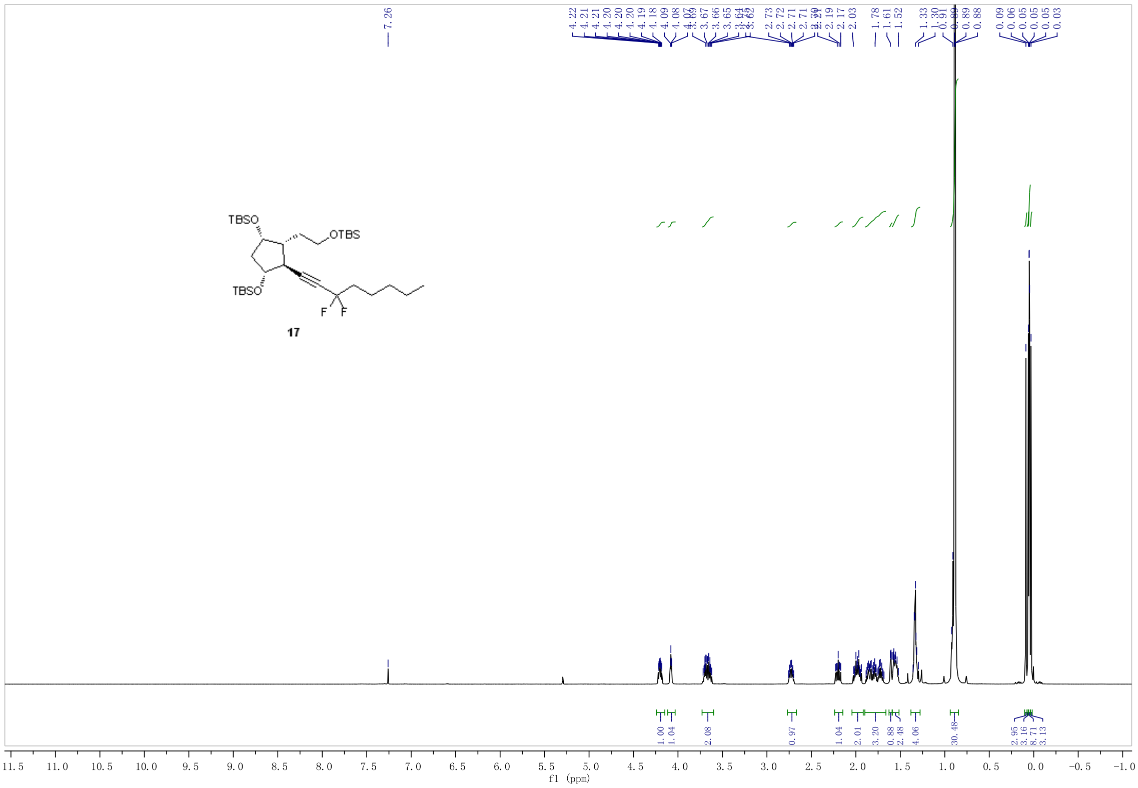

Supplementary Figure 158. ^{1}H NMR of product 17

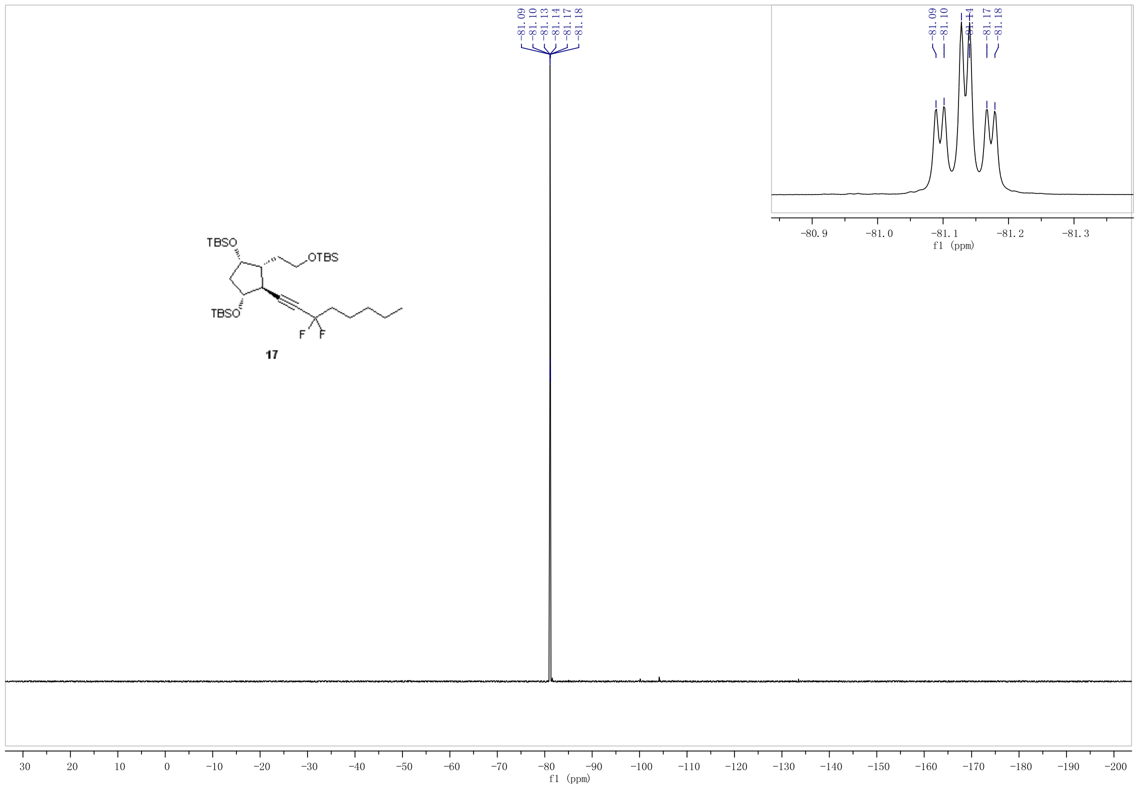

Supplementary Figure 159. ^{19}F NMR of product 17

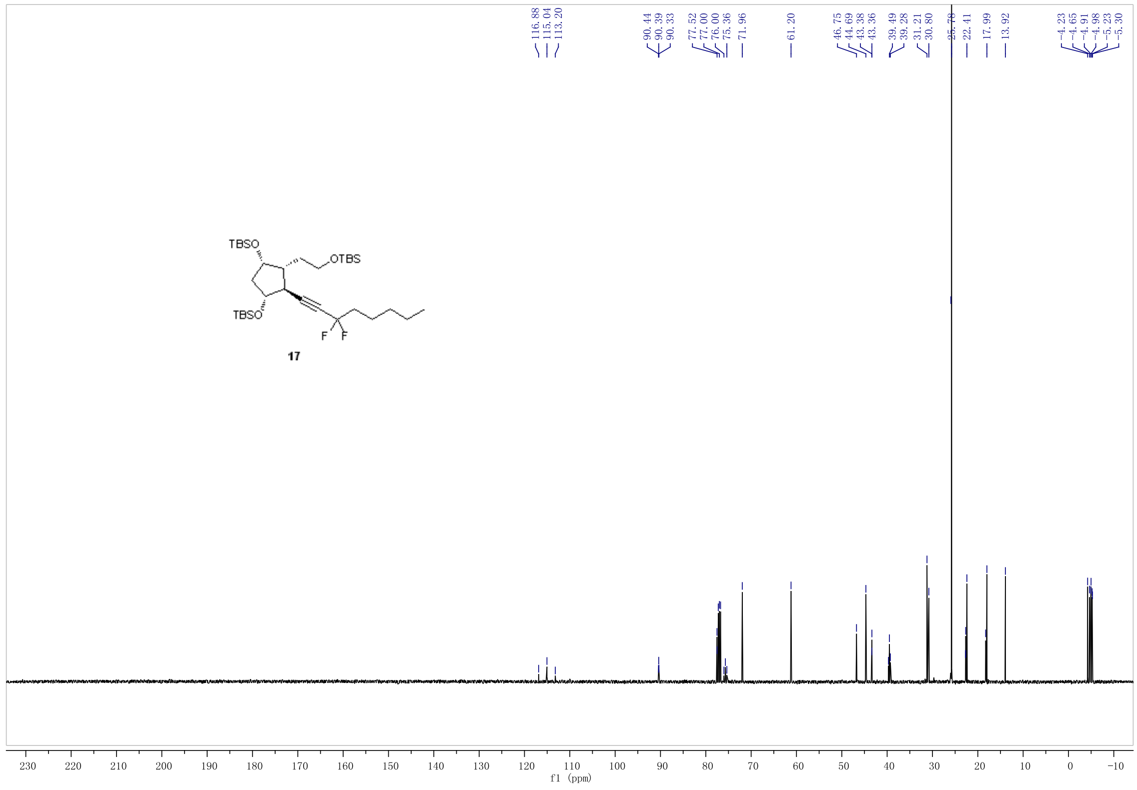

Supplementary Figure 160. ^{13}C NMR of product 17

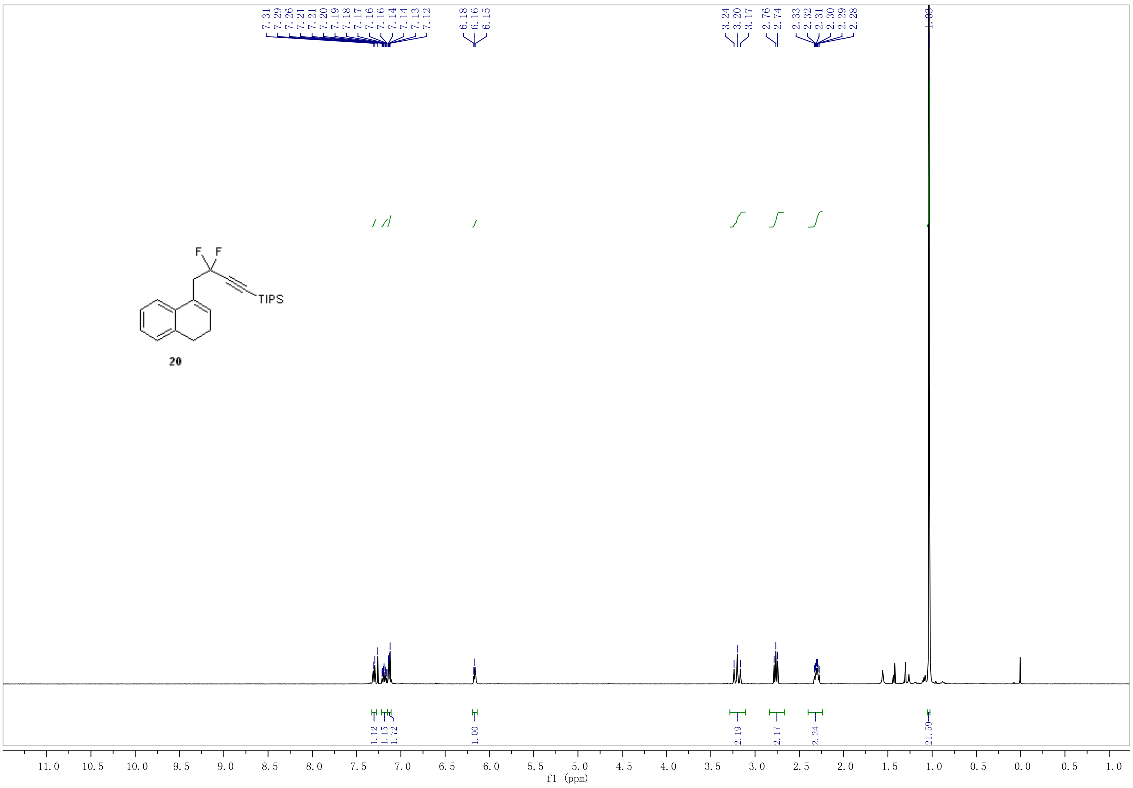

Supplementary Figure 161. ¹H NMR of (4-(3,4-dihydronaphthalen-1-yl)-3,3-difluorobut-1-yn-1-yl)triisopropylsilane (20)

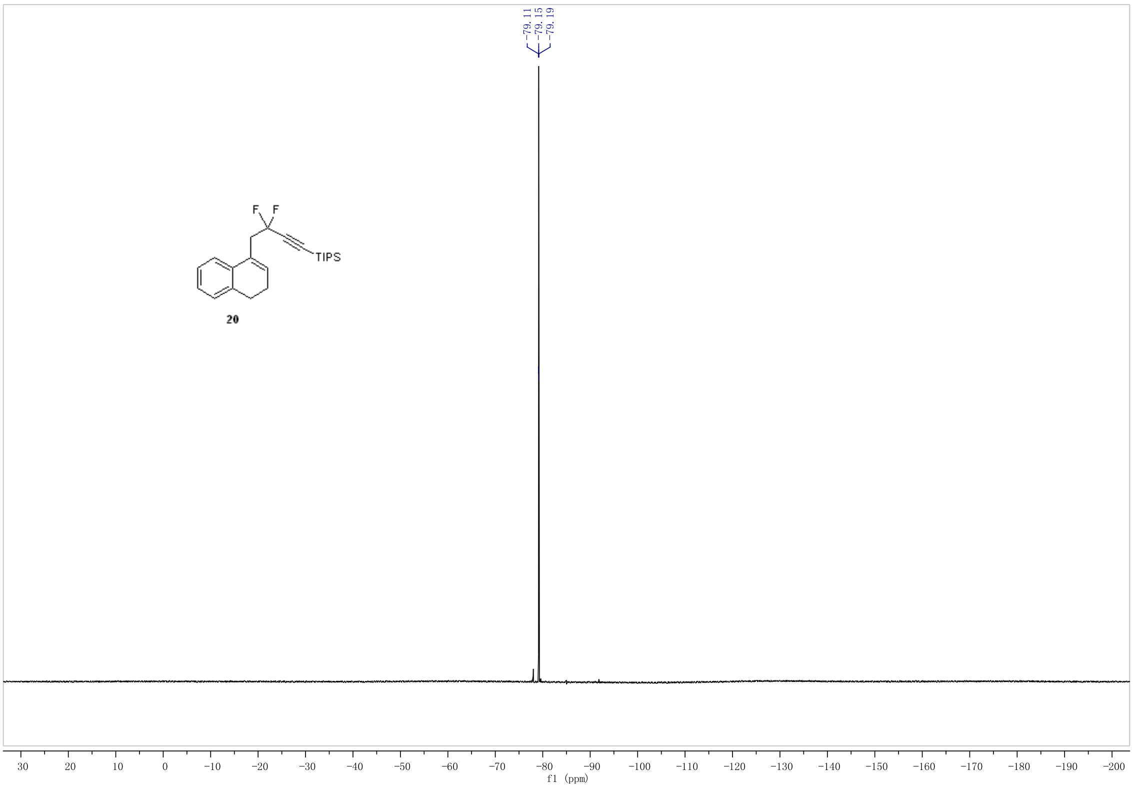

Supplementary Figure 162. ¹⁹F NMR of (4-(3,4-dihydronaphthalen-1-yl)-3,3-difluorobut-1-yn-1-yl)triisopropylsilane (20)

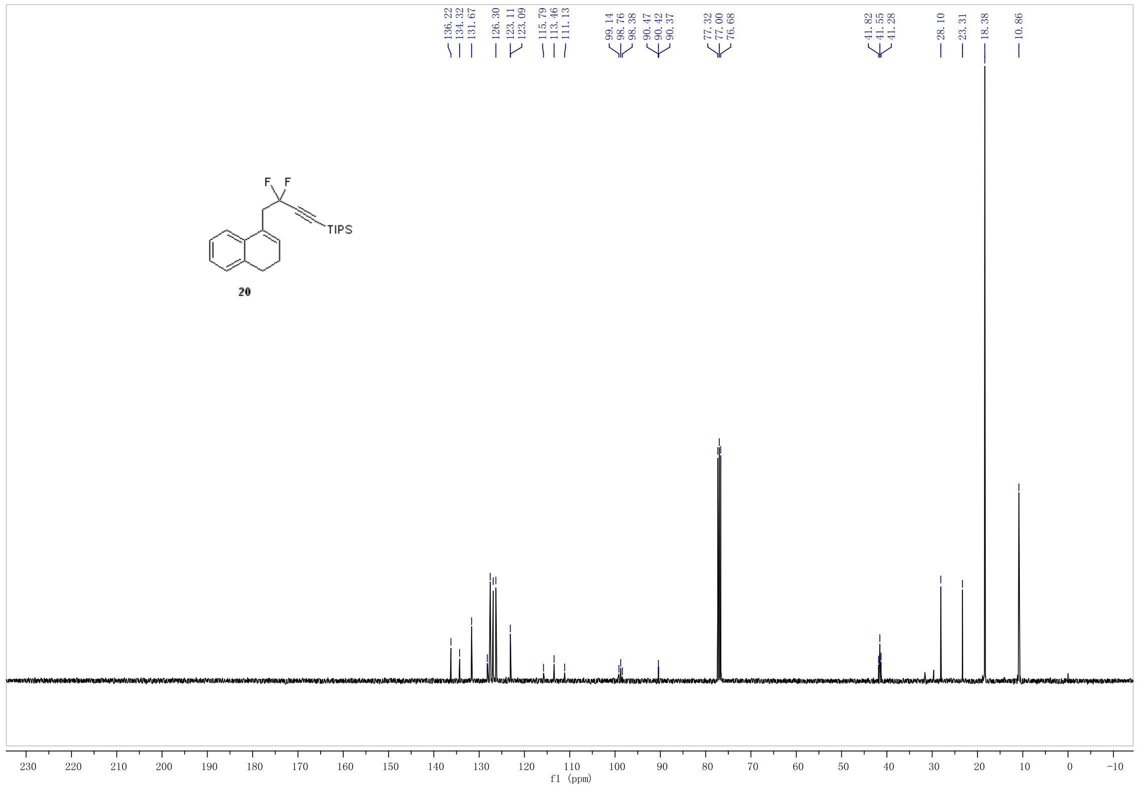

Supplementary Figure 163. ^{13}**C NMR of (4-(3,4-dihydronaphthalen-1-yl)-3,3-difluorobut-1-yn-1-yl)triisopropylsilane (20)**

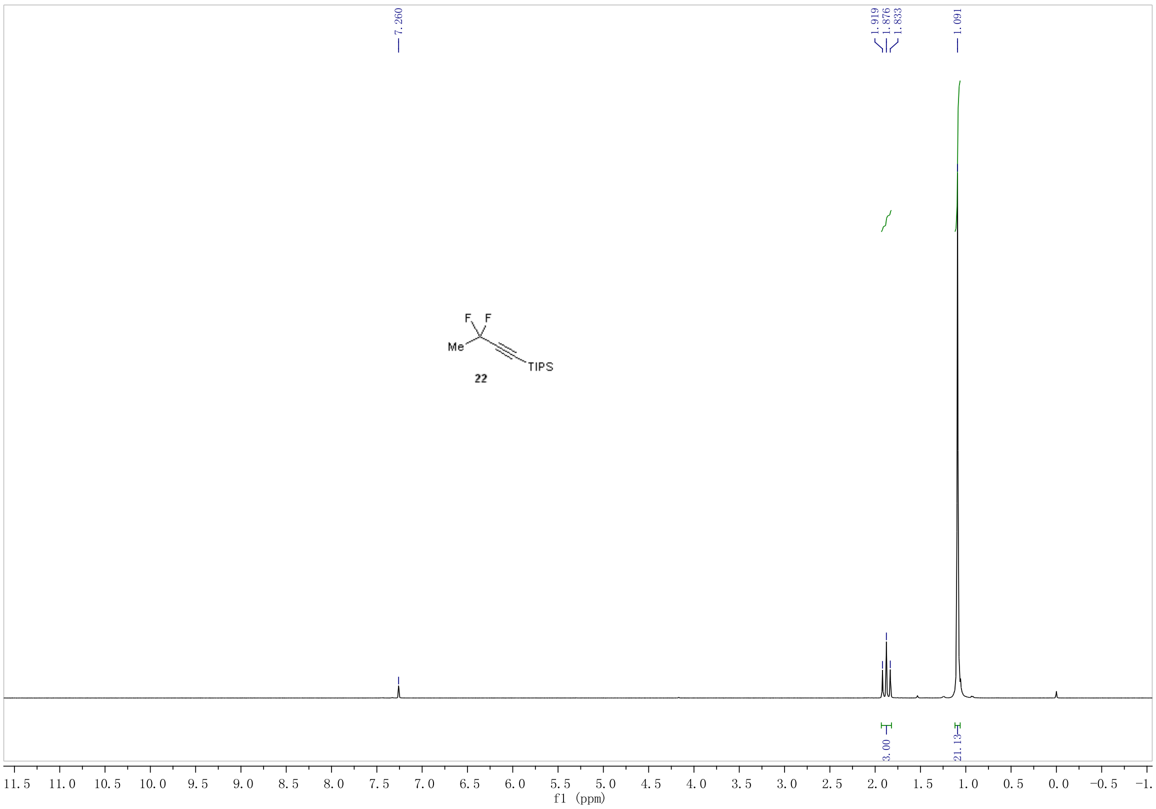

Supplementary Figure 164. ^{1}**H NMR of (3,3-difluorobut-1-yn-1-yl)triisopropylsilane (22)**

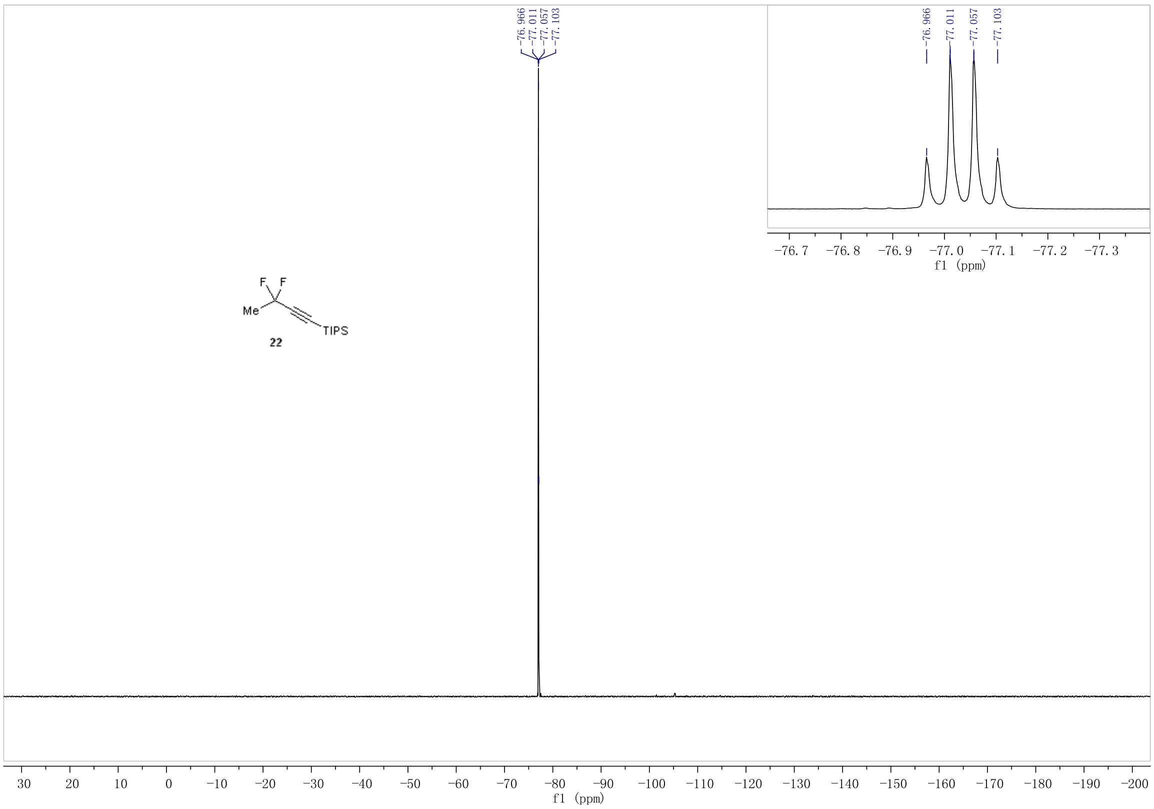

Supplementary Figure 165. ^{19}F NMR of (3,3-difluorobut-1-yn-1-yl)triisopropylsilane (22)

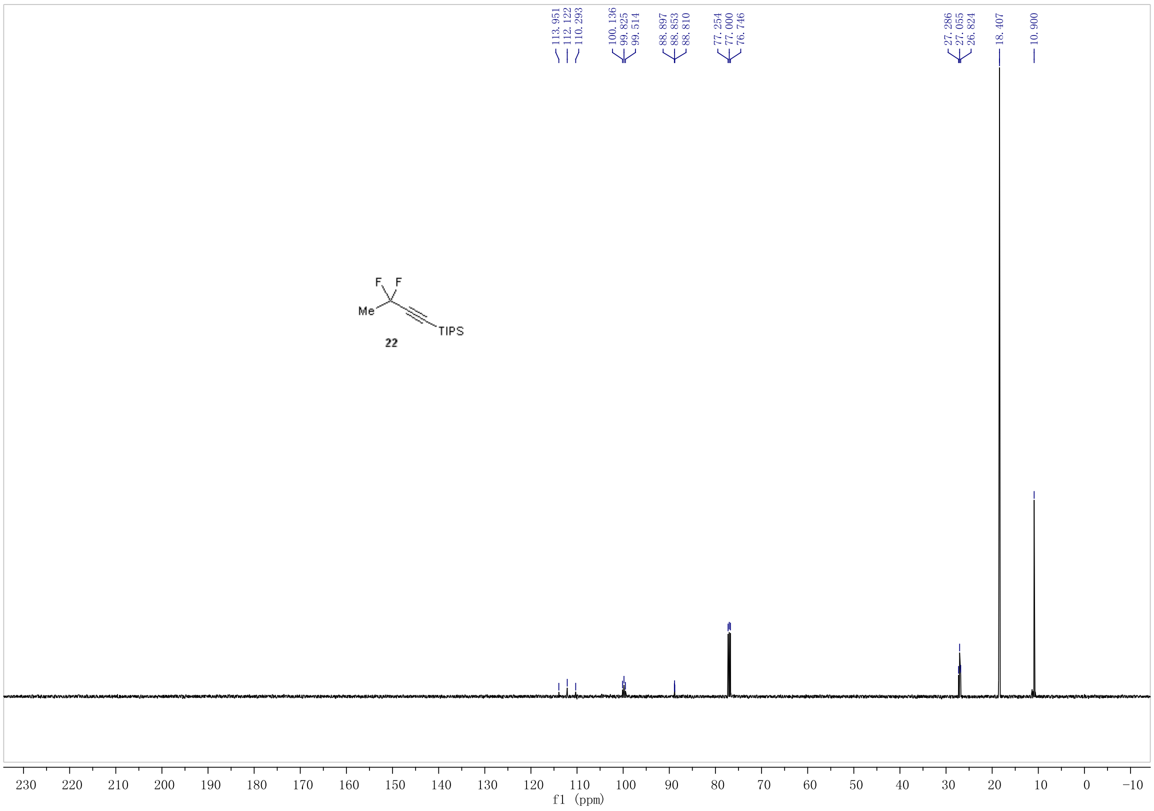

Supplementary Figure 166. ^{13}C NMR of (3,3-difluorobut-1-yn-1-yl)triisopropylsilane (22)

Supplementary Table 1 Optimization of nickel catalysts and ligands [a]

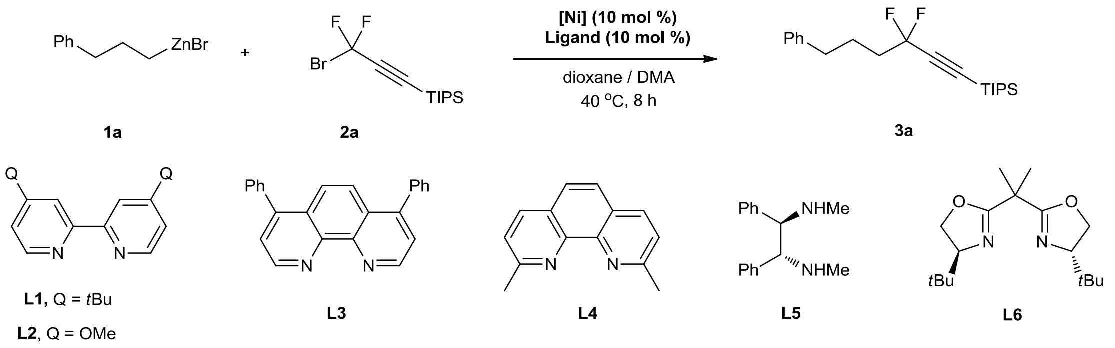

Entry	[Ni]	L	**3a** ,Yield (%)[b]	Entry	[Ni]	L	**3a** ,Yield (%)[b]
1	$NiCl_2 \cdot DME$	bpy	27	10	$NiCl_2$	Tpy	89
2	$NiCl_2 \cdot DME$	phen	24	11	$NiBr_2 \cdot$diglyme	Tpy	78
3	$NiCl_2 \cdot DME$	**L1**	18	12	$NiBr_2 \cdot DME$	Tpy	98
4	$NiCl_2 \cdot DME$	**L2**	22	13	$NiCl_2 \cdot 6H_2O$	Tpy	75
5	$NiCl_2 \cdot DME$	**L3**	6	14	$Ni(NO_3)_2 \cdot 6H_2O$	Tpy	73
6	$NiCl_2 \cdot DME$	**L4**	5	15	$Ni(PPh_3)Cl_2$	Tpy	91
7	$NiCl_2 \cdot DME$	**L5**	2	**16**	**Ni(dppf)Cl₂**	**Tpy**	**99**
8	$NiCl_2 \cdot DME$	**L6**	12	17	$Ni(dppb)Cl_2$	Tpy	95
9	$NiCl_2 \cdot DME$	Tpy	82	18	$Ni(COD)_2$	Tpy	30

[a]Reaction conditions (unless otherwise specified): **1a** (0.45 mmol, 1.5 equiv in 1 mL of DMA), **2a** (0.3 mmol, 1.0 equiv), dioxane (2 mL). [b]Determined by ^{19}F NMR using fluorobenzene as an internal standard.

Supplementary Table 2 Optimization of loading amount of **1a**[a]

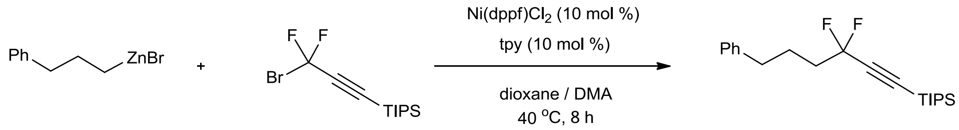

1a **2a** **3a**

Entry	**1a** equiv	**3a**, Yield (%)[b]
1	1.1	93
**2**	**1.2**	**99**
3	1.3	98
4	1.5	99

[a]Reaction conditions (unless otherwise specified): **1a** (1.1-1.5 equiv in 1 mL of DMA), **2a** (0.3 mmol, 1.0 equiv), dioxane (2 mL). [b]Determined by ^{19}F NMR using fluorobenzene as an internal standard.

Supplementary Table 3 Optimization of loading amount of Ni(dppf)Cl_2[a]

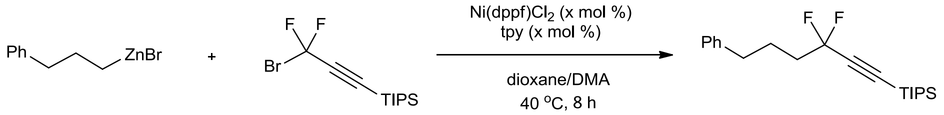

1a **2a** **3a**

Entry	x	**3a**, Yield (%)[b]
**1**	**2.5**	**99 (94)**
2	5	98
3	7.5	99
4	10	99

[a]Reaction conditions (unless otherwise specified): **1a** (0.36 mmol, 1.2 equiv in 1 mL of DMA), **2a** (0.3 mmol, 1.0 equiv), dioxane (2 mL). [b]Determined by ^{19}F NMR using fluorobenzene as an internal standard and number in parenthesis is isolated yield.

Supplementary Methods

General Information

^{1}H NMR and ^{13}C NMR spectra were recorded on a Bruker AM 400, Agilent MR 400 and Agilent MR 500 spectrometer. ^{19}F NMR was recorded on an Agilent MR 400 spectrometer ($CFCl_3$ as an external standard and low field is positive). Chemical shifts (δ) are reported in ppm, and coupling constants (*J*) are in Hertz (Hz). The following abbreviations were used to explain the multiplicities: s = singlet, d = doublet, t = triplet, q = quartet, m = multiplet, br = broad. NMR yield was determined by ^{19}F NMR using fluorobenzene as an internal standard before working up the reaction.

Materials

All reagents were used as received from commercial sources. Alkyl bromides were used as received from commercial sources without further purification, znic powder was activated by washing with 3% (v/v) HCl aqueous solution for 1 minutes and dried in vacuum. DMA was distilled under reduced pressure from CaH_2. THF, toluene, diethyl ether and 1,4-dioxane was distilled from sodium immediately before use.

Preparation of Alkylzinc Reagents

A 25-mL of Schlenk tube was charged with zinc powder (295 mg, 4.5 mmol) and heated to 80 ^oC under vacuum for 30 min. After the tube was back-filled with argon and cooled to room temperature, iodine (38 mg, 0.15 mmol) and DMA (3 mL) were added. The resulting mixture was stirred until the brown color disappeared, alkyl bromide (3 mmol) was added. The reaction mixture was heated to 80 ℃. After stirring for 10 h at 80 ℃, the mixture was cooled to room temperature. The gray solution was filtered and the filtrate was stored under argon in a Schlenk tube, the solution of the alkylzinc reagent was titrated with I_2 according to Knochel's method.[1] This alkylzinc solution can be stored at room temperature under argon for several weeks without deterioration.

General Procedure for the Preparation of *gem*-Difluoropropargyl Bromides 2

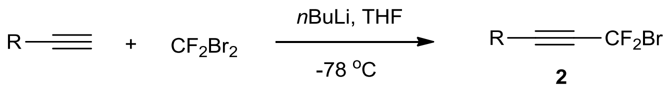

The preparation of compound **2** is according to the literature.[5] The preparation of (3-bromo-3,3-difluoroprop-1-yn-1-yl)triisopropylsilane **2a** as an example: To a solution of (triisoproylsilyl)acetylene (10.95 g, 60.0 mmol) in anhydrous THF (125 mL) was added dropwise *n*BuLi (2.5 M in hexane; 26.4 mL, 66 mmol) under an Ar atmosphere at -78 ℃. After stirring for 60 min at -78 ℃, the reaction mixture was cooled to -196 ℃ (liquid N_2 bath). CF_2Br_2 (26.5 g, 126 mmol) was added to the mixture via cannulation. After the addition was complete, the mixture was allowed to warm to room temperature very slowly and stirred overnight. The reaction mixture was then quenched with sat. aq NH_4Cl and the solvent THF was evaporated under reduced pressure. The residue was extracted with *n*-Pentane (3 × 150 mL) and the combined organic layers were washed

with H_2O (50 mL), dried over Na_2SO_4, and concentrated. The residue was purified with silica gel chromatography (Petroleum ether) to give a brown oil, which was distilled under reduced pressure (70 °C /1.1 mmHg) to give the pure product **2a** (14.0 g, 75% yield) as a colorless oil.

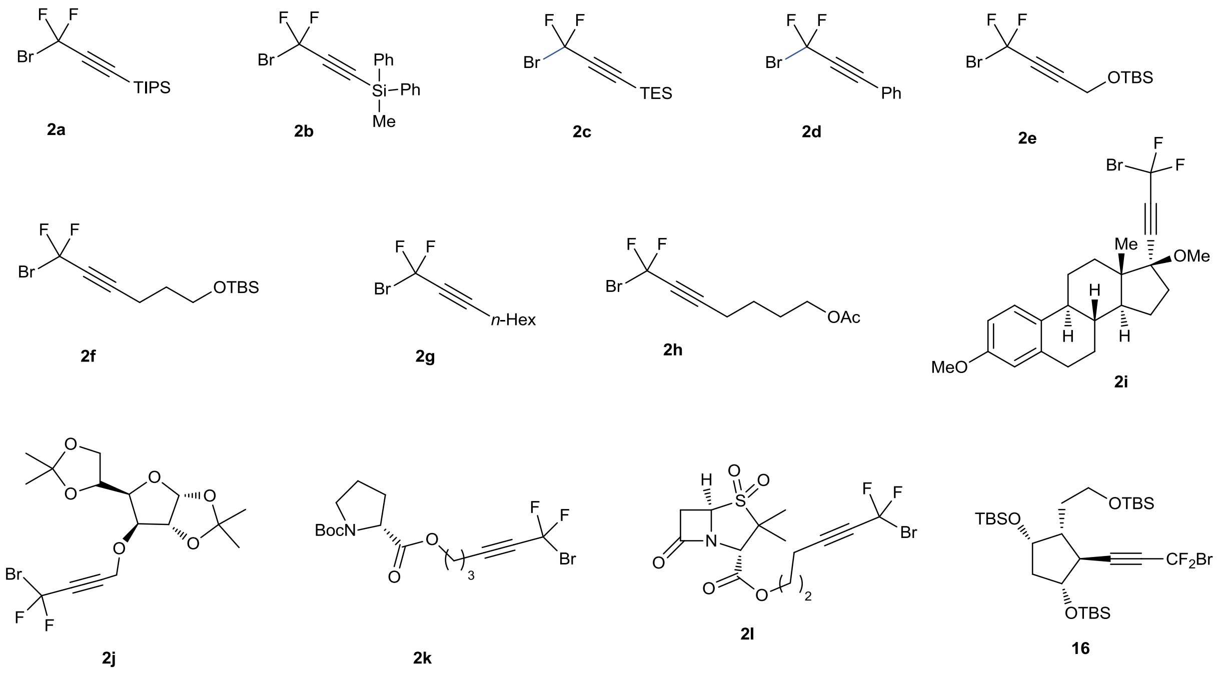

Supplementary Figure 167. Preparation of *gem*-Difluoropropargyl Bromides

Compounds **2a**[5], **2c**[5], **2d**[5], **2e**[6] and **2g**[5] are prepared according to the literature.

(3-Bromo-3,3-difluoroprop-1-yn-1-yl)(methyl)diphenylsilane (2b)

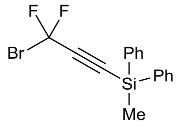

The product (85% yield) was purified with silica gel chromatography (Petroleum ether) as a yellow oil. ^{1}H NMR (400 MHz, $CDCl_3$) δ 7.68 – 7.58 (m, 4 H), 7.52 – 7.39 (m, 6 H), 0.86 – 0.71 (m, 3 H). ^{13}C NMR (101 MHz, $CDCl_3$) δ 134.5, 132.4, 130.4, 128.3, 100.7 (t, *J* = 290.5 Hz,), 97.2 (t, *J* = 37.6 Hz,), 93.9 (t, *J* = 4.9 Hz,), -2.9. ^{19}F NMR (376 MHz, $CDCl_3$) δ -33.4 – -33.5 (m, 2 F). IR (thin film) ν_{max} 3071, 2193, 1429, 1181, 950, 727 cm^{-1}. MS (DART): m/z (%) 370, 368 (100, M + NH_4^+), 349, 347. HRMS: Calculated for $C_{16}H_{17}NBrF_2Si$: 368.0276; Found: 368.0276.

((6-Bromo-6,6-difluorohex-4-yn-1-yl)oxy)(*tert*-butyl)dimethylsilane (2f)

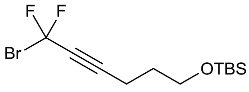

The product (70% yield) was purified with silica gel chromatography (Petroleum ether/EtOAc = 100:1) as a colorless oil. ^{1}H NMR (500 MHz, $CDCl_3$) δ 3.69 (t, *J* = 5.8 Hz, 2 H), 2.49 – 2.43 (m, 2 H), 1.81 – 7.74 (m, 2 H), 0.90 (s, 9 H), 0.06 (s, 6 H). ^{13}C NMR (126 MHz, $CDCl_3$) δ 101.4 (t, *J* = 288.7 Hz), 92.8 (t, *J* = 5.7 Hz), 74.0 (t, *J* = 37.7 Hz), 60.9, 30.3, 25.9, 18.3, 15.0 (t, *J* = 1.9 Hz), -5.4. ^{19}F NMR (376 MHz, $CDCl_3$) δ -30.2 (t, *J* = 4.6 Hz). IR (thin film) ν_{max} 2930，2858，2262，1472，1239，1107 cm^{-1}. MS (DART): m/z (%) 327 (100), 326 (M + H^{+}), 309, 307. HRMS: Calculated for $C_{12}H_{22}O_2BrF_2Si$: 327.0591; Found: 327.0587.

((7-Bromo-7,7-difluorohept-5-yn-1-yl)oxy)(*tert*-butyl)dimethylsilane (2h′)

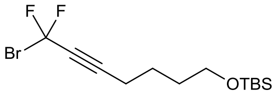

The product (9.65 g, 85% yield) was purified with silica gel chromatography (Petroleum ether) as a colorless oil. ^{1}H NMR (400 MHz, $CDCl_3$) δ 3.64 (t, *J* = 5.8 Hz, 2 H), 2.43 – 2.36 (m, 2 H), 1.73 – 1.53 (m, 2 H), 0.89 (s, 9 H), 0.05 (s, 6 H). ^{13}C NMR (126 MHz, $CDCl_3$) δ 101.4 (t, *J* = 288.7 Hz), 92.9 (t, *J* = 5.7 Hz), 74.1 (t, *J* = 37.7 Hz), 62.3, 31.7, 25.9, 23.9 (t, *J* = 1.6 Hz), 18.3 (t, *J* = 2.0 Hz), -5.3. ^{19}F NMR (376 MHz, $CDCl_3$) δ -30.10 (t, *J* = 4.7 Hz, 2 F). MS (DART): m/z (%) 343 (100), 341 (M+H^{+}), 293, 291, 270, 268, 214, 212. HRMS: Calculated for $C_{13}H_{24}OBrF_2Si$: 341.0742; Found: 341.0741.

Preparation of substrate 2h

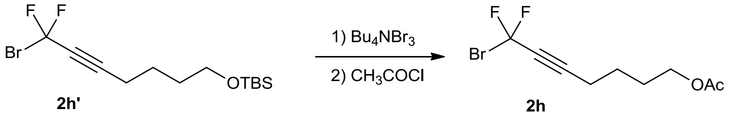

7-Bromo-7,7-difluorohept-5-yn-1-yl acetate (2h)

Step 1[7]: To a solution of tetrabutylammonium tribromide (TBATB) (1.12 g, 15 mol %) in methanol (60 mL) was added a solution of 2h′ (5.3 g, 15.5 mmol) in methanol (20 mL) at room temperature.

The reaction was monitored by TLC. When compound **2h′** was consumed, the solvent was removed and the residue was redissolved in ethyl acetate. The organic layer was washed with saturated sodium thiosulfate solution (10 mL), followed by water (2 x 20 mL). The organic layer was then dried over anhydrous Na_2SO_4, filtered, and concentrated. The crude product was directly used in the next step without purification. **Step 2:** A 250 mL roundbottomed flask was charged with the crude alcohol (3.2 g, 14.1 mmol, 1.0 equiv), trimethylamine (2.0 g, 1.4 equiv), 60 mL of DCM, and was cooled with an ice bath. To the solution was slowly added acetyl chloride. After stirring for 6 hours, the reaction was quenched with water, the organic layer was separated, dried over Na_2SO_4, filtered, and concentrated. The product **2h** (3.3 g, 87% yield) was purified with silica gel chromatography (Petroleum ether/EtOAc = 30:1) as a colorless oil. ^{1}H NMR (500 MHz, $CDCl_3$) δ 4.06 (t, *J* = 6.2 Hz, 2 H), 2.38 (tt, *J* = 6.9, 4.6 Hz, 2 H), 2.02 (s, 3 H), 1.75 – 1.69 (m, 2 H), 1.68 – 1.60 (m, 2 H). ^{13}C NMR (126 MHz, $CDCl_3$) δ 101.3 (t, *J* = 288.7 Hz), 92.1 (t, *J* = 5.7 Hz), 74.2 (t, *J* = 37.8 Hz), 63.4, 27.6, 23.8 (t, *J* = 1.6 Hz), 20.8, 18.1 (t, *J* = 1.9 Hz). ^{19}F NMR (376 MHz, $CDCl_3$) δ -30.5 (t, *J* = 4.6 Hz, 2 F). MS (DART): m/z (%) 288 (100), 286 ($M+NH_4^+$), 271, 269, 251, 249. HRMS: Calculated for $C_9H_{12}O_2BrF_2$: 268.9983; Found: 268.9982.

(8*R*,9*S*,13*S*,14*S*,17*S*)-17-(3-Bromo-3,3-difluoroprop-1-yn-1-yl)-3,17-dimethoxy-13-methyl-7,8,9, 11,12,13,14,15,16,17-decahydro-6H-cyclopenta[a]phenanthrene (2i)

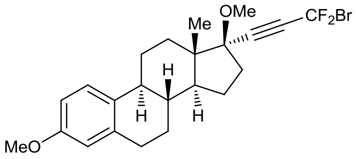

The product (1.50 g, 83% yield) was purified with silica gel chromatography (Petroleum ether/EtOAc = 100:1) as a white solid, m.p. 74 – 75 °C. ^{1}H NMR (400 MHz, $CDCl_3$) δ 7.22 (dd, *J* = 8.5, 2.4 Hz, 1 H), 6.76 – 6.70 (m, 1 H), 6.64 (s, 1 H), 3.80 (s, 3 H), 3.44 (s, 3 H), 2.87 (d, *J* = 3.5 Hz, 2 H), 2.43 – 2.20 (m, 3 H), 2.10 – 2.00 (m, 1 H), 1.93 – 1.80 (m, 4 H), 1.71 – 1.60 (m, 1 H), 1.58 – 1.33 (m, 4 H), 0.92 (s, 3 H). ^{13}C NMR (101 MHz, $CDCl_3$) δ 157.4, 137.8, 132.1, 126.3, 113.8, 111.5, 101.3 (t, *J* = 289.7 Hz), 93.1 (t, *J* = 6.0 Hz), 85.6 (t, *J* = 1.4 Hz), 80.5 (t, *J* = 38.3 Hz), 55.2, 53.8, 50.0, 48.4, 43.4, 39.1, 35.7, 34.1, 29.7, 27.2, 26.4, 22.8, 12.7. ^{19}F NMR (376 MHz, $CDCl_3$) δ -32.1 – -31.0 (m). IR (thin film) ν_{max} 3030, 2867, 2242, 1609, 1465, 1235, 982, 882 cm^{-1}. MS (EI): m/z (%) 454, 452 (M^+), 373, 227 (100), 147. HRMS: Calculated for $C_{23}H_{27}O_2F_2Br$: 452.1162; Found:

452.1161.

(3*aR*,5*R*,6*S*,6*aR*)-6-((4-Bromo-4,4-difluorobut-2-yn-1-yl)oxy)-5-((*R*)-2,2-dimethyl-1,3-dioxolan-4-yl)-2,2-dimethyltetrahydrofuro[2,3-*d*][1,3]dioxole (2j)

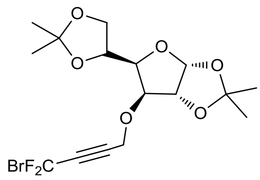

The product (1.28 g, 75% yield) was purified with silica gel chromatography (Petroleum ether/EtOAc = 10:1) as a pale yellow oil. ^{1}H NMR (400 MHz, $CDCl_3$) δ 5.86 (d, *J* = 3.7 Hz, 1 H), 4.59 (d, *J* = 3.7 Hz, 1 H), 4.42 (t, *J* = 3.9 Hz, 2 H), 4.28 – 4.19 (m, 1 H), 4.11 – 4.06 (m, 2 H), 4.03 (d, *J* = 2.9 Hz, 1 H), 4.00 – 3.95 (m, 1 H), 1.49 (s, 3 H), 1.41 (s, 3 H), 1.33 (s, 3 H), 1.30 (s, 3 H). ^{13}C NMR (101 MHz, $CDCl_3$) δ 112.0, 109.2, 105.2, 101.1 (t, *J* = 290.1 Hz), 86.3 (t, *J* = 5.7 Hz), 82.6, 82.3, 80.9, 78.4 (t, *J* = 38.8 Hz), 72.2, 67.4, 57.5 (t, *J* = 1.6 Hz), 26.9, 26.7, 26.1, 25.2. ^{19}F NMR (376 MHz, $CDCl_3$) δ -33.3 (t, *J* = 3.8 Hz). IR (thin film) ν_{max} 2988, 2895, 2267, 1456, 1374, 1227, 1023, 847 cm^{-1}. MS (DART): m/z (%) 429 (100), 427 ($M+NH_4^+$), 396, 394. HRMS: Calculated for $C_{16}H_{22}O_6BrF_2$: 427.0562; Found: 427.0562.

Preparation of 2k

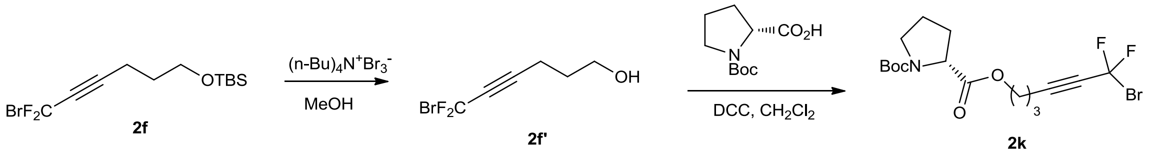

6-Bromo-6,6-difluorohex-4-yn-1-ol (2f′) [7]

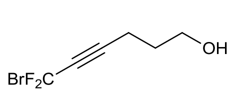

2f'

To a solution of tetrabutylammonium tribromide (TBATB) (1.76 g, 15 mol %) in methanol (100 mL) was added a solution of *gem*-difluoropropargyl bromide **2f** (8.0 g, 24.5 mmol) in methanol (30 mL) at room temperature. The reaction was monitored by TLC. When compound **2f** was consumed, the solvent was removed and the residue was redissolved in ethyl acetate. The organic layer was washed with saturated sodium thiosulfate solution (20 mL), followed by water (2 x 30 mL). The organic

layer was then dried over anhydrous Na_2SO_4, filtered, and concentrated. The residue was purified by column chromatography (petroleum/ethyl acetate=10:1) to afford alcohol **2f′** (3.13 g, 60 % yield). ^{1}H NMR (400 MHz, $CDCl_3$) δ 3.75 (t, *J* = 6.1 Hz, 2 H), 2.47 – 2.54 (m, 2 H), 1.89 – 1.78 (m, 2 H), 1.58 (s, 1 H). ^{13}C NMR (101 MHz, $CDCl_3$) δ 101.3 (t, *J* = 288.7 Hz), 92.2 (t, *J* = 5.7 Hz), 74.1 (t, *J* = 37.8 Hz), 60.8, 29.9, 15.0. ^{19}F NMR (376 MHz, $CDCl_3$) δ -30.4 (t, *J* = 4.6 Hz). IR (thin film) ν_{max} 3331, 2957, 2259, 1241, 1089, 1020 cm^{-1}. MS (DART): m/z (%) 195, 193. HRMS: Calculated for C_6H_7OBrF: 192.9659; Found: 192.9659.

2-(6-Bromo-6,6-difluorohex-4-yn-1-yl) 1-(*tert*-butyl) (*R*)-pyrrolidine-1,2-dicarboxylate (2k)

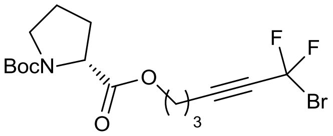

2k

To a 100 mL of round bottom flask were added DMAP (37 mg, 10 mol %), (*tert*-butoxycarbonyl)-*D*-proline (967 mg, 4.5 mmol), alcohol **2f′** (640 mg, 3.0 mmol) and dry CH_2Cl_2 (20 mL). The resulting mixture was then cooled to 0 ℃, a solution of dicyclohexylmethanediimine (DCC) (928 mg, 4.5 mmol) in CH_2Cl_2 (5 mL) was added dropwise. After stirring for 12 h at room temperature, the reaction mixture was filtered, the filtrate was concentrated. The crude product was purified by flash column chromatography (Petroleum ether/EtOAc = 4:1) to give compound **2k** (886 mg, 72%) as a colorless oil. ^{1}H NMR (400 MHz, $CDCl_3$) δ 4.29 – 4.11 (m, 3 H), 3.54 – 3.31 (m, 2 H), 2.49 – 2.39 (m, 2 H), 2.26 – 2.12 (m, 1 H), 1.97 – 1.79 (m, 5 H), 1.42 and 1.38 (s, 9 H, rotamer). ^{13}C NMR (101 MHz, $CDCl_3$) rotameric mixture, resonances for minor rotamer are enclosed in parenthesis: δ 173.0 (172.8), (154.3) 153.6, (101.2) 101.1 (t, *J* = 288.8 Hz), (91.5) 90.9 (t, *J* = 5.6 Hz), 79.9 (79.7), 74.5 (74.2) (t, *J* = 37.7 Hz), 62.8, 59.0 (58.8), (46.5) 46.2, 30.9, 29.9, (28.3) 28.2, (26.5) 26.4, 24.3, 23.5, 15.3 (15.2). ^{19}F NMR (376 MHz, $CDCl_3$) δ -30.7 and -30.9 (t, *J* = 3.8 Hz, rotamer). IR (thin film) ν_{max} 2977, 2882, 2259, 1748, 1698, 1399, 1240 cm^{-1}. MS (DART): m/z (%) 411, 410 (M + H$^+$, 100), 356, 354, 312, 310. HRMS: Calculated for $C_{16}H_{23}O_2N_4BrF_2Si$: 410.0774; Found: 410.0773.

6-Bromo-6,6-difluorohex-4-yn-1-yl-(2*S*,5*R*)-3,3-dimethyl-7-oxo-4-thia-1-azabicyclo[3.2.0]heptane-2-carboxylate 4,4-dioxide (2l)

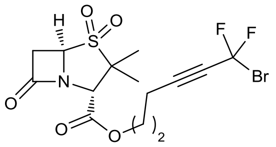

Compound **2l** was prepared according to the procedure for the preparation of **2k**. The product was purified by flash column chromatography (Petroleum ether/EtOAc = 3:1) to give **2l** (771 mg, 60%) as a colorless oil. ^{1}H NMR (400 MHz, $CDCl_3$) δ 4.61 (dd, *J* = 4.2, 2.1 Hz, 1 H), 4.38 (s, 1 H), 4.36 – 4.21 (m, 2 H), 3.49 (dd, *J* = 16.2, 4.3 Hz, 1 H), 3.43 (dd, *J* = 16.2, 2.1 Hz, 1 H), 2.53 – 2.46 (m, 2 H), 2.02 – 1.93 (m, 2 H), 1.60 (s, 3 H), 1.41 (s, 3 H). ^{13}C NMR (101 MHz, $CDCl_3$) δ 170.7, 166.8, 101.1 (t, *J* = 289.1 Hz), 90.3 (t, *J* = 5.6 Hz), 74.8 (t, *J* = 38.2 Hz), 64.3, 63.1, 62.6, 61.1, 38.3, 26.0 (t, *J* = 1.4 Hz), 20.3, 18.6, 15.3 .^{19}F NMR (376 MHz, $CDCl_3$) δ -31.2 (t, *J* = 4.4 Hz). IR (thin film) ν_{max} 2938, 2258, 1797, 1755, 1465, 1321, 1088, 857 cm^{-1}. MS (DART): m/z (%) 447, 445 ($M+NH_4^+$, 100), 430, 428 ($M+H^+$), 388, 386. HRMS: Calculated for $C_{14}H_{17}O_5NBrF_2S$: 427.9979; Found: 427.9976.

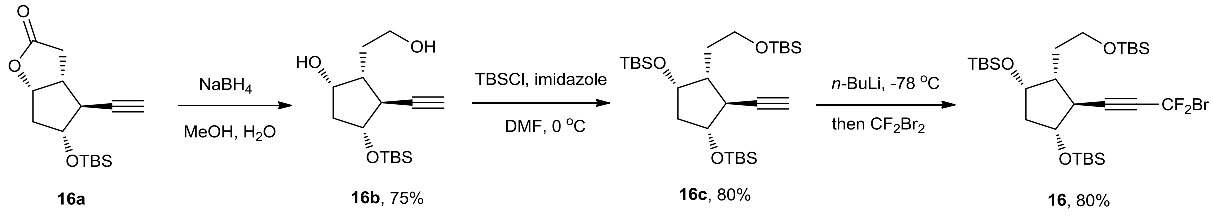

(1*S*,2*R*,3*S*,4*R*)-4-((*tert*-butyldimethylsilyl)oxy)-3-ethynyl-2-(2-hydroxyethyl)cyclopentanol (16b)

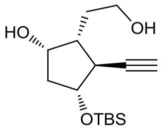

Compound **16b** was prepared according to the literature.[8] To a solution of **16a**[9] in MeOH (10 mL) and H_2O (2.5 mL) was added sodium borohydride (473 mg, 5.0 equiv) at room temperature. After stirring for 4 h at room temperature, additional sodium borohyride (189 mg, 2.0 equiv) was added. The reaction mixture was then stirred for an additional 2 h, neutralized by addition of solid $NaHSO_4$ and diluted with DCM. The organic layer was washed with saturated aqueous $NaHCO_3$ and brine, dried (Na_2SO_4), filtered, and concentrated. The product **16b** (530 mg, 75 % yield) was purified with silica gel chromatography (Petroleum ether/EtOAc = 4:1 to 2:1) as a colorless oil. ^{1}H NMR (400

MHz, $CDCl_3$) δ 4.28 – 4.14 (m, 2 H), 3.87 – 3.79 (m, 1H), 3.77 – 3.69 (m, 1H), 3.12 (s, 1 H), 2.55 – 2.48 (m, 1 H), 2.23 – 2.16 (m, 1 H), 2.15 (d, *J* = 2.5 Hz, 1 H), 1.97 – 1.88 (m, 3 H), 1.78 (ddd, *J* = 14.3, 1.7, 0.9 Hz, 1 H), 0.88 (s, 9 H), 0.11 (s, 3 H), 0.09 (s, 3 H). ^{13}C NMR (101 MHz, $CDCl_3$) δ 85.8, 79.3, 73.5, 70.4, 61.8, 50.3, 44.1, 43.0, 31.3, 25.7, 17.9, -4.7, -5.0. MS (DART): m/z (%) 285 ($M+H^+$, 100), 267. HRMS: Calculated for $C_{15}H_{29}O_3Si$: 285.1800; Found: 285.1879.

(((1*R*,3*S*,4*R*,5*S*)-4-(2-((*tert*-butyldimethylsilyl)oxy)ethyl)-5-ethynylcyclopentane-1,3-diyl)bis(oxy))bis(*tert*-butyldimethylsilane) (16c)

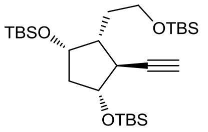

Compound **16b** (530 mg, 1.86 mmol, 1.0 equiv.) was suspended in dry DMF (3 mL) and the solution stirred in a three-necked flask under an argon atmosphere. Subsequently, imidazole (380 mg, 3.0 equiv.) and TBSCl (421 mg, 2.5 equiv.) were added. The reaction was stirred overnight and quenched with saturated ammonium chloride solution. The resulting mixture was extracted with Et_2O twice. The combined organic layers were washed with water and brine, dried over magnesium sulfate and concentrated. The product **16c** (763 mg, 80 % yield) was purified with silica gel chromatography (Petroleum ether/EtOAc = 50:1) as a colorless oil. ^1H NMR (400 MHz, $CDCl_3$) δ 4.17 (ddd, *J* = 8.7, 7.2, 4.1 Hz, 1 H), 4.09 – 4.04 (m, 1 H), 3.74 – 3.67 (m, 1 H), 3.67 – 3.61 (m, 1 H), 2.62 (ddd, *J* = 10.8, 7.0, 2.3 Hz, 1 H), 2.18 (ddd, *J* = 14.1, 8.8, 5.2 Hz, 1 H), 2.07 (d, *J* = 2.4 Hz, 1 H), 1.85 – 1.71 (m, 3 H), 1.61 – 1.52 (m, 1 H), 0.89 (s, 9 H), 0.88 (s, 9 H), 0.88 (s, 9 H), 0.09 (s, 3 H), 0.06 (s, 3 H), 0.04 (s, 9 H), 0.02 (s, 2 H). ^{13}C NMR (101 MHz, $CDCl_3$) δ 86.7, 78.0, 71.9, 69.7, 61.4, 46.9, 44.6, 43.4, 30.8, 26.0, 25.8, 25.8, 18.3, 18.0, 18.0, -4.2, -4.5, -4.9, -5.0, -5.18, -5.24. MS (DART): m/z (%) 513 ($M+H^+$, 100), 381. HRMS: Calculated for $C_{27}H_{57}O_3Si_3$: 513.3610; Found: 513.3604.

(((1*S*,3*R*,4*S*,5*R*)-4-(3-bromo-3,3-difluoroprop-1-yn-1-yl)-5-(2-((*tert*-butyldimethylsilyl)oxy)ethyl)cyclopentane-1,3-diyl)bis(oxy))bis(*tert*-butyldimethylsilane) (16)

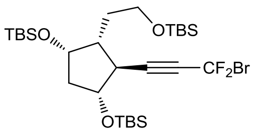

To a solution of **16c** (567 mg, 1.1 mmol, 1.0 equiv) in anhydrous THF (2.2 mL) was added dropwise *n*-BuLi (2.5 M in hexane; 0.5 mL, 1.25 mmol) under an Ar atmosphere at -78 ℃. After stirring for 60 min at -78 ℃, the reaction mixture was cooled to -196 ℃ (liquid N_2 bath). CF_2Br_2 (500 mg, 2.31 mmol) was added to the mixture via cannulation. After the addition was complete, the mixture was allowed to warm to room temperature very slowly and stirred overnight. The reaction mixture was then quenched with sat. aq NH_4Cl and the solvent THF was evaporated under reduced pressure. The residue was extracted with Et_2O and the combined organic layers were washed with H_2O, dried over Na_2SO_4, and concentrated. The residue was purified with silica gel chromatography (Petroleum ether/EtOAc = 100:1) to give the pure product **16** (565 mg, 80% yield) as a colorless oil. ^{1}H NMR (400 MHz, $CDCl_3$) δ 4.27 – 4.21 (m, 1 H), 4.11 – 4.06 (m, 1 H), 3.73 – 3.60 (m, 2 H), 2.86 – 2.77 (m, 1 H), 2.21 (ddd, *J* = 14.0, 8.9, 5.2 Hz, 1 H), 1.92 (ddd, *J* = 12.4, 8.8, 4.7 Hz, 1 H), 1.84 – 1.74 (m, 1 H), 1.74 – 1.65 (m, 1 H), 1.61 (ddd, *J* = 14.4, 3.8, 1.2 Hz, 1 H), 0.89 (s, 9 H), 0.89 (s, 9 H), 0.88 (s, 9 H), 0.10 (s, 3 H), 0.06 (s, 3 H), 0.05 (s, 6 H), 0.03 (s, 3 H). ^{13}C NMR (126 MHz, $CDCl_3$) δ 101.5 (t, *J* = 288.7 Hz), 95.2 (t, *J* = 5.7 Hz), 77.2 (t, *J* = 1.7 Hz), 75.4 (t, *J* = 37.7 Hz), 72.0, 61.0, 46.5, 44.7, 43.5 (t, *J* = 1.8 Hz), 30.8, 25.9, 25.8, 18.3, 18.0, -4.2, -4.7, -4.9, -5.0, -5.2, -5.3. ^{19}F NMR (376 MHz, $CDCl_3$) δ -29.97 (s), -29.99 (s). MS (DART): m/z (%) 643 (100), 641 (M+H$^+$), 511, 509. HRMS: Calculated for $C_{28}H_{56}O_3BrF_2Si_3$: 641.2683; Found: 641.2680.

General Procedure for Ni-Catalyzed Cross-Coupling of Alkylzinc Reagents 1 with *gem*-Difluoropropargyl Bromide 2a

To a 25 mL of Schlenk tube was added Ni(dppf)Cl_2 (2.5 mol %) and Tpy (2.5 mol %). The tube was evacuated and backfilled with Ar for 3 times, then *gem*-difluoropropargyl bromide **2a** (0.6 mmol, 1.0 equiv) and 1,4-dioxane (4 mL) were added. The resulting mixture was stirred at room temperature for 5 min, alkylzinc reagent **1** (0.72 mmol, 1.2 equiv in 2 mL of DMA) was added dropwise over a period of 5 min. The tube was screw capped and put into a preheated oil bath (40 ^{o}C). After stirring for 8 h, the reaction mixture was cooled to room temperature and diluted with EtOAc (50 mL). The resulting mixture was filtered with a pad of cellite. The filtrate was washed with water (15 mL), the organic layer was dried over Na_2SO_4, filtered, and concentrated. The residue was purified with silica gel chromatography to give the pure product.

Characterization Data for Products 3

(3, 3-Difluoro-6-phenylhex-1-yn-1-yl)triisopropylsilane (3a)

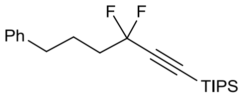

The product (Using Ni(dppf)Cl_2 as a catalyst: 198 mg, 94% yield; Using $NiCl_2$ as a catalyst: 189 mg, 90%) was purified with silica gel chromatography (Petroleum ether) as a colorless oil. ^{1}H NMR (400 MHz, $CDCl_3$) δ 7.33 – 7.28 (m, 2 H), 7.24 – 7.17 (m, 3 H), 2.71 (t, J = 7.4 Hz, 2 H), 2.14 – 2.00 (m, 2 H), 2.00 – 1.89 (m, 2 H), 1.11 (s, 21 H). ^{13}C NMR (126 MHz, $CDCl_3$) δ 141.2, 128.4, 128.4, 126.0, 114.3 (t, J = 232.4 Hz), 99.0 (t, J = 39.1 Hz), 90.0 (t, J = 5.5 Hz), 38.7 (t, J = 26.3 Hz), 34.9, 24.7 (t, J = 3.4 Hz), 18.4, 10.9. ^{19}F NMR (376 MHz, $CDCl_3$) δ -82.2 (t, J = 14.3 Hz). IR (thin film) ν_{max} 3028, 2944, 2867, 2190, 1463, 1310, 1169, 883 cm^{-1}. MS (EI): m/z (%) 307 ($M-C_3H_7^+$, 100), 279, 155, 105, 91, 77. HRMS: Calculated for $C_{18}H_{25}F_2Si$: 307.1694; Found: 307.1693.

Gram-Scale Synthesis of 3a

To a 100 mL of Schlenk tube was added Ni(dppf)Cl_2 (13.68 mg, 0.5 mol %) and Tpy (4.67 mg, 2.5 mol %). The mixture was evacuated and backfilled with Ar for 3 times, then *gem*-difluoropropargyl bromide **2a** (4 mmol, 1 equiv) and 1,4-dioxane (26 mL) were added. The resulting mixture was stirred at room temperature for 10 min, alkylzinc **1a** (4.8 mmol, 1.2 equiv in 13 mL of DMA) was added dropwise over a period of 15 min. The tube was screw capped and put into a preheated oil bath (40 ^oC). After stirring for 24 h, the reaction mixture was cooled to room temperature. The reaction mixture was diluted with EtOAc (250 mL) and filtered with a pad of cellite. The filtrate was washed with water (50 mL). The organic layer was dried over Na_2SO_4, filtered, and concentrated. The product **3a** (1.01 g, 72 % yield) was purified with silica gel chromatography (Petroleum ether) as a colorless oil.

(3,3-Difluoroundec-1-yn-1-yl)triisopropylsilane (3b)

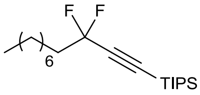

The product (Using Ni(dppf)Cl_2 as a catalyst: 149 mg, 72% yield; Using $NiCl_2$ as a catalyst: 124 mg,

60%) was purified with silica gel chromatography (Petroleum ether) as a colorless oil. ^{1}H NMR (400 MHz, $CDCl_3$) δ 2.08 – 1.95 (m, 2 H), 1.58 (m, 2 H), 1.42 – 1.22 (m, 10 H), 1.10 (s, 21 H), 0.89 (t, *J* = 6.6 Hz, 3 H). ^{13}C NMR (101 MHz, $CDCl_3$) δ 114.4 (t, *J* = 232.2 Hz), 99.2 (t, *J* = 39.2 Hz), 89.6 (t, *J* = 5.5 Hz), 39.4 (t, *J* = 26.0 Hz), 31.8, 29.3, 29.1, 28.9, 23.0 (t, *J* = 3.5 Hz), 22.7, 18.4, 14.1, 10.9. ^{19}F NMR (376 MHz, $CDCl_3$) δ -82.4 (t, *J* = 14.7 Hz). IR (thin film) ν_{max} 2930, 2867, 2185, 1464, 1256, 1075, 883 cm^{-1}. MS (DART): m/z (%) 362 ($M+NH_4^+$), 352, 351 (100), 340, 323. HRMS: Calculated for $C_{20}H_{42}NF_2Si$: 362.3055; Found: 362.3051.

(3, 3-Difluoro-6-phenoxyhex-1-yn-1-yl)triisopropylsilane (3c)

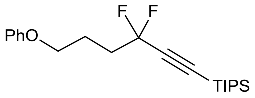

The product (Using Ni(dppf)Cl_2 as a catalyst: 198 mg, 90% yield; Using $NiCl_2$ as a catalyst: 178 mg, 81%) was purified with silica gel chromatography (Petroleum ether/EtOAc = 100:1) as a colorless oil. ^{1}H NMR (400 MHz, $CDCl_3$) δ 7.29 (t, *J* = 7.8 Hz, 2 H), 6.96 (t, *J* = 7.3 Hz, 1 H), 6.90 (d, *J* = 8.0 Hz, 2 H), 4.03 (t, *J* = 6.1 Hz, 2 H), 2.35 – 2.21 (m, 2 H), 2.12 – 2.03 (m, 1 H), 1.11 (s, 21 H). ^{13}C NMR (126 MHz, $CDCl_3$) δ 158.7, 129.4, 120.8, 114.5, 114.1 (t, *J* = 232.4 Hz). 98.8 (t, *J* = 38.9 Hz), 90.2 (t, *J* = 5.6 Hz), 66.5, 36.31 (t, *J* = 26.9 Hz), 23.21 (t, *J* = 3.6 Hz), 18.44, 10.9. ^{19}F NMR (376 MHz, $CDCl_3$) δ -82.8 (t, *J* = 14.7 Hz). IR (thin film) ν_{max} 3064, 2945, 2185, 1601, 1472, 1246, 1006, 883 cm^{-1}. MS (EI): m/z (%) 366 (M^+), 207, 155, 137, 91, 77 (100). HRMS: Calculated for $C_{18}H_{25}OF_2Si$: 366.2191; Found: 366.2189.

***tert*-Butyl((4,4-difluoro-6-(triisopropylsilyl)hex-5-yn-1-yl)oxy)dimethylsilane (3d)**

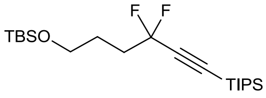

The product (212 mg, 87% yield) was purified with silica gel chromatography (Petroleum ether/EtOAc = 100:1) as a colorless oil. ^{1}H NMR (400 MHz, $CDCl_3$) δ 3.66 (t, *J* = 6.1 Hz, 2 H), 2.21 – 2.02 (m, 2 H), 1.82 – 1.74 (m, 2 H), 1.09 (s, 21 H), 0.89 (s, 9 H), 0.05 (s, 6 H). ^{13}C NMR (101 MHz, $CDCl_3$) δ 114.4 (t, *J* = 232.1 Hz), 99.1 (t, *J* = 39.1 Hz), 89.8 (t, *J* = 5.6 Hz), 61.9, 36.2 (t, *J* = 26.6 Hz), 26.5 (t, *J* = 3.3 Hz), 25.9, 18.4, 10.9, -5.4. ^{19}F NMR (376 MHz, $CDCl_3$) δ -82.5 (t, *J* = 14.8 Hz). IR (thin film) ν_{max}2947, 2867, 2310, 1463, 1257, 1103, 837 cm^{-1}. MS (DART): m/z (%) 405

(M+H$^+$), 391, 386, 385 (100). HRMS: Calculated for $C_{21}H_{43}OF_2Si_2$: 405.2815; Found: 405.2815.

(6-(Benzyloxy)-3,3-difluorohex-1-yn-1-yl)triisopropylsilane (3e)

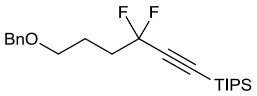

The product (165 mg, 72% yield) was purified with silica gel chromatography (Petroleum ether/EtOAc = 100:1) as a colorless oil. ^{1}H NMR (500 MHz, $CDCl_3$) δ 7.38 – 7.27 (m, 5 H), 4.52 (s, 2 H), 3.55 (t, *J* = 6.2 Hz, 2 H), 2.23 – 2.13 (m, 2 H), 1.98 – 1.84 (m, 2 H), 1.11 (s, 21 H). ^{13}C NMR (126 MHz, $CDCl_3$) δ 138.4, 128.4, 127.6, 127.5, 114.2 (t, *J* = 232.3 Hz), 99.0 (t, *J* = 39.1 Hz), 89.9 (t, *J* = 5.6 Hz), 72.8, 69.0, 36.4 (t, *J* = 26.7 Hz), 23.5 (t, *J* = 3.5 Hz), 18.4, 10.9. ^{19}F NMR (376 MHz, $CDCl_3$) δ -82.7 (t, *J* = 14.8 Hz). IR (thin film) ν_{max} 3088, 2944, 2190, 1722, 1455, 1205, 1103 cm^{-1}. MS (EI): m/z (%) 337 (M-$C_3H_7^+$), 187, 91 (100), 77. HRMS: Calculated for $C_{19}H_{27}OF_2Si$: 337.1799; Found: 337.1802.

7, 7-Difluoro-9-(triisopropylsilyl)non-8-yn-1-yl acetate (3f)

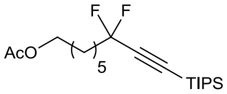

The product (Using Ni(dppf)Cl_2 as a catalyst: 128 mg, 57% yield; Using $NiCl_2$ as a catalyst: 146 mg, 65%) was purified with silica gel chromatography (Petroleum ether/EtOAc = 50:1) as a faint yellow oil. ^{1}H NMR (400 MHz, $CDCl_3$) δ 4.05 (t, *J* = 6.7 Hz, 2 H), 2.14 – 1.94 (m, 5 H), 1.67 – 1.54 (m, 4 H), 1.46 – 1.31 (m, 4 H), 1.09 (s, 21 H). ^{13}C NMR (101 MHz, $CDCl_3$) δ 171.1, 114.3 (t, *J* = 233.1 Hz), 99.1 (t, *J* = 39.1 Hz), 89.8 (t, *J* = 5.5 Hz), 64.4, 39.2 (t, *J* = 26.2 Hz), 28.5, 28.4, 25.7, 22.9 (t, *J* = 3.5 Hz), 21.0, 18.4, 10.9. ^{19}F NMR (376 MHz, $CDCl_3$) δ -82.5 (t, *J* = 14.7 Hz). IR (thin film) ν_{max} 2944, 2185, 1743, 1465, 1240, 1070, 680 cm^{-1}. MS (EI): m/z (%) 331 (M-$C_3H_7^+$), 173 (100), 91, 77. HRMS: Calculated for $C_{17}H_{29}O_2F_2Si$: 331.1905; Found: 331.1910.

1-(4,4-Difluoro-6-(triisopropylsilyl)hex-5-yn-1-yl)-1H-indole (3g)

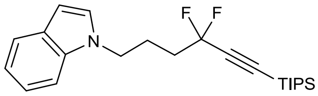

The product (199 mg, 85% yield) was purified with silica gel chromatography (Petroleum

ether/EtOAc = 100:1) as a faint yellow oil. ^{1}H NMR (500 MHz, $CDCl_3$) δ 7.64 (d, *J* = 7.9 Hz, 1 H), 7.34 (d, *J* = 8.2 Hz, 1 H), 7.21 (t, *J* = 7.2 Hz, 1 H), 7.12 (t, *J* = 7.4 Hz, 1 H), 7.09 (d, *J* = 3.1 Hz, 1 H), 6.52 (d, *J* = 3.1 Hz, 1 H), 4.22 (t, *J* = 6.9 Hz, 2 H), 2.20 – 2.13 (m, 2 H), 2.11 – 2.01 (m, 2 H), 1.08 (s, 21 H). ^{13}C NMR (126 MHz, $CDCl_3$) δ 135.9, 128.6, 127.5, 121.6, 121.0, 119.4, 113.8 (t, *J* = 232.7 Hz), 109.2, 101.5, 98.5 (t, *J* = 39.0 Hz), 90.6 (t, *J* = 5.4 Hz), 45.3, 36.7 (t, *J* = 27.0 Hz), 24.1 (t, *J* = 3.2 Hz), 18.4, 10.9. ^{19}F NMR (376 MHz, $CDCl_3$) δ -82.0 (t, *J* = 14.1 Hz). IR (thin film) ν_{max} 3056, 2944, 2867, 2500, 2302, 1513, 1464, 1247, 883 cm^{-1}. MS (EI): m/z (%) 389 (M$^+$), 346, 304, 130 (100), 77. HRMS: Calculated for $C_{23}H_{33}NF_2Si$: 389.2350; Found: 389.2345.

4-(4,4-Difluoro-6-(triisopropylsilyl)hex-5-yn-1-yl)morpholine (3h)

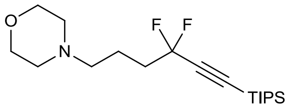

The product (173 mg, 80% yield) was purified with silica gel chromatography (Petroleum ether/EtOAc = 5:1) as a colorless oil. ^{1}H NMR (400 MHz, $CDCl_3$) δ 3.70 (t, *J* = 4.6 Hz, 4 H), 2.45 – 2.35 (m, 4 H), 2.15 – 2.03 (m, 2 H), 1.80 – 1.71 (m, 2 H), 1.08 (s, 21 H). ^{13}C NMR (101 MHz, $CDCl_3$) δ 114.2 (t, *J* = 232.3 Hz), 99.0 (t, *J* = 39.2 Hz), 89.8 (t, *J* = 5.6 Hz), 66.9, 57.6, 53.5, 37.1 (t, *J* = 26.6 Hz), 20.1 (t, *J* = 3.5 Hz), 18.4, 10.9. ^{19}F NMR (376 MHz, $CDCl_3$) δ -82.4 (t, *J* = 14.7 Hz). IR (thin film) ν_{max} 2945, 2867, 2133, 1463, 1037 cm^{-1}. MS (EI): m/z (%) 359, 316, 126, 100 (100). HRMS: Calculated for $C_{19}H_{35}NOF_2Si$: 359.2456; Found: 359.2460.

Ethyl 5, 5-difluoro-7-(triisopropylsilyl)hept-6-ynoate (3i)

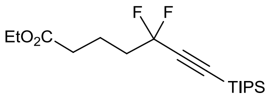

The product (Using Ni(dppf)Cl_2 as a catalyst: 189 mg, 91% yield; Using $NiCl_2$ as a catalyst: 170 mg, 82% yield) was purified with silica gel chromatography (Petroleum ether/EtOAc = 40:1) as a colorless oil. ^{1}H NMR (400 MHz, $CDCl_3$) δ 4.14 (q, *J* = 7.1 Hz, 2 H), 2.39 (t, *J* = 7.4 Hz, 2 H), 2.17 – 2.02 (m, 2 H), 1.96 – 1.87 (m, 2 H), 1.25 (t, *J* = 7.1 Hz, 3 H), 1.09 (s, 21 H). ^{13}C NMR (101 MHz, $CDCl_3$) δ 172.7, 113.9 (t, *J* = 232.5 Hz), 98.7 (t, *J* = 38.9 Hz), 90.3 (t, *J* = 5.6 Hz), 60.4, 38.5 (t, *J* = 26.7 Hz), 33.3, 18.6 (t, *J* = 3.9 Hz), 18.4, 14.2, 10.9. ^{19}F NMR (376 MHz, $CDCl_3$) δ -82.5 (t, *J* = 14.5 Hz). IR (thin film) ν_{max} 2946, 2868, 2182, 1739, 1464, 1170, 833 cm^{-1}. MS (EI): m/z (%) 346 (M$^+$),

327, 315, 306 (100). HRMS: Calculated for $C_{18}H_{32}O_2F_2Si$: 346.2140; Found: 346.2132.

(5-(4-Bromophenyl)-3,3-difluoropent-1-yn-1-yl)triisopropylsilane (3j)

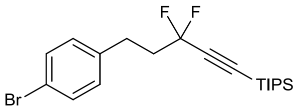

The product (212 mg, 85% yield) was purified with silica gel chromatography (Petroleum ether) as a colorless oil. ^{1}H NMR (500 MHz, $CDCl_3$) δ 7.43 (d, *J* = 8.4 Hz, 2 H), 7.08 (d, *J* = 8.3 Hz, 2 H), 2.89 – 2.79 (m, 2 H), 2.38 – 2.26 (m, 2 H), 1.11 (s, 21 H). ^{13}C NMR (125.7 MHz, $CDCl_3$) δ 138.9, 131.7, 130.1, 120.2, 113.5 (t, *J* = 233.2 Hz), 98.7 (t, *J* = 38.8 Hz), 90.6 (t, *J* = 5.6 Hz), 41.1 (t, *J* = 26.5 Hz), 28.8 (t, *J* = 3.9 Hz), 18.5, 10.9. ^{19}F NMR (376 MHz, $CDCl_3$) δ -83.3 (t, *J* = 14.2 Hz). IR (thin film) ν_{max} 2945, 2867, 2185, 1490, 1231, 1012 cm^{-1}. MS (EI): m/z (%) 416, 414 (M^+), 373, 371, 169, 167 (100), 115, 77. HRMS: Calculated for $C_{20}H_{29}F_2BrSi$: 414.1190; Found: 414.1188.

(8-Chloro-3,3-difluorooct-1-yn-1-yl)triisopropylsilane (3k)

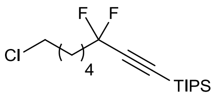

The product (144 mg, 71% yield) was purified with silica gel chromatography (Petroleum ether) as a colorless oil. ^{1}H NMR (400 MHz, $CDCl_3$) δ 3.54 (t, *J* = 6.6 Hz, 2 H), 2.11 – 1.98 (m, 2 H), 1.84 – 1.75 (m, 2 H), 1.66 – 1.57 (m, 2 H), 1.57 – 1.48 (m, 2 H), 1.10 (s, 21 H). ^{13}C NMR (101 MHz, $CDCl_3$) δ 114.2 (t, *J* = 232.4 Hz), 99.0 (t, *J* = 39.1 Hz), 90.0 (t, *J* = 5.6 Hz), 44.7, 39.2 (t, *J* = 26.3 Hz), 32.3, 26.2, 22.4 (t, *J* = 3.6 Hz), 18.5, 11.0. ^{19}F NMR (376 MHz, $CDCl_3$) δ -82.6 (t, *J* = 14.6 Hz). IR (thin film) ν_{max} 2945, 2868, 2267, 1463, 833 cm^{-1}. MS (EI): m/z (%) 293 ($M-C_3H_7^+$), 209, 105, 91, 79 (100). HRMS: Calculated for $C_{14}H_{24}F_2ClSi$: 293.1304; Found: 293.1301.

Triisopropyl(3,3,7-trifluorohept-1-yn-1-yl)silane (3l)

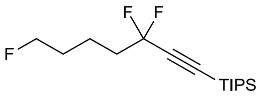

The product (123 mg, 67% yield) was purified with silica gel chromatography (Petroleum ether) as a colorless oil. ^{1}H NMR (400 MHz, $CDCl_3$) δ 4.46 (dt, *J* = 47.2, 5.6 Hz, 2 H), 2.16 – 2.03 (m, 2 H), 1.85 – 1.67 (m, 4 H), 1.09 (s, 21 H). ^{13}C NMR (101 MHz, $CDCl_3$) δ 114.1 (t, *J* = 233.6 Hz), 98.8 (t, *J*

= 39.3 Hz), 90.2 (t, J = 5.6 Hz), 83.5 (d, J = 166.1 Hz), 38.9 (t, J = 26.6 Hz), 29.7 (d, J = 20.0 Hz), 19.2 (dt, J = 5.2, 3.8 Hz), 18.4, 10.9. ^{19}F NMR (376 MHz, $CDCl_3$) δ -82.6 (t, J = 14.5 Hz, 2 F), -219.3 (tt, J = 47.4, 25.0 Hz, 1 F). IR (thin film) ν_{max} 2946, 2868, 2309, 1463, 1297, 1009, 883 cm^{-1}. MS (EI): m/z (%) 264 ($M-C_3H_7$ + H^+), 193, 109, 91 (100), 81, 77. HRMS: Calculated for $C_{13}H_{23}F_3Si$: 264.1521; Found: 264.1523.

(3,3-Difluoro-6-(4,4,5,5-tetramethyl-1,3,2-dioxaborolan-2-yl)hex-1-yn-1-yl)triisopropylsilane (3m)

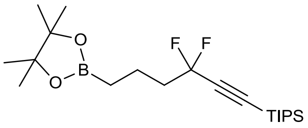

The product (144 mg, 60% yield) was purified with silica gel chromatography (Petroleum ether/EtOAc = 50:1) as a colorless oil. ^1H NMR (400 MHz, $CDCl_3$) δ 2.11 – 1.98 (m, 2 H), 1.74 – 1.64 (m, 2 H), 1.23 (s, 12 H), 1.09 (s, 21 H), 0.84 (t, J = 7.8 Hz, 2 H). ^{13}C NMR (101 MHz, $CDCl_3$) δ 114.3 (t, J = 233.5 Hz), 99.2 (t, J = 39.2 Hz), 89.6 (t, J = 5.6 Hz), 83.0, 41.5 (t, J = 25.9 Hz), 24.8, 18.4, 17.7 (t, J = 3.8 Hz), 10.9. ^{19}F NMR (376 MHz, $CDCl_3$) δ -82.4 (t, J = 14.7 Hz). IR (thin film) ν_{max} 2961, 2868, 2237, 1464, 1381, 1035 cm^{-1}. MS (DART): m/z (%) 418 ($M+NH_4^+$, 100), 417, 400, 381, 363. HRMS: Calculated for $C_{21}H_{43}O_2N^{10}BF_2Si$: 417.3160; Found: 417.3156.

(5-(1,3-Dioxolan-2-yl)-3,3-difluoropent-1-yn-1-yl)triisopropylsilane (3n)

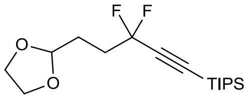

The product (Using Ni(dppf)Cl_2 as a catalyst: 150 mg, 75% yield; Using $NiCl_2$ as a catalyst: 130 mg, 65% yield) was purified with silica gel chromatography (Petroleum ether/EtOAc = 50:1) as a faint yellow oil. ^1H NMR (400 MHz, $CDCl_3$) δ 4.96 (t, J = 4.4 Hz, 1 H), 3.97 (t, J = 6.9 Hz, 2 H), 3.86 (t, J = 6.9 Hz, 2 H), 2.24 – 2.12 (m, 2 H), 1.97 – 1.90 (m, 2 H), 1.09 (s, 21 H). ^{13}C NMR (101 MHz, $CDCl_3$) δ 113.9 (t, J = 232.3 Hz), 103.0, 98.8 (t, J = 39.1 Hz), 90.0 (t, J = 5.6 Hz), 65.0, 33.7 (t, J = 27.3 Hz), 27.48 (t, J = 3.4 Hz), 18.4, 10.9. ^{19}F NMR (376 MHz, $CDCl_3$) δ -83.1 (t, J = 14.7 Hz). IR (thin film) ν_{max} 2945, 2867, 2185, 1464, 1038, 883 cm^{-1}. MS (EI): m/z (%) 331 ($M-H^+$), 289, 205, 73 (100). HRMS: Calculated for $C_{17}H_{19}O_2F_2Si$: 331.1905; Found: 331.1908.

7,7-Difluoro-9-(triisopropylsilyl)non-8-yn-1-yl ferrocene formate (3o)

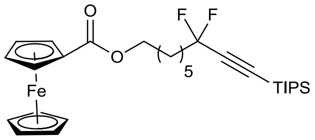

The reaction was carried out in 0.3 mmol scale and 1.5 equiv of alkylzinc reagent was employed. The product (120 mg, 73% yield) was purified with silica gel chromatography (Petroleum ether/EtOAc = 50:1) as a brown red oil. ^{1}H NMR (400 MHz, $CDCl_3$) δ 4.81 – 4.79 (m, 2 H), 4.40 – 4.38 (m, 2 H), 4.27 – 4.15 (m, 7 H), 2.12 – 1.99 (m, 2 H), 1.78 – 1.68 (m, 2 H), 1.67 – 1.58 (m, 2 H), 1.52 – 1.40 (m, 4 H), 1.09 (s, 21 H). ^{13}C NMR (101 MHz, $CDCl_3$) δ 171.7, 114.2 (t, *J* = 232.3 Hz), 99.0 (t, *J* = 39.1 Hz), 89.8 (t, *J* = 5.4 Hz), 71.2, 70.0, 69.7, 64.0, 39.2 (t, *J* = 26.1 Hz), 28.6, 28.5, 25.8, 22.9 (t, *J* = 3.0 Hz), 18.4, 10.8. ^{19}F NMR (376 MHz, $CDCl_3$) δ -82.4 (t, *J* = 14.7 Hz). IR (thin film) ν_{max} 3098, 2943, 2190, 1716, 1461, 1275, 1000, 822 cm^{-1}. MS (MALDI): m/z (%) 544 (M^+), 525 (100), 526, 505, 349. HRMS: Calculated for $C_{29}H_{42}O_2F_2{}^{54}FeSi$: 542.2; Found: 542.2.

(3,3-Difluorohept-6-en-1-yn-1-yl)triisopropylsilane (3p)

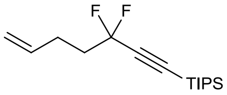

The product (120 mg, 70% yield) was purified with silica gel chromatography (Petroleum ether) as a colorless oil. ^{1}H NMR (400 MHz, $CDCl_3$) δ 5.84 (ddt, *J* = 16.8, 10.3, 6.6 Hz, 1 H), 5.08 (dd, *J* = 17.1, 1.5 Hz, 1 H), 5.02 (dd, *J* = 10.2, 1.1 Hz, 1 H), 2.39 – 2.39 (m, 2 H), 2.20 – 2.07 (m, 2 H), 1.10 (s, 21 H). ^{13}C NMR (101 MHz, $CDCl_3$) δ 136.3, 115.6, 113.9 (t, *J* = 232.7 Hz), 98.9 (t, *J* = 39.0 Hz), 90.1 (t, *J* = 5.7 Hz), 38.8 (t, *J* = 26.4 Hz), 27.4 (t, *J* = 3.9 Hz), 18.4, 10.9. ^{19}F NMR (376 MHz, $CDCl_3$) δ -82.9 (t, *J* = 14.3 Hz). IR (thin film) ν_{max} 3028, 2894, 2302, 1645, 1464, 1071 cm^{-1}. MS (TOF-MS-EI): m/z (%) 286 (M^+), 243, 173, 105, 91 (100), 77. HRMS: Calculated for $C_{16}H_{28}F_2Si$: 286.1928; Found: 286.1923.

(*R*)-(3,3-Difluoro-6,10-dimethylundec-9-en-1-yn-1-yl)triisopropylsilane (3q)

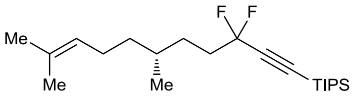

The product (162 mg, 73% yield) was purified with silica gel chromatography (Petroleum ether) as a

colorless oil. ^{1}H NMR (400 MHz, $CDCl_3$) δ 5.09 (t, *J* = 7.1 Hz, 1 H), 2.11 – 1.90 (m, 4 H), 1.69 (s, 3 H), 1.60 (s, 3 H), 1.51 – 1.28 (m, 4 H), 1.23 – 1.12 (m, 2 H), 1.09 (s, 21 H), 0.90 (d, *J* = 6.5 Hz, 3 H). ^{13}C NMR (101 MHz, $CDCl_3$) δ 131.4, 124.5, 114.6 (t, *J* = 232.2 Hz), 99.1 (t, *J* = 39.2 Hz), 89.7 (t, *J* = 5.5 Hz), 37.0 (t, *J* = 26.0 Hz), 36.8, 31.8, 29.8 (t, *J* = 3.2 Hz), 25.7, 25.4, 19.3, 18.4, 17.7, 10.9. ^{19}F NMR (376 MHz, $CDCl_3$) δ -82.5 (t, *J* = 14.5 Hz). IR (thin film) ν_{max} 2945, 2868, 2178, 1464, 1256, 1019 cm^{-1}. MS (EI): m/z (%) 370, 327, 175, 133, 105, 77, 69 (100), 55. HRMS: Calculated for $C_{22}H_{40}F_2Si$: 370.2867; Found: 370.2871.

(3,3-Difluoro-5-(thiophen-2-yl)pent-1-yn-1-yl)triisopropylsilane (3r)

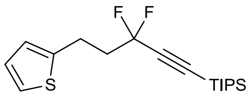

The product (185 mg, 90% yield) was purified with silica gel chromatography (Petroleum ether) as a colorless oil. ^{1}H NMR (400 MHz, $CDCl_3$) δ 7.16 (dd, *J* = 5.1, 1.1 Hz, 1 H), 6.94 (dd, *J* = 5.1, 3.5 Hz, 1 H), 6.84 (dd, *J* = 3.4, 1.0 Hz, 1 H), 3.17 – 3.08 (m, 2 H), 2.51 – 2.36 (m, 2 H), 1.12 (s, 21 H). ^{13}C NMR (101 MHz, $CDCl_3$) δ 142.4, 126.9, 124.6, 123.6, 113.3 (t, *J* = 233.2 Hz), 98.5 (t, *J* = 38.8 Hz), 90.7 (t, *J* = 5.7 Hz), 41.6 (t, *J* = 26.7 Hz), 23.6 (t, *J* = 4.3 Hz), 18.4, 10.9. ^{19}F NMR (376 MHz, $CDCl_3$) δ -83.6 (t, *J* = 14.1 Hz). IR (thin film) ν_{max} 2945, 2867, 2189, 1464, 1098 cm^{-1}. MS (EI): m/z (%) 342 (M^+), 299 (100), 97, 77. HRMS: Calculated for $C_{18}H_{28}F_2SSi$: 342.1649; Found: 342.1644.

General Procedure for Ni-Catalyzed Cross-Coupling of Alkylzinc Reagents 1 with *gem*-Difluoropropargyl Bromides 2

To a 25 mL of Schlenk tube was added Ni(dppf)Cl_2 (5 mol %) and Tpy (5 mol %). The tube was evacuated and backfilled with Ar for 3 times, then *gem*-difluoropropargyl bromide **2** (0.3 mmol, 1.0 equiv) and 1,4-dioxane (2 mL) were added. The resulting mixture was stirred at room temperature for 5 min, alkylzinc **1** (0.45 mmol, 1.5 equiv in 1 mL of DMA) was then added dropwise over a period of 5 min. The tube was screw capped and put into a preheated oil bath (40 ^{o}C). After stirring for 8 h, the reaction mixture was cooled to room temperature. The reaction mixture was diluted with EtOAc (50 mL) and filtered with a pad of cellite. The filtrate was washed with water (15 mL). The organic layer was dried over Na_2SO_4, filtered, and concentrated. The residue was purified with silica gel chromatography to give the pure product.

Characterization Data for Products 4

(3,3-Difluoro-6-phenylhex-1-yn-1-yl)(methyl)diphenylsilane (4a)

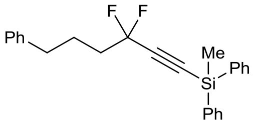

The product (96 mg, 82% yield) was purified with silica gel chromatography (Petroleum ether) as a colorless oil. ^{1}H NMR (400 MHz, $CDCl_3$) δ 7.64 (d, *J* = 6.6 Hz, 4 H), 7.50 – 7.38 (m, 6 H), 7.34 – 7.28 (m, 2 H), 7.27 – 7.21 (m, 1H), 7.18 (d, *J* = 7.4 Hz, 2 H), 2.72 (t, *J* = 7.6 Hz, 2 H), 2.20 – 2.07 (m, 2 H), 2.02 – 1.92 (m, 2 H), 0.77 (s, 3 H). ^{13}C NMR (101 MHz, $CDCl_3$) δ 141.2, 134.5, 133.4, 130.1, 128.4, 128.3, 128.1, 126.0, 114.1 (t, *J* = 233.2 Hz), 99.7 (t, *J* = 39.6 Hz), 89.7 (t, *J* = 5.5 Hz), 38.5 (t, *J* = 26.0 Hz), 34.9, 24.5 (t, *J* = 3.4 Hz), -2.6. ^{19}F NMR (376 MHz, $CDCl_3$) δ -83.2 (t, *J* = 14.7 Hz). IR (thin film) ν_{max} 3072, 2962, 1497, 1170, 1117 cm^{-1}. MS (EI): m/z (%) 375 ($M-CH_3^+$), 297, 197, 139, 105, 97 (100), 77. HRMS: Calculated for $C_{24}H_{21}F_2Si$: 375.1381; Found: 375.1378.

(3,3-Difluoro-6-phenylhex-1-yn-1-yl)triethylsilane (4b)

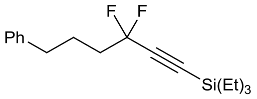

The product (76 mg, 82% yield) was purified with silica gel chromatography (Petroleum ether) as a colorless oil. ^{1}H NMR (400 MHz, $CDCl_3$) δ 7.33 – 7.27 (m, 2 H), 7.24 – 7.16 (m, 3 H), 2.70 (t, *J* = 7.5 Hz, 2 H), 2.12 – 1.98 (m, 2 H), 1.96 – 1.87 (m, 2 H), 1.01 (t, *J* = 7.9 Hz, 9 H), 0.65 (q, *J* = 7.9 Hz, 6 H). ^{13}C NMR (101 MHz, $CDCl_3$) δ 141.3, 128.4, 128.3, 126.0, 114.2 (t, *J* = 232.4 Hz), 98.1 (t, *J* = 39.1 Hz), 90.9 (t, *J* = 5.5 Hz), 38.6 (t, *J* = 26.3 Hz), 34.9, 24.6 (t, *J* = 3.4 Hz), 7.3, 3.9. ^{19}F NMR (376 MHz, $CDCl_3$) δ -82.4 (t, *J* = 14.5 Hz). IR (thin film) ν_{max} 3064, 2956, 2877, 2178, 1604, 1456, 1169, 740 cm^{-1}. MS (EI): m/z (%) 308 (M^+), 279, 251, 153, 117, 105 (100), 91, 77. HRMS: Calculated for $C_{18}H_{26}F_2Si$: 308.1772; Found: 308.1767.

(3,3-Difluorohex-1-yne-1,6-diyl)dibenzene (4c)

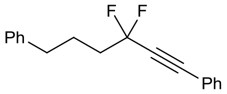

The product (65 mg, 80% yield) was purified with silica gel chromatography (Petroleum ether) as a

colorless oil. ^{1}H NMR (400 MHz, $CDCl_3$) δ 7.54 – 7.49 (m, 2 H), 7.45 – 7.29 (m, 5 H), 7.25 – 7.20 (m, 3 H), 2.75 (t, *J* = 7.6 Hz, 2 H), 2.25 – 2.12 (m, 2 H), 2.05 – 1.96 (m, 2 H). ^{13}C NMR (101 MHz, $CDCl_3$) δ 141.22, 132.1 (t, *J* = 2.2 Hz), 129.8, 128.4, 128.4, 126.1, 120.2 (t, *J* = 2.7 Hz), 115.3 (t, *J* = 232.3 Hz), 86.7 (t, *J* = 6.7 Hz), 81.7 (t, *J* = 40.7 Hz), 38.8 (t, *J* = 26.5 Hz), 35.0, 24.5 (t, *J* = 3.3 Hz). ^{19}F NMR (376 MHz, $CDCl_3$) δ -82.0 (t, *J* = 14.6 Hz). IR (thin film) ν_{max} 3086, 2962, 2242, 1603, 1507, 1323, 1165, 983 cm^{-1}. MS (EI): m/z (%) 270 (M^+), 205, 191, 153 (100), 117, 91, 77. HRMS: Calculated for $C_{18}H_{16}F_2$: 270.1220; Found: 270.1215.

(*R*)-(3,3-Difluoro-6,10-dimethylundec-9-en-1-yn-1-yl)benzene (4d)

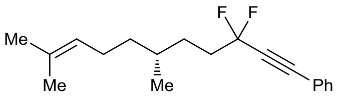

The product (65 mg, 74% yield) was purified with silica gel chromatography (Petroleum ether) as a colorless oil. ^{1}H NMR (400 MHz, $CDCl_3$) δ 7.53 – 7.49 (m, 2 H), 7.44 – 7.33 (m, 3 H), 5.11 (t, *J* = 7.1 Hz, 1 H), 2.23 – 1.94 (m, 4 H), 1.69 (s, 3 H), 1.61 (s, 3 H), 1.57 – 1.32 (m, 4 H), 1.28 – 1.16 (m, 1 H), 0.94 (d, *J* = 6.5 Hz, 3 H). ^{13}C NMR (101 MHz, $CDCl_3$) δ 132.1 (t, *J* = 2.2 Hz), 131.4, 129.8, 128.4, 124.5, 120.3 (t, *J* = 2.6 Hz), 115.7 (t, *J* = 232.1 Hz), 86.5 (t, *J* = 6.8 Hz), 81.8 (t, *J* = 40.9 Hz), 37.1 (t, *J* = 26.2 Hz), 36.7, 31.8, 29.6 (t, *J* = 3.1 Hz), 25.7, 25.4, 19.4, 17.6. ^{19}F NMR (376 MHz, $CDCl_3$) δ -82.2 (t, *J* = 14.7 Hz). IR (thin film) ν_{max} 3056, 2928, 2243, 1559, 1507, 1491, 1322, 1169, 1016 cm^{-1}. MS (EI): m/z (%) 290 (M^+), 275, 229, 151, 109, 69 (100), 41. HRMS: Calculated for $C_{19}H_{24}F_2$: 290.1846; Found: 290.1845.

***tert*-Butyl((4,4-difluoro-7-phenylhept-2-yn-1-yl)oxy)dimethylsilane (4e)**

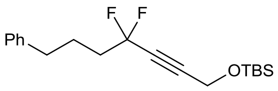

The product (66 mg, 65% yield) was purified with silica gel chromatography (Petroleum ether/EtOAc = 50:1) as a colorless oil. ^{1}H NMR (400 MHz, $CDCl_3$) δ 7.33 – 7.27 (m, 2 H), 7.23 – 7.17 (m, 2 H), 4.38 (t, *J* = 4.4 Hz, 2 H), 2.69 (t, *J* = 7.6 Hz, 2 H), 2.12 – 2.00 (m, 2 H), 1.94 – 1.86 (m, 2 H), 0.91 (s, 9 H), 0.12 (s, 6 H). ^{13}C NMR (101 MHz, $CDCl_3$) δ 141.2, 128.4, 128.4, 126.01, 114.6 (t, *J* = 232.4 Hz), 85.8 (t, *J* = 6.7 Hz), 77.8 (t, *J* = 40.8 Hz), 51.2 (t, *J* = 1.9 Hz), 38.6 (t, *J* = 26.2 Hz), 35.0, 25.7, 24.4 (t, *J* = 3.4 Hz), -5.2. ^{19}F NMR (376 MHz, $CDCl_3$) δ -82.9 (tt, *J* = 14.6, 4.3

Hz). IR (thin film) ν_{max} 3064, 2956, 2858, 2254, 1604, 1463, 1257, 1104, 837 cm^{-1}. MS (EI): m/z (%) 281 ($M-C_4H_9^+$), 261, 167, 159 (100), 105, 91, 77. HRMS: Calculated for $C_{15}H_{19}OF_2Si$: 281.1173; Found: 281.1168.

***tert*-Butyl((6,6-difluoro-9-phenylnon-4-yn-1-yl)oxy)dimethylsilane (4f)**

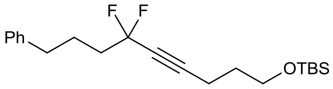

The product (95 mg, 86% yield) was purified with silica gel chromatography (Petroleum ether/EtOAc = 100:1) as a colorless oil. ^1H NMR (400 MHz, $CDCl_3$) δ 7.33 – 7.27 (m, 2 H), 7.23 – 7.15 (m, 3 H), 3.68 (t, *J* = 5.9 Hz, 2 H), 2.69 (t, *J* = 7.5 Hz, 2 H), 2.40 – 2.32 (m, 2 H), 2.09 – 1.97 (m, 2 H), 1.94 – 1.84 (m, 2 H), 1.78 – 1.69 (m, 2 H), 0.90 (s, 9 H), 0.05 (s, 6 H). ^{13}C NMR (101 MHz, $CDCl_3$) δ 141.3, 128.4, 128.3, 126.0, 114.8 (t, *J* = 231.3 Hz), 88.3 (t, *J* = 6.6 Hz), 74.0 (t, *J* = 40.3 Hz), 61.1, 38.8 (t, *J* = 26.7 Hz), 35.0, 30.8, 25.9, 24.6 (t, *J* = 3.4 Hz), 18.3, 14.8 (t, *J* = 2.1 Hz), -5.4. ^{19}F NMR (376 MHz, $CDCl_3$) δ -81.0 (tt, *J* = 14.4, 4.9 Hz). IR (thin film) ν_{max} 3064, 2956, 2857, 2251, 1497, 1284, 1108, 836 cm^{-1}. MS (DART): m/z (%) 384 ($M+NH_4^+$), 373 (100), 367 ($M+H^+$), 327. HRMS: Calculated for $C_{21}H_{33}OF_2Si$: 367.2269; Found: 367.2263.

(4,4-Difluorododec-5-yn-1-yl)benzene (4g)

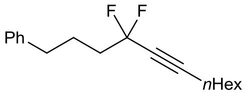

The product (69 mg, 82% yield) was purified with silica gel chromatography (Petroleum ether) as a colorless oil. ^1H NMR (400 MHz, $CDCl_3$) δ 7.33 – 7.27 (m, 2 H), 7.24 – 7.15 (m, 3 H), 2.69 (t, *J* = 7.5 Hz, 2 H), 2.30 – 2.22 (m, 2 H), 2.11 – 1.97 (m, 2 H), 1.90 (m, 2 H), 1.60 – 1.49 (m, 2 H), 1.44 – 1.33 (m, 2 H), 1.34 – 1.20 (m, 4 H), 0.90 (t, *J* = 6.7 Hz, 3 H). ^{13}C NMR (101 MHz, $CDCl_3$) δ 141.4, 128.39, 128.36, 126.0, 115.0 (t, *J* = 231.1 Hz), 88.8 (t, *J* = 6.7 Hz), 73.9 (t, *J* = 40.0 Hz), 38. 9 (t, *J* = 26.7 Hz), 35.0), 31.2, 28.4, 27.8 (t, *J* = 1.8 Hz), 24.6 (t, *J* = 3.4 Hz), 22.5, 18.4 (t, *J* = 2.3 Hz), 14.0. ^{19}F NMR (376 MHz, $CDCl_3$) δ -80.8 (tt, *J* = 14.5, 5.0 Hz). IR (thin film) ν_{max} 3064, 2932, 2859, 2253, 1497, 1316, 1170, 750 cm^{-1}. MS (EI): m/z (%) 278 (M^+), 208, 180, 129, 117 (100), 91. HRMS: Calculated for $C_{18}H_{24}F_2$: 278.1846; Found: 278.1843.

((4,4-Difluorododec-5-yn-1-yl)oxy)benzene (4h)

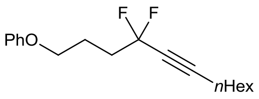

The product (65 mg, 74% yield) was purified with silica gel chromatography (Petroleum ether/EtOAc = 100:1) as a colorless oil. ^{1}H NMR (400 MHz, $CDCl_3$) δ 7.33 – 7.27 (m, 2 H), 6.98 – 6.94 (m, 1 H), 6.93 – 6.89 (m, 2 H), 4.03 (t, *J* = 6.2 Hz, 2 H), 2.31 – 2.18 (m, 4 H), 2.11 – 2.02 (m, 2 H), 1.60 – 1.51 (m, 2 H), 1.46 – 1.36 (m, 2 H), 1.36 – 1.24 (m, 4 H), 0.91 (t, *J* = 6.9 Hz, 3 H). ^{13}C NMR (101 MHz, $CDCl_3$) δ 158.7, 129.4, 120.8, 114.8 (t, *J* = 231.2 Hz), 114.4, 89.1 (t, *J* = 6.6 Hz), 73.7 (t, *J* = 40.2 Hz), 66.5, 36.3 (t, *J* = 27.3 Hz), 31.2, 28.4, 27.7 (t, *J* = 1.8 Hz), 23.2 (t, *J* = 3.7 Hz), 22.5, 18.3 (t, *J* = 2.2 Hz), 14.0. ^{19}F NMR (376 MHz, $CDCl_3$) δ -81.2 (tt, *J* = 14.7, 5.1 Hz). IR (thin film) ν_{max} 3063, 2932, 2860, 2251, 1601, 1498, 1245, 1016 cm^{-1}. MS (EI): m/z (%) 294 (M^+), 126, 100 (100), 94, 77. HRMS: Calculated for $C_{18}H_{24}OF_2$: 294.1795; Found: 294.1796.

Ethyl-5,5-difluorotridec-6-ynoate (4i)

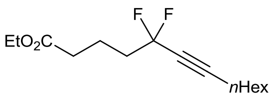

The product (63 mg, 77% yield) was purified with silica gel chromatography (Petroleum ether/EtOAc = 30:1) as a colorless oil. ^{1}H NMR (400 MHz, $CDCl_3$) δ 4.14 (q, *J* = 7.1 Hz, 2 H), 2.38 (t, *J* = 7.4 Hz, 2 H), 2.30 – 2.22 (m, 2 H), 2.12 – 1.99 (m, 2 H), 1.94 – 1.83 (m, 2 H), 1.59 – 1.49 (m,2 H), 1.43 – 1.33 (m, 2 H), 1.34 – 1.22 (m, 7 H), 0.89 (t, *J* = 6.9 Hz, 3 H). ^{13}C NMR (101 MHz, $CDCl_3$) δ 172.7, 114.6 (t, *J* = 231.3 Hz), 89.0 (t, *J* = 6.6 Hz), 73.6 (t, *J* = 40.0 Hz), 60.3, 38.6 (t, *J* = 27.1 Hz), 33.2, 31.1, 28.4, 27.7 (t, *J* = 1.9 Hz), 22.4, 18.5 (t, *J* = 3.9 Hz), 18.3 (t, *J* = 2.3 Hz), 14.1, 13.9. ^{19}F NMR (376 MHz, $CDCl_3$) δ -81.1 (tt, *J* = 14.4, 5.1 Hz). IR (thin film) ν_{max} 2933, 2860, 2252, 1736, 1168, 970 cm^{-1}. MS (EI): m/z (%) 255 (M-F^+), 205, 137, 111 (100), 97. HRMS: Calculated for $C_{15}H_{24}O_2F$: 255.1760; Found: 255.1758.

7,7-Difluorodec-5-yn-1-yl acetate (4j)

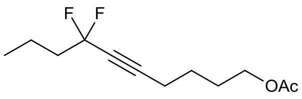

[Known compound[10]] The product (50 mg, 72% yield) was purified with silica gel chromatography

(Petroleum ether/EtOAc = 30:1) as a colorless oil. ^{1}H NMR (400 MHz, $CDCl_3$) δ 4.08 (t, *J* = 6.3 Hz, 2 H), 2.35 – 2.24 (m, 2 H), 2.05 (s, 3 H), 2.03 – 1.91 (m, 2 H), 1.78 – 1.69 (m, 2 H), 1.68 – 1.51 (m, 4 H), 0.98 (t, *J* = 7.4 Hz, 3 H). ^{13}C NMR (101 MHz, $CDCl_3$) δ 171.1, 114.9 (t, *J* = 231.1 Hz), 87.6 (t, *J* = 6.6 Hz), 74.5 (t, *J* = 40.4 Hz), 63.7, 41.3 (t, *J* = 26.2 Hz), 27.7, 24.4 (t, *J* = 2.0 Hz), 20.9, 18.0 (t, *J* = 2.3 Hz), 16.4 (t, *J* = 3.9 Hz), 13.6. ^{19}F NMR (376 MHz, $CDCl_3$) δ -81.2 (tt, *J* = 14.8, 4.9 Hz, 2 F).

(8*R*,9*S*,13*S*,14*S*,17*S*)-17-(3,3-Difluoro-6-phenylhex-1-yn-1-yl)-3,17-dimethoxy-13-methyl-7,8,9,11,12,13,14,15,16,17-decahydro-6H-cyclopenta[*a*]phenanthrene (4k)

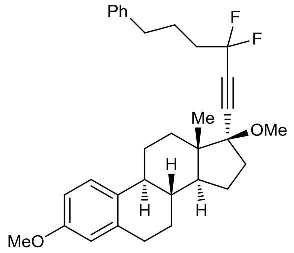

The product (133 mg, 90% yield) was purified with silica gel chromatography (Petroleum ether/EtOAc = 50:1) as a colorless oil. ^{1}H NMR (400 MHz, $CDCl_3$) δ 7.29 – 7.14 (m, 6 H), 6.73 (dd, *J* = 8.6, 2.3 Hz, 1 H), 6.65 (d, *J* = 1.9 Hz, 1 H), 3.80 (s, 3 H), 3.40 (s, 3 H), 2.92 – 2.80 (m, 2 H), 2.71 (t, *J* = 7.6 Hz, 2 H), 2.38 – 2.22 (m, 2 H), 2.22 – 2.00 (m, 4 H), 2.00 – 1.90 (m, 2 H), 1.89 – 1.75 (m, 4 H), 1.72 – 1.62 (m, 1 H), 1.55 – 1.39 (m, 3 H), 1.38 – 1.25 (m, 1 H), 0.89 (s, 3 H). ^{13}C NMR (101 MHz, $CDCl_3$) δ 157.4, 141.1, 137.8, 132.3, 128.4, 128.3, 126.3, 126.1, 114.9 (t, *J* = 232.4 Hz), 113.7, 111.5, 88.6 (t, *J* = 6.7 Hz), 85.6 (t, *J* = 1.7 Hz), 81.0 (t, *J* = 40.7 Hz), 55.2, 53.6, 49.8, 47.9, 43.5, 39.1, 38.9 (t, *J* = 26.5 Hz), 36.1, 34.9, 34.2, 29.8, 27.2, 26.4, 24.9 (t, *J* = 3.4 Hz), 22.7, 12.7. ^{19}F NMR (376 MHz, $CDCl_3$) δ -81.4 (t, *J* = 14.4 Hz). IR (thin film) ν_{max} 3085, 2934, 2242, 1610, 1499, 1256, 1095, 862 cm^{-1}. MS (EI): m/z (%) 492 (M^+), 477, 242, 227 (100), 174, 147, 91. HRMS: Calculated for $C_{32}H_{38}O_2F_2$: 492.2836; Found: 492.2840.

Ethyl-7-((8*R*,9*S*,13*S*,14*S*,17*S*)-3,17-dimethoxy-13-methyl-7,8,9,11,12,13,14,15,16,17-decahydro-6H-cyclopenta[*a*]phenanthren-17-yl)-5,5-difluorohept-6-ynoate (4l)

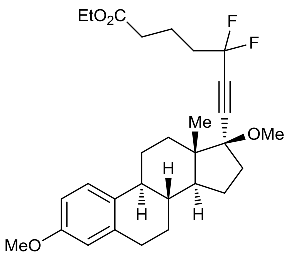

The product (122 mg, 83% yield) was purified with silica gel chromatography (Petroleum ether/EtOAc = 10:1) as a colorless oil. ^{1}H NMR (400 MHz, $CDCl_3$) δ 7.21 (d, *J* = 8.6 Hz, 1 H), 6.72 (dd, *J* = 8.5, 2.2 Hz, 1 H), 6.64 (s, 1 H), 4.12 (q, *J* = 7.1 Hz, 2 H), 3.78 (s, 3 H), 3.42 (s, 3 H), 2.85 (s, 2 H), 2.41 (t, *J* = 7.3 Hz, 2 H), 2.36 – 2.08 (m, 4 H), 2.08 – 1.95 (m, 2 H), 1.95 – 1.89 (m, 2 H), 1.89 – 1.78 (m, 3 H), 1.76 – 1.64 (m, 1 H), 1.56 – 1.35 (m, 4 H), 1.24 (t, *J* = 7.1 Hz, 3 H), 0.90 (s, 3 H). ^{13}C NMR (101 MHz, $CDCl_3$) δ 172.5, 157.4, 137.8, 132.2, 126.3, 114.6 (t, *J* = 232.5 Hz), 113.7, 111.4, 88.8 (t, *J* = 6.7 Hz), 85.5, 80.8 (t, *J* = 40.4 Hz), 60.4, 55.1, 53.6, 49.8, 47.8, 43.4, 39.1, 38.6 (t, *J* = 26.8 Hz), 36.1, 34.2, 33.1, 29.7, 27.2, 26.4, 22.7, 18.5 (t, *J* = 3.9 Hz), 14.1, 12.6. ^{19}F NMR (376 MHz, $CDCl_3$) δ -81.8 (t, *J* = 14.5 Hz). IR (thin film) ν_{max} 2934, 2832, 2243, 1735, 1610, 1500, 1256, 1168, 1039 cm^{-1}. MS (EI): m/z (%) 488 (M^+), 456, 242, 227 (100), 174, 147. HRMS: Calculated for $C_{29}H_{38}O_4F_2$: 488.2738; Found: 488.2730.

(3a*R*,5*R*,6*S*,6a*R*)-6-((4,4-Difluoro-7-phenylhept-2-yn-1-yl)oxy)-5-((*R*)-2,2-dimethyl-1,3-dioxolan-4-yl)-2,2-dimethyltetrahydrofuro[2,3-*d*][1,3]dioxole (4m)

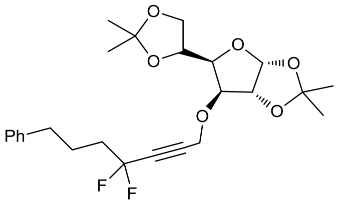

The product (74 mg, 53% yield) was purified with silica gel chromatography (Petroleum ether/EtOAc = 5:1) as a colorless oil. ^{1}H NMR (400 MHz, $CDCl_3$) δ 7.30 (t, *J* = 7.3 Hz, 2 H), 7.23 – 7.17 (m, 3 H), 5.87 (d, *J* = 3.7 Hz, 1 H), 4.60 (d, *J* = 3.7 Hz, 1 H), 4.36 (t, *J* = 4.1 Hz, 2 H), 4.30 – 4.21 (m, 1 H), 4.10 (dd, *J* = 8.1, 3.2 Hz, 1 H), 4.05 (d, *J* = 2.9 Hz, 1 H), AB part of ABX-system (δ_A = 4.09, δ_B = 4.09, J_{AB} = 8.6 Hz, J_{AX} = 6.0 Hz, J_{BX} = 5.2 Hz, 2 H), 2.70 (t, *J* = 7.6 Hz, 2 H), 2.14 – 2.01 (m, 2 H), 1.96 – 1.86 (m, 2 H), 1.50 (s, 3 H), 1.42 (s, 3 H), 1.34 (s, 3 H), 1.30 (s, 3 H). ^{13}C NMR

(101 MHz, $CDCl_3$) δ 141.0, 128.4, 128.3, 126.1, 114.5 (t, *J* = 232.9 Hz), 111.9, 109.1, 105.2, 82.7 (t, *J* = 6.6 Hz), 82.7, 81.9, 81.0, 72.3, 67.3, 57.7 (t, *J* = 1.8 Hz), 38.5 (t, *J* = 26.0 Hz), 34.9, 26.8, 26.7, 26.1, 25.3, 24.3 (t, *J* = 3.4 Hz). ^{19}F NMR (376 MHz, $CDCl_3$) δ -83.1 (tt, J = 14.8, 4.1 Hz). IR (thin film) ν_{max} 3027, 2934, 2256, 2011, 1604, 1455, 1373, 1216, 1085 cm^{-1}. MS (DART): m/z (%) 484 ($M+NH_4^+$), 467($M+H^+$), 409, 205. HRMS: Calculated for $C_{25}H_{33}O_6F_2$: 467.2240; Found: 467.2235.

1-(*tert*-Butyl)-2-(6,6-difluoro-9-phenylnon-4-yn-1-yl) pyrrolidine-1,2-dicarboxylate (4n)

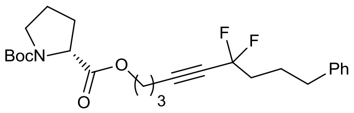

The product (101 mg, 75% yield) was purified with silica gel chromatography (Petroleum ether/EtOAc = 5:1) as a colorless oil. ^1H NMR (400 MHz, $CDCl_3$) δ 7.29 (t, *J* = 7.4 Hz, 2 H), 7.22 – 7.15 (m, 3 H), 4.33 – 4.12 (m, 3 H), 3.58 – 3.34 (m, 2 H), 2.67 (t, *J* = 7.5 Hz, 2 H), 2.40 – 2.32 (m, 2 H), 2.28 – 2.13 (m, 1 H), 2.08 – 1.81 (m, 9 H), 1.45 and 1.40 (s, 9 H, rotamer). ^{13}C NMR (101 MHz, $CDCl_3$) rotameric mixture, resonances for minor rotamer are enclosed in parenthesis: δ 173.0 (172.7), 154.3 (153.6), 141.2 (141.1), 128.33 (128.28), 125.96, (125.92), 114.68 (114.60) (t, *J* = 231.4), 87.1 (86.6) (t, *J* = 6.4 Hz), 79.8 (79.7), 74.7 (74.4) (t, *J* = 40.3 Hz), 63.05 (63.01), 59.0 (58.7), 46.4 (46.2), 38.7 (t, *J* = 26.5 Hz), 34.9, 30.8 (30.0), 28.3 (28.2), 27.0 (26.9), 24.4 (24.2), 23.5, 15.08 (15.06). ^{19}F NMR (376 MHz, $CDCl_3$) δ -81.3 – -81.5 (m, 2 F). IR (thin film) ν_{max} 3450, 3063, 2973, 2252, 2003, 1747, 1669, 1478, 1398, 1162 cm^{-1}. MS (EI): m/z (%) 467($M+NH_4^+$), 451, 450 (100). HRMS: Calculated for $C_{25}H_{34}O_4NF_2$: 450.2450; Found: 450.2449.

6,6-Difluoro-9-phenylnon-4-yn-1-yl-(2*S*,5*R*)-3,3-dimethyl-7-oxo-4-thia-1-azabicyclo[3.2.0]heptane-2-carboxylate 4,4-dioxide (4o)

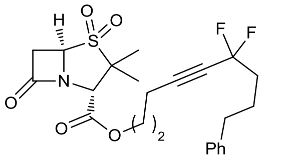

The product (101 mg, 72% yield) was purified with silica gel chromatography (Petroleum ether/EtOAc = 3:1) as a colorless oil. ^1H NMR (400 MHz, $CDCl_3$) δ 7.32 – 7.27 (m, 2 H), 7.23 – 7.15 (m, 3 H), 4.61 (dd, *J* = 4.1, 2.2 Hz, 1 H), 4.39 (s, 1 H), 4.34 – 4.22 (m, 2 H), 3.53 – 3.39 (m, 2 H), 2.68 (t, *J* = 7.6 Hz, 2 H), 2.44 – 2.36 (m, 2 H), 2.10 – 1.81 (m, 6 H), 1.60 (s, 3 H), 1.40 (s, 3 H).

^{13}C NMR (101 MHz, $CDCl_3$) δ 170.7, 166.8, 141.1, 128.4, 128.3, 126.0, 114.6 (t, *J* = 231.8 Hz), 86.0 (t, *J* = 6.6 Hz), 75.2 (t, *J* = 40.5 Hz), 64.5, 63.1, 62.6, 61.0, 38.6 (t, *J* = 26.4 Hz), 38.3, 34.9, 26.4 (t, *J* = 1.7 Hz), 24.4 (t, *J* = 3.4 Hz), 20.3, 18.5, 15.1 (t, *J* = 2.0 Hz). ^{19}F NMR (376 MHz, $CDCl_3$) δ -81.39 (tt, *J* = 14.7, 4.8 Hz) .IR (thin film) ν_{max} 3027, 2934, 2253, 1798, 1756, 1463, 1322, 1190 cm^{-1}. MS (DART): m/z (%) 468 ($M+NH_4{}^+$), 486, 485 (100), 468. HRMS: Calculated for $C_{23}H_{28}O_5NF_2S$: 468.1651; Found: 468.1650.

Transformations of Compounds 3 and 4

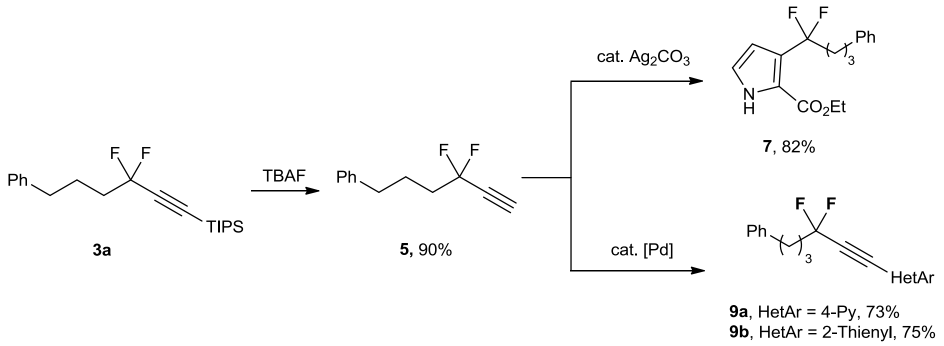

(4,4-Difluorohex-5-yn-1-yl)benzene (5) [11]

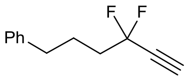

To a solution of compound **3a** (0.5 mmol) in THF (5 mL) was added TBAF (1.0 M in THF, 0.6 mL, 0.6 mmol) at -78 °C. The reaction mixture was stirred at same reaction temperature for 1 h and allowed to warm to -20 °C slowly. The reaction was then quenched with 20 mL of saturated aqueous NH_4Cl solution. After stirring for 5 min at 0 °C, the resulting mixture was extracted with diethyl ether (three times). The combined organic layers were washed with brine, dried over Na_2SO_4, filtered, and concentrated. The residue was purified by flash silica gel chromatography (Petroleum ether) to give alkyne **5** (87 mg, 90% yield) as a colorless oil. ^1H NMR (400 MHz, $CDCl_3$) δ 7.34 – 7.28 (m, 2 H), 7.25 – 7.17 (m, 3 H), 2.75 (t, *J* = 5.0 Hz, 1 H), 2.70 (t, *J* = 7.6 Hz, 2 H), 2.14 – 2.01 (m, 2 H), 1.97 – 1.87 (m, 2 H). ^{13}C NMR (101 MHz, $CDCl_3$) δ 141.1, 128.5, 128.4, 126.1, 114.2 (t, *J* = 233.0 Hz), 76.5 (t, *J* = 41.1 Hz), 75.2 (t, *J* = 6.9 Hz), 38.4 (t, *J* = 25.7 Hz), 34.9, 24.3 (t, *J* = 3.6 Hz). ^{19}F NMR (376 MHz, $CDCl_3$) δ -83.9 (td, *J* = 14.8, 4.9 Hz). IR (thin film) ν_{max} 2934, 2861, 2253, 1457, 1267, 1169 cm^{-1}. MS (EI): m/z (%) 194, 117, 91 (100), 77, 65. HRMS: Calculated for $C_{12}H_{12}F_2$: 194.0907; Found: 194.0913.

Ethyl 3-(1,1-difluoro-4-phenylbutyl)-1H-pyrrole-2-carboxylate (7) [12]

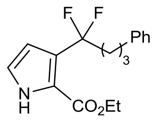

To a solution of compound **5** (88 mg, 0.45 mmol) and Ag_2CO_3 (8.3 mg, 0.03 mmol) in 1,4-dioxane (2 mL) was added a solution of ethyl isocyanoacetate (34 mg, 0.3 mmol) in 1,4-dioxane (1 mL) slowly at 80 ^{o}C. The reaction mixture was then stirred at same reaction temperature for 6 h. The reaction mixture was concentrated and redissovled in CH_2Cl_2. The organic layer was washed with brine, dried over $NaSO_4$, filtered, and concentrated. The residue was purified with flash silica gel column chromatography (Petroleum ether/ EtOAc = 10: 1) to give compound **7** (76 mg, 82%) as a faint yellow oil. ^{1}H NMR (500 MHz, $CDCl_3$) δ 9.42 (s, 1 H), 7.29 – 7.24 (m, 2 H), 7.20 – 7.13 (m, 3 H), 6.86 (t, J = 2.8 Hz, 1 H), 6.43 (t, J = 2.8 Hz, 1 H), 4.29 (q, J = 7.1 Hz, 2 H), 2.65 (t, J = 7.7 Hz, 2 H), 2.46 – 2.34 (m, 2 H), 1.84 – 1.76 (m, 2 H), 1.31 (t, J = 7.1 Hz, 3 H). ^{13}C NMR (101 MHz, $CDCl_3$) δ 160.0, 141.7, 128.3, 128.3, 126.8 (t, J = 32.2 Hz), 125.8, 121.8 (t, J = 238.9 Hz), 120.9, 118.8 (t, J = 5.6 Hz), 110.4 (t, J = 6.7 Hz), 60.8, 37.3 (t, J = 27.0 Hz), 35.2, 24.3 (t, J = 3.7 Hz), 14.0. ^{19}F NMR (376 MHz, $CDCl_3$) δ -89.1 (t, J = 16.6 Hz). IR (thin film) ν_{max} 3311, 2982, 1682, 1603, 1497, 1287, 1141, 950 cm^{-1}. MS (EI): m/z (%) 307 (M^+), 267, 214, 183 (100), 139, 91. HRMS: Calculated for $C_{17}H_{19}NO_2F_2$: 307.1384; Found: 307.1383.

Sonogashira reaction of 5 with heteroaryl iodides

To a septum capped 25 mL of Schlenck tube were added $PdCl_2(PPh_3)_2$ (5 mol %) and CuI (10 mol %) under Ar. *i*Pr_2NH (3 mmol, 10.0 equiv), HetAr-I (0.39 mmol, 1.3 equiv) and THF (5 mL) were then added. After the reaction mixture was stirred at room temperature for 15 min, alkyne **5** (0.3 mmol, 1.0 equiv) was added. The reaction mixture was stirred for 16 h and concentrated. The crude product was purified by silica gel chromatography to give product **9**.

4-(3,3-Difluoro-6-phenylhex-1-yn-1-yl)pyridine (9a)

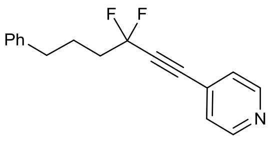

The product (59 mg, 73% yield) was purified with silica gel chromatography (Petroleum

ether/EtOAc = 5:1) as a faint yellow oil. ^{1}H NMR (400 MHz, $CDCl_3$) δ 8.67 – 8.62 (m, 2 H), 7.37– 7.34 (m, 2 H), 7.33 – 7.28 (m, 2 H), 7.25 – 7.17 (m, 3 H), 2.73 (t, *J* = 7.6 Hz, 2 H), 2.24 – 2.09 (m, 2 H), 2.02 – 1.91 (m, 2 H). ^{13}C NMR (101 MHz, $CDCl_3$) δ 1450.0, 141.0, 128.5, 128.3, 126.1, 125.7 (t, *J* = 1.9 Hz), 114.8 (t, *J* = 233.6 Hz), 85.6 (t, *J* = 41.3 Hz), 83.4 (t, *J* = 6.6 Hz), 38.5 (t, *J* = 26.0 Hz), 34.9, 24.32 (t, *J* = 3.3 Hz). ^{19}F NMR (376 MHz, $CDCl_3$) δ -83.4 (t, *J* = 14.8 Hz). IR (thin film) ν_{max} 3063, 2860, 2249, 1593, 1454, 1323, 1168, 990 cm^{-1}. MS (EI): m/z (%) 271 (M^+), 206, 117 (100), 91, 65. HRMS: Calculated for $C_{17}H_{15}NF_2$: 271.1173; Found: 271.1167.

2-(3,3-Difluoro-6-phenylhex-1-yn-1-yl)thiophene (9b)

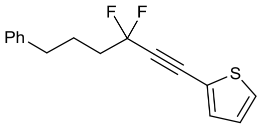

The product (62 mg, 75% yield) was purified with silica gel chromatography (Petroleum ether/EtOAc = 50:1) as a yellow oil. ^{1}H NMR (400 MHz, $CDCl_3$) δ 7.37 – 7.26 (m, 4 H), 7.23 – 7.17 (m, 3 H), 7.01 – 6.97 (m, 1 H), 2.71 (t, *J* = 7.6 Hz, 2 H), 2.21 – 2.07 (m, 2 H), 2.00 – 1.90 (m, 2 H). ^{13}C NMR (101 MHz, $CDCl_3$) δ 141.2, 134.3 (t, *J* = 2.2 Hz), 129.2, 128.4, 128.4, 127.2, 126.1, 119.8 (t, *J* = 3.1 Hz), 115.3 (t, *J* = 232.7 Hz), 85.3 (t, *J* = 41.0 Hz), 80.4 (t, *J* = 6.9 Hz), 38.7 (t, *J* = 26.4 Hz), 34.9, 24.4 (t, *J* = 3.3 Hz). ^{19}F NMR (376 MHz, $CDCl_3$) δ -82.3 (t, *J* = 14.6 Hz). IR (thin film) ν_{max} 3107, 2930, 2234, 1603, 1497, 1454, 1310, 1099, 978 cm^{-1}. MS (EI): m/z (%) 276 (M^+), 198, 159 (100), 117, 91. HRMS: Calculated for $C_{16}H_{14}F_2S$: 276.0784; Found: 276.0777.

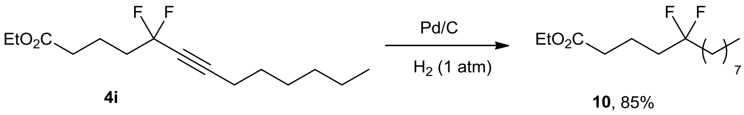

Ethyl 5,5-difluorotridecanoate (10)

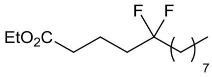

A mixture of Pd/C (10%, 50 mg) and **4i** (156 mg, 0.56 mmol) in 20 mL of DCM was stirred under 1 atm H_2 atmosphere at room temperature for 16 h. The reaction mixture was filtered. The filtrate was washed with brine, dried over Na_2SO_4 and concentrated. The product **10** (133 mg, 85% yield) was purified with silica gel chromatography (Petroleum ether/EtOAc = 50:1) as a colorless oil. ^{1}H NMR (400 MHz, $CDCl_3$) δ 4.12 (q, *J* = 7.1 Hz, 2 H), 2.34 (t, *J* = 6.9 Hz, 2 H), 1.91 – 1.65 (m, 5 H), 1.49 –

1.38 (m, 2 H), 1.35 – 1.15 (m, 14 H), 0.87 (t, *J* = 6.7 Hz, 3 H). ^{13}C NMR (126 MHz, $CDCl_3$) δ 173.0, 125.0 (t, *J* = 240.3 Hz), 60.3, 36.3 (t, *J* = 25.2 Hz), 35.5 (t, *J* = 25.8 Hz), 33.7, 31.8, 29.3, 29.3, 29.1, 22.6, 22.3 (t, *J* = 4.6 Hz), 17.9 (t, *J* = 5.0 Hz), 14.2, 14.0. ^{19}F NMR (376 MHz, $CDCl_3$) δ -98.09 (p, *J* = 16.2 Hz, 2 F). MS (EI): m/z (%) 258 $(M-HF)^+$, 193, 150, 88(100), 55. HRMS: Calculated for $C_{15}H_{27}O_2F$: 258.1995; Found: 258.1984.

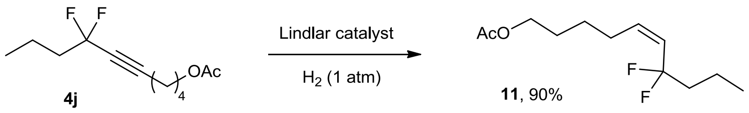

(Z)-7,7-Difluorodec-5-en-1-yl acetate (11)

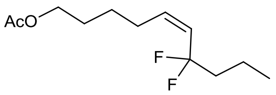

[Known compoud[13]] A mixture of Pd-$BaSO_4$ (100 mg) and quinoline (20 mg) and **4j** (110 mg, 0.47 mmol) in 10 mL of pyridine was stirred under 1 atm H_2 atmosphere H_2 at room temperature for 24 h. The reaction mixture was filtered. The filtrate was washed with saturated $CuSO_4$ solution, dried over Na_2SO_4 and concentrated. The product **11** (99 mg, 90% yield) was purified with silica gel chromatography (Petroleum ether/EtOAc = 30:1) as a colorless oil. ^{1}H NMR (400 MHz, $CDCl_3$) δ 5.81 – 5.60 (m, 1 H), 5.53 – 5.41 (m, 1 H), 4.06 (t, *J* = 6.6 Hz, 2 H), 2.35 – 2.17 (m, 2 H), 2.04 (s, 3 H), 1.95 – 1.80 (m, 2 H), 1.76 – 1.57 (m, 2 H), 1.57 – 1.40 (m, 4 H), 0.96 (t, *J* = 7.4 Hz, 3 H). ^{13}C NMR (101 MHz, $CDCl_3$) δ 171.2, 137.1 (t, *J* = 6.0 Hz), 125.3 (t, *J* = 27.0 Hz), 122.6 (t, *J* = 239.1 Hz), 64.2, 40.5 (t, *J* = 26.5 Hz), 28.1, 27.8 (t, *J* = 1.9 Hz), 25.8 (t, *J* = 1.4 Hz), 20.9, 15.8 (t, *J* = 4.4 Hz), 13.9. ^{19}F NMR (376 MHz, $CDCl_3$) δ -90.70 – -90.88 (m, 2 F).

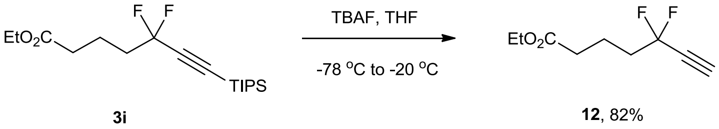

Ethyl 5,5-difluorohept-6-ynoate (12)

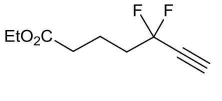

The product **12** was prepared according to the procedure of synthesis of compound **5**. Compound **12** (78 mg, 82% yield) was purified with silica gel chromatography (Petroleum ether/Et_2O = 9:1) as a yellow oil. ^{1}H NMR (400 MHz, $CDCl_3$) δ 4.14 (q, *J* = 7.1 Hz, 2 H), 2.78 (t, *J* = 4.9 Hz, 1 H), 2.39 (t,

J = 7.3 Hz, 2 H), 2.21 – 2.03 (m, 2 H), 1.97 – 1.82 (m, 2 H), 1.26 (t, *J* = 7.1 Hz, 3 H). ^{13}C NMR (101 MHz, $CDCl_3$) δ 172.7, 113.9 (t, *J* = 233.1 Hz), 76.2 (t, *J* = 41.0 Hz), 60.5, 38.2 (t, *J* = 26.0 Hz), 33.1, 18.2 (t, *J* = 4.1 Hz), 14.2. ^{19}F NMR (376 MHz, $CDCl_3$) δ -84.2 (td, *J* = 14.8, 4.9 Hz). IR (thin film) ν_{max} 3303, 2983, 2133, 1733, 1377, 975 cm^{-1}. MS (EI): m/z (%) 190 (M^+), 171, 146, 117, 77 (100). HRMS: Calculated for $C_9H_{12}O_2F_2$: 190.0805; Found: 190.0809.

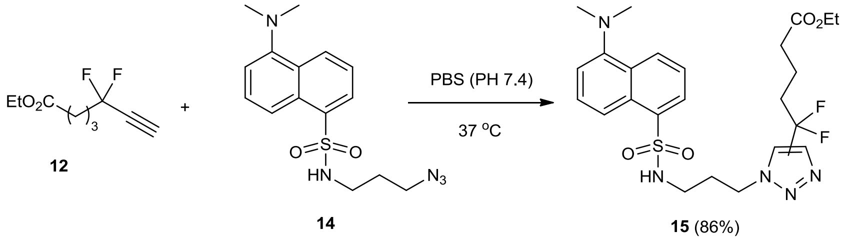

Procedure for the Copper-Free Click Chemistry

To a septum capped 25 mL of Schlenck tube were added terminal alkyne **12** (57 mg, 0.3 mmol), Dans $(CH_2)_3$-N_3 **14** (150 mg, 0.45 mmol), and phosphate buffered saline (PBS) (1 mL). The tube was screw capped and then vigorously stirred at 37 °C (oil bath) for 48 h. The reaction mixture was then diluted with water and extracted with EtOAc. The organic layers were dried over Na_2SO_4, filtered and concentrated. The resulting crude product was purified by flash chromatography (Pentane: EtOAc = 5:1 to 1:1) to afford the desired products **15** (135 mg, 86% yield) as a mixture of regioisomers. ^{1}H NMR (400 MHz, $CDCl_3$) δ 8.52 (d, *J* = 8.4 Hz, 1 H), 8.28 (d, *J* = 8.6 Hz, 1 H), 8.22 – 8.16 (m, 1 H), 7.77 (s, 0.23 H), 7.58 (s, 0.67 H), 7.56 – 7.43 (m, 2 H), 7.15 (d, *J* = 7.5 Hz, 1 H), 5.79 (t, *J* = 6.2 Hz, 0.68 H), 5.62 (t, *J* = 6.3 Hz, 0.24 H), 4.44 (t, *J* = 6.8 Hz, 0.5 H), 4.35 (t, *J* = 6.6 Hz, 1.42 H), 4.16 – 4.06 (m, 2 H), 3.01 – 2.78 (m, 8 H), 2.44 – 2.23 (m, 4 H), 2.11 – 1.95 (m, 2 H), 1.86 – 1.74 (m, 2 H), 1.27 – 1.18 (m, 3 H). ^{13}C NMR (101 MHz, $CDCl_3$) δ 172.8, 172.6, 151.85, 151.76, 144.2 (t, *J* = 33.0 Hz), 134.4, 134.1, 133.3 (t, *J* = 4.2 Hz), 132.5, 130.5, 130.3, 129.65, 129.63, 129.4, 129.3, 129.2, 128.35, 128.31, 123.06, 123.01, 122.6 (t, *J* = 2.6 Hz), 119.0 (t, *J* = 236.6 Hz), 118.5, 118.3, 117.8 (t, *J* = 238.2 Hz), 115.11, 115.08, 60.5, 60.3, 47.1, 46.9, 45.19, 45.16, 40.0, 39.6, 35.85 (t, *J* = 25.4 Hz), 35.80 (t, *J* = 24.8 Hz), 33.3, 32.6, 29.8, 29.7, 17.5 (t, *J* = 4.2 Hz), 17.3 (t, *J* = 4.0 Hz), 14.0. ^{19}F NMR (376 MHz, $CDCl_3$) δ -91.7 (t, *J* = 16.5 Hz), -91.9 (t, *J* = 16.6 Hz). MS (DART): m/z (%) 541 (M + NH_4^+), 524 (M + H^+, 100), 523 (M^+), 509. HRMS: Calculated for $C_{24}H_{32}O_4N_5F_2S$: 524.2138; Found: 524.2137.

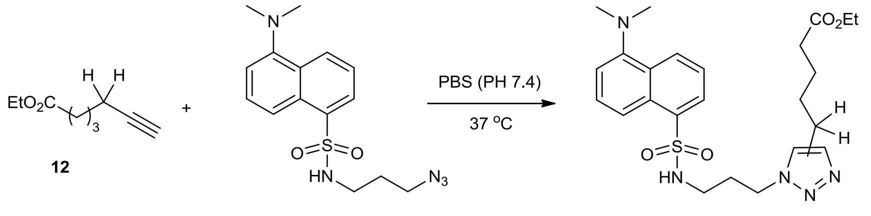

To a septum capped 25 mL of Schlenck tube were added terminal alkyne **12** (47 mg, 0.3 mmol), Dans $(CH_2)_3$-N_3 **14** (150 mg, 0.45 mmol), and phosphate buffered saline (PBS) (1 mL) subsequently. The tube was screw capped and then vigorously stirred at 37 °C (oil bath) for 48 h. The reaction mixture was then diluted with water and extracted with EtOAc. The organic layers were dried over $NaSO_4$, filtered and concentrated. The resulting crude product was purified by flash chromatography, only the starting material Dans $(CH_2)_3$-N_3 **14** was recovered, and no cycloaddition products **15′** were obtained.

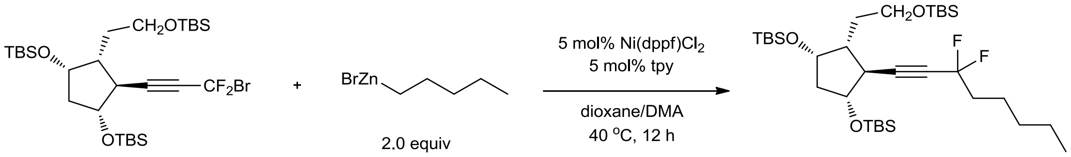

(((1*R*,3*S*,4*R*,5*S*)-4-(2-((*tert*-butyldimethylsilyl)oxy)ethyl)-5-(3,3-difluorooct-1-yn-1-yl)cyclopentane-1,3-diyl)bis(oxy))bis(*tert*-butyldimethylsilane) (17)

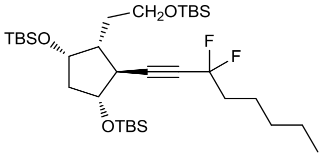

To a 25 mL of Schlenk tube was added Ni(dppf)Cl_2 (6.8 mg, 5 mol %) and Tpy (2.3 mg, 5 mol %). The tube was evacuated and backfilled with Ar for 3 times, then **16** (129 mg, 0.2 mmol, 1.0 equiv) and 1,4-dioxane (1.5 mL) were added. The resulting mixture was stirred at room temperature for 5 min, pentylzinc bromide (0.4 mmol, 1.2 equiv in 0.75 mL of DMA) was added dropwise over a period of 5 min. The tube was screw capped and put into a preheated oil bath (40 °C). After stirring for 12 h, the reaction mixture was cooled to room temperature and diluted with EtOAc. The resulting mixture was filtered with a pad of cellite. The filtrate was washed with water, the organic layer was dried over Na_2SO_4, filtered, and concentrated. The residue was purified with silica gel chromatography (Petroleum ether/EtOAc = 100:1) to give the pure product **17** as a colorless oil. ^1H NMR (500 MHz, $CDCl_3$) δ 4.20 (ddd, J = 8.7, 7.3, 4.0 Hz, 1 H), 4.08 (t, J = 3.8 Hz, 1 H), 3.72 – 3.61

(m, 2 H), 2.76 – 2.69 (m, 1 H), 2.20 (ddd, *J* = 14.1, 8.8, 5.1 Hz, 1 H), 2.04 – 1.93 (m, 2 H), 1.90 – 1.68 (m, 3 H), 1.61 (dd, *J* = 3.9, 1.4 Hz, 1 H), 1.60 – 1.51 (m, 2 H), 1.37 – 1.29 (m, 4 H), 0.96 – 0.85 (m, 30 H), 0.09 (s, 3 H), 0.06 (s, 3 H), 0.06 – 0.04 (m, 9 H), 0.03 (s, 3 H). ^{13}C NMR (126 MHz, $CDCl_3$) δ 115.0 (t, *J* = 231.3 Hz), 90.4 (t, *J* = 6.6 Hz), 77.5, 75.7 (t, *J* = 40.4 Hz), 72.0, 61.2, 46.7, 44.7, 43.4 (t, *J* = 2.0 Hz), 39.5 (t, *J* = 26.5 Hz), 31.2, 30.8, 25.9, 25.7, 22.7 (t, *J* = 3.4 Hz), 22.4, 18.3, 18.0, 13.9, -4.2, -4.6, -4.9, -5.0, -5.2, -5.3. ^{19}F NMR (376 MHz, $CDCl_3$) δ -81.13 (td, *J* = 14.6, 4.6 Hz, 2 F). MS (DART): m/z (%) 633 (M+H$^+$, 100), 501. HRMS: Calculated for $C_{33}H_{67}O_3F_2Si_3$: 633.4361; Found: 633.4354.

Radical Inhibition Experiment

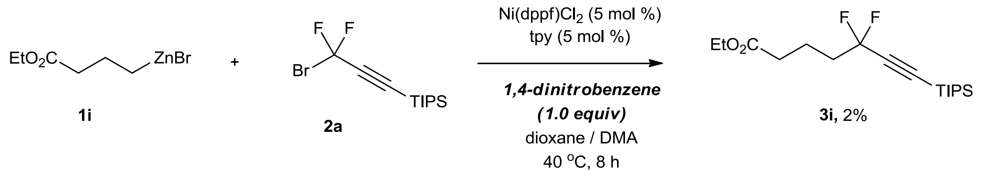

Procedure: To a 25 mL of Schlenk tube were added Ni(dppf)Cl_2 (10.3 mg, 5 mol %), Tpy (3.5 mg, 5 mol%), and 1,4-dinitrobenzene (50.4 mg, 0.3 mmol, 1.0 equiv) under air. The mixture was evacuated and backfilled with Ar for 3 times. *gem*-Difluoropropargyl bromide **2a** (0.3 mmol, 1 equiv) and 1,4-dioxane (2 mL) were added. The resulting mixture was stirred at room temperature for 5 min, (4-ethoxy-4-oxobutyl)zinc(II) bromide **1i** (0.36 mmol, 1.2 equiv in 1 mL of DMA) was added dropwise. The tube was screw capped and put into a preheated oil bath (40 °C). After stirring for 8 h, the reaction mixture was cooled to room temperature. The yield was determined by ^{19}F NMR.

Radical Clock Experiment

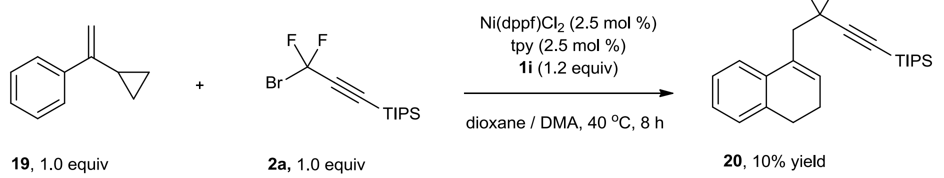

Procedure: To a 25 mL of Schlenk tube were added Ni(dppf)Cl_2 (5.2 mg, 2.5 mol %), Tpy (1.75 mg, 2.5 mol %). The mixture was evacuated and backfilled with Ar for 3 times. *gem*-Difluoropropargyl bromide **2a** (93.4 mg, 0.3 mmol, 1.0 equiv), (1-cyclopropylvinyl)benzene **19** (43.3 mg, 1.0 equiv)

and 1,4-dioxane (2 mL) were added. The resulting mixture was stirred at room temperature for 5 min, then (4-ethoxy-4-oxobutyl)zinc bromide **1i** (0.36 mmol, 1.2 equiv in DMA) was added dropwise. The tube was screw capped and put into a preheated oil bath (40 ^oC). After stirring for 8 h, the reaction mixture was cooled to room temperature. The yield was determined by ^{19}F NMR. The reaction mixture was diluted with EtOAc and filtered with a pad of cellite. The filtrate was washed with water (15 mL), the organic layer was dried over Na_2SO_4, filtered, and concentrated. The residue was purified with silica gel chromatography (Petroleum ether) to give compound **20** as a colorless oil. Data for compound **20**. ^1H NMR (400 MHz, $CDCl_3$) δ 7.30 (d, J = 7.5 Hz, 1 H), 7.22 – 7.15 (m, 1 H), 7.15 – 7.10 (m, 2 H), 6.16 (t, J = 4.5 Hz, 1 H), 3.20 (t, J = 14.3 Hz, 2 H), 2.76 (t, J = 8.0 Hz, 2 H), 2.30 (td, J = 8.0, 4.7 Hz, 2 H), 1.03 (s, 21 H). ^{13}C NMR (101 MHz, $CDCl_3$) δ 136.2, 134.3, 131.7, 128.2 (t, J = 4.2 Hz), 127.6, 126.9, 126.3, 123.1 (t, J = 1.4 Hz), 113.5 (t, J = 234.2 Hz), 98.8 (t, J = 38.5 Hz), 90.4 (t, J = 5.3 Hz), 41.5 (t, J = 27.5 Hz), 28.1, 23.3, 18.4, 10.9. ^{19}F NMR (376 MHz, $CDCl_3$) δ -79.15 (t, J = 14.2 Hz, 2 F). MS (DART): m/z (%) 392 ($M+NH_4^+$, 100), 375 ($M+H^+$), 355, 335. HRMS: Calculated for $C_{23}H_{33}F_2Si$: 375.2314; Found: 375.2313.

Reaction of PBN in the Presence of Ni(dppf)Cl₂, Tpy, 1i and 2a in 1,4-dioxane

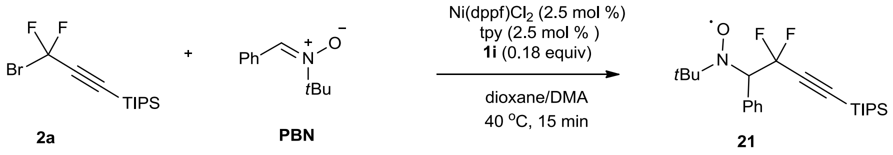

Procedure: A mixture of **2a** (18.7 mg, 0.06 mmol), Ni(dppf)Cl_2 (1.01 mg, 0.0015 mol), tpy (0.35 mg, 0.0015 mmol), and PBN (21 mg, 0.12 mmol) in dioxane (0.5 mL) was shaken under Ar for 1 min. Subsequently, **1i** (0.0108 mmol in 0.015 mL of DMA) was added to the reaction mixture. After heating at 40 ^oC (oil bath) for 25 min, the resulting mixture was then analyzed by EPR at 40 ^oC. The EPR showed an EPR sextet spectrum of nitroxide **21** (g = 2.00395, a_N = 14.10 G, a_H^β = 2.01 G) indicates that the difluoroalkyl group must be substituted at the β-carbon atom of nitroxide and the a_N and a_H^β values are decreased by the strong electron-withdrawing effect of $CF_2CCTIPs$ (Supplementary Figure. 168).

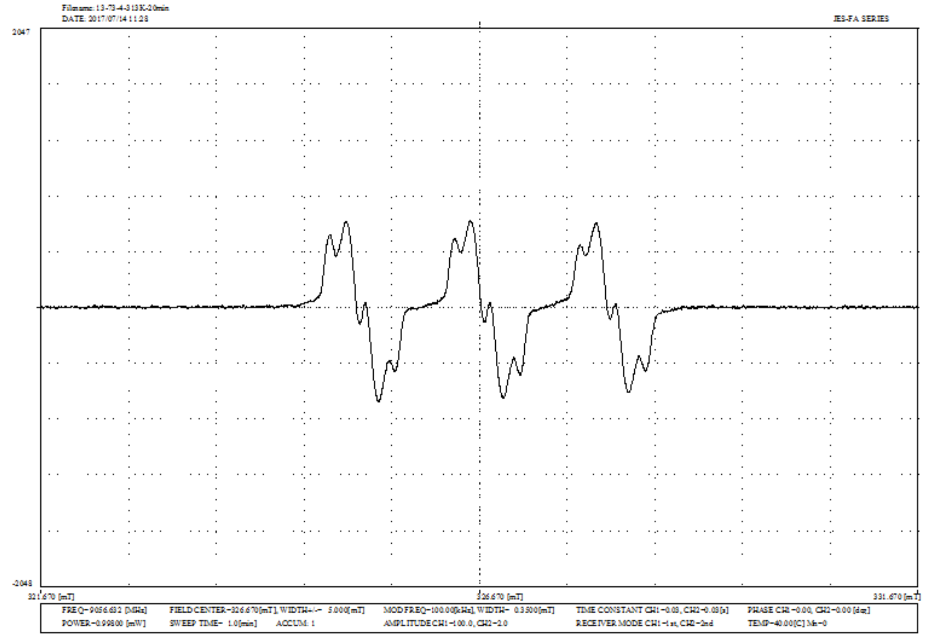

Supplementary Figure 168. The Electron Paramagnetic Resonance (EPR) Spectrum of a Mixture of PBN, Ni(dppf)Cl_2, Tpy, **1i** and **2a** in 1,4-dioxane at 40 ^oC.

Using EPR to Monitor the Reaction

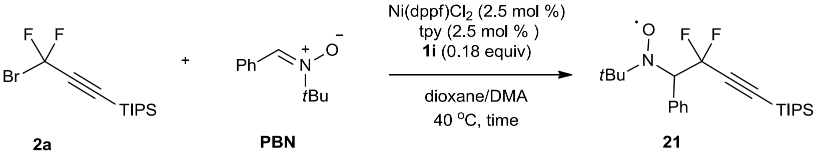

Procedure: A mixture of **2a** (18.7 mg, 0.06 mmol), Ni(dppf)Cl_2 (1.01 mg, 0.0015 mol), tpy (0.35 mg, 0.0015 mmol), and PBN (21 mg, 0.12 mmol) in dioxane (0.5 mL) was shaken under Ar for 1 min. Subsequently **1i** (0.0108 mmol in 0.015 mL of DMA) was added to the reaction mixture. The resulting mixture was monitored by EPR at 40 ^oC.

The reaction monitored by EPR showed the signal of spin adduct **21** at the beginning of the reaction, demonstrating that a *gem*-difluoropropargyl radical is generated during the reaction process (Supplementary Figure. 169).

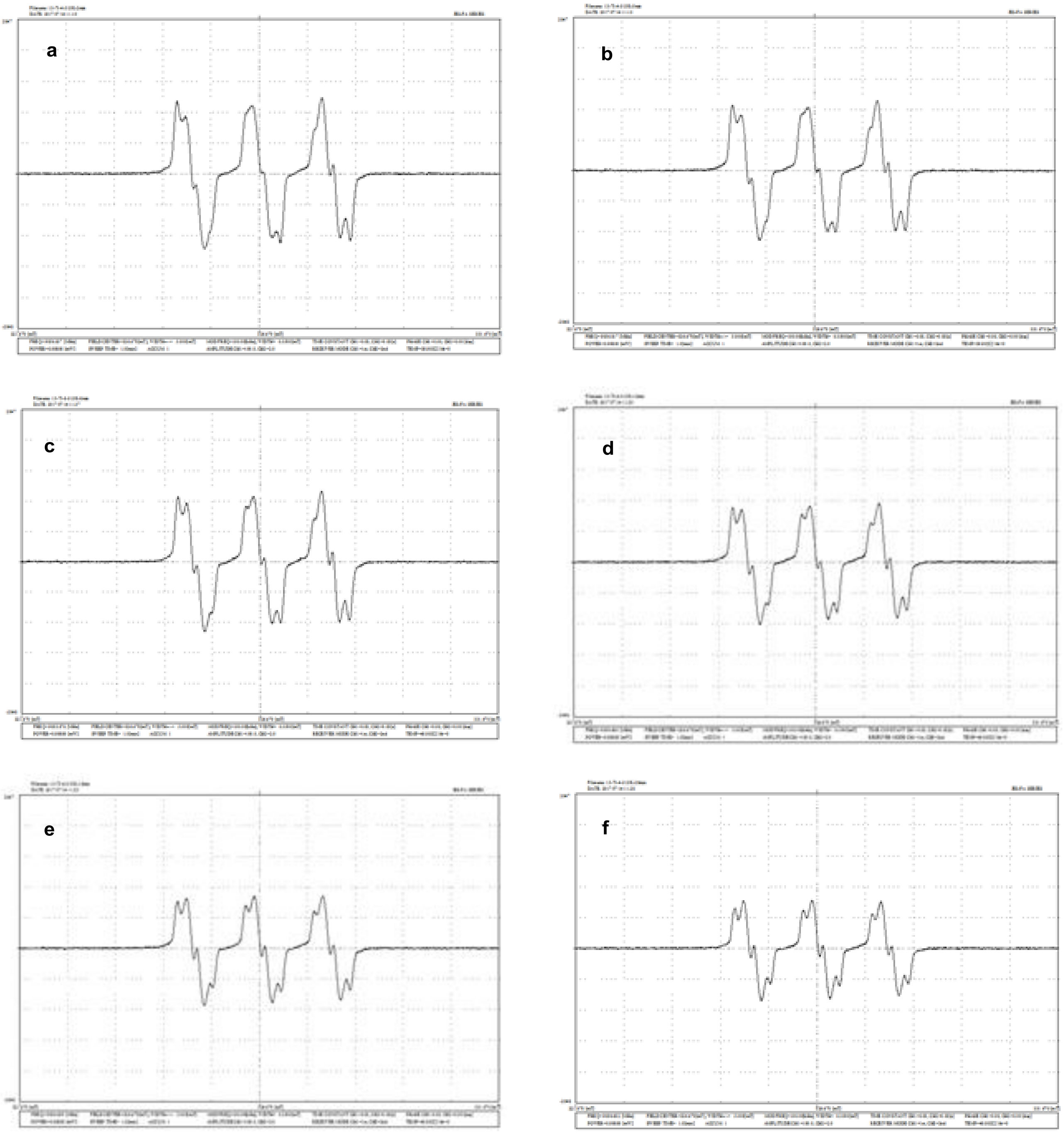

Supplementary Figure 169. Electron Paramagnetic Resonance (EPR) Spectrum of the mixture of PBN, Ni(dppf)Cl_2, Tpy, **1i** and **2a** in 1,4-dioxane at 40 ^{o}C from 2 to 25 minutes. **a** The reaction was monitored by EPR after 2 minutes. **b** The reaction was monitored by EPR after 5 minutes. **c** The reaction was monitored by EPR after 8 minutes. **d** The reaction was monitored by EPR after 10 minutes. **e** The reaction was monitored by EPR after 15 minutes. **f** The reaction was monitored by EPR after 25 minutes.

Preparation of Nickel(I) Complex [TpyNi^{I}-I] A1

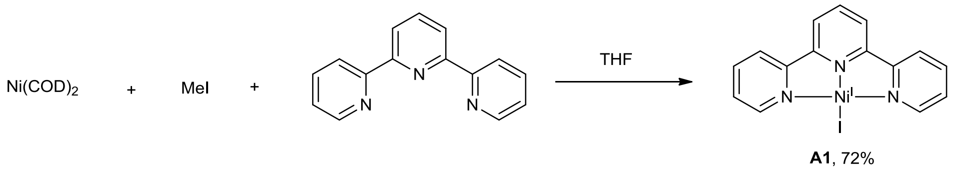

Procedure: Complex **A1** was prepared according to the literature.[2] In a glovebox, terpyridine (117 mg, 0.5 mmol) was added to a stirred solution of Ni(COD)$_2$ (138 mg, 0.5 mmol) in THF (20 mL). The resulting green solution was stirred at room temperature for 0.5 h. A solution of methyl iodide (32 μL) in 2 mL of THF was then added dropwise at room temperature. The resulting suspension was stirred for 4 h at room temperature. The olive green solid was then filtered, washed with THF and ether, and dried under vacuum to give complex **A1** (150 mg, 72 % yield). Anal. Calculated for $C_{15}H_{11}IN_3Ni$: C, 43.01; H, 2.65; N, 10.03. Found: C, 43.34; H, 2.93; N, 10.07.

Reaction of 1a with 2a using Nickel Complex A1 as a Catalyst

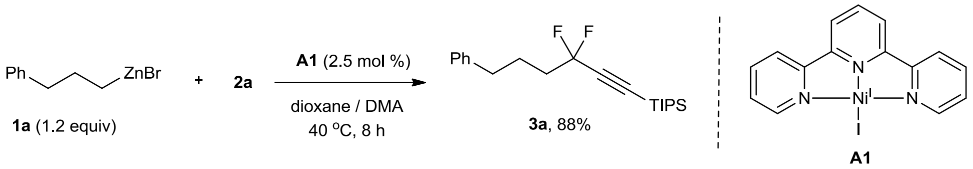

Procedure: In the glovebox, (Tpy)Ni^{I}-I (**A1**) (3.1 mg, 2.5 mol %) was added to a 25 mL of Schlenk tube. *gem*-Difluoropropargyl bromide **2a** (0.3 mmol, 1.0 equiv) and 1,4-dioxane (2 mL) were then added. The resulting mixture was stirred at room temperature for 5 min, (3-phenylpropyl)zinc bromide **1a** (0.36 mmol, 1.2 equiv in 1 mL of DMA) was then added dropwise. The tube was screw capped and put into a preheated oil bath (40 ^{o}C). After stirring for 8 h, the reaction mixture was cooled to room temperature. Compound **3a** was produced in 88% yield determined by ^{19}F NMR using fluorobenzene as an internal standard.

Preparation of Ni(II) Complex [(tpy)Ni^{II}Cl$_2$] (D1)

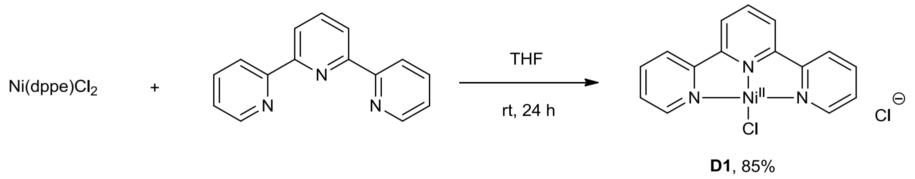

Procedure: Complex **D1** was prepared according to a modified literature prodcedure.[3] To a 100 mL of Schlenk tube were added Ni(dppe)Cl$_2$ (528 mg, 1 mmol) and terpyridine (233.3 mg, 1 mmol)

under air. The mixture was evacuated and backfilled with Ar for 3 times, 20 mL dry THF was added and the mixture was stirred at room temperature for 24 h. The tube was taken into glovebox, the resulting light green precipitate was collected and washed with THF, Et_2O, dried under high vacuum to give **D1** as a light-green powder (308 mg, 85 % yield). Anal. Calculated for $C_{15}H_{11}Cl_2N_3Ni$: C, 49.65; H, 3.06; N, 11.58. Found: C, 49.67; H, 3.31; N, 11.98.

Reaction of Ni(dppf)Cl_2 with Tpy to Obtain Complex [(tpy)$Ni^{II}Cl_2$] (D1)

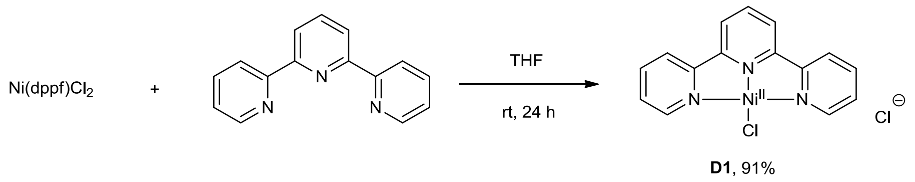

Procedure: To a 100 mL of Schlenk tube were added Ni(dppf)Cl_2 (684 mg, 1 mmol) and terpyridine (233.3 mg, 1 mmol) under air. The mixture was evacuated and backfilled with Ar for 3 times, 20 mL dry THF was added and the mixture was stirred at room temperature for 24 h. The tube was taken into glovebox, the resulting light green precipitate was collected and washed with THF until the filtrate became clear, then washed with Et_2O and dried under high vacuum to give **D1** as a light-green powder (329 mg, 91 % yield). Anal. Calculated for $C_{15}H_{11}Cl_2N_3Ni$: C, 49.65; H, 3.06; N, 11.58. Found: C, 49.40; H, 3.26; N, 12.05.

Reaction of 1a with 2a using Nickel Complex D1 as a Catalyst

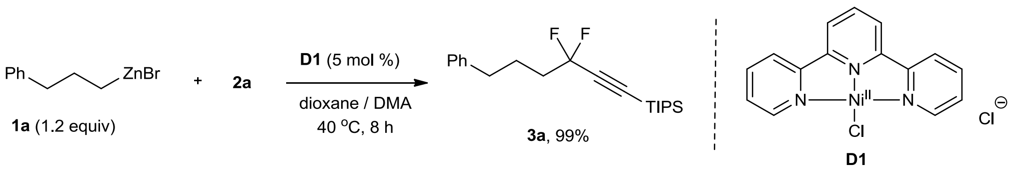

Procedure: In the glovebox, (Tpy)$NiCl_2$ (5.4 mg, 5 mol %) was added to a 25 mL of Schlenk tube. Then the tube was taken out of glovebox, *gem*-Difluoropropargyl bromide **2a** (0.3 mmol, 1.0 equiv) and 1,4-dioxane (2 mL) were then added. The resulting mixture was stirred at room temperature for 5 min, (3-phenylpropyl)zinc bromide **1a** (0.36 mmol, 1.2 equiv in 1 mL of DMA) was then added dropwise. The tube was screw capped and put into a preheated oil bath (40 °C). After stirring for 8 h, the reaction mixture was cooled to room temperature. Compound **3a** was produced in 99% yield determined by ^{19}F NMR using fluorobenzene as an internal standard.

Reaction of 1a with 2a using TpyNiCl$_2$ and dppf

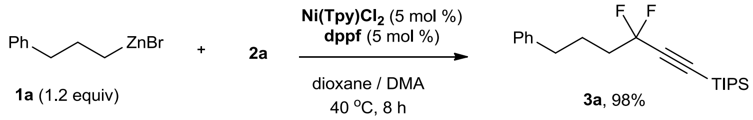

Procedure: In the glovebox, (Tpy)NiCl$_2$ (5.4 mg, 5 mol %) and dppf (8.3 mg, 5 mol %) were added to a 25 mL of Schlenk tube. Then the tube was taken out of glovebox, *gem*-Difluoropropargyl bromide **2a** (0.3 mmol, 1.0 equiv) and 1,4-dioxane (2 mL) were then added. The resulting mixture was stirred at room temperature for 5 min, (3-phenylpropyl)zinc bromide **1a** (0.36 mmol, 1.2 equiv in 1 mL of DMA) was then added dropwise. The tube was screw capped and put into a preheated oil bath (40 °C). After stirring for 8 h, the reaction mixture was cooled to room temperature. Compound **3a** was produced in 98% yield determined by ^{19}F NMR using fluorobenzene as an internal standard.

Preparation of Methyl Nickel Complex [(Tpy)NiMe] B1

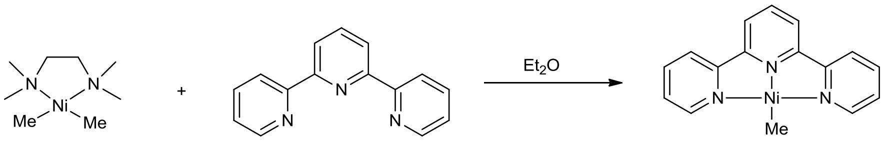

B1, 80%

Procedure: Complex **B1** was prepared according to the literature.[2] (TMEDA)Ni(Me)$_2$ (287 mg, 1.4 mmol) was dissolved in 30 mL of diethyl ether. The resulting solution was cooled to -78 ℃, and terpyridine (326 mg, 1.4 mmol) in 5 mL of anhydrous ether was added dropwise under Ar atomephere. After the addition was complete, the solution was allowed to warm to room temperature. The solution color changed from yellow-green to a dark violet/black color within 1 hour. The resulting suspension was then allowed to stir overnight. The solid was filtered and washed with ether in the glovebox to give nickel complex **B1** (344 mg, 80 % yield). Anal. Calculated for $C_{16}H_{14}N_3Ni$: C, 62.60; H, 4.60; N, 13.69. Found: C, 62.52; H, 4.54; N, 13.67.

Reaction of 1a with 2a Using Nickel Complex B1 as a Catalyst [a]

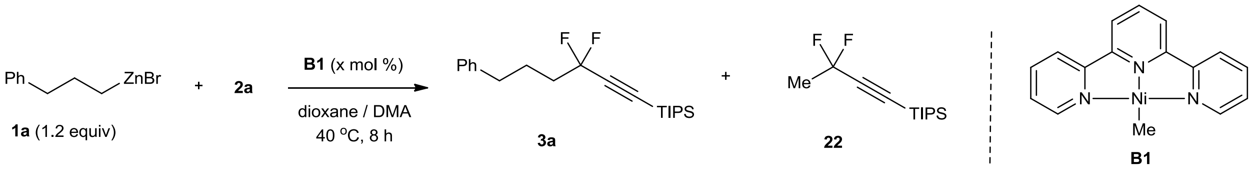

Entry	x	**3a,** Yield (%)[b]	**22,** Yield (%)[b]
1	5	95	3
2	7.5	91	6
3	10	87	9
4	20	76	17

[a]Reaction conditions (unless otherwise specified): **1a** (1.2 equiv in 1 mL of DMA), **2a** (0.3 mmol, 1.0 equiv), dioxane (2 mL). [b]Determined by ^{19}F NMR using fluorobenzene as an internal standard.

Procedure: In the glovebox, (Tpy)Ni^{I}-Me (**B1**) was added to a 25 mL of Schlenk tube. *gem*-Difluoropropargyl bromide **2a** (0.3 mmol, 1.0 equiv) and 1,4-dioxane (2 mL) were then added. The resulting mixture was stirred at room temperature for 5 min, (3-phenylpropyl)zinc bromide **1a** (0.36 mmol, 1.2 equiv in 1 mL of DMA) was then added dropwise. The tube was screw capped and put into a preheated oil bath (40 °C). After stirring for 8 h, the reaction mixture was cooled to room temperature. Data for compound **22**: ^{1}H NMR (400 MHz, $CDCl_3$) δ 1.88 (t, *J* = 17.2 Hz, 3 H), 1.09 (s, 21 H). ^{13}C NMR (126 MHz, $CDCl_3$) δ 112.1 (t, *J* = 230.0 Hz), 99.8 (t, *J* = 39.1 Hz), 88.8 (t, *J* = 5.5 Hz), 27.1 (t, *J* = 29.0 Hz), 18.4, 10.9. ^{19}F NMR (376 MHz, $CDCl_3$) δ -77.0 (q, *J* = 17.2 Hz). IR (thin film) ν_{max} 3009, 2964, 2190, 1464, 1383, 1134, 954 cm^{-1}. MS (EI): m/z (%) 246 (M^{+}), 203 (100), 175, 81. HRMS: Calculated for $C_{13}H_{24}F_2Si$: 246.1615; Found: 246.1613.

Stoichiometric Reaction of B1 with 2a

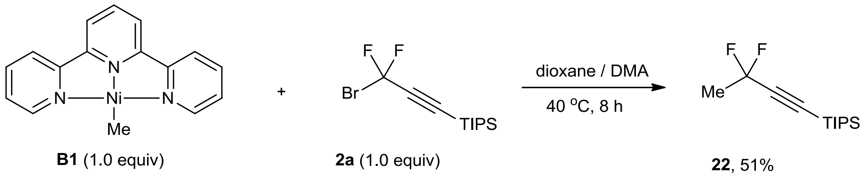

Procedure: In the glovebox, (Tpy)Ni^{I}-Me (**B1**) (30.7 mg, 0.1mmol, 1.0 equiv) was added to a 25 mL of Schlenk tube. The tube was then taken out of the glovebox, *gem*-Difluoropropargyl bromide **2a** (31.2 mg, 0.1 mmol, 1.0 equiv), 1,4-dioxane (0.68 mL) and DMA (0.32 mL) were then added. The tube was screw capped and put into a preheated oil bath (40 °C). After stirring for 8 h, the reaction

mixture was cooled to room temperature. Compound **22** was produced in 51% yield determined by ^{19}F NMR using fluorobenzene as an internal standard.

Ni(dppf)Cl_2 Catalyzed Cross-Coupling between 1a and 2a with Tpy′ as a Ligand

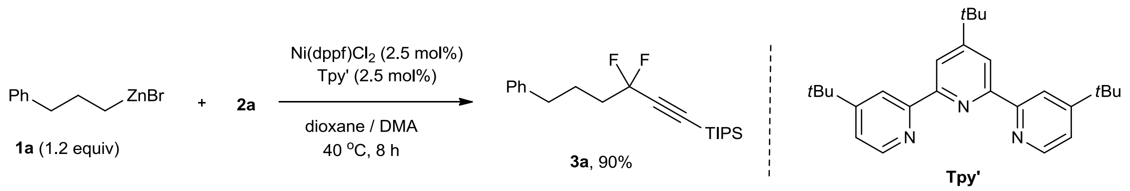

Procedure: To a 25 mL of Schlenk tube was added Ni(dppf)Cl_2 (5.2 mg, 2.5 mol %) and ligand (3.0 mg, 2.5 mol %) under air. The mixture was evacuated and backfilled with Ar for 3 times. *gem*-Difluoropropargyl bromide **2a** (0.3 mmol, 1 equiv) and 1,4-dioxane (2 mL) were added. The resulting mixture was stirred at room temperature for 5 min, (3-phenylpropyl)zinc bromide (0.33-0.45 mmol, 1.1-1.5 equiv in 1 mL of DMA) was added dropwise over a period of 5 min. The tube was screw capped and put into a preheated oil bath (40 °C). After stirring for 8 h, the reaction mixture was cooled to room temperature. Compound **3a** was produced in 90% yield determined by ^{19}F NMR using fluorobenzene as an internal standard.

Preparation of Methyl Nickel Complex [(Tpy′)Ni^{II}Me]I (C1)

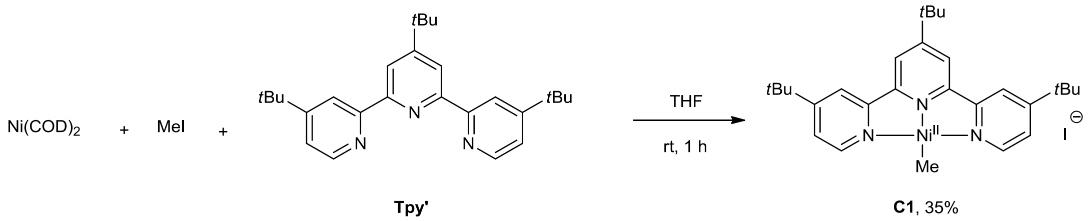

Procedure: Complex **C1** was prepared according to the literature.[4] A 1000 mL round bottom flask was charged with Ni(COD)$_2$ (802 mg, 2.9 mmol), Tpy′ (1.17 g, 2.9 mmol), and THF (250 mL). After the dark green solution was stirred for 40 minutes at room temperature, methyl iodide (415 mg, 2.9 mmol) in THF (20 mL) was added dropwise. The resulting solution was stirred at room temperature for 20 minutes. Diethyl ether (200 mL) was added, and the suspension was immediately filtered. The solid was discarded and the filtrate was stirred for an additional 30 min, at which point an orange/red solid precipitated. The solids were filtered, washed with toluene and ether, and dried under vacuum to give nickel(II) complex **C1** (611 mg, 35 % yield). The NMR data of **C1** is identical to the literature.[3] ^{1}H NMR (400 MHz, CD_2Cl_2) δ 8.28 (d, J = 6.3 Hz, 2 H), 8.14 (s, 2 H), 8.10 (s, 2 H), 7.55 (dd, J = 5.9, 1.7 Hz, 2 H), 1.56 (s, 9 H), 1.46 (s, 18 H), 0.21 (s, 3 H).

Reaction of 1a with 2a Using Nickel Complex C1 as a Catalyst

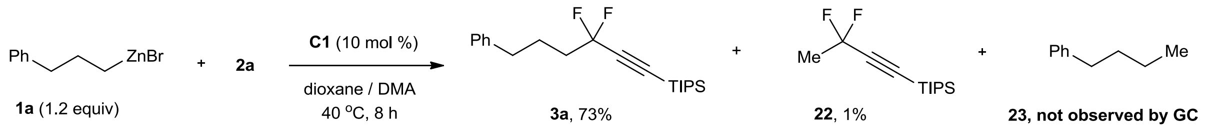

Procedure: In the glovebox, [(Tpy′)Ni^{II}Me]I (**C1**) (18.0 mg, 10 mol %) was added to a 25 mL of Schlenk tube. *gem*-Difluoropropargyl bromide **2a** (93.4 mg, 0.3 mmol, 1.0 equiv) and 1,4-dioxane (2 mL) were then added. The resulting mixture was stirred at room temperature for 5 min, (3-phenylpropyl)zinc bromide **1a** (0.36 mmol, 1.2 equiv in 1 mL of DMA) was added dropwise. The tube was screw capped and put into a preheated oil bath (40 °C). After stirring for 8 h, the reaction mixture was cooled to room temperature. ^{19}F NMR showed that compound **3a** was produced in 73% yield and compound **22** was produced in 1% yield. No compound **23** was observed by GC analysis.

Stoichiometric Reaction of C1 with 2a

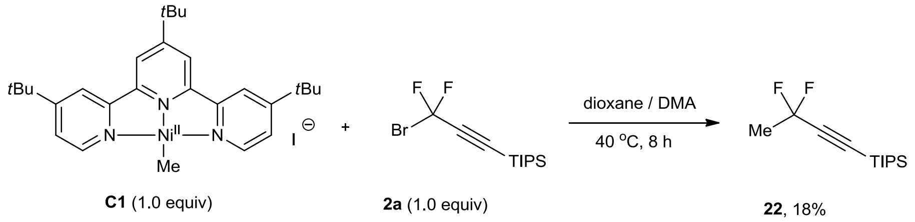

Procedure: In the glovebox, (Tpy)Ni^{I}-Me (**C1**) (30.1 mg, 0.1 mmol, 1.0 equiv) was added to a 25 mL of Schlenk tube. *gem*-Difluoropropargyl bromide **2a** (31.2 mg, 0.1 mmol, 1.0 equiv), 1,4-dioxane (0.68 mL) and DMA (0.32 mL) were then added. The tube was screw capped and put into a preheated oil bath (40 °C). After stirring for 8 h, the reaction mixture was cooled to room temperature. Compound **22** was produced in 18% yield determined by ^{19}F NMR using fluorobenzene as an internal standard.

Stoichiometric Reaction of C1 with 1a

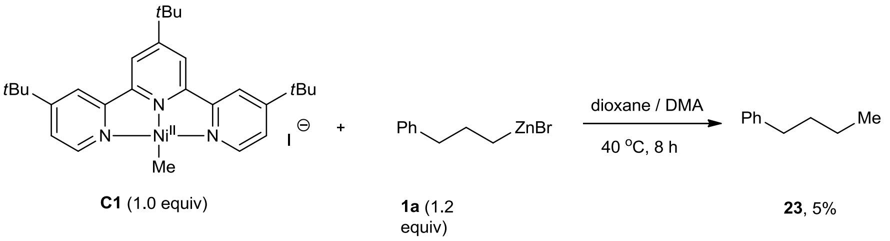

Procedure: In the glovebox, (Tpy)Ni^{I}-Me (**C1**) (30.1 mg, 0.1 mmol, 1.0 equiv) was added to a 25 mL of Schlenk tube. 1,4-Dioxane (0.68 mL) was then added and the reaction mixture was stirred at room temperature for 3 minutes. (3-Phenylpropyl)zinc bromide **1a** (0.12 mmol in 0.32 mL of DMA, 1.2 equiv) was added dropwise. The tube was screw capped and put into a preheated oil bath (40 ^{o}C). After stirring for 8 h, the reaction mixture was cooled to room temperature. The yield of compound **23** (5% yield) was determined by GC analysis using 1,3,5-trimethylbenzene as an internal standard.

Supplementary References

(1) Krasovskiy, A. & Knochel, P. Convenient titration method for organometallic zinc, magnesium, and lanthanide Reagents. *Synthesis* 890-891 (2006).

(2) Anderson, T. J., Jones, G. D. & Vicic, D. A. Evidence for a Ni^{I} active species in the catalytic cross-coupling of alkyl electrophiles. *J. Am. Chem. Soc.* **126**, 8100-8101 (2004).

(3) Suzuki, H., Matsumura, S., Satoh, Y., Sogoh, K. & Yasuda, H. Random copolymerizations of norbornene with other monomers catalyzed by novel Ni compounds involving N- or O-donated ligands. *React. Funct. Polym.* **58**, 77-91 (2004).

(4) Jones, G. D., McFarland, C., Anderson, T. J. & Vicic, D. A. Analysis of key steps in the catalytic cross-coupling of alkyl electrophiles under Negishi-like conditions. *Chem. Commun.* 4211-4213 (2005).

(5) Xu, B., Mae, M., Hong, J. A., Li, Y. & Hammond, G. B. An efficient synthesis of difluoropropargyl bromides. *Synthesis* 803-806 (2006).

(6) You, Z.-W., Jiang, Z.-X., Wang, B.-L. & Qing, F.-L. An efficient and general route to *gem*-difluoromethylenated *α,β*-unsaturated δ-lactones: High enantioselective synthesis of *gem*-difluoromethylenated goniothalamins. *J. Org. Chem.* **71**, 7261-7267 (2006).

(7) Gopinath, R. & Patel, B. K. Tetrabutylammonium tribromide (TBATB)−MeOH: An efficient chemoselective reagent for the cleavage of *tert*-butyldimethylsilyl (TBDMS) ethers. *Org. Lett.*, **2**, 4177-4180 (2000).

(8) Komiotis, D., Lim, C T., Dieter, J. P., Le Breton, G. C. & Venton, D. L. PgH2 analogs as potential antiplatelet derivatives**.** *J. Med. Chem.* **35**, 3033-3039 (1992).

(9) Sommer, H. & Fürstner, A. Stereospecific synthesis of fluoroalkenes by silver-mediated fluorination of functionalized alkenylstannanes. *Chem. Eur. J.* **23**, 558-562 (2017).

(10) Sun, W-C., Ng, C-S. & Prestwich, G. D. Synthesis of partially fluorinated analogs of (Z)-5-decenyl acetate: probes for hydrophobic interaction in pheromone reception. *J. Org. Chem.* **57**, 132-137 (1992).

(11) Xu, B. & Hammond, G. B. Difluoroallenyl bromide as a wide-ranging difluoromethylene cation equivalent: S_N2 substitution of difluoropropargyl bromide through sequential S_E2' and S_N2' reactions. *Angew. Chem. Int. Ed.* **44**, 7404-7407 (2005).

(12) Liu, J., Fang, Z., Zhang, Q., Liu, Q. & Bi, X. Silver-catalyzed isocyanide-alkyne cycloaddition: A general and practical method to oligosubstituted pyrroles. *Angew. Chem. Int. Ed.* **52**, 6953-6957 (2013).

(13) Sun, W-C., Ng, C-S. & Prestwich, G. D. Synthesis of partially fluorinated analogues of (2)-5-decenyl acetate: Probes for hydrophobic interaction in pheromone reception. *J. Org. Chem.* **57**, 132-137 (1992).
